# Saudi Clinical Practice Guideline for the Assessment and Management of Low Back Pain and Sciatica in Adults

**DOI:** 10.3390/jcm15020528

**Published:** 2026-01-08

**Authors:** Mai Aldera, Ahmed Alturkistany, Hanan Al Rayes, Gabriel Rada, Hani H. Alsulaimany, Hana I. Alsobayel, Khalid Alghamdi, Waleed Awwad, Omar A. Alyamani, Mohamed Bedaiwi, Yahya Alqahtani, Ibrahim Almaghlouth, Sami M. Bahlas, Mansour S. Alazmi, Klara Brunnhuber, Fahad Alhelal, Mansour Abdullah Alshehri

**Affiliations:** 1 Department of Rehabilitation Health Sciences, College of Applied Medical Science, King Saud University, Riyadh 11451, Saudi Arabia; maldera@ksu.edu.sa (M.A.); hsobayel@ksu.edu.sa (H.I.A.); 2 Orthopedic Section, Department of Surgery, King Faisal Specialist and Research Center, Jeddah 21499, Saudi Arabia; a.alturkistany77@gmail.com; 3 Prince Sultan Military Medical City, Riyadh 12233, Saudi Arabia; alrayesh@hotmail.com; 4 Epistemonikos Foundation, Santiago 7510321, Chile; radagabriel@epistemonikos.org; 5 Facultad de Medicina, Pontificia Universidad Católica de Chile, Santiago 7820436, Chile; 6 Department of Orthopedics, King Abdulaziz Medical City, National Guard, King Saud Bin Abdulaziz University for Health Sciences (KSAU-HS), Jeddah 21423, Saudi Arabia; hsulaimany@gmail.com; 7 Department of Orthopedic Surgery, Al-Noor Specialist Hospital, Mecca 24241, Saudi Arabia; smya1423@gmail.com; 8 King Saud University Medical City, King Saud University, Riyadh 11451, Saudi Arabia; wmawwad@gmail.com; 9 Department of Anesthesiology, King Faisal Specialist Hospital and Research Centre, Riyadh 12713, Saudi Arabia; omar.yamani@gmail.com; 10 Division of Rheumatology, College of Medicine, King Saud University, Riyadh 11451, Saudi Arabia; dr.mkbedaiwi@gmail.com; 11 Dr Sulaiman AlHabib Medical Centers, Riyadh 11564, Saudi Arabia; drqatani@yahoo.com; 12 Rheumatology Unit, Department of Medicine, King Saud University, Riyadh 11451, Saudi Arabia; ialmaghlouth@gmail.com; 13 Department of Internal Medicine/Rheumatology, King Abdulaziz University, Jeddah 22254, Saudi Arabia; drbahlas@gmail.com; 14 Prince Mohammed Medical City, Aljouf 72346, Saudi Arabia; dr_mansour2005@hotmail.com; 15 Clinical Solutions, Elsevier Limited, London EC2Y 5AS, UK; k.brunnhuber@elsevier.com; 16 Spine Surgery, Ministry of National Guard-Health Affairs, Riyadh 11426, Saudi Arabia; alhelalf@gmail.com; 17 Department of Medical Rehabilitation Sciences, Faculty of Applied Medical Sciences, Umm Al-Qura University, Mecca 24382, Saudi Arabia

**Keywords:** low back pain, LBP, sciatica, clinical practice guideline, Saudi Arabia

## Abstract

**Background/Objectives**: Low back pain (LBP) is the leading cause of disability in Saudi Arabia and contributes substantially to healthcare utilisation, reduced quality of life, and lost productivity. This guideline provides nationally standardised, evidence-based recommendations for the assessment and management of non-specific LBP and sciatica in adults, adapted to the clinical and health-system context in Saudi Arabia. **Methods**: A multidisciplinary Task Force developed the guideline using the GRADE ADOLOPMENT approach, using NICE guideline NG59 as the primary evidence source. One additional clinical question was formulated to address pain neuroscience education, informed by a relevant systematic review. Update literature searches were conducted in PubMed, Embase, and the Cochrane Library (2016–2022). The evidence was appraised using GRADE, and recommendations were formulated through structured Evidence-to-Decision deliberations and consensus voting. **Results**: The Task Force addressed eleven clinical questions in this guideline. Strong recommendations were provided for the use of validated risk assessment tools (very low certainty of evidence) and stratified management (moderate certainty of evidence). Conditional recommendations were made for indications for imaging, pharmacological treatment for sciatica, psychological interventions, multidisciplinary return to work programmes, epidural injections, prognostic value of image-concordant pathology, spinal decompression, radiofrequency denervation, and pain neuroscience education, with certainty of evidence ranging from very low to low. **Conclusions**: The findings indicate that management of non-specific LBP and sciatica in Saudi Arabia should be guided by clinical assessment, with restricted use of imaging, careful selection of pharmacological treatments, and appropriate use of psychological, multidisciplinary, and procedural interventions.

## 1. Introduction

Low back pain (LBP) is the leading global cause of years lived with disability. The Global Burden of Disease (GBD) 2021 analysis reported 619 million prevalent cases in 2020, with projections rising to 843 million by 2050, confirming LBP as the condition with the highest worldwide disability burden [1]. Findings from the GBD 2023 study show that musculoskeletal disorders, including LBP, continue to contribute substantially to noncommunicable disease morbidity, and that the burden attributable to LBP is increasing in parallel with global population ageing [2]. In Saudi Arabia, LBP has consistently ranked as the leading cause of age-standardised disability among both males and females [3]. Its impact extends beyond pain and includes reduced quality of life, increased use of healthcare services, work absenteeism, and considerable long-term socioeconomic costs [4,5,6,7]. These consequences highlight the need for nationally standardised, evidence-based clinical guidance that reflects the characteristics of the population and healthcare system in Saudi Arabia. In the absence of such guidance, clinical practice may rely on outdated protocols, non-validated assessment methods, and treatments that are not consistently aligned with current evidence. This contributes to variation in care quality and avoidable use of healthcare resources.

LBP results from the interaction of biological, psychological and social factors. Many spinal structures are capable of producing pain [8], yet routine clinical examination and diagnostic imaging rarely identify a single anatomical source. As a result, most patients are classified as having non-specific LBP. Serious spinal pathology is rare in primary care and occurs in fewer than 1% of cases [9]. Although many patients improve within weeks or months, a substantial proportion experience persistent or fluctuating symptoms, recurrent episodes, or ongoing functional limitations [8]. Sciatica usually arises from lumbar nerve root compression or irritation, which is most often caused by disc protrusion or narrowing of the spinal canal. It may be associated with radicular pain and neurological deficits that include muscle weakness, sensory disturbance, or altered reflexes [10]. This diagnostic and prognostic complexity can lead to inconsistencies in clinical decision-making, unnecessary investigations, and continued use of interventions whose effectiveness or appropriateness is not well-supported by current evidence.

Epidemiological studies conducted in Saudi Arabia show a high and persistent burden of LBP across different population groups. Community-based studies conducted before the COVID-19 pandemic reported prevalence estimates ranging from 23.8% to 38.8% [6,11,12], and one study observed an increase from 38.8% before quarantine to 43.8% during quarantine [12]. Saudi studies have also reported associations between LBP and older age, hypertension, arthritis, anaemia, osteoporosis, and previous fractures [6,11]. Stress urinary incontinence appears more common among individuals with LBP, with reported rates of 60% compared with 20% among those without LBP [13]. Vitamin D deficiency may represent an additional regional factor. One Saudi cohort found low 25-hydroxyvitamin D levels in 83% of patients with chronic LBP [14], which is consistent with wider regional findings [15,16]. High prevalence has been documented across multiple occupational and population groups, including healthcare workers, allied health professionals, educators, construction workers, and students [17,18,19,20,21,22,23,24,25,26,27,28,29,30,31,32,33,34,35].

However, much of the available Saudi epidemiological evidence is derived from cross-sectional and self-reported studies conducted in specific populations, which limits causal inference and generalisability to the broader population. Importantly, despite the documented burden of LBP, there are currently no nationally standardised care pathways describing how patients with LBP are assessed, referred, and managed across healthcare sectors in Saudi Arabia. As a result, patients may follow different routes of care across primary, secondary, and private healthcare services, with referral-based processes in the public sector potentially delaying access to interventions (e.g., physiotherapy).

This clinical practice guideline was developed by a multidisciplinary Task Force convened under the National Guidelines Programme. The Programme was established in 2021 by the Ministry of Health (MoH) and the Health Holding Company as part of the broader healthcare transformation initiative of Vision 2030. In 2022, responsibility for national guideline development was transferred to the National Centre for Evidence-Based Medicine at the Saudi Health Council [36]. This structure ensures the use of standardised, transparent, and methodologically robust development processes designed to enhance clinical consistency, reduce unwarranted variation, and support the delivery of high-quality, evidence-based care across healthcare sectors. Within this framework, the present guideline aims to replace heterogeneous or outdated approaches with clear, updated and locally relevant recommendations that discourage low-value or non-validated practices and support more rational and efficient use of diagnostic and therapeutic options for LBP in Saudi Arabia.

### 1.1. Scope

This guideline was adapted from the National Institute for Health and Care Excellence (NICE) guideline NG59, titled “Low back pain and sciatica in over 16 s: assessment and management” [37]. The Task Force selected, updated, and adapted ten clinical questions from this NICE guideline to generate recommendations relevant to key areas of assessment and management in Saudi Arabia. Such areas include the use of risk assessment and clinical prediction tools, imaging, pharmacological and psychological therapies, multidisciplinary and return to work interventions, epidural injections, prognostic significance of image-concordant pathology, and the role of locally invasive procedures or surgery. An additional question addresses pain neuroscience education, based on a systematic review [38]. This guideline focuses on adults with non-specific LBP and sciatica. It does not address serious spinal pathology such as infection, malignancy or fracture, inflammatory causes of LBP or sciatica with progressive neurological deficit, or suspected cauda equina syndrome.

### 1.2. Objectives

The objective of this guideline is to support consistent, high-quality, and evidence-based clinical decision-making for adults with non-specific LBP or sciatica within the Saudi healthcare system. By adapting and contextualising NICE guideline NG59, the guideline ensures that assessment and management strategies are informed by current international evidence while reflecting clinical, organisational, and population characteristics relevant to Saudi Arabia. Specifically, the guideline aims to promote evidence-based and patient-centred care, reduce unwarranted variation in assessment and management, improve consistency across healthcare sectors, and align clinical practice with national healthcare transformation priorities. The recommendations are designed to provide clear and practical guidance while allowing for individualised management based on each patient’s clinical presentation, preferences, and circumstances. In addition, the guideline seeks to encourage a shift away from interventions with limited or uncertain benefit toward approaches supported by stronger evidence of effectiveness and safety.

### 1.3. Target Population

This guideline applies to adults aged 16 years and older who present with non-specific LBP or sciatica in primary, secondary, or tertiary care settings in Saudi Arabia. The lower age threshold of 16 years was adopted to ensure methodological consistency with the source guideline used for adaptation, NICE Guideline NG59, which defines its target population for LBP and sciatica as individuals aged 16 years and older. Age 16 is used as the clinical transition point, marking entry into adult LBP care pathways. It does not cover serious spinal pathology, inflammatory back pain or sciatica accompanied by progressive neurological deficit, or suspected cauda equina syndrome. The guideline may also assist individuals with LBP, as well as their families and caregivers, in understanding recommended approaches to assessment and management.

### 1.4. End-Users

The guideline is intended for healthcare professionals involved in the management of adults with LBP. These include primary care physicians, neurologists, neurosurgeons, spine surgeons, orthopaedic surgeons, rheumatologists, emergency physicians, internists, physiotherapists, psychologists, pain management specialists, nurses, and allied health practitioners. It is also relevant to administrators and policymakers responsible for planning and organising LBP-related healthcare services in Saudi Arabia.

### 1.5. How to Use This Guideline

This guideline is designed to support clinicians and patients in making evidence-based decisions regarding the assessment and management of LBP. As in all guidelines developed using the GRADE approach, recommendations should be interpreted together with the accompanying explanatory statements. These statements describe the key considerations that informed the Task Force’s judgements, including anticipated benefits and harms, patient values and preferences, feasibility and acceptability, resource implications, and equity considerations.

The recommendations are not intended to function as rigid standards of care or as a uniform approach applicable to all patients. Clinical variation is expected due to the heterogeneous nature of LBP presentations and differences across care settings. Healthcare professionals should integrate the recommendations with their clinical expertise and with each patient’s preferences, comorbidities, expectations, and goals. Applying the recommendations in this manner supports the delivery of safe, effective evidence-based, and patient-centred care across the Saudi healthcare system.

## 2. Materials and Methods

### 2.1. Guideline Development Framework

This national guideline was developed using the GRADE-ADOLOPMENT methodology [39], an internationally recognised and rigorously defined approach that supports the adoption, adaptation and, when required, de novo development of recommendations. This methodology was selected to ensure that the final recommendations reflect the best available international evidence while remaining responsive to the clinical, cultural, and health-system context of Saudi Arabia. In line with this approach, the Task Force began by identifying high-quality source guidelines and systematic reviews suitable for adaptation. The NICE guideline NG59 [37] was selected as the primary source guideline over other available guidelines based on its methodological quality, scope, and international relevance. This selection was made following a structured vote among Task Force members, with written justifications documented to support the final decision. One additional clinical question concerning pain neuroscience education was incorporated using a systematic review [38].

### 2.2. Organisation and Governance

Guideline development was overseen by the Task Force, a multidisciplinary group of local experts in LBP representing rheumatology, orthopaedic and spine surgery, physical therapy, rehabilitation, and anaesthesiology. Members were drawn from the MoH, academic institutions, military hospitals, and National Guard facilities. This composition ensured broad clinical expertise, regional representation and an understanding of health-system variation across Saudi Arabia. Several members had advanced training in epidemiology and clinical guideline methodology, which strengthened the methodological integrity of the development process. All Task Force members successfully completed Level 1 of the Clinical Group or Panel Member course within the INGUIDE programme, authorised by McMaster University, ensuring a shared foundation in evidence-based guideline development. The Task Force was supported by an international Guideline Support Team based at, or contracted by, Elsevier. The Support Team provided methodological guidance; conducted all the literature searches; performed data screening and extraction; prepared GRADE evidence profiles; developed Evidence-to-Decision (EtD) frameworks; and assisted with dissemination and early implementation planning. Collaboration between the Task Force and the Support Team was maintained through structured and frequent communication.

### 2.3. Selection of Clinical Questions and Prioritisation of Outcomes

The scope of the guideline was defined through a systematic search for potential source guidelines, followed by structured appraisal using the AGREE II instrument [40]. The NICE guideline was selected as the principal source based on its methodological quality and relevance. Task Force members subsequently completed an online survey in which they rated the importance of proposed clinical questions on a nine-point scale. Ten clinical questions were selected from NICE guideline NG59 [37]. An additional question on pain neuroscience education was added at the request of the Task Force, resulting in a total of eleven clinical questions.

Outcome identification and prioritisation were undertaken through an iterative process that combined online surveys with facilitated workshops. These workshops, held on 21 March and 13 April 2022, enabled the Task Force to distinguish between outcomes considered critical for decision-making and those regarded as important but not critical. Prioritised outcomes included those defined in the source guideline and additional outcomes identified by the Task Force as clinically relevant in the Saudi context. Decisions throughout the scoping and prioritisation stages were reached by consensus.

### 2.4. Search Strategy

The evidence for all clinical questions was updated through comprehensive literature searches conducted by the Guideline Support Team between 27 April and 19 May 2022. These searches replicated, as closely as possible, the strategies used in NICE guideline NG59 [37] and the systematic review on pain neuroscience education [38], ensuring methodological continuity with the source documents. Searches were conducted in PubMed, Embase, and the Cochrane Library, covering 1 January 2016 to 4 May 2022 for PubMed and Embase, and January 2016 to May 2022 for Cochrane.

High-sensitivity strategies were developed that combined controlled vocabulary terms (e.g., MeSH and Emtree) with text words and incorporated validated methodological filters relevant to LBP, sciatica and risk assessment or prognostic tools. Search strategies were adapted only to account for updated database interfaces, indexing changes, and the extended time window. Full search strategies for each clinical question, including all PubMed, Embase, and Cochrane search blocks, are provided in Supplementary Material S1.

All citations were imported into a reference management system and deduplicated before screening to ensure complete coverage of the evidence base without redundancy, which was particularly important given overlapping database scopes. Searches were configured to retrieve randomised controlled trials (RCTs), cohort studies, non-randomised studies, and systematic reviews involving adults aged 16 years or older with non-specific LBP or sciatica.

In addition, the Support Team conducted targeted searches using PubMed and Embase. Searches for epidemiological data were conducted between 1 April 2017 and 28 April 2022, focusing on incidence and prevalence of interventions and outcomes relevant to the clinical questions. Additional searches were conducted between 28 April and 5 May 2022 to identify local and regional evidence related to contextual factors, such as patient values and preferences, equity, feasibility, acceptability, implementation issues, and costs (Supplementary Material S1).

### 2.5. Study Selection

After deduplication, search results for the clinical questions were uploaded to the Cadima platform. Two reviewers independently screened all records. Screening occurred in two stages. First, titles and abstracts were assessed against predefined inclusion and exclusion criteria aligned with the PICO specifications for each clinical question. Studies meeting or potentially meeting criteria were retrieved in full text. Second, full texts were independently assessed by both reviewers. Disagreements were resolved through structured discussion to ensure consistent and transparent decision-making. The same rigorous processes were utilised for the appraisal of search results on contextual factors.

### 2.6. Data Extraction and Risk of Bias Assessment

All included studies underwent detailed data extraction using prespecified structured templates. These templates captured study design, participant characteristics, intervention and comparator descriptions, outcome definitions, numerical results, subgroup analyses, follow-up periods and any methodological limitations. Embedded fields corresponding to each domain of the relevant risk of bias tool ensured systematic evaluation of randomisation, allocation concealment, blinding, outcome assessment, attrition, reporting completeness, and other potential sources of bias. One reviewer performed extraction and a second reviewer independently verified all entries. Risk of bias assessments were conducted using domain-based frameworks aligned with Cochrane guidance and the criteria applied in the source guideline. All assessments were completed independently by two reviewers, with disagreements resolved through discussion.

### 2.7. Evidence Synthesis

Extracted numerical data were transferred to RevMan (version 5.4; The Cochrane Collaboration, Copenhagen, Denmark), this software used to generate effect estimates, forest plots, and summary tables. The use of RevMan ensured consistency with the NICE guideline development process. For each clinical question, meta-analysis was performed when studies were sufficiently homogeneous in population, intervention, outcomes, and methods. When meta-analysis was not feasible, structured narrative synthesis was undertaken. RevMan outputs, including forest plots, risk of bias figures and effect estimates, were imported into the GRADEpro platform (McMaster University and Evidence Prime) to produce Summary of Findings tables and detailed GRADE evidence profiles. These profiles were then reviewed by a second Support Team member to confirm internal accuracy and consistency.

Findings from included studies on contextual factors, which included systematic reviews and regional primary studies, were summarised narratively to support the interpretation and applicability of guideline recommendations.

### 2.8. Certainty of Evidence

Certainty of evidence for each outcome was assessed using the GRADE framework, which evaluates risk of bias, inconsistency, indirectness, imprecision, and publication bias [39]. Certainty was classified as high, moderate, low, or very low [41]. High certainty indicates strong confidence that the true effect is close to the estimate. Moderate certainty reflects moderate confidence, with the true effect likely to be similar. Low certainty indicates limited confidence, with the true effect potentially differing substantially. Very low certainty indicates very limited confidence and a high likelihood that the true effect differs markedly from the estimate.

### 2.9. Development of Recommendations

Recommendations were developed during seven online meetings held between December 2022 and May 2023. Before each meeting, Task Force members reviewed the evidence and proposed EtD judgements via PanelVoice surveys in GRADEpro. During the meetings, the methodologist co-chair presented key evidence, and the Task Force reviewed all EtD criteria. Judgements were finalised through discussion or, when necessary, by a 60% vote, with differing views recorded. After completing all judgements for a clinical question, the Task Force voted on the final recommendation.

The complete guideline underwent external peer review by independent experts. After revisions based on their feedback, the guideline was submitted to the Scientific Committee of the Saudi Health Council, which approved and formally endorsed it as a national guideline.

Recommendations were categorised as strong or conditional in accordance with the GRADE approach. Strong recommendations reflect high confidence that desirable effects outweigh undesirable effects for most patients. Conditional recommendations reflect situations in which the balance depends more heavily on patient preferences, clinical context, or lower certainty. When evidence was insufficient to judge the balance of effects, the Task Force issued no recommendation for or against the intervention. Recommendations against a given option were made when the overall harms were judged to outweigh the benefits, with the strength reflecting the certainty of evidence and the extent to which clinicians should avoid its use. The predetermined consensus threshold was 60%, consistent with thresholds reported in the guideline development literature and applied uniformly across all clinical questions.

## 3. Results

**Figure 1:**
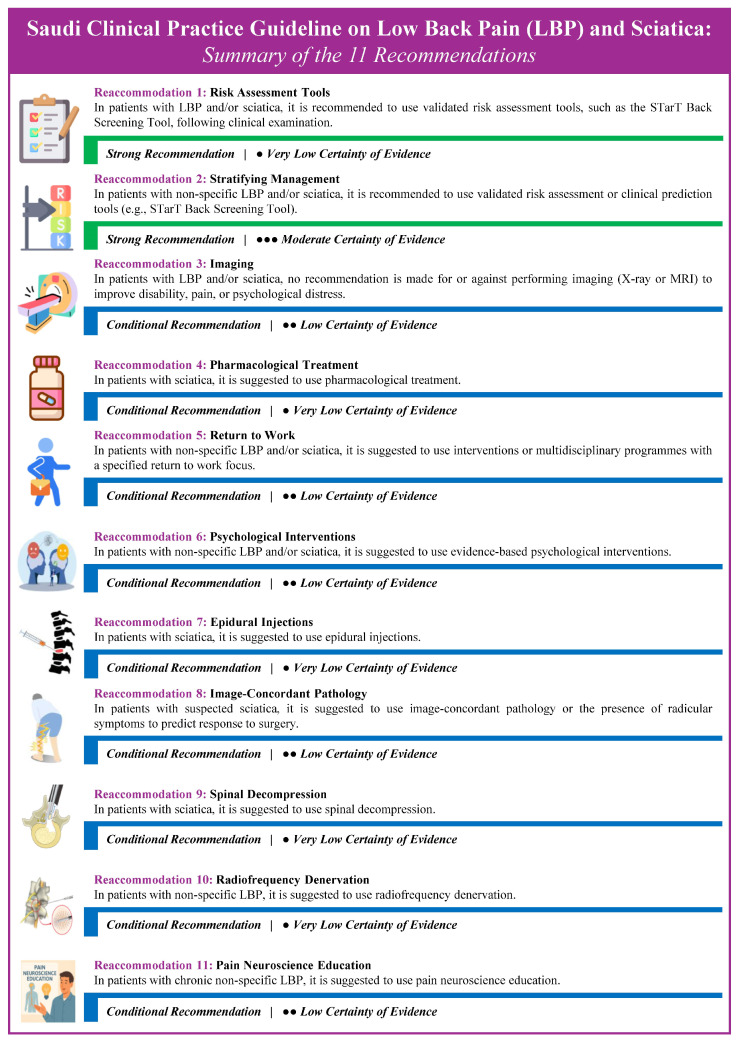
Summary of the 11 recommendations from the Saudi clinical practice guideline for the assessment and management of low back pain and sciatica in adults. Each recommendation is displayed with its classification (strong or conditional) and the corresponding GRADE certainty of evidence (high, moderate, low, or very low).

This section summarises 11 national guideline recommendations in Saudi Arabia, each structured by clinical question, recommendation, evidence summary, benefits and harms, certainty of evidence, and contextual factors. Quantitative results are presented in the accompanying tables, while forest plots, including effect estimates and risk of bias assessments for each meta-analysis, are provided in Supplementary Material S2. Figure 1 presents a summary of the 11 recommendations, including the classification of each recommendation and the corresponding certainty of evidence.

### 3.1. Risk Assessment Tools

#### 3.1.1. Question

Should validated risk assessment tools versus no validated risk assessment tools be used for screening patients with LBP and/or sciatica who are at risk of poor outcome or delayed improvement?

#### 3.1.2. Recommendation

In patients with LBP and/or sciatica, the Task Force recommends using validated risk assessment tools, such as the STarT Back Screening Tool (SBST), following clinical examination (*strong recommendation*, *very low certainty of evidence*). Although the certainty of the supporting evidence is very low, the Task Force judged that the clear net clinical benefit and the appropriateness of this intervention in the Saudi context justify a strong recommendation. Validated risk assessment tools should be considered at the first clinical encounter to help identify patients who may need further treatment, including those who return for subsequent consultations. These tools are intended to support, rather than replace, clinical decision-making.

#### 3.1.3. Evidence Summary

The NICE guideline [37] identified ten observational studies [42,43,44,45,46,47,48,49,50,51] and conducted a meta-analysis that compared the effects of using validated risk assessment tools after clinical examination versus not using such tools for screening patients with LBP and/or sciatica who are at risk of poor outcomes or slow recovery. The update search identified no additional eligible studies. Evidence was available for discrimination, reported using area under the curve (AUC) and sensitivity and specificity, and for calibration, reported using R-squared (R^2^) values, for the outcomes of pain and function. No evidence on reclassification was identified. All studies were conducted in an LBP population, two of which included mixed samples of patients with or without additional sciatica. The risk assessment tools evaluated in the included studies are listed in Supplementary Material S3.

#### 3.1.4. Benefits and Harms

The included studies assessed the prognostic performance of validated risk assessment tools, with evidence available for discrimination (AUC, sensitivity, specificity) and, for some tools, calibration (R^2^). Across primary and secondary care settings, the SBST [42,43,46,47,48,49,51] was judged to be a reasonably useful risk assessment tool, consistently demonstrating moderate to high discrimination for predicting pain and functional outcomes at 3, 6, and 12 months. Reported AUC values generally ranged from approximately 0.66 to 0.82, with one primary care study reporting 80% sensitivity and 65% specificity for functional improvement. The NICE guideline similarly reported moderate calibration. Evidence for other tools was very uncertain, but some findings suggested moderate discrimination for selected outcomes. The Chronic Pain Risk Item Set [43] demonstrated moderate discrimination for predicting pain. The Low Back Pain Perception Scale [45] showed low discrimination for recovery, despite reporting reasonable calibration metrics. The modified Örebro Musculoskeletal Pain Questionnaire [44] demonstrated moderate discrimination for problem severity, although discrimination was poor at lower thresholds and calibration performance was weak. The Oswestry Disability Index (ODI) [50] showed high discrimination for predicting functional improvement at 1 month. Further details are summarised in Table 1.

#### 3.1.5. Certainty of Evidence

The overall certainty in the evidence of effects was judged to be very low, based on the lowest certainty rating across the critical outcomes. This was due to very serious risk of bias in the included studies and serious imprecision in the effect estimates.

#### 3.1.6. Contextual Factors

No direct evidence was identified for any contextual factor domains, so judgements relied on Task Force experience (Table 2). Patients may have greater confidence in imaging than questionnaires, creating uncertainty and variability in values and preferences. Resource requirements were judged minimal due to low item costs, brief training, and short application time, and the early identification of patients is likely to benefit and may support favourable cost-effectiveness. Implementation across settings with negligible expense may improve equity, and the tools were considered simple, acceptable to clinicians and decision-makers, and feasible to integrate into routine care.

### 3.2. Stratifying Management

#### 3.2.1. Question

Should validated risk assessment or clinical prediction tools, compared with no tools or with each other, be used to stratify the management of patients with non-specific LBP and/or sciatica based on the outcome of the tool or questionnaire?

#### 3.2.2. Recommendation

In patients with non-specific LBP and/or sciatica, the Task Force recommends using validated risk assessment or clinical prediction tools (e.g., SBST) (*strong recommendation*, *moderate certainty of evidence*). Some tools, including SBST, stratify patients according to their risk of chronicity and disability, and the Task Force agreed that intervention protocols should incorporate this stratification.

#### 3.2.3. Evidence Summary

The NICE guideline [37] identified two RCTs [52,53] comparing stratified management (using SBST) with non-stratified care. The update search found no additional eligible trials. The Task Force identified one further RCT (MATCH) for inclusion [54]. The evidence showed no clinically important difference between stratified care and usual care for most outcomes, including pain, function, mental quality of life, and psychological distress. However, improvement in the physical component score of the 12-Item Short Form Survey (SF-12) was observed in favour of stratified care at >4 months. A detailed description of the stratification tools evaluated in the included studies is provided in Supplementary Material S3.

#### 3.2.4. Benefits and Harms

The evidence for this question comes from two RCTs included in the NICE guideline [37] and one additional trial identified by the Task Force [52]. These studies evaluated the use of the SBST to guide stratified management for non-specific LBP and sciatica. The evidence suggests that stratified care increases physical quality of life, with one trial reporting a significant improvement in the SF-12 physical component score [52]. For mental quality of life (SF-12), pain severity (Visual Analog Scale [VAS]), psychological distress (Hospital Anxiety and Depression Scale [HADS]), and functional status (Roland–Morris Disability Questionnaire [RMDQ]), the evidence probably results in little to no difference compared with non-stratified care [52,54]. The evidence is very uncertain regarding the effect of stratified care on healthcare utilisation (prescribing, investigations, hospitalisation, or health-professional visits), primarily due to wide confidence intervals and imprecision, and the available data showed no clear reduction in prescribing, investigations, hospitalisation, or health-professional visits [53]. All effect estimates are summarised in Table 3.

#### 3.2.5. Certainty of Evidence

The overall certainty in the evidence of effects was judged to be moderate, based on the lowest certainty rating across the critical outcomes, due to serious imprecision.

#### 3.2.6. Contextual Factors

No direct evidence was identified for any contextual factor domains apart from the cost-utility analyses reported in the NICE source guideline, so judgements otherwise relied on the Task Force’s experience (Table 4). There were important uncertainty and variability in patients’ values and preferences. Resource requirements were considered minimal because validated risk assessment and clinical prediction tools incur no additional costs and can be delivered by clinicians. Two cost-utility analyses reported in the NICE source guideline [55,56] found that stratification-based intervention using SBST dominated usual care, indicating favourable cost-effectiveness. Given Saudi Arabia’s comprehensive health coverage, implementing these tools may enhance equity by supporting more efficient distribution of services. The tools were also regarded as acceptable to patients, clinicians, and decision-makers, and feasible to implement consistently across routine healthcare settings.

### 3.3. Imaging

#### 3.3.1. Question

Should imaging (plain radiography [X-ray] or magnetic resonance imaging [MRI]) versus no investigation be used to improve functional disability, pain, or psychological distress in patients with LBP and/or sciatica?

#### 3.3.2. Recommendation

In patients with LBP and/or sciatica, the Task Force does not make a recommendation for or against performing imaging (X-ray or MRI) to improve functional disability, pain, or psychological distress (*conditional recommendation*, *low certainty of evidence*). Although patients often seek imaging for reassurance, imaging findings (e.g., disc and joint degeneration) rarely confirm or refute clinical impressions and frequently reveal changes that are also observed in asymptomatic individuals. The Task Force agreed that imaging should be undertaken only when red flags are present or when the results are likely to influence subsequent management decisions. Patients with additional medical conditions (e.g., cancer, osteoporosis, previous spinal fracture, recent infection unexplained significant weight loss, or suspected osteomyelitis) or a history of malignancy may require imaging to exclude serious pathology. When red flags are present, imaging should be performed within specialist settings. Referrals to specialist care should be made for the purpose of obtaining a clinical assessment rather than with the expectation that imaging will necessarily be performed. In circumstances where imaging is unlikely to alter management, individuals may be more willing to accept the decision not to undergo imaging when this is advised by specialist clinicians.

#### 3.3.3. Evidence Summary

The NICE clinical practice guideline [37] described several imaging modalities available for the assessment of spinal pathology. X-ray imaging is inexpensive and readily accessible but provides limited information on soft tissue and is mainly useful for fracture follow-up and assessment of alignment. MRI provides detailed information on spinal cord, discs, and ligaments without radiation exposure but remains relatively costly. Imaging should be guided by clinical assessment rather than used as a screening tool. Three RCTs [57,58,59] were identified by the NICE clinical practice guideline [37] evaluating the effects of imaging versus no imaging on outcomes >4 months in patients with LBP with or without sciatica. Two trials compared X-ray with no imaging, and one compared MRI with no imaging. The update search identified no additional studies.

#### 3.3.4. Benefits and Harms

RCTs compared imaging with no imaging in adults with LBP with or without sciatica at >4 months to 1 year. The evidence suggests that imaging improves health-related quality of life, specifically the SF-36 bodily pain and SF-36 mental health subscales [57,58], and improved pain severity on the Aberdeen Low Back Pain Score in one study [57]. Imaging also increases healthcare utilisation, including repeat imaging and outpatient consultations [57,59]. In contrast, the evidence probably results in little to no difference in function, the SF-36 physical functioning subscale, and psychological distress measured using the HADS anxiety and depression subscales [57,58]. Other healthcare utilisation outcomes (including hospital admission, prescribed drugs, referrals, and primary-care consultations) also probably results in little to no difference, although the findings were characterised by wide uncertainty [57,58,59]. No adverse events were reported in any of the included studies. All effect estimates are summarised in Table 5.

#### 3.3.5. Certainty of Evidence

The overall certainty in the evidence of effects was judged to be low based on the lowest certainty rating across critical outcomes, due to serious risk of bias, serious inconsistency, and very serious imprecision.

#### 3.3.6. Contextual Factors

Direct evidence for contextual factors was limited to resource use and cost-effectiveness; all other judgements relied on the Task Force’s experience only (Table 6). For values and preferences, the Task Force judged that there was probably no important variability in how patients prioritise outcomes, and that patient assessments of outcomes are likely similar. One study [60] showed that imaging increases costs, healthcare utilisation and possibly work absenteeism, and MoH data indicated that MRI is substantially more expensive than X-ray (Supplementary Material S4). A cost-utility analysis reported in the source guideline [57] found that early imaging increased costs but also improved quality-adjusted life years (QALYs), though with important limitations. Overall, the Task Force concluded that imaging entails moderate costs and that cost-effectiveness may favour comparison strategies rather than routine early imaging. MRI availability is limited in rural and vulnerable regions, whereas radiography is widely accessible, suggesting that imaging would probably increase equity. Imaging was considered acceptable to patients, clinicians and decision-makers, with X-ray feasible in most settings and MRI less feasible due to specialist requirements.

### 3.4. Pharmacological Treatment

#### 3.4.1. Question

Should pharmacological treatment versus placebo or usual care/waiting list, or one or more pharmacological interventions compared with each other, be used in patients with sciatica?

#### 3.4.2. Recommendation

In patients with sciatica, the Task Force suggests using pharmacological treatment (*conditional recommendation*, *very low certainty of evidence*). The Task Force noted that the NICE Guideline Development Committee did not identify clear clinical benefit for any specific pharmacological agent and did not recommend one particular medicine as first-line treatment. Instead, non-pharmacological and invasive options should be considered as the basis for managing sciatica where appropriate. For individual drug classes, the NICE Guideline Development Committee made the following judgements [37], which are summarised in Table 7.

#### 3.4.3. Evidence Summary

The source guideline identified six RCTs [61,62,63,64,65,66] and conducted a meta-analysis comparing pharmacological treatment with placebo, usual care or waiting list, or comparing active treatments with each other in patients with LBP and/or sciatica. The update search identified two additional studies [67,68]. Trials evaluated four main comparisons: non-steroidal anti-inflammatory drugs (NSAIDs) versus placebo, benzodiazepines versus placebo, gabapentinoids versus placebo, and oral corticosteroids versus placebo. No RCTs were identified for paracetamol, opioids, antidepressants, nefopam, antiepileptic drugs other than gabapentinoids, or muscle relaxants other than benzodiazepines. Across studies, sample sizes ranged from 50 to over 500 participants and treatment durations varied from a few days to several weeks. The evidence base was limited by small numbers of trials per comparison, heterogeneity in drug regimens and outcomes, and imprecision of estimates.

#### 3.4.4. Benefits and Harms

RCTs compared NSAIDs, benzodiazepines, gabapentinoids and oral corticosteroids with placebo for the management of sciatica [61,62,63,64,65,66,68]. The evidence suggests that NSAIDs probably result in little to no difference in pain severity (VAS) compared with placebo and may increase adverse events, with dyspepsia, nausea, abdominal pain, diarrhoea and one episode of anaphylactic shock reported [63,67]. Benzodiazepines resulted in worse outcomes for responder-defined pain relief (VAS) compared with placebo [62]. Gabapentinoids improved rest pain in one study but overall probably result in little to no difference in pain (Numeric Rating Scale [NRS]) or function (RMDQ) compared with placebo. They were associated with increased adverse events, although the evidence was very uncertain in some studies, with dizziness, somnolence, fatigue, reduced salivation, concentration difficulties, and increased sleep reported [61,65,68]. Corticosteroids probably result in little to no difference in function (ODI) or pain (NRS) compared with placebo, and increased adverse events, with insomnia, sweating, increased appetite, and nervousness were commonly reported [66]. All effect estimates are summarised in Table 8.

#### 3.4.5. Certainty of Evidence

The overall certainty in the evidence of effects was judged to be very low based on the lowest certainty in the evidence for the critical outcomes due to serious risk of bias, serious inconsistency, and very serious imprecision.

#### 3.4.6. Contextual Factors

No direct evidence was identified for values, resource use, cost-effectiveness, equity, or feasibility, so judgements relied on the Task Force’s experience (Table 9). The Task Force considered that there was probably no important variability in patients’ values and preferences. Information from the Centre of Health Technology Assessment (MoH) indicated that medication costs per package are modest, and no evidence on resource requirements was identified. A cost-utility analysis comparing electroacupuncture with NSAIDs for chronic LBP [69] suggested greater cost-effectiveness for electroacupuncture, although this evidence is indirect for sciatica. Overall, the Task Force judged pharmacological treatment to involve negligible costs, with cost-effectiveness probably favouring its use when indicated. No evidence on equity was identified; given Saudi Arabia’s comprehensive health coverage, pharmacological treatment is unlikely to affect equity. Evidence on muscle relaxants [70] and antidepressants [71] indicated small or uncertain benefits and increased adverse events, but treatment was judged probably acceptable and feasible in routine care.

### 3.5. Return to Work

#### 3.5.1. Question

Should interventions or multidisciplinary programmes with a specified return to work focus (or including ergonomic interventions) versus placebo, usual care or waiting list, or versus one or more interventions compared with each other or other non-invasive interventions (either alone or in combination) be used in patients with non-specific LBP and/or sciatica?

#### 3.5.2. Recommendation

In patients with non-specific LBP and/or sciatica, the Task Force suggests using interventions or multidisciplinary programmes with a specified return to work focus (or including ergonomic interventions) (*conditional recommendation*, *low certainty of evidence*).

#### 3.5.3. Evidence Summary

The NICE guideline [37] identified three RCTs [72,73,74] and conducted a meta-analysis comparing the effects of interventions or multidisciplinary programmes with a specified return to work focus (or including ergonomic interventions) versus placebo, usual care or waiting list, or versus one or more interventions compared with each other, or other non-invasive interventions (either alone or in combination) in patients with non-specific LBP and/or sciatica. The update search identified no additional studies for inclusion. All three studies reported multidisciplinary programmes. In one trial [72], participants were initially randomised to a multidisciplinary return to work programme or usual care (primary randomisation), and those who remained sick-listed at 8 weeks underwent a secondary randomisation to either a unidisciplinary graded activity programme or usual care.

#### 3.5.4. Benefits and Harms

RCTs of multidisciplinary programmes with a specified return to work focus, or including ergonomic components, suggest that these interventions improve days to return to work and return to work rates at ≤4 months [72], function (RMDQ) at >4 months [73], and several healthcare utilisation outcomes, including reduced physiotherapy, manual therapy, and medical specialist visits, and lead to fewer diagnostic tests and lower use of LBP medications [72]. One study indicated that they may improve return to work at >4 months [74]. These programmes probably result in little to no difference in quality of life (EuroQol 5-Dimension [EQ-5D]), function (RMDQ) and pain (NRS) at ≤4 months [72], and in occupational-physician and general-practitioner utilisation at the same time point [72]. For pain (NRS) [73,74] and psychological distress (Beck Depression Inventory [BDI]) [74] at >4 months, these programmes also probably result in little to no difference. All effect estimates are summarised in Table 10.

#### 3.5.5. Certainty of Evidence

The overall certainty in the evidence of effects was judged to be low based on the lowest certainty in critical outcomes, reflecting serious risk of bias and very serious imprecision.

#### 3.5.6. Contextual Factors

Direct evidence was available for values, resource use and cost-effectiveness, and acceptability, whereas equity and feasibility relied primarily on Task Force experience (Table 11). A systematic review [75] reported that multidisciplinary biopsychosocial rehabilitation is effective for non-specific LBP, although further research is required to establish its cost-effectiveness and impact on sick leave. Based on this evidence and their experience, the Task Force concluded that patients’ values and preferences were probably not subject to important variability. Two RCTs [72,73] included in the NICE guideline [37] conducted within-trial economic evaluations [76,77], which found that multidisciplinary return to work interventions incurred slightly higher direct costs than usual care but reduced overall and indirect costs, supporting favourable cost-effectiveness. No evidence on equity was identified; however, the Task Force judged that equity would likely improve with appropriate resource allocation, while noting that disparities may persist in rural areas. A pilot study [60] found integrated care programmes to be acceptable and feasible, with time investment identified as the principal implementation barrier. Overall, the Task Force considered these programmes acceptable to patients, clinicians, and decision-makers and generally feasible to implement, with some context-specific challenges.

### 3.6. Psychological Interventions

#### 3.6.1. Question

Should psychological interventions (behavioural therapies, cognitive therapies, cognitive–behavioural approaches, mindfulness, and acceptance and commitment therapy) versus placebo or usual care/waiting list, or versus one or more interventions compared with each other or other non-invasive interventions (either alone or in combination), be used in patients with non-specific LBP and/or sciatica?

#### 3.6.2. Recommendation

In patients with non-specific LBP and/or sciatica, the Task Force suggests the use of evidence-based psychological interventions (*conditional recommendation*, *low certainty of evidence*). In line with the NICE guideline [37], psychological therapies using a cognitive–behavioural approach for managing LBP with or without sciatica should only be used as part of a treatment package that includes exercise with or without manual therapy (spinal manipulation, mobilisation, or soft tissue techniques such as massage).

#### 3.6.3. Evidence Summary

The NICE guideline [37] identified 11 RCTs [78,79,80,81,82,83,84,85,86,87,88] and conducted a meta-analysis comparing the effects of psychological interventions versus placebo, usual care/waiting list, or other non-invasive interventions in patients with non-specific LBP and/or sciatica. A contributing factor to the predominantly low to very low certainty ratings was the difficulty of achieving adequate blinding in trials of psychological interventions. Several studies used waiting list control groups, which do not reflect usual practice and may inflate effect sizes because individuals assigned to delayed treatment often experience negative expectations. In addition, many trials provided insufficient detail regarding the background care received by participants outside the intervention, making it difficult to determine whether co-interventions were comparable between groups. This limitation increases the risk of overestimating effects for subjective outcomes such as pain and function. The update search identified two further studies [89,90] for inclusion.

#### 3.6.4. Benefits and Harms

RCTs evaluated cognitive behavioural therapy (CBT), mindfulness-based interventions (MBIs), and cognitive therapy (CT) compared with placebo, usual care or waiting-list controls. For CBT, one trial comparing CBT with placebo reported that CBT probably results in little to no difference in pain severity (Pain and Impairment Relationship Scale) and in function (ODI) at >4 months [78]. When compared with usual care, CBT may improve function (RMDQ) at ≤4 months [80,84,89] and >4 months [89]. CBT probably results in little to no difference in psychological distress (BDI) at ≤4 months [84] and in quality of life (SF-36 perceived general health) at ≤4 months and >4 months [85]. Effects on pain severity (VAS) at ≤4 months [79,80,81,82,83,84] and >4 months [81], and on function (Pain Disability Index), were very uncertain [82]. For MBIs, the evidence suggests probable improvements in pain severity (NRS) [90], in function (RMDQ) at ≤4 months and >4 months [86,89], and in quality of life (Health Status Inventory—mental and physical health) at >4 months [90]. Other outcomes, including quality of life (SF-36 global health composite) at ≤4 months [86] and depression (BDI) [90], probably result in little to no difference. Several outcomes, including pain severity (McGill Pain Questionnaire [MPQ]) and quality of life (SF-36—mental and physical health), were very uncertain [86,87]. For CT, the evidence was very uncertain across all reported quality of life outcomes (SF-36—physical function, general health, and mental health) at >4 months [88]. Further details are presented in Table 12.

#### 3.6.5. Certainty of Evidence

The overall certainty in the evidence of effects was judged to be very low based on the lowest certainty rating for critical outcomes, reflecting very serious risk of bias, serious inconsistency, and very serious imprecision.

#### 3.6.6. Contextual Factors

Evidence on resource use and cost-effectiveness came from cost data and economic evaluations, whereas values and preferences, equity, acceptability, and feasibility relied primarily on Task Force experience (Table 13). No evidence was identified on how patients value the outcomes of psychological interventions, and the Task Force judged that there may be important uncertainty and variability in patients’ values and preferences. Evidence on resource requirements was not found, although cost information for cognitive therapy was provided by the Centre of Health Technology Assessment, MoH (Supplementary Material S4). Personnel time constitutes the main cost, and delivery may be undertaken by psychologists or other trained health professionals such as nurses or physiotherapists. The Task Force noted insufficient infrastructure and shortages of trained personnel in rural areas, which would increase resource needs and result in a moderate cost impact. Economic evidence from the source guideline indicated that the cognitive behavioural approach had lower total costs and favourable cost-effectiveness [91], while an economic evaluation showed modest additional costs for cognitive behavioural therapy and potential cost savings for mindfulness-based stress reduction; both were accompanied by improvements in quality-adjusted life years [92]. No evidence was identified for equity, acceptability, or feasibility. The Task Force concluded that limited infrastructure may reduce equity, while evidence-based psychological interventions are probably acceptable to patients, clinicians, and decision-makers and probably feasible to implement, although challenges may arise in some settings.

### 3.7. Epidural Injections

#### 3.7.1. Question

Should epidural injections versus non-invasive treatments be used in patients with sciatica?

#### 3.7.2. Recommendation

In patients with sciatica, the Task Force suggests using epidural injections (*conditional recommendation*, *very low certainty of evidence*).

#### 3.7.3. Evidence Summary

The NICE guideline [37] identified three RCTs [93,94,95] and conducted a meta-analysis comparing the effects of image-guided or non-image-guided steroid plus epidural injections with non-invasive treatments in patients with sciatica primarily caused by ≥70% disc prolapse. The update search identified no additional studies for inclusion. The trials were conducted in small populations with at least moderately severe sciatica who had no further treatment options available to them apart from surgery.

#### 3.7.4. Benefits and Harms

RCTs evaluated image-guided and non-image-guided epidural steroid injections compared with non-invasive or pharmacological interventions. For image-guided steroid plus anaesthetic injection, one trial showed that it probably results in little to no difference in pain (VAS) at 2 weeks compared with combined non-invasive interventions [93]. For non-image-guided steroid plus anaesthetic injection compared with NSAIDs, the evidence suggests an improvement in pain (VAS) at >4 months and a probable improvement in disability (ODI) at ≤4 months, with little to no difference in healthcare utilisation (analgesic/paracetamol use) [94]. When compared with combined NSAIDs, opioids, and muscle relaxants, non-image-guided injection probably results in little to no difference in pain (VAS) at >4 months and in adverse events (flushing, headache or backache), while effects on pain (VAS) at ≤4 months were very uncertain [95]. All effect estimates are summarised in Table 14.

#### 3.7.5. Certainty of Evidence

The overall certainty in the evidence of effects was judged to be very low, based on the lowest certainty ratings for the critical outcomes, reflecting very serious risk of bias and serious imprecision.

#### 3.7.6. Contextual Factors

No direct evidence was identified for values, resource use and cost-effectiveness, equity, acceptability, or feasibility, and judgements therefore relied on Task Force experience (Table 15). The Task Force considered that patients’ values and preferences for epidural injections may vary, reflecting possible uncertainty. Cost information from the MoH indicated that resource requirements depend on medication costs, with moderate overall cost implications and no additional considerations noted (Supplementary Material S4). The lack of adequate infrastructure and limited trained personnel in rural areas was judged likely to reduce equity by restricting access. Epidural injections were considered probably acceptable to patients, clinicians and decision-makers, and probably feasible to implement within healthcare facilities that have sufficient specialist capacity.

### 3.8. Image-Concordant Pathology

#### 3.8.1. Question

Should image-concordant pathology or the presence of radicular symptoms versus no image-concordant pathology or the presence of radicular symptoms be used for predicting response to surgery in patients with suspected sciatica?

#### 3.8.2. Recommendation

In patients with suspected sciatica, the Task Force suggests using image-concordant pathology or the presence of radicular symptoms to predict response to surgery (*conditional recommendation*, *low certainty of evidence*). For patients with other surgical indications (e.g., objective weakness, positive MRI findings, or cauda equina symptoms) along with long-standing symptoms and failure of non-surgical management, the Task Force strongly recommends prompt surgical evaluation. In line with the source guideline [37], body mass index (BMI), smoking status, and psychological distress should not influence referral decisions for surgical opinion for sciatica.

#### 3.8.3. Evidence Summary

The NICE guideline [37] identified three RCTs [96,97,98] and conducted a meta-analysis to determine whether the presence of image-concordant pathology or radicular symptoms versus no image-concordant pathology or the presence of radicular symptoms predicts response to surgery in patients with suspected sciatica. The update search identified no additional studies for inclusion. Although all three included studies conducted multivariable analyses, they adjusted for different confounding variables [37].

#### 3.8.4. Benefits and Harms

Evidence from prognostic studies suggests that specific radicular symptoms are prognostic factors for improved surgical outcomes in patients with sciatica. One study reported that radicular symptoms were prognostic for better function, with improved ODI scores at 4 years following open decompressive laminectomy [96]. Another study found that radicular symptoms predicted reduced leg pain at 12 months [98]. In patients undergoing discectomy, having leg pain greater than back pain predicted greater improvements, including a 50% improvement in function (ODI) and in pain (VAS), both with low certainty [97]. None of the included studies reported adverse events. All effect estimates are summarised in Table 16.

#### 3.8.5. Certainty of Evidence

The overall certainty in the evidence of effects was judged to be low, based on the lowest certainty in the evidence for the critical outcomes due to the observational study designs.

#### 3.8.6. Contextual Factors

No direct evidence was identified for any contextual factor domains, so judgements relied on Task Force experience (Table 17). For values and preferences, the Task Force considered that there was probably no important variability in how patients value using image-concordant pathology or radicular symptoms to guide surgical decisions. Regarding resources and cost-effectiveness, cost information from the MoH indicated that requirements are mainly linked to imaging expenses, including MRI equipment and workforce availability, and establishing a diagnosis with MRI may reduce repeated consultations (Supplementary Material S4). Overall resource needs likely vary by setting, and cost-effectiveness probably favours the intervention. For equity, extended MRI waiting times may limit timely diagnosis, and the Task Force judged that reliance on imaging or radicular symptoms would probably reduce equity despite national coverage. The approach was considered probably acceptable to patients, clinicians, and decision-makers and feasible within existing healthcare teams.

### 3.9. Spinal Decompression

#### 3.9.1. Question

Should spinal decompression versus usual care or other interventions be used in patients with sciatica?

#### 3.9.2. Recommendation

In patients with sciatica, the Task Force suggests using spinal decompression (*conditional recommendation*, *very low certainty of evidence*). The Task Force noted that the choice of treatment depends on symptom profile and duration, and surgical management should be considered only when non-surgical treatments have been unsuccessful, radiological findings are consistent with symptoms, and established clinical pathways have been followed.

#### 3.9.3. Evidence Summary

The NICE guideline [37] identified seven RCTs [99,100,101,102,103,104,105] comparing spinal decompression with usual care or other interventions for sciatica. The update search found no additional studies. The guideline reported high cross-over from usual care to discectomy, with those crossing over showing high pain scores after usual care. Because results were analysed using intention-to-treat, the treatment effect in the usual care group may have been overestimated. This reflects the common challenge in surgical trials without placebo controls. If cross-over was not permitted, high withdrawal rates from usual care would be expected. The guideline also noted that excluding cross-over participants would likely increase the observed benefit of discectomy, though this would introduce bias. For other comparisons, evidence came from single small trials, resulting in substantial uncertainty.

#### 3.9.4. Benefits and Harms

Across five comparisons, evidence from RCTs evaluated discectomy, percutaneous decompression, plasma disc decompression and laminectomy against usual care, combination therapy, epidural steroid injection or other non-invasive treatments. For discectomy versus usual care, the evidence suggests probable improvements in leg pain (VAS) and back pain (VAS and Sciatica Bothersomeness Index [SBI]) at >4 months to 1 year [100,101,102], with probably little to no difference in function (ODI) or mortality. Effects on multiple SF-36 domains (bodily pain and physical functioning) at ≤4 months and at 2 years, as well as effects on healthcare utilisation and morbidity were very uncertain [100,101,102]. When compared with combination manual therapy, exercise, and self-management, discectomy probably results in little to no difference in pain (MPQ), function (RMDQ), or quality of life (SF-36 bodily pain) at ≤4 months, with very uncertain effects on SF-36 physical function [103]. Percutaneous decompression probably improves leg pain (Visual Numeric Scale [VNS]) from 4 months to 2 years [104]. Plasma disc decompression probably improves leg pain (VAS), back pain (VAS), and function (ODI) at 6 months, with little to no difference in procedure-related adverse events, and very uncertain effects on morbidity and mortality [105]. For laminectomy versus usual care, the evidence suggests little to no difference in pain (Low Back Pain Bothersomeness Index), function (ODI), or quality of life (SF-36 bodily pain and physical functioning) at 1 year, with very uncertain effects on SBI [99]. All effect estimates are summarised in Table 18.

#### 3.9.5. Certainty of Evidence

The overall certainty in the evidence of effects was judged to be very low, reflecting very serious risk of bias, serious inconsistency, and serious imprecision across outcomes.

#### 3.9.6. Contextual Factors

No direct evidence was identified for values, equity, acceptability, or feasibility, so judgements relied on Task Force experience (Table 19). For values and preferences, the Task Force concluded that there was probably no important variability in how patients value outcomes of spinal decompression. Evidence on resource use and cost-effectiveness came from three cost-utility analyses in the source guideline, which reported higher incremental costs [106,107,108], together with MoH cost estimates for laminectomy and discectomy (Supplementary Material S4). Overall, spinal decompression requires substantial resources, and cost-effectiveness generally does not favour the intervention, although it may vary by diagnosis. No evidence was identified for equity; because the procedure depends on specialised facilities and trained personnel, the Task Force judged that equity would probably be reduced despite national coverage. No evidence addressed acceptability or feasibility, but decompression was considered probably acceptable and probably feasible, with delivery constrained in regions with limited infrastructure.

### 3.10. Radiofrequency Denervation

#### 3.10.1. Question

Should radiofrequency denervation for facet joint pain versus placebo or usual care, or versus other treatments, be used in patients with non-specific LBP?

#### 3.10.2. Recommendation

In patients with non-specific LBP, the Task Force suggests using radiofrequency denervation (*conditional recommendation*, *very low certainty of evidence*). The Task Force agreed that this intervention should not be used in patients with non-specific LBP without evidence of facet joint arthropathy but be reserved for appropriately selected subgroups, such as those with suspected facet joint arthropathy (spondylosis). They further agreed that at least one diagnostic medial branch nerve block producing ≥50% improvement in pain or daily activities should be performed before proceeding to radiofrequency denervation.

#### 3.10.3. Evidence Summary

The NICE guideline [37] identified seven RCTs [109,110,111,112,113,114,115] and conducted a meta-analysis comparing radiofrequency denervation for facet joint pain with placebo/sham, usual care or other active treatments in adults with non-specific LBP. The update search identified no additional eligible studies. Overall, trials were small and subject to methodological limitations, contributing to very low certainty for several outcomes.

#### 3.10.4. Benefits and Harms

Across trials, radiofrequency denervation was compared mainly with placebo or sham procedures and, in one study, with medial branch block. Compared with placebo or sham, radiofrequency denervation probably improves pain intensity (VAS) in the short-term (≤4 months) [109,110,111,112] and at longer periods (>4 months) [109,110,113] and probably improves vitality-related quality of life (SF-36 vitality subscale) at ≤4 months [114]. It also probably increases the proportion of responders achieving at least 50% pain reduction or a favourable global perceived effect [112,114]. Radiofrequency denervation probably results in worse outcomes with respect to treatment-related pain of moderate or severe intensity [114], and results in little to no difference in several quality of life domains (SF-36), including general health, mental health, social functioning and physical functioning [114]. Effects on function (RMDQ) [111], additional quality of life outcomes (SF-36 pain subscale) [114], healthcare utilisation [112,113], responder rates at >4 months [112], and neurological adverse events including sensory changes and motor loss) [114] were very uncertain. When compared with medial branch block, evidence for radiofrequency denervation was very uncertain across pain (VNS) and quality of life outcomes (EQ-5D) at both ≤4 months and >4 months [115]. All effect estimates are summarised in Table 20.

#### 3.10.5. Certainty of Evidence

The overall certainty in the evidence of effects was judged to be very low, based on the lowest certainty for critical outcomes. This reflects serious risk of bias and very serious imprecision across trials, including small sample sizes and wide confidence intervals.

#### 3.10.6. Contextual Factors

No direct evidence was identified for values, equity, acceptability or feasibility, so judgements relied on Task Force experience (Table 21). For values and preferences, the Task Force concluded that there is probably no important variability. For resource use and cost-effectiveness, evidence came from a within-trial economic evaluation [114] and the economic model developed in the NICE guideline [37], both indicating that radiofrequency denervation involves notable procedure-related costs and that cost-effectiveness depends on sufficient duration of pain relief, with favourable results only when relief persists beyond approximately 16 months; however, these analyses had potentially serious limitations. A Dutch societal-perspective study [116] suggested that radiofrequency denervation combined with structured exercise may not be cost-effective. No further evidence on resource requirements was identified. Overall, the Task Force judged that the intervention entails moderate costs and that cost-effectiveness probably favours its use. Given national health coverage, equity impact is likely minimal, and radiofrequency denervation is probably acceptable and feasible where expertise and equipment are available.

### 3.11. Pain Neuroscience Education

#### 3.11.1. Question

Should pain neuroscience education versus no pain neuroscience education be used to reduce disability, pain and recurrence in patients with chronic non-specific LBP?

#### 3.11.2. Recommendation

In patients with chronic non-specific LBP, the Task Force suggests using pain neuroscience education (*conditional recommendation*, *low certainty of evidence*). The Task Force suggests prioritising pain neuroscience education for patients at increased risk of disability and chronicity.

#### 3.11.3. Evidence Summary

The source systematic review [38] identified eight RCTs [117,118,119,120,121,122,123,124] and conducted a meta-analysis comparing pain neuroscience education with no pain neuroscience education in patients with chronic non-specific LBP. The update search identified no additional studies for inclusion.

#### 3.11.4. Benefits and Harms

The trials evaluated pain neuroscience education delivered alone and pain neuroscience education combined with physiotherapeutic treatment compared with no pain neuroscience education. For pain neuroscience education alone, the evidence suggests that it improves disability (RMDQ) at 32.8 days [117,118,119,120,121]. Pain neuroscience education may result in little to no difference in short-term pain (NRS) at 31.8 days [117,118,120,121,122,123], long-term pain (NRS) at 12 months [118,120], short-term psychological outcomes (Tampa Scale for Kinesiophobia) at 3.33 weeks [121,122,124], and long-term disability (RMDQ) at 12 months [118,120]. For pain neuroscience education combined with physiotherapeutic treatment, the evidence suggests improvements in short-term disability (RMDQ) at 40 days [117,120,121] and in short-term pain (NRS) at 32.6 days [117,120,121,122,123]. None of the included studies reported adverse events. All effect estimates for each comparison are presented in Table 22.

#### 3.11.5. Certainty of Evidence

The overall certainty in the evidence of effects was judged to be low, based on the lowest certainty for the critical outcomes. This was due to inconsistency, indirectness, and imprecision in the evidence, all of which were rated serious.

#### 3.11.6. Contextual Factors

Evidence was identified only for acceptability, with no direct evidence found for values and preferences, resource requirements, cost-effectiveness, equity, or feasibility (Table 23). For values and preferences, the Task Force judged, based on experience, that there is probably no important variability in how patients value the outcomes of pain neuroscience education. For resource use, the Task Force considered training costs, required materials, patient travel, potential out-of-pocket expenses, the need for specialised care, and the availability of additional appointments, noting that telehealth delivery may reduce these demands. Cost-effectiveness evidence was not available, but the Task Force agreed that a formal analysis would likely show no meaningful difference between the intervention and the comparison. For equity, access in rural areas may be limited without telehealth, which could reduce equity. Acceptability was supported by the GLITtER feasibility trial [125], although the Task Force judged pain neuroscience education probably not acceptable in Saudi Arabia because it may increase clinician workload. For feasibility, the Task Force concluded that the intervention is probably feasible, although dependent on training, staff availability, and infrastructure.

## 4. Discussion

This national clinical practice guideline provides a comprehensive, evidence-based framework for the assessment and management of non-specific LBP and sciatica in adults in Saudi Arabia. Developed using the GRADE ADOLOPMENT methodology, the guideline offers a rigorously adapted set of recommendations rooted in high-quality international evidence while explicitly incorporating clinical, cultural, and health-system considerations relevant to the Kingdom. LBP remains a leading cause of disability and healthcare utilisation across Saudi Arabia, affecting individuals in diverse demographic and occupational groups. The recommendations presented in this document aim to reduce unwarranted variation in practice, promote high-value care, and support consistent, high-quality clinical decision-making across primary, secondary, and tertiary care settings.

### 4.1. Key Messages

Across the eleven clinical questions examined, several overarching conclusions inform optimal LBP management in the Saudi context.

- First, validated risk assessment and stratification tools exhibited sufficient prognostic accuracy to support their integration into routine clinical assessment. These tools facilitate timely identification of patients at increased risk of persistent symptoms, enabling more rational referral pathways and potentially reducing unnecessary imaging.
- Second, routine early imaging in the absence of red flags did not yield meaningful improvements in pain, function, or psychological outcomes and was consistently associated with increased healthcare utilisation. Imaging decisions should therefore be guided primarily by clinical evaluation, with investigations reserved for situations in which results are expected to influence management.
- Third, pharmacological treatments commonly used for sciatica demonstrated limited clinical benefit and an unfavourable risk profile, reinforcing the need for selective and judicious prescribing, particularly regarding benzodiazepines, gabapentinoids, corticosteroids, and long-term opioids. Short-term opioid therapy may be considered only for carefully selected patients with acute sciatica.
- Fourth, multidisciplinary interventions incorporating workplace, ergonomic or rehabilitation components showed improvements in return to work outcomes and may mitigate indirect costs associated with prolonged work absenteeism, findings that hold particular relevance within a rapidly developing national labour market.
- Fifth, psychological interventions, including cognitive behavioural therapy and mindfulness-based interventions, produced small but meaningful improvements in function and quality of life when implemented as part of a broader biopsychosocial care approach. Their contribution remains important despite heterogeneity and methodological limitations in the underlying evidence.
- Sixth, procedural interventions such as epidural injections, spinal decompression, and radiofrequency denervation were supported by mixed or very low certainty evidence. These procedures may benefit carefully selected patients with severe or persistent symptoms and image-concordant pathology but should be reserved for clearly defined indications requiring specialist expertise.

Collectively, the evidence supports a measured, evidence-informed, and context-sensitive approach to LBP that prioritises effective, acceptable, and feasible interventions while promoting high-value care across the Saudi healthcare system.

### 4.2. Strengths

This guideline benefits from several strengths that enhance its methodological rigour, clinical relevance, and national applicability. The use of the GRADE ADOLOPMENT approach ensured transparent, systematic, and structured adaptation of international evidence to the Saudi context. Each recommendation reflects a careful synthesis of available research, assessment of evidence certainty, and explicit consideration of benefits, harms, patient values, feasibility, acceptability, and resource implications. The multidisciplinary composition of the Task Force, which included experts from a wide range of clinical specialties, academic institutions, and geographic regions, enabled comprehensive deliberation that incorporated diverse clinical perspectives and region-specific considerations. This representation ensured that the recommendations align with prevailing practice patterns, resource availability, and system-level characteristics across the Kingdom. Furthermore, the guideline places notable emphasis on implementation feasibility. Recommendations were formulated with explicit attention to existing variation in imaging access, distribution of specialist services, referral structures, and patterns of healthcare utilisation. This contextualisation enhances the practical utility of the guideline and supports consistent, equitable, and high-quality practice across all levels of care.

### 4.3. Limitations

Despite its strengths, several limitations should be acknowledged. The certainty of evidence supporting many recommendations was low or very low, reflecting the methodological shortcomings of primary studies, including small sample sizes, inconsistency, imprecision, and indirectness. Consequently, several recommendations required careful judgement by the Task Force to balance potential benefits and harms in accordance with GRADE principles. Much of the evidence originated from studies conducted outside Saudi Arabia. Although the ADOLOPMENT methodology explicitly incorporates contextualisation, certain findings may not fully reflect local clinical characteristics, cultural expectations, or patterns of healthcare delivery. This limitation is particularly relevant for interventions influenced by psychosocial factors or requiring specialised multidisciplinary services. Additionally, the guideline does not address the evaluation or management of serious spinal pathology, inflammatory causes of LBP, progressive neurological deficits, or cauda equina syndrome, as these fall outside the defined scope. Finally, the guideline reflects evidence available up to the final literature search date and will require periodic updates as new research emerges.

### 4.4. Research Needs

The guideline development process identified several priority areas for future research that are essential to strengthen the evidence base and inform subsequent updates. The NICE guideline [37] highlighted the need for robust evaluations of the clinical and cost-effectiveness of opioids for acute sciatica, antidepressants for sciatica, benzodiazepines for acute LBP, and codeine, alone or in combination with paracetamol, for acute LBP. These pharmacological treatments remain widely used despite limited evidence, underscoring the need for trials capable of clarifying their role in contemporary practice. Further research is also required to evaluate procedural interventions. High-quality comparative trials of image-guided versus non-image-guided epidural injections are needed to determine their relative clinical utility. For radiofrequency denervation, long-term studies examining effectiveness, safety, and cost-effectiveness are essential to determine its appropriate place in management. The systematic review [38] informing the pain neuroscience education question emphasised the need for trials that assess long-term effects on pain, disability and cost-effectiveness when integrated into standard care.

Beyond these topic-specific priorities, broader research relevant to the Saudi context is needed. This includes the validation of risk assessment and stratification tools in local populations, evaluations of stratified care pathways across different levels of service delivery, and studies assessing the feasibility and acceptability of psychological and multidisciplinary interventions within the national healthcare infrastructure. Longitudinal studies examining prognostic factors, recurrence patterns, and functional outcomes among individuals with LBP in Saudi Arabia would strengthen the understanding of disease trajectories and support more targeted prevention and rehabilitation strategies. Implementation research examining barriers and facilitators to guideline adoption is also essential to ensure consistent and effective integration of evidence-based care across diverse clinical settings.

## 5. Conclusions

This national clinical practice guideline offers a comprehensive and rigorously developed framework for the assessment and management of non-specific LBP and sciatica in adults in Saudi Arabia. By systematically adapting high-quality international evidence to the clinical and healthcare context of the Kingdom, the guideline aims to support consistent, effective, and high-value practice across all levels of care. The recommendations emphasise accurate clinical assessment, appropriate use of imaging, careful selection of pharmacological options, and the integration of psychological, multidisciplinary, and procedural interventions where supported by evidence. Although the certainty of evidence varies across topics, the guideline provides clear and practical direction for improving the quality of care and reducing unwarranted variation. Ongoing research within Saudi populations will be essential to refine future guidance and ensure sustained improvements in patient outcomes.

## Figures and Tables

**Table 1:** Benefits of validated risk assessment tools.

Instrument	Outcome	Time Frame	Effect Estimate	Conclusion	GRADE Certainty
SBST	To predict pain ^a^ [42,43,46,47,48,51]	12 mos	AUC 0.71 (95% CI 0.54 to 0.88)	May be a reasonably useful tool	Very low
		6 mos	AUC 0.73 (95% CI 0.72 to 0.73)		
		6 mos	AUC 0.66 (95% CI 0.46 to 0.85)		
		3 mos	AUC 0.79 (95% CI 0.68 to 0.89)		
		3 mos	AUC 0.68 (95% CI 0.55 to 0.81)		
	To predict functional improvement ^b^ [42,47,48,49,51]	12 mos	AUC 0.82 (range 0.61 to 0.10)	May be a reasonably useful tool	Very low
		6 mos	AUC 0.77 (range 0.69 to 0.84)		
		6 mos	AUC 0.82 (range 0.73 to 0.90); Sensitivity 80.1%; Specificity 65.4%		
		3 mos	AUC 0.71 (range 0.66 to 0.77)		
		3 mos	AUC 0.81 (range 0.78 to 0.84)		
CPRIS	To predict pain [43]	4 mos	AUC 0.79 (95% CI 0.75 to 0.83); Sensitivity 72%; Specificity 70%	Very uncertain effect	Very low
LBPPS	To predict recovery from pain [45]	12 mos	Recovery cut-off ≥2: AUC 0.59 (95% CI 0.52 to 0.66); Sensitivity 80%; Specificity 27%. Recovery cut-off ≥4: AUC 0.59 (95% CI 0.52 to 0.66); Sensitivity 30%; Specificity 81%. Calibration: intercept 0.02 (95% CI 0.02 to 0.03).	Very uncertain effect	Very low
ÖMPQ	To predict problem severity [44]	6 mos	AUC 0.88 (95% CI 0.78 to 0.99); Sensitivity 88%; Specificity 85.7%	Very uncertain effect	Very low
ODI	To predict functional improvement [50]	1 mo	AUC 0.93 (95% CI 0.88 to 0.98)	Very uncertain effect	Very low

Abbreviations: SBST, STarT Back Screening Tool; CPRIS, Chronic Pain Risk Item Set; LBPPS, Low Back Pain Perception Scale; ÖMPQ, Modified Örebro Musculoskeletal Pain Questionnaire; ODI, Oswestry Disability Index; mos, months; mo, month; AUC, area under the curve; CI, confidence interval. ^a^ Assessed using a Numeric Rating Scale, and the Patient’s Global Impression of Change scale. ^b^ Assessed using a variety of methods including self-reporting, Oswestry Disability Index, Roland–Morris Disability Questionnaire, and the Global Rating of Change.

**Table 2:** Contextual factors for risk assessment tools.

GRADE EtD Domain	Evidence Identified	Task Force Judgement	Implications for Saudi Arabia
Values and preferences	No	Patients tend to have greater confidence in imaging than questionnaire-based assessments, with important uncertainty and variability in preferences.	Variability in preferences is likely.
Resource use and cost-effectiveness	No	Costs are negligible and tools can be administered by clinicians; stratification may reduce unnecessary resource use and cost-effectiveness probably favours the intervention.	Minimal cost with potential savings; likely acceptable.
Equity	No	Tools can be implemented across settings at minimal expense and may support more efficient allocation of resources.	Probably increases equity given universal coverage.
Acceptability	No	Tools are straightforward and user-friendly and are probably acceptable to patients, clinicians, and decision-makers, although some patients may prefer imaging.	Probably acceptable overall.
Feasibility	No	Tools require only training and administration time and can be applied across clinical scenarios.	Feasible across healthcare settings.

Abbreviations: EtD, Evidence-to-Decision.

**Table 3:** Benefits of stratified management using SBST.

Instrument	Outcome	Time Frame	Effect Estimate	Conclusion	GRADE Certainty
SF-12 [52]	Quality of life (physical)	>4 mos	MD 2.3 (95% CI 0.73 to 3.87)	Increases quality of life	High
	Quality of life (mental)	>4 mos	MD 0.5 (95% CI −1.39 to 2.39)	Probably little to no difference	Moderate
VAS [52]	Pain severity	>4 mos	MD −0.2 (95% CI −0.58 to 0.18)	Probably little to no difference	Moderate
HADS [52]	Anxiety	>4 mos	MD −0.3 (95% CI −0.9 to 0.3)	Probably little to no difference	Moderate
	Depression	>4 mos	MD −0.5 (95% CI −1.08 to 0.08)	Probably little to no difference	Moderate
RMDQ [52,54]	Function	>4 mos	MD −0.65 (95% CI −1.34 to 0.04)	Probably little to no difference	High
Various [53]	Healthcare utilisation ^a^	Not reported	RR 0.95 (95% CI 0.51 to 1.78)	Very uncertain effect	Very low

Abbreviations: STarT Back Screening Tool, SBST; SF-12, 12-Item Short Form Survey; VAS, Visual Analogue Scale; HADS, Hospital Anxiety and Depression Scale; RMDQ, Roland–Morris Disability Questionnaire; mos, months; MD, mean difference; CI, confidence interval; RR, risk ratio. ^a^ Included prescribing, investigations, hospitalisation, or health professional visit.

**Table 4:** Contextual factors for stratifying management.

GRADE EtD Domain	Evidence Identified	Task Force Judgement	Implications for Saudi Arabia
Values and preferences	No	Patients tend to have greater confidence in imaging than questionnaire-based assessments, with important uncertainty and variability in values and preferences.	Variability in preferences is expected.
Resource use and cost-effectiveness	Yes	Two cost-utility analyses [55,56] showed stratification using SBST reduced costs and increased QALYs. The Task Force judged the tools cost-free to deliver, producing moderate savings and likely favourable cost-effectiveness.	Probably cost-effective with minimal cost.
Equity	No	Tools can be implemented across settings with minimal expense and may support more efficient allocation of resources.	Likely increases equity given universal coverage.
Acceptability	No	Tools are straightforward and user-friendly and are probably acceptable to patients, clinicians, and decision-makers, although some patients may prefer imaging.	Probably acceptable overall.
Feasibility	No	Require only training and administration time and can be applied across clinical scenarios.	Feasible across healthcare settings.

Abbreviations: EtD, Evidence-to-Decision; STarT Back Screening Tool, SBST; QALYs, quality-adjusted life years.

**Table 5:** Benefits of imaging.

Instrument	Outcome	Time Frame	Effect Estimate	Conclusion	GRADE Certainty
SF-36	Quality of life (bodily pain) [57,58]	>4 mos–1 y	MD 3.97 (95% CI 0.36 to 7.59)	Improves bodily pain	High
	Quality of life (mental health) [57,58]	>4 mos–1 y	MD 2.77 (95% CI 0.03 to 5.51)	Improves mental health	Moderate
ALBPS	Pain severity [57]	>4 mos–1 y	MD −4.2 (95% CI −7.17 to −1.23)	Reduces pain	High
Various (healthcare utilisation)	Imaging at least once [57]	>4 mos–1 y	RR 3.04 (95% CI 2.60 to 3.55)	Increases imaging	High
	Outpatient consultation [57,59]	>4 mos–1 y	RR 1.24 (95% CI 1.14 to 1.35)	Increases outpatient visits	Moderate
RMDQ	Function [58]	>4 mos–1 y	MD 0.2 (95% CI −1.88 to 2.28)	Probably little to no difference	Low
SF-36	Quality of life (physical functioning) [57,58]	>4 mos–1 y	MD 3.25 (95% CI −0.6 to 7.11)	Probably little to no difference	Moderate
HADS	Psychological distress (anxiety) [58]	>4 mos–1 y	MD −0.4 (95% CI −2.08 to 1.28)	Probably little to no difference	Low
	Psychological distress (depression) [58]	>4 mos–1 y	MD −0.3 (95% CI −1.68 to 1.08)	Probably little to no difference	Low
Various (healthcare utilisation)	Hospital admission [57,59]	>4 mos–1 y	RR 1.25 (95% CI 0.77 to 2.05)	Probably little to no difference	Very low
	Prescribed drugs [59]	>4 mos–1 y	RR 1.17 (95% CI 0.84 to 1.62)	Probably little to no difference	Very low
	Referral to physiotherapy/other professionals [58]	>4 mos–1 y	RR 0.97 (95% CI 0.67 to 1.39)	Probably little to no difference	Low
	Primary care consultation [57]	>4 mos–1 y	RR 1.01 (95% CI 0.92 to 1.11)	Probably little to no difference	Moderate

Abbreviations: SF-36, 36-Item Short Form Survey; ALBPS, Aberdeen Low Back Pain Score; RMDQ, Roland–Morris Disability Questionnaire; HADS, Hospital Anxiety and Depression Scale; mos, months; y, year; MD, mean difference; CI, confidence interval; RR, risk ratio.

**Table 6:** Contextual factors for imaging.

GRADE EtD Domain	Evidence Identified	Task Force Judgement	Implications for Saudi Arabia
Values and preferences	No	Probably no important variability in how patients value outcomes.	Values expected to be similar across patients.
Resource use and cost-effectiveness	Yes	One study [60] showed imaging increases costs, utilisation and possibly absenteeism; MoH data showed MRI is high cost and X-ray moderate cost; one cost-utility analysis [57] found early imaging increased costs but improved QALYs. Overall, imaging leads to moderate costs and may be cost-effective depending on setting.	MRI high cost; X-ray accessible; selective imaging likely more cost-effective and preferable.
Equity	No	MRI less available in rural and vulnerable regions; X-ray widely accessible.	Likely increases equity due to comprehensive national coverage.
Acceptability	No	Imaging generally acceptable to patients, clinicians and decision-makers.	Probably acceptable overall.
Feasibility	No	X-ray feasible; MRI less feasible due to specialised equipment and staffing.	Feasible depending on modality.

Abbreviations: EtD, Evidence-to-Decision; MoH, Ministry of Health; MRI, magnetic resonance imaging; X-ray, plain radiograph; QALYs, quality-adjusted life years.

**Table 7:** Summary of recommendations for pharmacological treatments in sciatica.

Drug Class	Recommendation	Rationale
NSAIDs	No recommendation for or against use	Risk of harm and lack of clear evidence of benefit; evidence insufficient to support or oppose their use.
Benzodiazepines	Recommended against	Lack of evidence for benefit, evidence of worse pain outcomes, and risk of misuse.
Gabapentinoids and other antiepileptics	Recommended against	Limited evidence of benefit and increased risk of harm; no reason to expect other antiepileptics to be more effective or safer.
Oral corticosteroids	Recommended against	Small apparent improvements in quality of life from one study were not convincing; outweighed by evidence of harm; no benefit on pain severity or function.
Opioids	Short-term use may be beneficial for acute sciatica; recommended against for chronic sciatica	Consensus judgement: potential benefit for acute pain relief; lack of benefit for long-term use and increased risk of harm with prolonged use.
Antidepressants	No recommendation for or against use	Lack of direct evidence; commonly used; clinical experience suggests lower risk of harm compared with some other medicines (e.g., long-term opioids).
Paracetamol, nefopam, and non-benzodiazepine muscle relaxants	No recommendation for or against use	No direct evidence identified; not widely prescribed for management of sciatica alone.

Abbreviations: NSAIDs, non-steroidal anti-inflammatory drugs.

**Table 8:** Benefits and potential harms of pharmacological treatments in sciatica.

Treatment	Outcome (Instrument)	Time Frame	Effect Estimate	Conclusion	GRADE Certainty
NSAIDs vs. placebo	Pain severity (VAS) [63]	≤4 mos	MD −4.5 (95% CI −9.28 to 0.28)	Probably little to no difference	High
	Adverse events (morbidity) [63,67]	≤4 mos	RR 1.41 (95% CI 0.94 to 2.11)	Probably little to no difference ^a^	Moderate
Benzodiazepines vs. placebo	Responder (VAS, ≥50% pain reduction) [62]	≤4 mos	RR 0.52 (95% CI 0.33 to 0.84)	Worse outcomes	Moderate
Gabapentinoids vs. placebo	Pain at rest [65]	≤4 mos	MD −0.8 (95% CI −1.15 to −0.45)	Reduces pain	Moderate
	Pain severity (NRS) [61,64]	≤4 mos	MD −0.16 (95% CI −0.53 to 0.21)	Probably little to no difference	Moderate
	Pain severity (NRS) [64]	>4 mos	MD 0.4 (95% CI −0.45 to 1.25)	Probably little to no difference	Low
	Function (RMDQ) [64]	≤4 mos	MD −0.1 (95% CI −2.21 to 2.01)	Probably little to no difference	Moderate
		>4 mos	MD 0.8 (95% CI −1.48 to 3.08)	Probably little to no difference	Moderate
	Adverse events (morbidity) [61]	>4 mos	RR 1.54 (95% CI 1.17 to 2.02)	Worse outcomes	Moderate
	Adverse events (morbidity) [61,65,68]	≤4 mos	RR 1.20 (95% CI 0.93 to 1.55)	Very uncertain ^b^	Very low
Corticosteroids vs. placebo	Function (ODI) [66]	≤4 mos	MD −5.7 (95% CI −9.97 to −1.43)	Probably little to no difference	High
		>4 mos	MD −7.4 (95% CI −12.68 to −2.12)	Probably little to no difference	High
	Pain severity (NRS) [66]	≤4 mos	MD −0.2 (95% CI −0.85 to 0.45)	Probably little to no difference	Moderate
		>4 mos	MD −0.6 (95% CI −1.35 to 0.15)	Probably little to no difference	Moderate
	Adverse events (morbidity) [66]	≤4 mos	RR 2.06 (95% CI 1.38 to 3.08)	Worse outcomes ^c^	High

Abbreviations: NSAIDs, non-steroidal anti-inflammatory drugs; VAS, Visual Analogue Scale; NRS, Numeric Rating Scale; RMDQ, Roland–Morris Disability Questionnaire; ODI, Oswestry Disability Index; mos, months; MD, mean difference; CI, confidence interval; RR, risk ratio. ^a^ Included mainly dyspepsia, nausea, abdominal pain, and diarrhoea. ^b^ Included dizziness, somnolence, fatigue, decreased salivation, concentration difficulties, and increased sleep. ^c^ Included insomnia, sweating, increased appetite, and nervousness.

**Table 9:** Contextual factors for pharmacological treatments in sciatica.

GRADE EtD Domain	Evidence Identified	Task Force Judgement	Implications for Saudi Arabia
Values and preferences	No	Probably no important variability in how patients value outcomes.	Values likely similar across patients.
Resource use and cost-effectiveness	Yes	MoH cost per package data showed modest medication costs. One indirect cost-utility analysis [69] found electroacupuncture more cost-effective than NSAIDs in chronic LBP. Overall, pharmacological treatment involves negligible costs and cost-effectiveness probably favours its use.	Pharmacological treatment likely associated with negligible costs; cost-effectiveness acceptable when used appropriately.
Equity	No	Pharmacological treatment unlikely to affect equity under comprehensive national coverage.	Comprehensive coverage suggests minimal equity effect.
Acceptability	Yes	Evidence on muscle relaxants [70] showed small or uncertain benefit and increased adverse events; evidence on antidepressants [71] showed reduced pain but higher discontinuation. Despite this, pharmacological treatments remain widely used and are probably acceptable to patients, clinicians and decision-makers.	Probably acceptable to key stakeholders.
Feasibility	No	Pharmacological treatments are widely available and straightforward to prescribe, making implementation feasible across healthcare settings.	Feasible across healthcare settings.

Abbreviations: EtD, Evidence-to-Decision; MoH, Ministry of Health; NSAIDs, non-steroidal anti-inflammatory drugs; LBP, low back pain.

**Table 10:** Benefits of interventions with a return to work focus.

Instrument	Outcome	Time Frame	Effect Estimate	Conclusion	GRADE Certainty
Not specified	Days to return to work [72]	≤4 mos	MD −29.98 (95% CI −53.6 to −6.36)	Reduces days to return to work	Low
	Return to work [72]	≤4 mos	HR 1.7 (95% CI 1.2 to 2.3)	Improves return to work rates	Low
RMDQ	Function [73]	>4 mos	MD 2.73 (95% CI 2.47 to 2.99)	Improves function	Low
Various (healthcare utilisation)	Physiotherapist [72]	≤4 mos	RR 0.56 (95% CI 0.39 to 0.82)	Reduces physiotherapy use	—
	Manual therapist [72]	≤4 mos	RR 0.31 (95% CI 0.13 to 0.72)	Reduces manual therapy use	—
	Medical specialist [72]	≤4 mos	RR 0.46 (95% CI 0.26 to 0.81)	Reduces specialist visits	—
	Diagnostic tests [72]	≤4 mos	RR 0.49 (95% CI 0.33 to 0.73)	Reduces diagnostic testing	—
	Drugs for back pain [72]	≤4 mos	RR 0.70 (95% CI 0.49 to 0.99)	Reduces drug use	—
Not specified	Return to work [74]	>4 mos	RR −1.39 (95% CI 0.96 to 2.02)	May improve return to work	Moderate
EQ-5D	Quality of life [72]	≤4 mos	MD −0.05 (95% CI −0.13 to 0.03)	Probably little to no difference	High
RMDQ	Function [72]	≤4 mos	MD 0.91 (95% CI −0.8 to 2.62)	Probably little to no difference	Moderate
NRS	Pain [72]	≤4 mos	MD 0.21 (95% CI −0.55 to 0.97)	Probably little to no difference	Moderate
BDI	Psychological distress [74]	>4 mos	MD −1.3 (95% CI −4.71 to 2.11)	Probably little to no difference	Moderate
NRS	Pain [73]	>4 mos	MD −0.21 (95% CI −0.34 to −0.08)	Probably little to no difference	Moderate
	Pain [74]	>4 mos	MD −1.16 (95% CI −2.12 to −0.20)	Probably little to no difference	Low
Various (healthcare utilisation)	Occupational physician [72]	≤4 mos	RR 0.64 (95% CI 0.32 to 1.31)	Probably little to no difference	—
	General practitioner [72]	≤4 mos	RR 0.70 (95% CI 0.43 to 2.06)	Probably little to no difference	—

Abbreviations: RMDQ, Roland–Morris Disability Questionnaire; EQ-5D, EuroQol 5-Dimension; NRS, Numeric Rating Scale; BDI, Beck Depression Inventory; mos, months; MD, mean difference; CI, confidence interval; HR, hazard ratio; RR, risk ratio.

**Table 11:** Contextual factors for interventions with a return to work focus.

GRADE EtD Domain	Evidence Identified	Task Force Judgement	Implications for Saudi Arabia
Values and preferences	Yes	Systematic review [75] showed that multidisciplinary rehabilitation aligns with patient priorities. There is probably no important variability in how outcomes are valued.	Values likely similar across patients.
Resource use and cost-effectiveness	Yes	Within-trial economic evaluations [76,77] found that multidisciplinary return to work programmes had slightly higher direct costs but substantially lower total and indirect costs. The systematic review [75] supported clinical utility but noted uncertainty regarding cost-effectiveness. Overall, cost-effectiveness is likely favourable.	Programmes may reduce indirect costs and improve efficiency if implemented regionally.
Equity	No	Access may vary across regions. Equity gains depend on whether programmes are implemented beyond major centres.	Equity likely improved with comprehensive national coverage if programmes are accessible.
Acceptability	Yes	A pilot study [60] found these programmes acceptable to stakeholders and feasible to deliver. Time investment was the primary barrier.	Probably acceptable to patients, clinicians and decision-makers.
Feasibility	No	Implementation is feasible, although challenges may arise due to coordination demands and variation in regional resources.	Likely feasible across most settings.

Abbreviations: EtD, Evidence-to-Decision.

**Table 12:** Benefits of psychological interventions.

Treatment	Outcome (Instrument)	Time Frame	Effect Estimate	Conclusion	GRADE Certainty
CBT vs. placebo/sham	Pain severity (PAIRS) [78]	>4 mos	MD 0.9 (95% CI –3.61 to 5.41)	Probably little to no difference	Low
	Function (ODI) [78]	>4 mos	MD 0.7 (95% CI –4.81 to 6.21)	Probably little to no difference	Low
	Function (RMDQ) [80,84,89]	≤4 mos	MD –2.11 (95% CI –2.94 to –1.28)	May improve function	Low
CBT vs. usual care/waiting list	Function (RMDQ) [89]	>4 mos	MD –1.42 (95% CI –2.66 to –0.18)	May improve function	Moderate
	Psychological distress (BDI) [84]	≤4 mos	MD –1.65 (95% CI –3.42 to 0.12)	Probably little to no difference	Low
	Quality of life (SF-36—perceived general health) [85]	≤4 mos	MD 0 (95% CI –0.18 to 0.18)	Probably little to no difference	Moderate
	Quality of life (SF-36—perceived general health) [85]	>4 mos	MD 0 (95% CI –0.19 to 0.19)	Probably little to no difference	Moderate
	Pain severity (VAS) [79,80,81,82,83,84]	≤4 mos	MD –0.66 (95% CI –1.01 to –0.31)	Very uncertain effect	Very low
	Pain severity (VAS) [81]	>4 mos	MD –0.02 (95% CI –0.99 to 0.95)	Very uncertain effect	Very low
	Function (PDI) [82]	—	MD –1.2 (95% CI –6.44 to 4.04)	Very uncertain effect	Very low
Mindfulness vs. usual care/waiting list	Pain severity (NRS) [90]	—	MD –30.4 (95% CI –40.08 to –20.72)	Probably improves pain	Moderate
	Function (RMDQ) [86,89]	≤4 mos	MD –1.53 (95% CI –2.59 to –0.48)	Probably improves function	Moderate
	Function (RMDQ) [89]	>4 mos	MD –1.87 (95% CI –3.12 to –0.62)	Probably improves function	Moderate
	Quality of life (HSI—mental health) [90]	>4 mos	MD 4.5 (95% CI 0.45 to 8.55)	Probably improves mental health	Low
	Quality of life (HSI—physical health) [90]	>4 mos	MD 13 (95% CI 9.78 to 16.22)	Probably improves physical health	Moderate
	Quality of life (SF-36—global health composite) [86]	≤4 mos	MD 1.8 (95% CI –4.56 to 8.16)	Probably little to no difference	Low
	Depression (BDI-II) [90]	—	MD 1.61 (95% CI –2.99 to 6.21)	Probably little to no difference	Low
	Pain severity (MPQ) [86,87]	≤4 mos	MD –5.55 (95% CI –11.17 to 0.08)	Very uncertain effect	Very low
	Quality of life (SF-36—mental composite) [86,87]	≤4 mos	MD 4.74 (95% CI 2.87 to 6.62)	Very uncertain effect	Very low
	Quality of life (SF-36—physical composite) [86,87]	≤4 mos	MD 3.69 (95% CI 2.59 to 4.8)	Very uncertain effect	Very low
Cognitive therapy vs. usual care/waiting list	Quality of life (SF-36—physical function) [88]	>4 mos	MD 6.7 (95% CI –2.01 to 15.41)	Very uncertain effect	Very low
	Quality of life (SF-36—general health) [88]	>4 mos	MD 5 (95% CI –1.12 to 11.12)	Very uncertain effect	Very low
	Quality of life (SF-36—mental health) [88]	>4 mos	MD 6.8 (95% CI –0.7 to 14.3)	Very uncertain effect	Very low

Abbreviations: CBT, cognitive behavioural therapy; PAIRS, Pain and Impairment Relationship Scale; ODI, Oswestry Disability Index; RMDQ, Roland–Morris Disability Questionnaire; BDI, Beck Depression Inventory; SF-36, 36-Item Short Form Survey; VAS, Visual Analogue Scale; PDI, Pain Disability Index; NRS, Numeric Rating Scale; HSI, Health Status Inventory; MPQ, McGill Pain Questionnaire; mos, months; MD, mean difference; CI, confidence interval.

**Table 13:** Contextual factors for psychological interventions.

GRADE EtD Domain	Evidence Identified	Task Force Judgement	Implications for Saudi Arabia
Values and preferences	No	Possible important uncertainty and variability in how patients value outcomes.	Variability in values should be expected.
Resource use and cost-effectiveness	Yes	Cost information showed that personnel time is the main resource, with rural workforce shortages increasing resource needs and resulting in a moderate cost impact. One cost-utility analysis reported lower total costs and favourable cost-effectiveness for cognitive behavioural therapy [91], while another found modest additional costs for CBT and potential cost savings for mindfulness-based stress reduction [92]. Overall, cost-effectiveness probably favours psychological interventions.	Psychological interventions likely cost-effective when adequate workforce and infrastructure are available.
Equity	No	Unequal access likely; gaps in workforce and infrastructure would probably reduce equity.	Regional disparities may reduce equity despite national health coverage.
Acceptability	No	Psychological interventions are probably acceptable to patients, clinicians and decision-makers.	Likely acceptable if services are accessible and adequately staffed.
Feasibility	No	Probably feasible to implement, although feasibility may be constrained by workforce shortages and infrastructure limitations.	Feasible within existing healthcare facilities but dependent on training capacity and local resources.

Abbreviations: EtD, Evidence-to-Decision; CBT, cognitive behavioural therapy.

**Table 14:** Benefits and potential harms of epidural injections.

Treatment	Outcome (Instrument)	Time Frame	Effect Estimate	Conclusion	GRADE Certainty
Image-guided steroid + anaesthetic vs. non-invasive interventions	Pain (VAS) [93]	2 wks	MD −0.97 (95% CI −11.95 to 10.01)	Probably little to no difference	Moderate
Non-image-guided steroid + anaesthetic vs. NSAIDs	Pain (VAS) [94]	>4 mos	MD −0.8 (95% CI −1.49 to −0.11)	Reduces pain	Low
	Disability (ODI) [94]	≤4 mos	MD −4.1 (95% CI −8.9 to 0.7)	Probably improves disability	Low
	Healthcare use (analgesics/paracetamol) [94]	—	RR 0.55 (95% CI 0.20–1.50)	Probably little to no difference	Low
Non-image-guided steroid + anaesthetic vs. NSAIDs + opioids + muscle relaxants	Pain (VAS) [95]	>4 mos	MD −0.5 (95% CI −1.26 to 0.26)	Probably little to no difference	Low
	Minor adverse events (flushing, headache, backache) [95]	—	RR 1.25 (95% CI 0.38–4.12)	Probably little to no difference	Low
	Pain (VAS) [95]	≤4 mos	MD −0.5 (95% CI −1.23 to 0.23)	Very uncertain effect	Very low

Abbreviations: NSAIDs, non-steroidal anti-inflammatory drugs; VAS, Visual Analogue Scale; ODI, Oswestry Disability Index; wks, weeks; mos, months; MD, mean difference; CI, confidence interval; RR, risk ratio.

**Table 15:** Contextual factors for epidural injections.

GRADE EtD Domain	Evidence Identified	Task Force Judgement	Implications for Saudi Arabia
Values and preferences	No	Possible uncertainty and variability in preferences.	Variability in expectations should be anticipated.
Resource use and cost-effectiveness	No	Cost information from MoH (Supplementary Material S4). Personnel costs are the primary resource requirement; overall moderate cost impact; cost-effectiveness probably favours the intervention.	Costs depend on personnel availability; moderate cost impact expected.
Equity	No	Unequal access likely where infrastructure or trained personnel are limited.	Access gaps may arise between urban and rural regions.
Acceptability	No	Probably acceptable to patients, clinicians and decision-makers.	Likely acceptable where services are available.
Feasibility	No	Probably feasible in appropriately resourced facilities.	Feasible in specialist centres; more limited in rural areas.

Abbreviations: EtD, Evidence-to-Decision; MoH, Ministry of Health.

**Table 16:** Benefits of image-concordant pathology.

Predictor	Outcome (Instrument)	Time Frame	Effect Estimate	Conclusion	GRADE Certainty
Radicular symptoms [96]	Function (ODI) after open decompressive laminectomy	4 yrs	MD −4.2 (95% CI −6.33 to −2.07)	Predictor of functional outcome	Low
Radicular symptoms [98]	Leg pain (VAS)	1 yr	Adjusted OR 0.38 (95% CI 0.16–0.90)	Predictor of leg pain outcome	Low
Leg pain > back pain [97]	50% improvement in function (ODI)	1 yr	Adjusted OR 6.89 (95% CI 3.86–12.30)	Predictor of functional improvement	Low
Leg pain > back pain [97]	50% improvement in pain (VAS)	1 yr	Adjusted OR 2.77 (95% CI 2.01–3.82)	Predictor of pain improvement	Low

Abbreviations: ODI, Oswestry Disability Index; VAS, Visual Analogue Scale; yrs, years; yr, year; MD, mean difference; CI, confidence interval; OR, odds ratio.

**Table 17:** Contextual factors for image-concordant pathology.

GRADE EtD Domain	Evidence Identified	Task Force Judgement	Implications for Saudi Arabia
Values and preferences	No	Probably no important variability in patients’ values and preferences when using image-concordant pathology or radicular symptoms to predict response to surgery.	Stable values expected; major variability unlikely.
Resource use and cost-effectiveness	No	Cost information from MoH on imaging modalities (Supplementary Material S4). Resource needs relate to indirect imaging expenses, including equipment and human resources; performing MRI and establishing a diagnosis may reduce repeated consultations and procedures. Overall, resource requirements vary by setting and cost-effectiveness probably favours the intervention.	Costs and resource needs differ across settings; earlier diagnosis may reduce overall care costs.
Equity	No	Use of image-concordant pathology or radicular symptoms to guide surgery would probably reduce health equity due to delays and uneven access.	Regional disparities may emerge despite comprehensive national coverage.
Acceptability	No	Using image-concordant pathology or radicular symptoms to predict surgical response is probably acceptable to patients, clinicians and decision-makers.	Likely acceptable where services are available.
Feasibility	No	Using image-concordant pathology or radicular symptoms is considered feasible to implement in Saudi Arabia’s facilities and healthcare teams.	Feasible within existing healthcare teams and pathways.

Abbreviations: EtD, Evidence-to-Decision; MoH, Ministry of Health; MRI, magnetic resonance imaging.

**Table 18:** Benefits and potential harms of spinal decompression.

Treatment	Outcome (Instrument)	Time Frame	Effect Estimate	Conclusion	GRADE Certainty
Discectomy vs. usual care	Leg pain (VAS) [101,102]	>4 mos to 1 yr	MD −0.57 (95% CI −0.87 to −0.28)	Probably improves leg pain	Low
	Back pain (VAS) [101,102]	>4 mos to 1 yr	MD −0.23 (95% CI −0.28 to −0.18)	Probably improves back pain	Low
	Back pain (SBI) [100]	>4 mos to 1 yr	MD −1.6 (95% CI −2.86 to −0.34)	Probably improves back pain	Low
	Function (ODI) [100,102]	>4 mos to 1 yr	MD −2.58 (95% CI −6.47 to 1.3)	Probably little to no difference	Low
	Adverse events (mortality) [100]	—	RR 0.15 (95% CI 0.01–2.87)	Probably little to no difference	Low
	Quality of life (SF-36—bodily pain) [100]	≤4 mos	MD 8.35 (95% CI 7.87–8.83)	Very uncertain effect	Very low
	Quality of life (SF-36—bodily pain) [100]	2 yrs	MD 3.2 (95% CI −2.07 to 8.47)	Very uncertain effect	Very low
	Quality of life (SF-36—physical functioning) [100,101]	≤4 mos	MD 9.26 (95% CI 8.84–9.68)	Very uncertain effect	Very low
	Quality of life (SF-36—physical functioning) [100]	2 yrs	MD 0 (95% CI −5.41 to 5.41)	Very uncertain effect	Very low
	Healthcare utilisation (PT visits) [102]	>4 mos to 2 yrs	RR 0.49 (95% CI 0.26–0.95)	Very uncertain effect	Very low
	Adverse events (morbidity) [100]	—	RR 1.00 (95% CI 0.37–2.73)	Very uncertain effect	Very low
Discectomy vs. combination therapy	Pain (MPQ) [103]	≤4 mos	MD −6.4 (95% CI −15.9 to 3.1)	Probably little to no difference	Low
	Function (RMDQ) [103]	≤4 mos	MD −1.8 (95% CI −5.87 to 2.27)	Probably little to no difference	Low
	Quality of life (SF-36—bodily pain) [103]	≤4 mos	MD 10.3 (95% CI −2.37 to 22.97)	Probably little to no difference	Low
	Quality of life (SF-36—physical function) [103]	≤4 mos	MD 6.8 (95% CI −9.64 to 23.24)	Very uncertain effect	Very low
Percutaneous decompression vs. usual care	Leg pain (VNS) [104]	4 mos to 2 yrs	MD −3.1 (95% CI −4.45 to −1.75)	Probably improves pain	Low
Plasma disc decompression vs. epidural steroid injection	Leg pain (VAS) [105]	6 mos	MD −1.8 (95% CI −3.05 to −0.55)	Probably improves pain	Low
	Back pain (VAS) [105]	6 mos	MD −1.62 (95% CI −2.73 to −0.51)	Probably improves pain	Low
	Function (ODI) [105]	6 mos	MD −1.6 (95% CI −2.31 to −0.89)	Probably improves function	Low
	Adverse events (procedure-related) [105]	6 mos	RR 0.63 (95% CI 0.22–1.84)	May be little to no difference	Low
	Adverse events (morbidity) [105]	—	RR 0.48 (95% CI 0.04–5.09)	Very uncertain effect	Very low
	Adverse events (mortality) [105]	—	RR 0.96 (95% CI 0.06–14.83)	Very uncertain effect	Very low
Laminectomy vs. usual care	Pain (LBPBI) [99]	1 yr	MD 0 (95% CI −0.55 to 0.55)	Little to no difference	Low
	Function (ODI) [99]	1 yr	MD −2.2 (95% CI −7.33 to 2.93)	Little to no difference	Low
	Quality of life (SF-36—bodily pain) [99]	1 yr	MD 5.5 (95% CI −0.74 to 11.74)	Little to no difference	Low
	Quality of life (SF-36—physical functioning) [99]	1 yr	MD 1.6 (95% CI −4.64 to 7.84)	Little to no difference	Low
	Adverse events (mortality) [99]	—	RR 0.94 (95% CI 0.32–2.72)	Little to no difference	Low
	Pain (SBI) [99]	1 yr	MD −0.6 (95% CI −1.15 to −0.05)	Very uncertain effect	Very low

Abbreviations: VAS, Visual Analogue Scale; SBI, Sciatica Bothersomeness Index; ODI, Oswestry Disability Index; SF-36, 36-Item Short Form Survey; MPQ, McGill Pain Questionnaire; RMDQ, Roland–Morris Disability Questionnaire; VNS, Visual Numeric Scale; LBPBI, Low Back Pain Bothersomeness Index; mos, months; yrs, years; yr, year; MD, mean difference; CI, confidence interval; RR, risk ratio.

**Table 19:** Contextual factors for spinal decompression.

GRADE EtD Domain	Evidence Identified	Task Force Judgement	Implications for Saudi Arabia
Values and preferences	No	Probably no important variability in preferences.	Preferences expected to be stable.
Resource use and cost-effectiveness	Yes	Three cost-utility analyses [106,107,108] and MoH cost data for laminectomy and discectomy (Supplementary Material S4); spinal decompression associated with high resource requirements and cost-effectiveness generally unfavourable, though diagnosis-specific variation may occur.	Surgical decompression is resource-intensive; cost-effectiveness generally does not support its use over usual care.
Equity	No	Reduced equity likely where specialised personnel and facilities are limited.	Regional access disparities may occur despite national coverage.
Acceptability	No	Probably acceptable to patients, clinicians and decision-makers.	Acceptability high where surgical capacity exists.
Feasibility	No	Probably feasible in most settings but limited by regional resource constraints.	Feasible in major centres; constrained in resource-restricted regions.

Abbreviations: EtD, Evidence-to-Decision; MoH, Ministry of Health.

**Table 20:** Benefits and potential harms of radiofrequency denervation.

Treatment	Outcome (Instrument)	Time Frame	Effect Estimate	Conclusion	GRADE Certainty
RF denervation vs. placebo/sham	Pain (VAS) [109,110,111,112]	≤4 mos	MD −1.83 (95% CI −2.41 to −1.24)	Probably improves pain	Moderate
	Pain (VAS) [109,110,113]	>4 mos	MD −1.57 (95% CI −2.20 to −0.95)	Probably improves pain	Low
	Quality of life (SF-36—vitality) [114]	≤4 mos	MD 7.70 (95% CI 0.64 to 14.76)	Probably improves vitality	Low
	Responder criteria (>50% pain reduction or favourable global perceived effect) [112,114]	≤4 mos	RR 1.74 (95% CI 1.15 to 2.63)	Probably improves responder rate	Moderate
	Adverse events (treatment-related pain, moderate or severe) [114]	≤4 mos	RR 1.64 (95% CI 1.00 to 2.69)	Probably worse outcomes	Low
	Quality of life (SF-36—general health) [114]	≤4 mos	MD 3.10 (95% CI −3.72 to 9.92)	Little to no difference	Moderate
	Quality of life (SF-36—mental health) [114]	≤4 mos	MD 2.00 (95% CI −9.07 to 13.07)	Little to no difference	Low
	Quality of life (SF-36—social functioning) [114]	≤4 mos	MD 2.70 (95% CI −11.70 to 17.10)	Little to no difference	Low
	Quality of life (SF-36—physical functioning) [114]	≤4 mos	MD −3.10 (95% CI −11.09 to 4.89)	Little to no difference	Low
	Function (RMDQ) [111]	≤4 mos	MD 2.6 (95% CI −6.21 to 11.41)	Very uncertain effect	Very low
	Quality of life (SF-36—pain subscale) [114]	≤4 mos	MD 0.20 (95% CI −9.29 to 9.69)	Very uncertain effect	Very low
	Healthcare utilisation (analgesic tablets over 4 days) [112]	≤4 mos	MD −3.24 (95% CI −6.60 to 0.12)	Very uncertain effect	Very low
	Healthcare utilisation (global perception of improvement) [113]	>4 mos	MD −0.8 (95% CI −1.56 to −0.04)	Very uncertain effect	Very low
	Responder criteria (>50% pain reduction/global effect) [112]	>4 mos	RR 3.73 (95% CI 0.92 to 15.21)	Very uncertain effect	Very low
	Adverse events (sensory change: dysaesthesia/allodynia) [114]	<4 mos	RR 5.13 (95% CI 0.25 to 103.45)	Very uncertain effect	Very low
	Adverse events (loss of motor function) [114]	≤4 mos	RR 0.36 (95% CI 0.02 to 8.55)	Very uncertain effect	Very low
RF denervation vs. medial branch block	Pain (VNS) [115]	≤4 mos	MD −1.2 (95% CI −1.79 to −0.61)	Very uncertain effect	Very low
	Pain (VNS) [115]	>4 mos	MD −2.3 (95% CI −3.42 to −1.18)	Very uncertain effect	Very low
	Quality of life (EQ-5D) [115]	≤4 mos	MD −0.4 (95% CI −0.97 to 0.17)	Very uncertain effect	Very low
	Quality of life (EQ-5D) [115]	>4 mos	MD −1.3 (95% CI −2.87 to 0.27)	Very uncertain effect	Very low

Abbreviations: RF, radiofrequency; VAS, Visual Analogue Scale; SF-36, 36-Item Short Form Survey; RMDQ, Roland–Morris Disability Questionnaire; VNS, Visual Numeric Scale; EQ-5D, EuroQol 5-Dimension; mos, months; MD, mean difference; CI, confidence interval; RR, risk ratio.

**Table 21:** Contextual factors for radiofrequency denervation.

GRADE EtD Domain	Evidence Identified	Task Force Judgement	Implications for Saudi Arabia
Values and preferences	No	Probably no important uncertainty or variability in preferences.	Preferences are unlikely to limit use in appropriately selected patients.
Resource use and cost-effectiveness	Yes	Within-trial economic analysis [114], alongside the NICE economic model [37] and a Dutch societal evaluation [116], indicated moderate costs. These analyses suggested potential cost-effectiveness in appropriately selected patients, though uncertainty and methodological limitations were noted. Overall, cost-effectiveness probably favours the intervention.	Radiofrequency denervation probably cost-effective when used for well-selected facet joint pain after failed conservative care.
Equity	No	Probably no major impact on equity overall.	Comprehensive coverage likely offsets inequities, although service availability may vary by region.
Acceptability	No	Probably acceptable to patients, clinicians and decision-makers.	Likely acceptable where services are available.
Feasibility	No	Might be feasible in centres with trained clinicians and appropriate facilities.	

Abbreviations: EtD, Evidence-to-Decision; NICE, National Institute for Health and Care Excellence.

**Table 22:** Benefits of pain neuroscience education.

Treatment	Outcome (Instrument)	Time Frame	Effect Estimate	Conclusion	GRADE Certainty
PNE vs. no PNE	Disability (RMDQ) [117,118,119,120,121]	32.8 days	MD 2.28 (95% CI 0.30 to 4.25)	Improves disability	Moderate
	Pain (NRS) [117,118,120,121,122,123]	31.8	MD 0.73 (95% CI −0.14 to 1.61)	May be little to no effect	Low
	Pain (NRS) [118,120]	12 mos	MD 0.44 (95% CI −1.03 to 1.91)	May be little to no effect	Low
	Psychological effects (TSK) [121,122,124]	3.3 wks	MD 4.72 (95% CI 2.32 to 7.13)	May be little to no effect	Low
	Disability (RMDQ) [118,120]	12 mos	MD 2.18 (95% CI −0.67 to 5.02)	May be little to no effect	Low
PNE + physiotherapy vs. no PNE	Disability (RMDQ) [117,120,121]	40 days	MD 3.94 (95% CI 3.37 to 4.52)	Improves disability	Moderate
	Pain (NRS) [117,120,121,122,123]	32.6 days	MD 1.32 (95% CI 1.08 to 1.56)	Improves pain	Moderate

Abbreviations: PNE, pain neuroscience education; RMDQ, Roland–Morris Disability Questionnaire; NRS, Numeric Rating Scale; TSK, Tampa Scale for Kinesiophobia; mos, months; wks, weeks; MD, mean difference; CI, confidence interval.

**Table 23:** Contextual factors for pain neuroscience education.

GRADE EtD Domain	Evidence Identified	Task Force Judgement	Implications for Saudi Arabia
Values and preferences	No	Probably no important variability in how patients value outcomes	Variability unlikely; expectations generally stable
Resource use and cost-effectiveness	No	Moderate costs based on training, materials, travel, appointments; telehealth may reduce burden; cost-effectiveness judged neutral	Moderate resource needs; telehealth may ease access; no clear cost-effectiveness advantage
Equity	No	Equity may be reduced where rural access and older-adult support are limited	Regional and age-related disparities possible despite national coverage
Acceptability	Yes	Evidence from the GLITtER feasibility trial [125] showed psychoeducation to be acceptable, easily integrated into consultations, and beneficial for patients and clinicians. However, the Task Force considered that pain neuroscience education may increase clinician workload and is therefore probably not acceptable to key stakeholders.	Acceptability may be limited unless integrated without adding workload
Feasibility	No	Probably feasible with training, materials and appointment availability, but dependent on infrastructure	Feasible in most facilities with adequate workforce and infrastructure.

Abbreviations: EtD, Evidence-to-Decision; GLITtER, Green Light Imaging Intervention to Enhance Recovery.

## Data Availability

The original contributions presented in the study are included in the article; further inquiries can be directed at the corresponding author.

## Supplementary Materials

The following supporting information can be downloaded at: https://www.mdpi.com/article/10.3390/jcm15020528/s1, Supplementary Material S1: Search Strategy Methods; Supplementary Material S2: Forest Plots; Supplementary Material S3: Risk Assessment Tools and Stratification; Supplementary Material S4: Cost Tables. Refs. [42,43,44,45,125,126,127,128,129,130,131,132,133,134,135,136,137] are cited in Supplementary Material S3 and Ref. [138] is cited in Supplementary Material S4.

## Abbreviations

The following abbreviations are used in this manuscript:

LBP	Low back pain
MoH	Ministry of Health
GBD	Global Burden of Disease
NICE	National Institute for Health and Care Excellence
EtD	Evidence-to-Decision
RCTs	Randomized controlled trials
SBST	STarT Back Screening Tool
AUC	Under the curve
R^2^	R-squared
ODI	Oswestry Disability Index
CPRIS	Chronic Pain Risk Item Set
LBPPS	Low Back Pain Perception Scale
ÖMPQ	Modified Örebro Musculoskeletal Pain Questionnaire
mos	Months
mo	Month
CI	Confidence interval
SF-12	12-Item Short Form Survey
VAS	Visual Analogue Scale
HADS	Hospital Anxiety and Depression Scale
RMDQ	Roland–Morris Disability Questionnaire
MD	Mean difference
RR	Risk ratio
QALYs	Quality-adjusted life years
X-ray	Plain radiography
MRI	Magnetic resonance imaging
SF-36	36-Item Short Form Survey
ALBPS	Aberdeen Low Back Pain Score
y	Year
NSAIDs	Non-steroidal anti-inflammatory drugs
NRS	Numeric Rating Scale
EQ-5D	EuroQol 5-Dimension
BDI	Beck Depression Inventory
HR	Hazard ratio
CBT	Cognitive behavioural therapy
MBIs	Mindfulness-based interventions
CT	Cognitive therapy
MPQ	McGill Pain Questionnaire
PAIRS	Pain and Impairment Relationship Scale
PDI	Pain Disability Index
HSI	Health Status Inventory
wks	Weeks
BMI	Body mass index
yrs	Years
OR	Odds ratio
SBI	Sciatica Bothersomeness Index
VNS	Visual Numeric Scale
LBPBI	Low Back Pain Bothersomeness
RF	Radiofrequency
PNE	Pain neuroscience education;
TSK	Tampa Scale for Kinesiophobia
GLITtER	Green Light Imaging Intervention to Enhance Recovery
