# Supplementary material for: Saudi Clinical Practice Guideline for the Assessment and Management of Low Back Pain and Sciatica in Adults
Source: J Clin Med. 2026 Jan 8;15(2):528. doi: 10.3390/jcm15020528 (PMC12842004; doi:10.3390/jcm15020528)
Supplement: Supplementary file 1 [file jcm-15-00528-s001.zip › Supplementary Material S1.pdf]

Supplementary Material S1: Search Strategy Methods

This supplementary material contains the search strategies used to update the literature searches from NICE guideline NG59: *"Low back pain and sciatica in over 16s: assessment and management"* [37], as well as the additional searches conducted to identify relevant evidence on local contextual factors.

1.1. Clinical questions

Question 1: Should validated risk assessment tools versus no validated risk assessment tools be used for screening patients with LBP and/or sciatica who are at risk of poor outcome or delayed improvement?

Databases searched: PubMed, Embase, and Cochrane

Search period: 01 January 2016 to 04 May 2022 (PubMed and Embase); January 2016 to May 2022 (Cochrane)

Search strategy used:

PubMed: ((((("Low Back Pain"[Mesh] OR "Sciatica"[Mesh] OR "Low Backache*"[tiab] OR "Lower Backache*"[tiab] OR Lumbago[tiab] OR Sciatic* [tiab] OR (("Lumbar Vertebrae"[Mesh] OR "Lumbosacral Region"[Mesh] OR "Low Back"[tiab] OR "Lower Back"[tiab] OR Lumbar[tiab] OR Lumbosacral[tiab] OR "Lumbo-sacral"[tiab]) AND ("Pain"[Mesh] OR Ache*[tiab] OR Aching[tiab] OR Pain*[tiab])) OR (("Radiculopathy"[Mesh] OR "Radicular Pain"[tiab] OR Radiculitides[tiab] OR Radiculitis[tiab] OR Radiculopath*[tiab] OR (("Spinal Nerve Roots"[Mesh] OR "Nerve Root"[tiab]) AND ("Inflammation"[Mesh] OR "Pain"[Mesh] OR Ache*[tiab] OR Aching[tiab] OR Avulsion*[tiab] OR Compress*[tiab] OR Disorder*[tiab] OR Entrap*[tiab] OR Imping*[tiab] OR Inflam*[tiab] OR Irritat*[tiab] OR Pinch*[tiab] OR Trap*[tiab]))) AND ("Low Back Pain"[Mesh] OR "Lumbar Vertebrae"[Mesh] OR "Lumbosacral Region"[Mesh] OR Low[tiab] OR Lower[tiab] OR Lumbar[tiab] OR Lumbosacral[tiab] OR "Lumbo-sacral"[tiab]))) AND (valid*[tiab] AND ("Risk Assessment"[Mesh] OR delitto[tiab] OR dram[tiab] OR flynn[tiab] OR gatchel[tiab] OR hancock[tiab] OR orebro [tiab] OR "risk analys*"[tiab] OR "risk assessment*"[tiab] OR "risk-benefit assessment"[tiab] OR sullivan[tiab] OR "start back"[tiab] OR Startback[tiab] OR Childs JD[Author] OR Delitto A[Author] OR Flynn T[Author] OR Gatchel RJ[Author] OR Hancock MJ[Author] OR O'Sullivan P[Author] OR O'Sullivan PB[Author] OR ((consequence*[tiab] OR damag*[tiab] OR exposure*[tiab] OR harm*[tiab] OR hazard*[tiab] OR impair*[tiab] OR risk*[tiab] OR threat*[tiab] OR vulnerab*[tiab]) AND (algorithm[tiab] OR analys*[tiab] OR assess*[tiab] OR calculat*[tiab] OR criteria[tiab] OR equation*[tiab] OR index*[tiab] OR instrument*[tiab] OR predict*[tiab] OR questionnaire*[tiab] OR rule*[tiab] OR score*[tiab] OR scoring[tiab] OR screen*[tiab] OR technique*[tiab] OR test*[tiab] OR tool*[tiab])))) AND ("Sensitivity and Specificity"[Mesh] OR accura*[tiab] OR "predictive value*"[tiab] OR "roc analys*"[tiab] OR "roc curve"[tiab] OR sensitiv*[tiab] OR specific*[tiab]) AND (2016/1/1:2022/5/4[pdat]) AND (english[Filter]) AND ("Cohort Studies"[Mesh] OR "cohort analys*"[tiab] OR "cohort stud*"[tiab] OR "concurrent stud*"[tiab] OR "incidence stud*"[tiab] OR "Randomized Controlled Trial"[Publication Type] OR placebo[tiab] OR random*[tiab] OR trial*[ti] OR "Meta-Analysis"[Publication Type] OR "Systematic Review"[Publication Type] OR "Systematic Reviews as Topic"[Mesh] OR metaanalys*[ti] OR meta-analys*[ti] OR "meta analys*"[ti] OR "systematic review"[ti] OR trial*[ti] OR ((meta[ti] OR systematic*[ti]) AND (overview*[ti] OR review*[ti] OR synthesis*[ti])))) NOT ("Chromatography"[Mesh] OR "Chromosomes"[Mesh] OR "Genetics"[Mesh] OR "Genome"[Mesh] OR "Genomics"[Mesh] OR "Genotype"[Mesh] OR "Mass Spectrometry"[Mesh] OR "Microbiota"[Mesh] OR "Mutation"[Mesh] OR "Pharmacokinetics"[Mesh] OR "Polymorphism,

Genetic"[Mesh] OR "congenital" [Subheading] OR "genetics"[Subheading] OR "pharmacokinetics"[Subheading] OR assay*[tiab] OR cell[tiab] OR cells[tiab] OR chromosome*[tiab] OR genetic*[tiab] OR genome*[tiab] OR genomic*[tiab] OR "in vitro"[tiab] OR microbiom*[tiab] OR microbiota*[tiab] OR mutat*[tiab] OR pharmacokinetic*[tiab] OR polymorphism*[tiab] OR SNP[tiab] OR spectrometry[tiab] OR tissue*[tiab])) NOT (("Animals"[Mesh] OR animal*[tiab] OR ape[tiab] OR apes[tiab] OR canine*[tiab] OR cat[tiab] OR cats[tiab] OR chimpanzee*[tiab] OR dog[tiab] OR dogs[tiab] OR feline*[tiab] OR hamster*[tiab] OR lamb*[tiab] OR mice[tiab] OR monkey*[tiab] OR mouse[tiab] OR murine[tiab] OR pig[tiab] OR pigs[tiab] OR piglet*[tiab] OR porcine[tiab] OR primate*[tiab] OR rabbit*[tiab] OR rat[tiab] OR rats[tiab] OR rodent*[tiab] OR sheep*[tiab] OR swine[tiab]) NOT ("Humans"[Mesh] OR human*[tiab] OR man[tiab] OR men[tiab] OR patient*[tiab] OR woman[tiab] OR women[tiab]))) NOT ("address"[Publication Type] OR "autobiography"[Publication Type] OR "bibliography"[Publication Type] OR "biography"[Publication Type] OR "Book Illustrations"[Publication Type] OR "Case Reports"[Publication Type] OR "Comment"[Publication Type] OR "congress"[Publication Type] OR "consensus development conference"[Publication Type] OR "consensus development conference, nih"[Publication Type] OR "dictionary"[Publication Type] OR "directory"[Publication Type] OR "editorial"[Publication Type] OR "Expression of Concern"[Publication Type] OR "Guideline"[Publication Type] OR "interactive tutorial"[Publication Type] OR "interview"[Publication Type] OR "lecture"[Publication Type] OR "legal case"[Publication Type] OR "legislation"[Publication Type] OR "letter"[Publication Type] OR "Meta-Analysis"[Publication Type] OR "news"[Publication Type] OR "newspaper article"[Publication Type] OR "overall"[Publication Type] OR "patient education handout"[Publication Type] OR "periodical index"[Publication Type] OR "personal narrative"[Publication Type] OR "portrait"[Publication Type] OR "Review"[Publication Type] OR "Scientific Integrity Review"[Publication Type] OR "Systematic Review"[Publication Type] OR "hascommenton"[All Fields] OR "Cartoons as Topic"[Mesh] OR "Meta-Analysis as Topic"[Mesh] OR "Review Literature as Topic"[Mesh] OR "Systematic Reviews as Topic"[Mesh] OR "case report*"[tiab] OR "case series"[tiab] OR "integrative research review*"[tiab] OR "integrative review*"[tiab] OR "literature review"[tiab] OR meta-analys*[tiab] OR "meta analys*"[tiab] OR metaanalys*[tiab] OR "narrative review"[tiab] OR "research integration"[tiab] OR "scoping review"[tiab] OR ((methodologic*[tiab] OR quantitative*[tiab] OR systematic*[tiab]) AND (overview*[tiab] OR review*[tiab] OR synthesis*[tiab]))))

Embase: ((((('low back pain'/exp OR 'sciatica'/exp OR (("Low Backache*" OR "Lower Backache*" OR Lumbago OR Sciatic*):ti,ab) OR (('lumbar vertebra'/exp OR 'lumbosacral region'/exp OR (("Low Back" OR "Lower Back" OR Lumbar OR Lumbosacral OR "Lumbo-sacral"):ti,ab)) AND ('pain'/exp OR ((Ache* OR Aching OR Pain*):ti,ab))) OR (('radicular pain'/exp OR 'radiculopathy'/exp OR (("Radicular Pain" OR Radiculitides OR Radiculitis OR Radiculopath*):ti,ab) OR (('nerve root'/exp OR "Nerve Root":ti,ab) AND ('inflammation'/exp OR 'pain'/exp OR ((Ache* OR Aching OR Avulsion* OR Compress* OR Disorder* OR Entrap* OR Imping* OR Inflam* OR Irritat* OR Pinch* OR Trap*):ti,ab)))) AND ('low back pain'/exp OR 'lumbar vertebra'/exp OR 'lumbosacral region'/exp OR ((Low OR Lower OR Lumbar OR Lumbosacral OR "Lumbo-sacral"):ti,ab)))) AND (valid*:ti,ab AND ('risk assessment'/exp OR ((delitto OR dram OR flynn OR gatchel OR hancock OR Orebro OR "risk analys*" OR "risk assessment*" OR "risk-benefit assessment" OR sullivan OR "start back" OR Startback):ti,ab) OR Childs J D:au OR Delitto A:au OR Flynn T:au OR Gatchel R J:au OR Hancock M J:au OR OSullivan P:au OR OSullivan P B:au OR (((consequence* OR damag* OR exposure* OR harm* OR hazard* OR impair* OR risk* OR threat* OR vulnerab*):ti,ab) AND ((algorithm OR analys* OR assess* OR calculat* OR criteria OR equation* OR index* OR instrument* OR predict* OR questionnaire* OR rule* OR score* OR scoring OR screen* OR technique* OR test* OR tool*):ti,ab)))) AND ('accuracy'/exp OR 'predictive value'/exp OR 'sensitivity'/exp OR 'sensitivity and specificity'/exp OR 'specificity'/exp OR ((accura* OR "predictive value*" OR "roc analys*" OR "roc curve" OR sensitiv* OR specific*):ti,ab)) AND ([1-1-2016]/sd NOT [5-5-2022]/sd) AND ([english]/lim) AND ('cohort analysis'/exp OR "cohort analys*":ti,ab OR "cohort stud*":ti,ab OR "concurrent stud*":ti,ab OR "incidence stud*":ti,ab OR 'randomized controlled trial'/exp OR placebo:ti,ab OR random*:ti,ab OR trial*:ti OR 'meta analysis'/exp OR 'meta analysis topic'/exp OR 'systematic review'/exp OR 'systematic review (topic)'/exp OR metaanalys*:ti OR meta-analys*:ti OR

"meta analys*":ti OR "systematic review":ti OR trial*:ti OR (((meta OR systematic*) AND (overview* OR review* OR synthesis*)):ti))) NOT ('chromatography'/exp OR 'chromosome'/exp OR 'congenital'/exp OR 'genetic polymorphism'/exp OR 'genetics'/exp OR 'genome'/exp OR 'genomics'/exp OR 'genotype'/exp OR 'mass spectrometry'/exp OR 'microbiome'/exp OR 'mutation'/exp OR 'pharmacokinetics'/exp OR 'polymorphism'/exp OR assay*:ti,ab OR cell:ti,ab OR cells:ti,ab OR chromosome*:ti,ab OR genetic*:ti,ab OR genome*:ti,ab OR genomic*:ti,ab OR "in vitro":ti,ab OR microbiom*:ti,ab OR microbiota*:ti,ab OR mutat*:ti,ab OR pharmacokinetic*:ti,ab OR polymorphism*:ti,ab OR SNP:ti,ab OR spectrometry:ti,ab OR tissue*:ti,ab)) NOT (('animal'/exp OR animal*:ti,ab OR ape:ti,ab OR apes:ti,ab OR canine*:ti,ab OR cat:ti,ab OR cats:ti,ab OR chimpanzee*:ti,ab OR dog:ti,ab OR dogs:ti,ab OR feline*:ti,ab OR hamster*:ti,ab OR lamb*:ti,ab OR mice:ti,ab OR monkey*:ti,ab OR mouse:ti,ab OR murine:ti,ab OR pig:ti,ab OR pigs:ti,ab OR piglet*:ti,ab OR porcine:ti,ab OR primate*:ti,ab OR rabbit*:ti,ab OR rat:ti,ab OR rats:ti,ab OR rodent*:ti,ab OR sheep*:ti,ab OR swine:ti,ab) NOT ('human'/exp OR human*:ti,ab OR man:ti,ab OR men:ti,ab OR patient*:ti,ab OR woman:ti,ab OR women:ti,ab))) NOT (('abstract report'/exp OR 'animal experiment'/exp OR 'book'/exp OR 'case finding'/exp OR 'case report'/exp OR 'case study'/exp OR 'conference paper'/exp OR 'editorial'/exp OR 'feasibility study'/exp OR 'in vitro study'/exp) AND 'letter'/exp OR 'meta analysis'/exp OR 'meta analysis topic'/exp OR 'meta analysis (topic)'/exp OR 'note'/exp OR 'practice guideline'/exp OR 'review'/exp OR 'systematic review'/exp OR 'systematic review topic'/exp OR 'systematic review (topic)'/exp OR 'veterinary clinical trial'/exp OR 'veterinary study'/exp OR [conference abstract]/lim OR [conference paper]/lim OR [conference review]/lim OR [editorial]/lim OR [letter]/lim OR [note]/lim OR [short survey]/lim OR 'case report*':ti,ab OR 'case series':ti,ab OR 'integrative research review*':ti,ab OR 'integrative review*':ti,ab OR 'literature review':ti,ab OR 'meta analys*':ti,ab OR metaanalys*:ti,ab OR meta*analys*:ti,ab OR 'narrative review':ti,ab OR 'research integration':ti,ab OR 'scoping review':ti,ab OR ((integrative NEAR/5 research NEAR/5 review*):ti,ab) OR ((methodologic* NEAR/5 overview*):ti,ab) OR ((methodologic* NEAR/5 review*):ti,ab) OR ((quantitativ* NEAR/5 overview*):ti,ab) OR ((quantitativ* NEAR/5 review*):ti,ab) OR ((quantitativ* NEAR/5 synthesi*):ti,ab) OR ((research NEAR/5 integration):ti,ab) OR ((systematic* NEAR/5 overview*):ti,ab) OR ((systematic* NEAR/5 review*):ti,ab)))

Cochrane:

**ID**	**Search**
#1	MeSH descriptor: [Low Back Pain] explode all trees
#2	MeSH descriptor: [Sciatica] explode all trees
#3	(("Low Backache*" OR "Lower Backache*" OR Lumbago OR Sciatic*):ti,ab)
#4	MeSH descriptor: [Lumbar Vertebrae] explode all trees
#5	MeSH descriptor: [Lumbosacral Region] explode all trees
#6	(("Low Back" OR "Lower Back" OR Lumbar OR Lumbosacral OR "Lumbo-sacral"):ti,ab)
#7	OR/#4-#6
#8	MeSH descriptor: [Pain] explode all trees
#9	((Ache* OR Aching OR Pain*):ti,ab)
#10	#8 OR #9
#11	#7 AND #10
#12	MeSH descriptor: [Radiculopathy] explode all trees
#13	(("Radicular Pain" OR Radiculitides OR Radiculitis OR Radiculopath*):ti,ab)
#14	#12 OR #13
#15	MeSH descriptor: [Spinal Nerve Roots] explode all trees
#16	"Nerve Root":ti,ab

#17 #15 OR #16

#18 MeSH descriptor: [Inflammation] explode all trees

#19 MeSH descriptor: [Pain] explode all trees

#20 ((Ache* OR Aching OR Avulsion* OR Compress* OR Disorder* OR Entrap* OR Imping* OR Inflam* OR Irritat* OR Pinch* OR Trap*):ti,ab)

#21 OR/#18-#20

#22 #14 OR #17 OR #20

#23 MeSH descriptor: [Low Back Pain] explode all trees

#24 MeSH descriptor: [Lumbar Vertebrae] explode all trees

#25 MeSH descriptor: [Lumbosacral Region] explode all trees

#26 ((Low OR Lower OR Lumbar OR Lumbosacral OR "Lumbo-sacral"):ti,ab)

#27 OR/#23-#26

#28 #22 AND #27

#29 #1 OR #2 OR #3 OR #11 OR #28

#30 valid*:ti,ab

#31 MeSH descriptor: [Risk Assessment] explode all trees

#32 ((delitto OR dram OR flynn OR gatchel OR hancock OR orebro OR "risk analys*" OR "risk assessment*" OR "risk-benefit assessment" OR sullivan OR "start back" OR Startback):ti,ab) OR Childs JD:au OR Delitto A:au OR Flynn T:au OR Gatchel RJ:au OR Hancock MJ:au OR O'Sullivan P:au OR O'Sullivan PB:au OR (((consequence* OR damag* OR exposure* OR harm* OR hazard* OR impair* OR risk* OR threat* OR vulnerab*):ti,ab) AND ((algorithm OR analys* OR assess* OR calculat* OR criteria OR equation* OR index* OR instrument* OR predict* OR questionnaire* OR rule* OR score* OR scoring OR screen* OR technique* OR test* OR tool*):ti,ab))

#34 #31 OR #32

#35 #30 AND #34

#36 MeSH descriptor: [Sensitivity and Specificity] explode all trees

#37 ((accura* OR "predictive value*" OR "roc analys*" OR "roc curve" OR sensitiv* OR specific*):ti,ab)

#38 #36 OR #37

#39 #29 AND #35 AND #38

#40 MeSH descriptor: [Biomarkers] explode all trees

#41 MeSH descriptor: [Chromatography] explode all trees

#42 MeSH descriptor: [Chromosomes] explode all trees

#43 MeSH descriptor: [Echocardiography] explode all trees

#44 MeSH descriptor: [Genetics] in all MeSH products

#45 MeSH descriptor: [Genome] in all MeSH products

#46 MeSH descriptor: [Genotype] explode all trees

#47 MeSH descriptor: [Incidence] explode all trees

#48 MeSH descriptor: [Mass Spectrometry] explode all trees

#49 MeSH descriptor: [Microbiota] explode all trees

#50 MeSH descriptor: [Mutation] explode all trees

#51 MeSH descriptor: [Pharmacokinetics] explode all trees

#52 MeSH descriptor: [Polymorphism, Genetic] explode all trees

#53 Any MeSH descriptor in all MeSH products and with qualifier(s): [congenital - CN]

#54 Any MeSH descriptor in all MeSH products and with qualifier(s): [genetics - GE]

#55 Any MeSH descriptor in all MeSH products and with qualifier(s): [pharmacokinetics - PK]

#56 ((assay* OR biomarker* OR cell OR cells OR chromosome* OR genetic* OR genome* OR genomic* OR "in vitro" OR microbiom* OR microbiota* OR mutat* OR pharmacokinetic* OR polymorphism* OR SNP OR spectrometry OR tissue*):ti,ab)

#57 OR/#40-#56

#58 #39 NOT #57

#59 #58 AND publication date between January 2016 and May 2022

Eligibility criteria:

- Inclusion criteria: ***A) Population:*** People aged 16 or above with non-specific low back pain; People aged 16 or above with sciatica. ***B) Intervention:*** Validated risk assessment/clinical prediction tools including STarT Back, DRAM, ÖREBRO. ***C) Comparison:*** All interventions will be compared with each other. ***D) Study Type:*** Randomized controlled trials, cohort studies, and systematic reviews.
- Exclusion criteria: None.

PRISMA flow diagram:

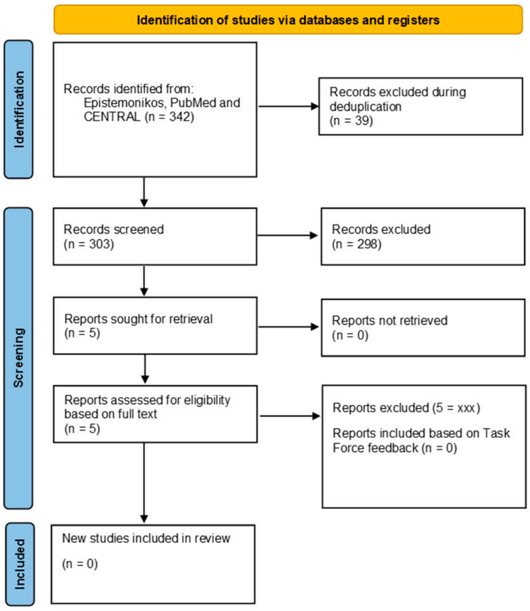

Question 2: Should validated risk assessment or clinical prediction tools, compared with no tools or with each other, be used to stratify the management of patients with non-specific LBP and/or sciatica based on the outcome of the tool or questionnaire?

Databases searched: PubMed, Embase, and Cochrane

Search period: 01 January 2016 to 29 April 2022 (PubMed and Embase); January 2016 to April 2022 (Cochrane)

Search strategy used:

PubMed: ((((("Low Back Pain"[Mesh] OR "Sciatica"[Mesh] OR "Low Backache*"[tiab] OR "Lower Backache*"[tiab] OR Lumbago[tiab] OR Sciatic* [tiab] OR (("Lumbar Vertebrae"[Mesh] OR "Lumbosacral Region"[Mesh] OR "Low Back"[tiab] OR "Lower Back"[tiab] OR Lumbar[tiab] OR Lumbosacral[tiab] OR "Lumbo-sacral"[tiab]) AND ("Pain"[Mesh] OR Ache*[tiab] OR Aching[tiab] OR Pain*[tiab])) OR (("Radiculopathy"[Mesh] OR "Radicular Pain"[tiab] OR Radiculitides[tiab] OR Radiculitis[tiab] OR Radiculopath*[tiab] OR (("Spinal Nerve Roots"[Mesh] OR "Nerve Root"[tiab]) AND ("Inflammation"[Mesh] OR "Pain"[Mesh] OR Ache*[tiab] OR Aching[tiab] OR Avulsion*[tiab] OR Compress*[tiab] OR Disorder*[tiab] OR Entrap*[tiab] OR Imping*[tiab] OR Inflam*[tiab] OR Irritat*[tiab] OR Pinch*[tiab] OR Trap*[tiab]))) AND ("Low Back Pain"[Mesh] OR "Lumbar Vertebrae"[Mesh] OR "Lumbosacral Region"[Mesh] OR Low[tiab] OR Lower[tiab] OR Lumbar[tiab] OR Lumbosacral[tiab] OR "Lumbo-sacral"[tiab]))) AND (stratif*[tiab] AND ("Risk Assessment"[Mesh] OR delitto[tiab] OR dram[tiab] OR flynn[tiab] OR gatchel[tiab] OR hancock[tiab] OR orebro [tiab] OR "risk analys*"[tiab] OR "risk assessment*"[tiab] OR "risk-benefit assessment"[tiab] OR sullivan[tiab] OR "start back"[tiab] OR Startback[tiab] OR Childs JD[Author] OR Delitto A[Author] OR Flynn T[Author] OR Gatchel RJ[Author] OR Hancock MJ[Author] OR O'Sullivan P[Author] OR O'Sullivan PB[Author] OR ((consequence*[tiab] OR damag*[tiab] OR exposure*[tiab] OR harm*[tiab] OR hazard*[tiab] OR impair*[tiab] OR risk*[tiab] OR threat*[tiab] OR vulnerab*[tiab]) AND (algorithm[tiab] OR analys*[tiab] OR assess*[tiab] OR calculat*[tiab] OR criteria[tiab] OR equation*[tiab] OR index*[tiab] OR instrument*[tiab] OR predict*[tiab] OR questionnaire*[tiab] OR rule*[tiab] OR score*[tiab] OR scoring[tiab] OR screen*[tiab] OR technique*[tiab] OR test*[tiab] OR tool*[tiab])))) AND (2016/1/1:2022/4/29[pdat]) AND (english[Filter]) AND ("Cohort Studies"[Mesh] OR "cohort analys*"[tiab] OR "cohort stud*"[tiab] OR "concurrent stud*"[tiab] OR "incidence stud*"[tiab] OR "Randomized Controlled Trial"[Publication Type] OR placebo[tiab] OR random*[tiab] OR trial*[ti] OR "Meta-Analysis"[Publication Type] OR "Systematic Review"[Publication Type] OR "Systematic Reviews as Topic"[Mesh] OR metaanalys*[ti] OR meta-analys*[ti] OR "meta analys*"[ti] OR "systematic review"[ti] OR trial*[ti] OR ((meta[ti] OR systematic*[ti]) AND (overview*[ti] OR review*[ti] OR synthesis*[ti])))) NOT ("Chromatography"[Mesh] OR "Chromosomes"[Mesh] OR "Genetics"[Mesh] OR "Genome"[Mesh] OR "Genomics"[Mesh] OR "Genotype"[Mesh] OR "Mass Spectrometry"[Mesh] OR "Microbiota"[Mesh] OR "Mutation"[Mesh] OR "Pharmacokinetics"[Mesh] OR "Polymorphism, Genetic"[Mesh] OR "congenital" [Subheading] OR "genetics"[Subheading] OR "pharmacokinetics"[Subheading] OR assay*[tiab] OR cell[tiab] OR cells[tiab] OR chromosome*[tiab] OR genetic*[tiab] OR genome*[tiab] OR genomic*[tiab] OR "in vitro"[tiab] OR microbiom*[tiab] OR microbiota*[tiab] OR mutat*[tiab] OR pharmacokinetic*[tiab] OR polymorphism*[tiab] OR SNP[tiab] OR spectrometry[tiab] OR tissue*[tiab])) NOT (("Animals"[Mesh] OR animal*[tiab] OR ape[tiab] OR apes[tiab] OR canine*[tiab] OR cat[tiab] OR cats[tiab] OR chimpanzee*[tiab] OR dog[tiab] OR dogs[tiab] OR feline*[tiab] OR hamster*[tiab] OR lamb*[tiab] OR mice[tiab] OR monkey*[tiab] OR mouse[tiab] OR murine[tiab] OR pig[tiab] OR pigs[tiab] OR piglet*[tiab] OR porcine[tiab] OR primate*[tiab] OR rabbit*[tiab] OR rat[tiab] OR rats[tiab] OR rodent*[tiab] OR sheep*[tiab] OR swine[tiab]) NOT ("Humans"[Mesh] OR human*[tiab] OR man[tiab] OR men[tiab] OR patient*[tiab] OR woman[tiab] OR women[tiab]))) NOT ("address"[Publication Type] OR "autobiography"[Publication Type] OR "bibliography"[Publication Type] OR "biography"[Publication Type] OR "Book Illustrations"[Publication Type] OR "Case Reports"[Publication Type] OR "Comment"[Publication Type] OR "congress"[Publication Type] OR "consensus development conference"[Publication Type] OR "consensus development conference, nih"[Publication Type] OR "dictionary"[Publication Type] OR "directory"[Publication Type] OR "editorial"[Publication Type] OR "Expression of Concern"[Publication Type] OR "Guideline"[Publication Type] OR "interactive tutorial"[Publication Type] OR "interview"[Publication Type] OR "lecture"[Publication Type] OR "legal case"[Publication Type] OR "legislation"[Publication Type] OR "letter"[Publication Type] OR "news"[Publication Type] OR "newspaper article"[Publication Type] OR "overall"[Publication Type] OR "patient education handout"[Publication Type] OR "periodical index"[Publication Type] OR "personal narrative"[Publication Type] OR "portrait"[Publication Type] OR "Review"[Publication Type] OR "Scientific Integrity Review"[Publication Type] OR "hascommenton"[All Fields] OR

"Cartoons as Topic"[Mesh] OR "Review Literature as Topic"[Mesh] OR "case report*"[tiab] OR "case series"[tiab] OR "integrative research review*"[tiab] OR "integrative review*"[tiab] OR "literature review"[tiab] OR "narrative review"[tiab] OR "research integration"[tiab] OR "scoping review"[tiab] OR ((methodologic*[tiab] OR quantitative*[tiab]) AND (overview*[tiab] OR review*[tiab] OR synthesis*[tiab]))))

Embase: ((((('low back pain'/exp OR 'sciatica'/exp OR (("Low Backache*" OR "Lower Backache*" OR Lumbago OR Sciatic*):ti,ab) OR (('lumbar vertebra'/exp OR 'lumbosacral region'/exp OR (("Low Back" OR "Lower Back" OR Lumbar OR Lumbosacral OR "Lumbo-sacral"):ti,ab)) AND ('pain'/exp OR ((Ache* OR Aching OR Pain*):ti,ab))) OR (('radicular pain'/exp OR 'radiculopathy'/exp OR (("Radicular Pain" OR Radiculitides OR Radiculitis OR Radiculopath*):ti,ab) OR (('nerve root'/exp OR "Nerve Root":ti,ab) AND ('inflammation'/exp OR 'pain'/exp OR ((Ache* OR Aching OR Avulsion* OR Compress* OR Disorder* OR Entrap* OR Imping* OR Inflam* OR Irritat* OR Pinch* OR Trap*):ti,ab)))) AND ('low back pain'/exp OR 'lumbar vertebra'/exp OR 'lumbosacral region'/exp OR ((Low OR Lower OR Lumbar OR Lumbosacral OR "Lumbo-sacral"):ti,ab)))) AND (stratif*:ti,ab AND ('risk assessment'/exp OR ((delitto OR dram OR flynn OR gatchel OR hancock OR Orebro OR "risk analys*" OR "risk assessment*" OR "risk-benefit assessment" OR sullivan OR "start back" OR Startback):ti,ab) OR Childs J D:au OR Delitto A:au OR Flynn T:au OR Gatchel R J:au OR Hancock M J:au OR OSullivan P:au OR OSullivan P B:au OR (((consequence* OR damag* OR exposure* OR harm* OR hazard* OR impair* OR risk* OR threat* OR vulnerab*):ti,ab) AND ((algorithm OR analys* OR assess* OR calculat* OR criteria OR equation* OR index* OR instrument* OR predict* OR questionnaire* OR rule* OR score* OR scoring OR screen* OR technique* OR test* OR tool*):ti,ab)))) AND ([1-1-2016]/sd NOT [30-4-2022]/sd) AND ([english]/lim) AND ('cohort analysis'/exp OR "cohort analys*":ti,ab OR "cohort stud*":ti,ab OR "concurrent stud*":ti,ab OR "incidence stud*":ti,ab OR 'randomized controlled trial'/exp OR placebo:ti,ab OR random*:ti,ab OR trial*:ti OR 'meta analysis'/exp OR 'meta analysis topic'/exp OR 'systematic review'/exp OR 'systematic review (topic)'/exp OR metaanalys*:ti OR meta-analys*:ti OR "meta analys*":ti OR "systematic review":ti OR trial*:ti OR (((meta OR systematic*) AND (overview* OR review* OR synthesis*)):ti))) NOT ('chromatography'/exp OR 'chromosome'/exp OR 'congenital'/exp OR 'genetic polymorphism'/exp OR 'genetics'/exp OR 'genome'/exp OR 'genomics'/exp OR 'genotype'/exp OR 'mass spectrometry'/exp OR 'microbiome'/exp OR 'mutation'/exp OR 'pharmacokinetics'/exp OR 'polymorphism'/exp OR assay*:ti,ab OR cell:ti,ab OR cells:ti,ab OR chromosome*:ti,ab OR genetic*:ti,ab OR genome*:ti,ab OR genomic*:ti,ab OR "in vitro":ti,ab OR microbiom*:ti,ab OR microbiota*:ti,ab OR mutat*:ti,ab OR pharmacokinetic*:ti,ab OR polymorphism*:ti,ab OR SNP:ti,ab OR spectrometry:ti,ab OR tissue*:ti,ab)) NOT (('animal'/exp OR animal*:ti,ab OR ape:ti,ab OR apes:ti,ab OR canine*:ti,ab OR cat:ti,ab OR cats:ti,ab OR chimpanzee*:ti,ab OR dog:ti,ab OR dogs:ti,ab OR feline*:ti,ab OR hamster*:ti,ab OR lamb*:ti,ab OR mice:ti,ab OR monkey*:ti,ab OR mouse:ti,ab OR murine:ti,ab OR pig:ti,ab OR pigs:ti,ab OR piglet*:ti,ab OR porcine:ti,ab OR primate*:ti,ab OR rabbit*:ti,ab OR rat:ti,ab OR rats:ti,ab OR rodent*:ti,ab OR sheep*:ti,ab OR swine:ti,ab) NOT ('human'/exp OR human*:ti,ab OR man:ti,ab OR men:ti,ab OR patient*:ti,ab OR woman:ti,ab OR women:ti,ab))) NOT (('abstract report'/exp OR 'animal experiment'/exp OR 'book'/exp OR 'case finding'/exp OR 'case report'/exp OR 'case study'/exp OR 'conference paper'/exp OR 'editorial'/exp OR 'feasibility study'/exp OR 'in vitro study'/exp) AND 'letter'/exp OR 'note'/exp OR 'practice guideline'/exp OR 'review'/exp OR 'veterinary clinical trial'/exp OR 'veterinary study'/exp OR [conference abstract]/lim OR [conference paper]/lim OR [conference review]/lim OR [editorial]/lim OR [letter]/lim OR [note]/lim OR [short survey]/lim OR 'case report*':ti,ab OR 'case series':ti,ab OR 'integrative research review*':ti,ab OR 'integrative review*':ti,ab OR 'literature review':ti,ab OR 'narrative review':ti,ab OR 'research integration':ti,ab OR 'scoping review':ti,ab OR ((integrative NEAR/5 research NEAR/5 review*):ti,ab) OR ((methodologic* NEAR/5 overview*):ti,ab) OR ((methodologic* NEAR/5 review*):ti,ab) OR ((quantitativ* NEAR/5 overview*):ti,ab) OR ((quantitativ* NEAR/5 review*):ti,ab) OR ((quantitativ* NEAR/5 synthesi*):ti,ab) OR ((research NEAR/5 integration):ti,ab)))

Cochrane:

ID	Search
#1	MeSH descriptor: [Low Back Pain] explode all trees

#2 MeSH descriptor: [Sciatica] explode all trees

#3 (("Low Backache*" OR "Lower Backache*" OR Lumbago OR Sciatic*):ti,ab)

#4 MeSH descriptor: [Lumbar Vertebrae] explode all trees

#5 MeSH descriptor: [Lumbosacral Region] explode all trees

#6 (("Low Back" OR "Lower Back" OR Lumbar OR Lumbosacral OR "Lumbo-sacral"):ti,ab)

#7 OR/#4-#6

#8 MeSH descriptor: [Pain] explode all trees

#9 ((Ache* OR Aching OR Pain*):ti,ab)

#10 #8 OR #9

#11 #7 AND #10

#12 MeSH descriptor: [Radiculopathy] explode all trees

#13 (("Radicular Pain" OR Radiculitides OR Radiculitis OR Radiculopath*):ti,ab)

#14 #12 OR #13

#15 MeSH descriptor: [Spinal Nerve Roots] explode all trees

#16 "Nerve Root":ti,ab

#17 #15 OR #16

#18 MeSH descriptor: [Inflammation] explode all trees

#19 MeSH descriptor: [Pain] explode all trees

#20 ((Ache* OR Aching OR Avulsion* OR Compress* OR Disorder* OR Entrap* OR Imping* OR Inflam* OR Irritat* OR Pinch* OR Trap*):ti,ab)

#21 OR/#18-#20

#22 #14 OR #17 OR #20

#23 MeSH descriptor: [Low Back Pain] explode all trees

#24 MeSH descriptor: [Lumbar Vertebrae] explode all trees

#25 MeSH descriptor: [Lumbosacral Region] explode all trees

#26 ((Low OR Lower OR Lumbar OR Lumbosacral OR "Lumbo-sacral"):ti,ab)

#27 OR/#23-#26

#28 #22 AND #27

#29 #1 OR #2 OR #3 OR #11 OR #28

#30 stratif*:ti,ab

#31 MeSH descriptor: [Risk Assessment] explode all trees

#32 ((delitto OR dram OR flynn OR gatchel OR hancock OR orebro OR "risk analys*" OR "risk assessment*" OR "risk-benefit assessment" OR sullivan OR "start back" OR Startback):ti,ab) OR Childs JD:au OR Delitto A:au OR Flynn T:au OR Gatchel RJ:au OR Hancock MJ:au OR O'Sullivan P:au OR O'Sullivan PB:au OR (((consequence* OR damag* OR exposure* OR harm* OR hazard* OR impair* OR risk* OR threat* OR vulnerab*):ti,ab) AND ((algorithm OR analys* OR assess* OR calculat* OR criteria OR equation* OR index* OR instrument* OR predict* OR questionnaire* OR rule* OR score* OR scoring OR screen* OR technique* OR test* OR tool*):ti,ab))

#33 #31 OR #32

#34 #30 AND #33

#35 #29 AND #34

#36 MeSH descriptor: [Biomarkers] explode all trees

#37 MeSH descriptor: [Chromatography] explode all trees

#38 MeSH descriptor: [Chromosomes] explode all trees

#39 MeSH descriptor: [Echocardiography] explode all trees

#40 MeSH descriptor: [Genetics] in all MeSH products

#41 MeSH descriptor: [Genome] in all MeSH products

#42 MeSH descriptor: [Genotype] explode all trees

#43 MeSH descriptor: [Incidence] explode all trees

#44 MeSH descriptor: [Mass Spectrometry] explode all trees

#45 MeSH descriptor: [Microbiota] explode all trees

#46 MeSH descriptor: [Mutation] explode all trees

#47 MeSH descriptor: [Pharmacokinetics] explode all trees

#48 MeSH descriptor: [Polymorphism, Genetic] explode all trees

#49 Any MeSH descriptor in all MeSH products and with qualifier(s): [congenital - CN]

#50 Any MeSH descriptor in all MeSH products and with qualifier(s): [genetics - GE]

#51 Any MeSH descriptor in all MeSH products and with qualifier(s): [pharmacokinetics - PK]

#52 ((assay* OR biomarker* OR cell OR cells OR chromosome* OR genetic* OR genome* OR genomic* OR "in vitro" OR microbiom* OR microbiota* OR mutat* OR pharmacokinetic* OR polymorphism* OR SNP OR spectrometry OR tissue*):ti,ab)

#53 OR/#36-#52

#54 #35 NOT #53

#55 #54 AND publication date between January 2016 and April 2022

Eligibility criteria:

- Inclusion criteria: ***A) Population:*** People aged 16 or above with non-specific low back pain; People aged 16 or above with sciatica. ***B) Intervention:*** Risk assessment tools + treatment, STarT Back; Risk assessment tools + treatment, DRAM; Risk assessment tools + treatment, ÖREBRO; Risk assessment tools + treatment, Gatchel; Risk assessment tools + treatment, Hicks/Delitto; Risk assessment tools + treatment, Childs/Flynn; Risk assessment tools + treatment, Hancock; Risk assessment tools + treatment, O'Sullivan; Unstratified treatment, treatment without risk tool. ***C) Comparisons***: All interventions will be compared with each other, unless otherwise stated. ***D) Study Type:*** RCT Systematic Review Prospective cohort study Retrospective cohort study.
- Exclusion criteria: None.

PRISMA flow diagram:

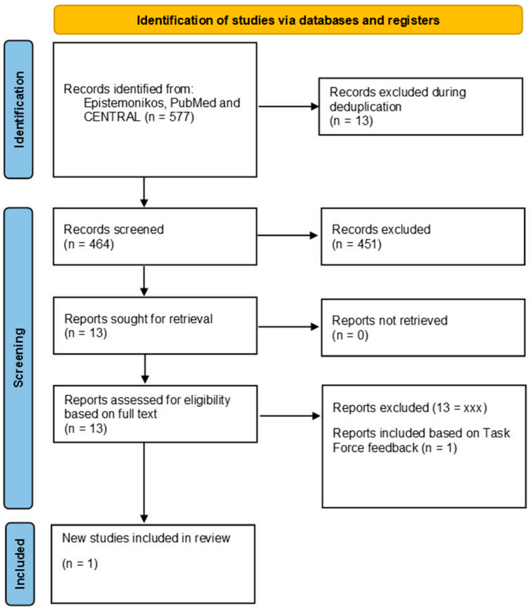

Question 3: Should imaging (plain radiography [X-ray] or magnetic resonance imaging [MRI]) versus no investigation be used to improve functional disability, pain, or psychological distress in patients with LBP and/or sciatica?

Databases searched: PubMed, Embase, and Cochrane

Search period: 01 January 2016 to 04 May 2022 (PubMed and Embase); January 2016 to May 2022 (Cochrane)

Search strategy used:

PubMed: (((("Low Back Pain"[Mesh] OR "Sciatica"[Mesh] OR "Low Backache*"[tiab] OR "Lower Backache*"[tiab] OR Lumbago[tiab] OR Sciatic* [tiab] OR (("Lumbar Vertebrae"[Mesh] OR "Lumbosacral Region"[Mesh] OR "Low Back"[tiab] OR "Lower Back"[tiab] OR Lumbar[tiab] OR Lumbosacral[tiab] OR "Lumbo-sacral"[tiab]) AND ("Pain"[Mesh] OR Ache*[tiab] OR Aching[tiab] OR Pain*[tiab])) OR (("Radiculopathy"[Mesh] OR "Radicular Pain"[tiab] OR Radiculitides[tiab] OR Radiculitis[tiab] OR Radiculopath*[tiab] OR (("Spinal Nerve Roots"[Mesh] OR "Nerve Root"[tiab]) AND ("Inflammation"[Mesh] OR "Pain"[Mesh] OR Ache*[tiab] OR Aching[tiab] OR Avulsion*[tiab] OR Compress*[tiab] OR Disorder*[tiab] OR Entrap*[tiab] OR Imping*[tiab] OR Inflam*[tiab] OR Irritat*[tiab] OR Pinch*[tiab] OR Trap*[tiab]))) AND ("Low Back Pain"[Mesh] OR "Lumbar Vertebrae"[Mesh] OR "Lumbosacral Region"[Mesh] OR Low[tiab] OR Lower[tiab] OR Lumbar[tiab] OR Lumbosacral[tiab] OR "Lumbo-sacral"[tiab]))) AND ("Magnetic Resonance Imaging"[Majr] OR "Radiography"[Majr] OR "radiotherapy"[Subheading] OR "beam tomograph*"[ti] OR "ct imag*"[ti] OR "ct diagnos*"[ti] OR "ct scan*"[ti] OR "cat imag*"[ti] OR "cat diagnos*"[ti] OR "cat scan*"[ti] OR "computed tomograph*"[ti] OR "computer assisted tomograph*"[ti] OR "computerized axial tomograph*"[ti] OR "computerized tomograph*"[ti] OR fmri[ti] OR "magnetic resonance imag*"[ti] OR "magnetic resonance scan*"[ti] OR "magnetic resonance tomogra*"[ti] OR mri[ti] OR neuroimag*[ti] OR roentgenograph*[ti] OR "tomodensitometr*"[ti] OR xray[ti] OR "x-ray"[ti] OR zeugmatography[ti]) AND (2016/1/1:2022/5/4[pdat]) AND (english[Filter]) AND ("Cohort Studies"[Mesh] OR "cohort analys*"[tiab] OR "cohort stud*"[tiab] OR "concurrent stud*"[tiab] OR "incidence stud*"[tiab] OR "Randomized Controlled Trial"[Publication Type] OR placebo[tiab] OR random*[tiab] OR trial*[ti])) NOT ("Chromatography"[Mesh] OR "Chromosomes"[Mesh] OR "Genetics"[Mesh] OR "Genome"[Mesh] OR "Genomics"[Mesh] OR "Genotype"[Mesh] OR "Mass Spectrometry"[Mesh] OR "Microbiota"[Mesh] OR "Mutation"[Mesh] OR "Pharmacokinetics"[Mesh] OR "Polymorphism, Genetic"[Mesh] OR "congenital" [Subheading] OR "genetics"[Subheading] OR "pharmacokinetics"[Subheading] OR assay*[tiab] OR cell[tiab] OR cells[tiab] OR chromosome*[tiab]

OR genetic*[tiab] OR genome*[tiab] OR genomic*[tiab] OR "in vitro"[tiab] OR microbiom*[tiab] OR microbiota*[tiab] OR mutat*[tiab] OR pharmacokinetic*[tiab] OR polymorphism*[tiab] OR SNP[tiab] OR spectrometry[tiab] OR tissue*[tiab])) NOT (("Animals"[Mesh] OR animal*[tiab] OR ape[tiab] OR apes[tiab] OR canine*[tiab] OR cat[tiab] OR cats[tiab] OR chimpanzee*[tiab] OR dog[tiab] OR dogs[tiab] OR feline*[tiab] OR hamster*[tiab] OR lamb*[tiab] OR mice[tiab] OR monkey*[tiab] OR mouse[tiab] OR murine[tiab] OR pig[tiab] OR pigs[tiab] OR piglet*[tiab] OR porcine[tiab] OR primate*[tiab] OR rabbit*[tiab] OR rat[tiab] OR rats[tiab] OR rodent*[tiab] OR sheep*[tiab] OR swine[tiab]) NOT ("Humans"[Mesh] OR human*[tiab] OR man[tiab] OR men[tiab] OR patient*[tiab] OR woman[tiab] OR women[tiab]))) NOT ("address"[Publication Type] OR "autobiography"[Publication Type] OR "bibliography"[Publication Type] OR "biography"[Publication Type] OR "Book Illustrations"[Publication Type] OR "Case Reports"[Publication Type] OR "Comment"[Publication Type] OR "congress"[Publication Type] OR "consensus development conference"[Publication Type] OR "consensus development conference, nih"[Publication Type] OR "dictionary"[Publication Type] OR "directory"[Publication Type] OR "editorial"[Publication Type] OR "Expression of Concern"[Publication Type] OR "Guideline"[Publication Type] OR "interactive tutorial"[Publication Type] OR "interview"[Publication Type] OR "lecture"[Publication Type] OR "legal case"[Publication Type] OR "legislation"[Publication Type] OR "letter"[Publication Type] OR "Meta-Analysis"[Publication Type] OR "news"[Publication Type] OR "newspaper article"[Publication Type] OR "overall"[Publication Type] OR "patient education handout"[Publication Type] OR "periodical index"[Publication Type] OR "personal narrative"[Publication Type] OR "portrait"[Publication Type] OR "Review"[Publication Type] OR "Scientific Integrity Review"[Publication Type] OR "Systematic Review"[Publication Type] OR "hascommenton"[All Fields] OR "Cartoons as Topic"[Mesh] OR "Meta-Analysis as Topic"[Mesh] OR "Review Literature as Topic"[Mesh] OR "Systematic Reviews as Topic"[Mesh] OR "case report*"[tiab] OR "case series"[tiab] OR "integrative research review*"[tiab] OR "integrative review*"[tiab] OR "literature review"[tiab] OR meta-analys*[tiab] OR "meta analys*"[tiab] OR metaanalys*[tiab] OR "narrative review"[tiab] OR "research integration"[tiab] OR "scoping review"[tiab] OR ((methodologic*[tiab] OR quantitative*[tiab] OR systematic*[tiab]) AND (overview*[tiab] OR review*[tiab] OR synthesis*[tiab]))))

Embase: ((((('low back pain'/exp OR 'sciatica'/exp OR (("Low Backache*" OR "Lower Backache*" OR Lumbago OR Sciatic*):ti,ab) OR (('lumbar vertebra'/exp OR 'lumbosacral region'/exp OR (("Low Back" OR "Lower Back" OR Lumbar OR Lumbosacral OR "Lumbo-sacral"):ti,ab)) AND ('pain'/exp OR ((Ache* OR Aching OR Pain*):ti,ab))) OR (('radicular pain'/exp OR 'radiculopathy'/exp OR (("Radicular Pain" OR Radiculitides OR Radiculitis OR Radiculopath*):ti,ab) OR (('nerve root'/exp OR "Nerve Root":ti,ab) AND ('inflammation'/exp OR 'pain'/exp OR ((Ache* OR Aching OR Avulsion* OR Compress* OR Disorder* OR Entrap* OR Imping* OR Inflam* OR Irritat* OR Pinch* OR Trap*):ti,ab)))) AND ('low back pain'/exp OR 'lumbar vertebra'/exp OR 'lumbosacral region'/exp OR ((Low OR Lower OR Lumbar OR Lumbosacral OR "Lumbo-sacral"):ti,ab)))) AND ('radiodiagnosis'/exp/mj OR (("beam tomograph*" OR "ct imag*" OR "ct diagnos*" OR "ct scan*" OR "cat imag*" OR "cat diagnos*" OR "cat scan*" OR "computed tomograph*" OR "computer assisted tomograph*" OR "computerized axial tomograph*" OR "computerized tomograph*" OR fmri OR "magnetic resonance imag*" OR "magnetic resonance scan*" OR "magnetic resonance tomogra*" OR mri OR neuroimag* OR roentgenograph* OR "tomodensitometr*" OR xray OR "x-ray" OR zeugmatography):ti)) AND ([1-1-2016]/sd NOT [5-5-2022]/sd) AND ([english]/lim) AND ('cohort analysis'/exp OR "cohort analys*":ti,ab OR "cohort stud*":ti,ab OR "concurrent stud*":ti,ab OR "incidence stud*":ti,ab OR 'randomized controlled trial'/exp OR placebo:ti,ab OR random*:ti,ab OR trial*:ti)) NOT ('chromatography'/exp OR 'chromosome'/exp OR 'congenital'/exp OR 'genetic polymorphism'/exp OR 'genetics'/exp OR 'genome'/exp OR 'genomics'/exp OR 'genotype'/exp OR 'mass spectrometry'/exp OR 'microbiome'/exp OR 'mutation'/exp OR 'pharmacokinetics'/exp OR 'polymorphism'/exp OR assay*:ti,ab OR cell:ti,ab OR cells:ti,ab OR chromosome*:ti,ab OR genetic*:ti,ab OR genome*:ti,ab OR genomic*:ti,ab OR "in vitro":ti,ab OR microbiom*:ti,ab OR microbiota*:ti,ab OR mutat*:ti,ab OR pharmacokinetic*:ti,ab OR polymorphism*:ti,ab OR SNP:ti,ab OR spectrometry:ti,ab OR tissue*:ti,ab)) NOT (('animal'/exp OR animal*:ti,ab OR ape:ti,ab OR apes:ti,ab OR canine*:ti,ab OR cat:ti,ab OR cats:ti,ab OR chimpanzee*:ti,ab OR dog:ti,ab OR

dogs:ti,ab OR feline*:ti,ab OR hamster*:ti,ab OR lamb*:ti,ab OR mice:ti,ab OR monkey*:ti,ab OR mouse:ti,ab OR murine:ti,ab OR pig:ti,ab OR pigs:ti,ab OR piglet*:ti,ab OR porcine:ti,ab OR primate*:ti,ab OR rabbit*:ti,ab OR rat:ti,ab OR rats:ti,ab OR rodent*:ti,ab OR sheep*:ti,ab OR swine:ti,ab) NOT ('human'/exp OR human*:ti,ab OR man:ti,ab OR men:ti,ab OR patient*:ti,ab OR woman:ti,ab OR women:ti,ab))) NOT (('abstract report'/exp OR 'animal experiment'/exp OR 'book'/exp OR 'case finding'/exp OR 'case report'/exp OR 'case study'/exp OR 'conference paper'/exp OR 'editorial'/exp OR 'feasibility study'/exp OR 'in vitro study'/exp) AND 'letter'/exp OR 'meta analysis'/exp OR 'meta analysis topic'/exp OR 'meta analysis (topic)'/exp OR 'note'/exp OR 'practice guideline'/exp OR 'review'/exp OR 'systematic review'/exp OR 'systematic review topic'/exp OR 'systematic review (topic)'/exp OR 'veterinary clinical trial'/exp OR 'veterinary study'/exp OR [conference abstract]/lim OR [conference paper]/lim OR [conference review]/lim OR [editorial]/lim OR [letter]/lim OR [note]/lim OR [short survey]/lim OR 'case report*':ti,ab OR 'case series':ti,ab OR 'integrative research review*':ti,ab OR 'integrative review*':ti,ab OR 'literature review':ti,ab OR 'meta analys*':ti,ab OR metaanalys*:ti,ab OR meta*analys*:ti,ab OR 'narrative review':ti,ab OR 'research integration':ti,ab OR 'scoping review':ti,ab OR ((integrative NEAR/5 research NEAR/5 review*):ti,ab) OR ((methodologic* NEAR/5 overview*):ti,ab) OR ((methodologic* NEAR/5 review*):ti,ab) OR ((quantitativ* NEAR/5 overview*):ti,ab) OR ((quantitativ* NEAR/5 review*):ti,ab) OR ((quantitativ* NEAR/5 synthesi*):ti,ab) OR ((research NEAR/5 integration):ti,ab) OR ((systematic* NEAR/5 overview*):ti,ab) OR ((systematic* NEAR/5 review*):ti,ab)))

Cochrane:

**ID**	**Search**
#1	MeSH descriptor: [Low Back Pain] explode all trees
#2	MeSH descriptor: [Sciatica] explode all trees
#3	(("Low Backache*" OR "Lower Backache*" OR Lumbago OR Sciatic*):ti,ab)
#4	MeSH descriptor: [Lumbar Vertebrae] explode all trees
#5	MeSH descriptor: [Lumbosacral Region] explode all trees
#6	(("Low Back" OR "Lower Back" OR Lumbar OR Lumbosacral OR "Lumbo-sacral"):ti,ab)
#7	OR/#4-#6
#8	MeSH descriptor: [Pain] explode all trees
#9	((Ache* OR Aching OR Pain*):ti,ab)
#10	#8 OR #9
#11	#7 AND #10
#12	MeSH descriptor: [Radiculopathy] explode all trees
#13	(("Radicular Pain" OR Radiculitides OR Radiculitis OR Radiculopath*):ti,ab)
#14	#12 OR #13
#15	MeSH descriptor: [Spinal Nerve Roots] explode all trees
#16	"Nerve Root":ti,ab
#17	#15 OR #16
#18	MeSH descriptor: [Inflammation] explode all trees
#19	MeSH descriptor: [Pain] explode all trees
#20	((Ache* OR Aching OR Avulsion* OR Compress* OR Disorder* OR Entrap* OR Imping* OR Inflam* OR Irritat* OR Pinch* OR Trap*):ti,ab)
#21	OR/#18-#20

#22 #14 OR #17 OR #20

#23 MeSH descriptor: [Low Back Pain] explode all trees

#24 MeSH descriptor: [Lumbar Vertebrae] explode all trees

#25 MeSH descriptor: [Lumbosacral Region] explode all trees

#26 ((Low OR Lower OR Lumbar OR Lumbosacral OR "Lumbo-sacral"):ti,ab)

#27 OR/#23-#26

#28 #22 AND #27

#29 #1 OR #2 OR #3 OR #11 OR #28

#30 MeSH descriptor: [Magnetic Resonance Imaging] explode all trees

#31 MeSH descriptor: [Radiography] explode all trees

#32 (("beam tomograph*" OR "ct imag*" OR "ct diagnos*" OR "ct scan*" OR "cat imag*" OR "cat diagnos*" OR "cat scan*" OR "computed tomograph*" OR "computer assisted tomograph*" OR "computerized axial tomograph*" OR "computerized tomograph*" OR fmri OR "magnetic resonance imag*" OR "magnetic resonance scan*" OR "magnetic resonance tomogra*" OR mri OR neuroimag* OR roentgenograph* OR "tomodensitometr*" OR xray OR "x-ray" OR zeugmatography):ti)

#33 OR/#30-#32

#34 #29 AND #33

#35 MeSH descriptor: [Biomarkers] explode all trees

#36 MeSH descriptor: [Chromatography] explode all trees

#37 MeSH descriptor: [Chromosomes] explode all trees

#38 MeSH descriptor: [Echocardiography] explode all trees

#39 MeSH descriptor: [Genetics] in all MeSH products

#40 MeSH descriptor: [Genome] in all MeSH products

#41 MeSH descriptor: [Genotype] explode all trees

#42 MeSH descriptor: [Incidence] explode all trees

#43 MeSH descriptor: [Mass Spectrometry] explode all trees

#44 MeSH descriptor: [Microbiota] explode all trees

#45 MeSH descriptor: [Mutation] explode all trees

#46 MeSH descriptor: [Pharmacokinetics] explode all trees

#47 MeSH descriptor: [Polymorphism, Genetic] explode all trees

#48 Any MeSH descriptor in all MeSH products and with qualifier(s): [congenital - CN]

#49 Any MeSH descriptor in all MeSH products and with qualifier(s): [genetics - GE]

#50 Any MeSH descriptor in all MeSH products and with qualifier(s): [pharmacokinetics - PK]

#51 ((assay* OR biomarker* OR cell OR cells OR chromosome* OR genetic* OR genome* OR genomic* OR "in vitro" OR microbiom* OR microbiota* OR mutat* OR pharmacokinetic* OR polymorphism* OR SNP OR spectrometry OR tissue*):ti,ab)

#52 OR/#35-#51

#53 #34 NOT #52

#54 #53 AND publication date between January 2016 and May 2022, in Trials

Eligibility criteria:

- Inclusion criteria: ***A) Population:*** People aged 16 or above with non-specific low back pain with or without sciatica; people aged 16 or above with sciatica. ***B) Intervention:*** Imaging for low back pain, MRI, CT or X-ray; Imaging for sciatica, MRI; No imaging; Deferred imaging. ***C)* Comparison:** All interventions will be compared with each other, unless otherwise stated. ***D) Study Type:*** Randomized controlled trials, prospective cohort studies, and retrospective cohort studies.
- Exclusion criteria: None.

PRISMA flow diagram:

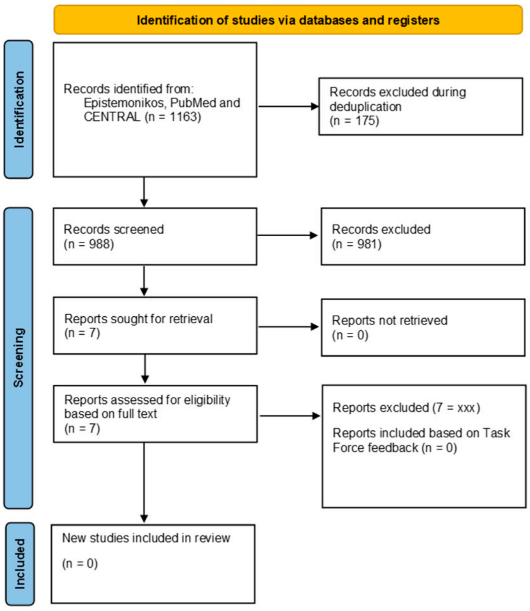

Question 4: Should pharmacological treatment versus placebo or usual care/waiting list, or one or more pharmacological interventions compared with each other, be used in patients with sciatica?

Databases searched: PubMed, Embase, and Cochrane

Search period: 01 September 2020 to 28 May 2022 (PubMed and Embase); September 2020 to May 2022 (Cochrane)

Search strategy used:

PubMed: ((((("Sciatica"[Mesh] OR Sciatic*[tiab] OR (("Radiculopathy"[Mesh] OR "Radicular Pain"[tiab] OR Radiculitides[tiab] OR Radiculitis[tiab] OR Radiculopath*[tiab] OR (("Spinal Nerve Roots"[Mesh] OR "Nerve Root"[tiab]) AND ("Inflammation"[Mesh] OR "Pain"[Mesh] OR Ache*[tiab] OR Aching[tiab] OR Avulsion*[tiab] OR Compress*[tiab] OR Disorder*[tiab] OR Entrap*[tiab] OR Imping*[tiab] OR Inflam*[tiab] OR Irritat*[tiab] OR Pinch*[tiab] OR Trap*[tiab]))) AND ("Low Back Pain"[Mesh] OR "Lumbar Vertebrae"[Mesh] OR "Lumbosacral Region"[Mesh] OR Low[tiab] OR Lower[tiab] OR Lumbar[tiab] OR Lumbosacral[tiab] OR "Lumbo-sacral"[tiab]))) AND ("Analgesics"[Mesh] OR "Anti-Bacterial Agents"[Mesh] OR "Anticonvulsants"[Mesh] OR "Antidepressive Agents"[Mesh] OR "Anti-Inflammatory Agents, Non-Steroidal"[Mesh] OR "Drug Therapy"[Mesh] OR "Muscle Relaxants, Central"[Mesh] OR "Serotonin and Noradrenaline Reuptake Inhibitors"[Mesh] OR "Serotonin Uptake Inhibitors"[Mesh] OR "Vitamin D"[Mesh] OR "Analgesics"[Pharmacological Action] OR "Analgesics, Opioid"[Pharmacological Action] OR "Analgesics, Opioid"[Pharmacological Action] OR "Anti-Bacterial Agents"[Pharmacological Action] OR "Anticonvulsants"[Pharmacological Action] OR "Antidepressive Agents"[Pharmacological

Action] OR "Antidepressive Agents, Second-Generation"[Pharmacological Action] OR "Antidepressive Agents, Tricyclic"[Pharmacological Action] OR "Anti-Inflammatory Agents, Non-Steroidal"[Pharmacological Action] OR "Cyclooxygenase 2 Inhibitors"[Pharmacological Action] OR "Muscle Relaxants, Central"[Pharmacological Action] OR "Serotonin and Noradrenaline Reuptake Inhibitors"[Pharmacological Action] OR "Serotonin Uptake Inhibitors"[Pharmacological Action] OR "drug therapy"[Subheading] OR aceclofenac[tiab] OR acetaminophen[tiab] OR acetylsalicyl*[tiab] OR alclofenac[tiab] OR amitriptyline[tiab] OR amoxapine[tiab] OR analgesic*[tiab] OR antibacterial[tiab] OR anti-bacterial[tiab] OR antibiotic*[tiab] OR anticonvulsant*[tiab] OR antidepress*[tiab] OR anti-depress*[tiab] OR antimicrobial*[tiab] OR anti-microbial*[tiab] OR antimycobacterial*[tiab] OR anti-mycobacterial*[tiab] OR baclofen[tiab] OR bactericid*[tiab] OR bacteriocid*[tiab] OR bupropion[tiab] OR carbamazepine[tiab] OR "carbasalate calcium"[tiab] OR carisoprodol[tiab] OR celecoxib[tiab] OR chlorzoxazone[tiab] OR citalopram[tiab] OR clomipramine[tiab] OR codeine[tiab] OR cyclobenzaprine[tiab] OR "cyclooxygenase 2 inhibitor*"[tiab] OR dantrolene[tiab] OR desipramine[tiab] OR dexibuprofen[tiab] OR diazepam[tiab] OR diclofenac[tiab] OR diflunisal[tiab] OR "drug therap*"[tiab] OR "drug treatment*"[tiab] OR dothiepin[tiab] OR doxepin[tiab] OR etoricoxib[tiab] OR fenoprofen[tiab] OR fentanyl[tiab] OR fluoxetine[tiab] OR flupirtine[tiab] OR flurbiprofen[tiab] OR fluvoxamine[tiab] OR gabapentin*[tiab] OR hydrocodone[tiab] OR hydromorphone[tiab] OR ibuprofen[tiab] OR imipramine[tiab] OR indometacin[tiab] OR iprindole[tiab] OR ketoprofen[tiab] OR levorphanol[tiab] OR lofepramine[tiab] OR maprotiline[tiab] OR meloxicam[tiab] OR meperidine[tiab] OR meprobramate[tiab] OR metamizol[tiab] OR metaxalone[tiab] OR methocarbamol[tiab] OR mianserin[tiab] OR morphine[tiab] OR "muscle relax*"[tiab] OR nabumeton[tiab] OR naproxen[tiab] OR "non-steroidal anti-inflammatory agent*"[tiab] OR "non-steroidal anti-inflammatory drug*"[tiab] OR "noradrenaline reuptake inhibitor*"[tiab] OR nortriptyline[tiab] OR nsaid*[tiab] OR opipramol[tiab] OR orphenadrine[tiab] OR oxycodone[tiab] OR oxymorphone[tiab] OR paracetamol[tiab] OR parecoxib[tiab] OR paroxetine[tiab] OR pentazocine[tiab] OR pharmacotherap*[tiab] OR phenazone[tiab] OR phenylbutazone[tiab] OR phenytoin[tiab] OR piroxicam[tiab] OR pregabalin[tiab] OR propoxyphene[tiab] OR propyphenazone[tiab] OR protriptyline[tiab] OR quipazine[tiab] OR ritanserin[tiab] OR "serotonin and noradrenaline reuptake inhibitor*"[tiab] OR "serotonin reuptake inhibitor*"[tiab] OR snri*[tiab] OR ssri*[tiab] OR sufentanil[tiab] OR sulindac[tiab] OR sulpiride[tiab] OR tapentadol[tiab] OR tetrazepam[tiab] OR tiapro*[tiab] OR tizanidine[tiab] OR topiramate[tiab] OR tramadol[tiab] OR trazodone[tiab] OR trimipramine[tiab] OR tryptophan[tiab] OR viloxazine[tiab] OR "vitamin d"[tiab] OR ((antiinflamm*[tiab] OR "anti-inflamm*"[tiab]) AND (nonsteroid*[tiab] OR "non- steroid*"[tiab])) OR ((cox2[tiab] OR "cox-2"[tiab] OR coxii[tiab] OR "cox-ii"[tiab]) AND inhibitor*[tiab]) OR ((cyclooxygenaseii[tiab] OR "cyclooxygenase-ii"[tiab] OR "cyclo-oxygenaseii"[tiab] OR "cyclo-oxygenase-ii"[tiab]) AND inhibitor*[tiab])) AND (2016/1/1:2022/5/8[pdat]) AND (english[Filter]) AND ("Randomized Controlled Trial"[Publication Type] OR placebo[tiab] OR random*[tiab] OR trial*[ti] OR "Meta-Analysis"[Publication Type] OR "Systematic Review"[Publication Type] OR "Systematic Reviews as Topic"[Mesh] OR metaanalys*[ti] OR meta-analys*[ti] OR "meta analys*"[ti] OR "systematic review"[ti] OR trial*[ti] OR ((meta[ti] OR systematic*[ti]) AND (overview*[ti] OR review*[ti] OR synthesis*[ti])))) NOT ("Chromatography"[Mesh] OR "Chromosomes"[Mesh] OR "Genetics"[Mesh] OR "Genome"[Mesh] OR "Genomics"[Mesh] OR "Genotype"[Mesh] OR "Mass Spectrometry"[Mesh] OR "Microbiota"[Mesh] OR "Mutation"[Mesh] OR "Pharmacokinetics"[Mesh] OR "Polymorphism, Genetic"[Mesh] OR "congenital" [Subheading] OR "genetics"[Subheading] OR "pharmacokinetics"[Subheading] OR assay*[tiab] OR cell[tiab] OR cells[tiab] OR chromosome*[tiab] OR genetic*[tiab] OR genome*[tiab] OR genomic*[tiab] OR "in vitro"[tiab] OR microbiom*[tiab] OR microbiota*[tiab] OR mutat*[tiab] OR pharmacokinetic*[tiab] OR polymorphism*[tiab] OR SNP[tiab] OR spectrometry[tiab] OR tissue*[tiab]))) NOT (("Animals"[Mesh] OR animal*[tiab] OR ape[tiab] OR apes[tiab] OR canine*[tiab] OR cat[tiab] OR cats[tiab] OR chimpanzee*[tiab] OR dog[tiab] OR dogs[tiab] OR feline*[tiab] OR hamster*[tiab] OR lamb*[tiab] OR mice[tiab] OR monkey*[tiab] OR mouse[tiab] OR murine[tiab] OR pig[tiab] OR pigs[tiab] OR piglet*[tiab] OR porcine[tiab] OR primate*[tiab] OR rabbit*[tiab] OR rat[tiab] OR rats[tiab] OR rodent*[tiab] OR sheep*[tiab] OR swine[tiab]) NOT ("Humans"[Mesh] OR human*[tiab] OR man[tiab] OR men[tiab] OR patient*[tiab] OR woman[tiab] OR women[tiab]))) NOT ("address"[Publication Type] OR "autobiography"[Publication Type] OR "bibliography"[Publication Type] OR "biography"[Publication

Type] OR "Book Illustrations"[Publication Type] OR "Case Reports"[Publication Type] OR "Comment"[Publication Type] OR "congress"[Publication Type] OR "consensus development conference"[Publication Type] OR "consensus development conference, nih"[Publication Type] OR "dictionary"[Publication Type] OR "directory"[Publication Type] OR "editorial"[Publication Type] OR "Expression of Concern"[Publication Type] OR "Guideline"[Publication Type] OR "interactive tutorial"[Publication Type] OR "interview"[Publication Type] OR "lecture"[Publication Type] OR "legal case"[Publication Type] OR "legislation"[Publication Type] OR "letter"[Publication Type] OR "news"[Publication Type] OR "newspaper article"[Publication Type] OR "overall"[Publication Type] OR "patient education handout"[Publication Type] OR "periodical index"[Publication Type] OR "personal narrative"[Publication Type] OR "portrait"[Publication Type] OR "Review"[Publication Type] OR "Scientific Integrity Review"[Publication Type] OR "hascommenton"[All Fields] OR "Cartoons as Topic"[Mesh] OR "Review Literature as Topic"[Mesh] OR "case report*"[tiab] OR "case series"[tiab] OR "integrative research review*"[tiab] OR "integrative review*"[tiab] OR "literature review"[tiab] OR "narrative review"[tiab] OR "research integration"[tiab] OR "scoping review"[tiab] OR ((methodologic*[tiab] OR quantitative*[tiab]) AND (overview*[tiab] OR review*[tiab] OR synthesis*[tiab]))))

Embase: ((((('sciatica'/exp OR sciatic:ti,ab OR (('radicular pain'/exp OR 'radiculopathy'/exp OR (("Radicular Pain" OR Radiculitides OR Radiculitis OR Radiculopath*):ti,ab) OR (('nerve root'/exp OR "Nerve Root":ti,ab) AND ('inflammation'/exp OR 'pain'/exp OR ((Ache* OR Aching OR Avulsion* OR Compress* OR Disorder* OR Entrap* OR Imping* OR Inflam* OR Irritat* OR Pinch* OR Trap*):ti,ab)))) AND ('low back pain'/exp OR 'lumbar vertebra'/exp OR 'lumbosacral region'/exp OR ((Low OR Lower OR Lumbar OR Lumbosacral OR "Lumbo-sacral"):ti,ab)))) AND ('analgesic agent'/exp OR 'anticonvulsive agent'/exp OR 'antidepressant agent'/exp OR 'antiinfective agent'/exp OR 'cyclooxygenase 2 inhibitor'/exp OR 'gabapentinoid'/exp OR 'muscle relaxant agent'/exp OR 'muscle relaxants'/exp OR 'nonsteroid antiinflammatory agent'/exp OR 'serotonin uptake inhibitor'/exp OR 'vitamin D'/exp OR 'drug therapy'/lnk OR ((aceclofenac OR acetaminophen OR acetylsalicyl* OR alclofenac OR amitriptyline OR amoxapine OR analgesic* OR antibacterial OR anti-bacterial OR antibiotic* OR anticonvulsant* OR antidepress* OR anti-depress* OR antimicrobial* OR anti-microbial* OR antimycobacterial* OR anti-mycobacterial* OR baclofen OR bactericid* OR bacteriocid* OR bupropion OR carbamazepine OR "carbasalate calcium" OR carisoprodol OR celecoxib OR chlorzoxazone OR citalopram OR clomipramine OR codeine OR cyclobenzaprine OR "cyclooxygenase 2 inhibitor*" OR dantrolene OR desipramine OR dexibuprofen OR diazepam OR diclofenac OR diflunisal OR "drug therap*" OR "drug treatment*" OR dothiepin OR doxepin OR etoricoxib OR fenoprofen OR fentanyl OR fluoxetine OR flupirtine OR flurbiprofen OR fluvoxamine OR gabapentin* OR hydrocodone OR hydromorphone OR ibuprofen OR imipramine OR indometacin OR iprindole OR ketoprofen OR levorphanol OR lofepramine OR maprotiline OR meloxicam OR meperidine OR meprobramate OR metamizol OR metaxalone OR methocarbamol OR mianserin OR morphine OR "muscle relax*" OR nabumeton OR naproxen OR "non-steroidal anti-inflammatory agent*" OR "non-steroidal anti-inflammatory drug*" OR "noradrenaline reuptake inhibitor*" OR nortriptyline OR nsaid* OR opipramol OR orphenadrine OR oxycodone OR oxymorphone OR paracetamol OR parecoxib OR paroxetine OR pentazocine OR pharmacotherap* OR phenazone OR phenylbutazone OR phenytoin OR piroxicam OR pregabalin OR propoxyphene OR propyphenazone OR protriptyline OR quipazine OR ritanserin OR "serotonin and noradrenaline reuptake inhibitor*" OR "serotonin reuptake inhibitor*" OR snri* OR ssri* OR sufentanil OR sulindac OR sulpiride OR tapentadol OR tetrazepam OR tiapro* OR tizanidine OR topiramate OR tramadol OR trazodone OR trimipramine OR tryptophan OR viloxazine OR "vitamin d"):ti,ab) OR (((antiinflamm* OR "anti-inflamm*") AND (nonsteroid* OR "non- steroid*")):ti,ab) OR (((cox2 OR "cox-2" OR coxii OR cox-ii) AND inhibitor*):ti,ab) OR (((cyclooxygenaseii OR "cyclooxygenase-ii" OR "cyclo-oxygenaseii" OR "cyclo-oxygenase-ii") AND inhibitor*):ti,ab)) AND ([1-1-2016]/sd NOT [9-5-2022]/sd) AND ([english]/lim) AND ('randomized controlled trial'/exp OR placebo:ti,ab OR random*:ti,ab OR trial*:ti OR 'meta analysis'/exp OR 'meta analysis topic'/exp OR 'systematic review'/exp OR 'systematic review (topic)'/exp OR metaanalys*:ti OR meta-analys*:ti OR "meta analys*":ti OR "systematic review":ti OR trial*:ti OR (((meta OR systematic*) AND (overview* OR review* OR synthesis*)):ti))) NOT ('chromatography'/exp OR 'chromosome'/exp OR 'congenital'/exp OR 'genetic polymorphism'/exp OR

'genetics'/exp OR 'genome'/exp OR 'genomics'/exp OR 'genotype'/exp OR 'mass spectrometry'/exp OR 'microbiome'/exp OR 'mutation'/exp OR 'pharmacokinetics'/exp OR 'polymorphism'/exp OR assay*:ti,ab OR cell:ti,ab OR cells:ti,ab OR chromosome*:ti,ab OR genetic*:ti,ab OR genome*:ti,ab OR genomic*:ti,ab OR "in vitro":ti,ab OR microbiom*:ti,ab OR microbiota*:ti,ab OR mutat*:ti,ab OR pharmacokinetic*:ti,ab OR polymorphism*:ti,ab OR SNP:ti,ab OR spectrometry:ti,ab OR tissue*:ti,ab)) NOT (('animal'/exp OR animal*:ti,ab OR ape:ti,ab OR apes:ti,ab OR canine*:ti,ab OR cat:ti,ab OR cats:ti,ab OR chimpanzee*:ti,ab OR dog:ti,ab OR dogs:ti,ab OR feline*:ti,ab OR hamster*:ti,ab OR lamb*:ti,ab OR mice:ti,ab OR monkey*:ti,ab OR mouse:ti,ab OR murine:ti,ab OR pig:ti,ab OR pigs:ti,ab OR piglet*:ti,ab OR porcine:ti,ab OR primate*:ti,ab OR rabbit*:ti,ab OR rat:ti,ab OR rats:ti,ab OR rodent*:ti,ab OR sheep*:ti,ab OR swine:ti,ab) NOT ('human'/exp OR human*:ti,ab OR man:ti,ab OR men:ti,ab OR patient*:ti,ab OR woman:ti,ab OR women:ti,ab))) NOT (('abstract report'/exp OR 'animal experiment'/exp OR 'book'/exp OR 'case finding'/exp OR 'case report'/exp OR 'case study'/exp OR 'conference paper'/exp OR 'editorial'/exp OR 'feasibility study'/exp OR 'in vitro study'/exp) AND 'letter'/exp OR 'note'/exp OR 'practice guideline'/exp OR 'review'/exp OR 'veterinary clinical trial'/exp OR 'veterinary study'/exp OR [conference abstract]/lim OR [conference paper]/lim OR [conference review]/lim OR [editorial]/lim OR [letter]/lim OR [note]/lim OR [short survey]/lim OR 'case report*':ti,ab OR 'case series':ti,ab OR 'integrative research review*':ti,ab OR 'integrative review*':ti,ab OR 'literature review':ti,ab OR 'narrative review':ti,ab OR 'research integration':ti,ab OR 'scoping review':ti,ab OR ((integrative NEAR/5 research NEAR/5 review*):ti,ab) OR ((methodologic* NEAR/5 overview*):ti,ab) OR ((methodologic* NEAR/5 review*):ti,ab) OR ((quantitativ* NEAR/5 overview*):ti,ab) OR ((quantitativ* NEAR/5 review*):ti,ab) OR ((quantitativ* NEAR/5 synthesi*):ti,ab) OR ((research NEAR/5 integration):ti,ab)))

Cochrane:

**ID**	**Search**
#1	MeSH descriptor: [Sciatica] explode all trees
#2	Sciatic*:ti,ab
#3	MeSH descriptor: [Radiculopathy] explode all trees
#4	(("Radicular Pain" OR Radiculitides OR Radiculitis OR Radiculopath*):ti,ab)
#5	#3 OR #4
#6	MeSH descriptor: [Spinal Nerve Roots] explode all trees
#7	"Nerve Root":ti,ab
#8	#6 OR #7
#9	MeSH descriptor: [Inflammation] explode all trees
#10	MeSH descriptor: [Pain] explode all trees
#11	((Ache* OR Aching OR Avulsion* OR Compress* OR Disorder* OR Entrap* OR Imping* OR Inflam* OR Irritat* OR Pinch* OR Trap*):ti,ab)
#12	OR/#9-#11
#13	#5 OR #8 OR #12
#14	(MeSH descriptor: [Low Back Pain] explode all trees
#15	MeSH descriptor: [Lumbar Vertebrae] explode all trees
#16	MeSH descriptor: [Lumbosacral Region] explode all trees
#17	((Low OR Lower OR Lumbar OR Lumbosacral OR "Lumbo-sacral"):ti,ab)
#18	OR/#14-#17
#19	#13 AND #18

#20 #1 OR #2 OR #19

#21 MeSH descriptor: [Analgesics] explode all trees

#22 MeSH descriptor: [Anti-Bacterial Agents] explode all trees

#23 MeSH descriptor: [Anticonvulsants] explode all trees

#24 MeSH descriptor: [Antidepressive Agents] explode all trees

#25 MeSH descriptor: [Anti-Inflammatory Agents, Non-Steroidal] explode all trees

#26 MeSH descriptor: [Drug Therapy] explode all trees

#27 MeSH descriptor: [Muscle Relaxants, Central] explode all trees

#28 MeSH descriptor: [Serotonin and Noradrenaline Reuptake Inhibitors] explode all trees

#29 MeSH descriptor: [Serotonin Uptake Inhibitors] explode all trees

#30 MeSH descriptor: [Vitamin D] explode all trees

#31 ((aceclofenac OR acetaminophen OR acetylsalicyl* OR alclofenac OR amitriptyline OR amoxapine OR analgesic* OR antibacterial OR anti-bacterial OR antibiotic* OR anticonvulsant* OR antidepress* OR anti-depress* OR antimicrobial* OR anti-microbial* OR antimycobacterial* OR anti-mycobacterial* OR baclofen OR bactericid* OR bacteriocid* OR bupropion OR carbamazepine OR "carbasalate calcium" OR carisoprodol OR celecoxib OR chlorzoxazone OR citalopram OR clomipramine OR codeine OR cyclobenzaprine OR "cyclooxygenase 2 inhibitor*" OR dantrolene OR desipramine OR dexibuprofen OR diazepam OR diclofenac OR diflunisal OR "drug therap*" OR "drug treatment*" OR dothiepin OR doxepin OR etoricoxib OR fenoprofen OR fentanyl OR fluoxetine OR flupirtine OR flurbiprofen OR fluvoxamine OR gabapentin* OR hydrocodone OR hydromorphone OR ibuprofen OR imipramine OR indometacin OR iprindole OR ketoprofen OR levorphanol OR lofepramine OR maprotiline OR meloxicam OR meperidine OR meprobramate OR metamizol OR metaxalone OR methocarbamol OR mianserin OR morphine OR "muscle relax*" OR nabumeton OR naproxen OR "non-steroidal anti-inflammatory agent*" OR "non-steroidal anti-inflammatory drug*" OR "noradrenaline reuptake inhibitor*" OR nortriptyline OR nsaid* OR opipramol OR orphenadrine OR oxycodone OR oxymorphone OR paracetamol OR parecoxib OR paroxetine OR pentazocine OR pharmacotherap* OR phenazone OR phenylbutazone OR phenytoin OR piroxicam OR pregabalin OR propoxyphene OR propyphenazone OR protriptyline OR quipazine OR ritanserin OR "serotonin and noradrenaline reuptake inhibitor*" OR "serotonin reuptake inhibitor*" OR snri* OR ssri* OR sufentanil OR sulindac OR sulpiride OR tapentadol OR tetrazepam OR tiapro* OR tizanidine OR topiramate OR tramadol OR trazodone OR trimipramine OR tryptophan OR viloxazine OR "vitamin d"):ti,ab) OR (((antiinflamm* OR "anti-inflamm*") AND (nonsteroid* OR "non- steroid*")):ti,ab) OR (((cox2 OR "cox-2" OR coxii OR cox-ii) AND inhibitor*):ti,ab) OR (((cyclooxygenaseii OR "cyclooxygenase-ii" OR "cyclo-oxygenaseii" OR "cyclo-oxygenase-ii") AND inhibitor*):ti,ab)

#32 OR/#21-#31

#33 #20 AND #32

#34 MeSH descriptor: [Biomarkers] explode all trees

#35 MeSH descriptor: [Chromatography] explode all trees

#36 MeSH descriptor: [Chromosomes] explode all trees

#37 MeSH descriptor: [Echocardiography] explode all trees

#38 MeSH descriptor: [Genetics] in all MeSH products

#39 MeSH descriptor: [Genome] in all MeSH products

#40 MeSH descriptor: [Genotype] explode all trees

#41 MeSH descriptor: [Incidence] explode all trees

#42 MeSH descriptor: [Mass Spectrometry] explode all trees

#43 MeSH descriptor: [Microbiota] explode all trees

#44 MeSH descriptor: [Mutation] explode all trees

#45 MeSH descriptor: [Pharmacokinetics] explode all trees

#46 MeSH descriptor: [Polymorphism, Genetic] explode all trees

#47 Any MeSH descriptor in all MeSH products and with qualifier(s): [congenital - CN]

#48 Any MeSH descriptor in all MeSH products and with qualifier(s): [genetics - GE]

#49 Any MeSH descriptor in all MeSH products and with qualifier(s): [pharmacokinetics - PK]

#50 ((assay* OR biomarker* OR cell OR cells OR chromosome* OR genetic* OR genome* OR genomic* OR "in vitro" OR microbiom* OR microbiota* OR mutat* OR pharmacokinetic* OR polymorphism* OR SNP OR spectrometry OR tissue*):ti,ab)

#51 OR/#34-#50

#52 #33 NOT #51

#53 #52 AND publication date between January 2016 and May 2022

Eligibility criteria:

- Inclusion criteria: ***A) Population:*** People aged 16 or above with non-specific low back pain with or without sciatica; people aged 16 or above with sciatica. ***B) Intervention:*** Pharmacological treatment (oral/sublingual, rectal, intra-muscular and transdermal but not intravenous): Paracetamol, Non-steroidal anti-inflammatory drugs, opioid analgesics, muscle relaxants, antidepressants (SSRIs, SNRIs, tri-cyclic antidepressants), Anticonvulsants (gabapentinoids, other anticonvulsants), antibiotics, Vitamin D; any other non-invasive intervention included in the guideline; combination of interventions: any combination of the non-invasive interventions; placebo/sham/attention control; or usual care/waiting-list. ***C) Comparison:*** All interventions will be compared with each other, unless otherwise stated. ***D) Study Type:*** Randomized controlled trials, systematic reviews, and non-randomized studies.
- Exclusion criteria: None.

PRISMA flow diagram:

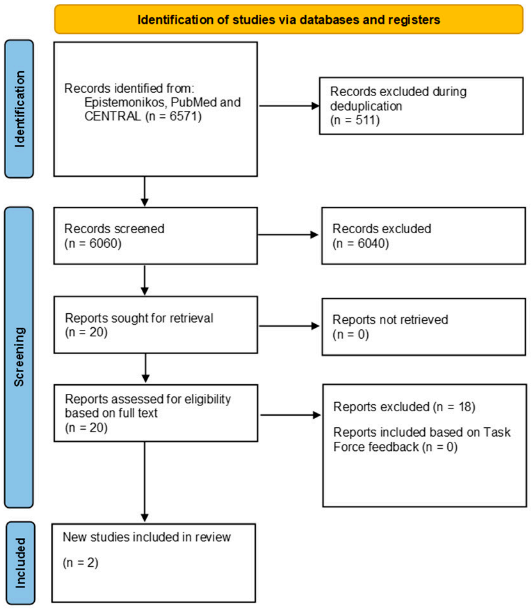

Question 5: Should interventions or multidisciplinary programmes with a specified return to work focus (or including ergonomic interventions) versus placebo, usual care or waiting list, or versus one or more interventions compared with each other or other non-invasive interventions (either alone or in combination) be used in patients with non-specific LBP and/or sciatica?

Databases searched: PubMed, Embase, and Cochrane

Search period: 01 January 2016 to 10 May 2022 (PubMed and Embase); January 2016 to May 2022 (Cochrane)

Search strategy used:

PubMed: ((((("Low Back Pain"[Mesh] OR "Sciatica"[Mesh] OR "Low Backache*"[tiab] OR "Lower Backache*"[tiab] OR Lumbago[tiab] OR Sciatic* [tiab] OR (("Lumbar Vertebrae"[Mesh] OR "Lumbosacral Region"[Mesh] OR "Low Back"[tiab] OR "Lower Back"[tiab] OR Lumbar[tiab] OR Lumbosacral[tiab] OR "Lumbo-sacral"[tiab]) AND ("Pain"[Mesh] OR Ache*[tiab] OR Aching[tiab] OR Pain*[tiab])) OR (("Radiculopathy"[Mesh] OR "Radicular Pain"[tiab] OR Radiculitides[tiab] OR Radiculitis[tiab] OR Radiculopath*[tiab] OR (("Spinal Nerve Roots"[Mesh] OR "Nerve Root"[tiab]) AND ("Inflammation"[Mesh] OR "Pain"[Mesh] OR Ache*[tiab] OR Aching[tiab] OR Avulsion*[tiab] OR Compress*[tiab] OR Disorder*[tiab] OR Entrap*[tiab] OR Imping*[tiab] OR Inflam*[tiab] OR Irritat*[tiab] OR Pinch*[tiab] OR Trap*[tiab]))) AND ("Low Back Pain"[Mesh] OR "Lumbar Vertebrae"[Mesh] OR "Lumbosacral Region"[Mesh] OR Low[tiab] OR Lower[tiab] OR Lumbar[tiab] OR Lumbosacral[tiab] OR "Lumbo-sacral"[tiab]))) AND ("Return to Work"[Mesh] OR "Back to Work"[tiab] OR "Return to Work"[tiab] OR (("Workplace"[Mesh] OR work*[tiab]) AND (exercise*[tiab] OR intervention*[tiab]OR modification*[tiab] OR modify[tiab] OR program*[tiab] OR therap*[tiab] OR train*[tiab] OR treat*[tiab]))) AND (2016/1/1:2022/5/10[pdat]) AND (english[Filter]) AND ("Randomized Controlled Trial"[Publication Type] OR placebo[tiab] OR random*[tiab] OR trial*[ti] OR "Meta-Analysis"[Publication Type] OR "Systematic Review"[Publication Type] OR "Systematic Reviews as Topic"[Mesh] OR metaanalys*[ti] OR meta-analys*[ti] OR "meta analys*"[ti] OR "systematic review"[ti] OR trial*[ti] OR ((meta[ti] OR systematic*[ti]) AND (overview*[ti] OR review*[ti] OR synthesis*[ti])))) NOT ("Chromatography"[Mesh] OR "Chromosomes"[Mesh] OR "Genetics"[Mesh] OR "Genome"[Mesh] OR "Genomics"[Mesh] OR "Genotype"[Mesh] OR "Mass Spectrometry"[Mesh] OR "Microbiota"[Mesh] OR "Mutation"[Mesh] OR "Pharmacokinetics"[Mesh] OR "Polymorphism, Genetic"[Mesh] OR "congenital" [Subheading] OR "genetics"[Subheading] OR "pharmacokinetics"[Subheading] OR assay*[tiab] OR cell[tiab] OR cells[tiab] OR chromosome*[tiab] OR genetic*[tiab] OR genome*[tiab] OR genomic*[tiab] OR "in vitro"[tiab] OR microbiom*[tiab] OR microbiota*[tiab] OR mutat*[tiab] OR pharmacokinetic*[tiab] OR polymorphism*[tiab] OR SNP[tiab] OR spectrometry[tiab] OR tissue*[tiab])) NOT (("Animals"[Mesh] OR animal*[tiab] OR ape[tiab] OR apes[tiab] OR canine*[tiab] OR cat[tiab] OR cats[tiab] OR chimpanzee*[tiab] OR dog[tiab] OR dogs[tiab] OR feline*[tiab] OR hamster*[tiab] OR lamb*[tiab] OR mice[tiab] OR monkey*[tiab] OR mouse[tiab] OR murine[tiab] OR pig[tiab] OR pigs[tiab] OR piglet*[tiab] OR porcine[tiab] OR primate*[tiab] OR rabbit*[tiab] OR rat[tiab] OR rats[tiab] OR rodent*[tiab] OR sheep*[tiab] OR swine[tiab]) NOT ("Humans"[Mesh] OR human*[tiab] OR man[tiab] OR men[tiab] OR patient*[tiab] OR woman[tiab] OR women[tiab]))) NOT ("address"[Publication Type] OR "autobiography"[Publication Type] OR "bibliography"[Publication Type] OR "biography"[Publication Type] OR "Book Illustrations"[Publication Type] OR "Case Reports"[Publication Type] OR "Comment"[Publication Type] OR "congress"[Publication Type] OR "consensus development conference"[Publication Type] OR "consensus development conference, nih"[Publication Type] OR "dictionary"[Publication Type] OR "directory"[Publication Type] OR "editorial"[Publication Type] OR "Expression of Concern"[Publication Type] OR "Guideline"[Publication Type] OR "interactive tutorial"[Publication Type] OR "interview"[Publication Type] OR "lecture"[Publication Type] OR "legal case"[Publication Type] OR "legislation"[Publication Type] OR "letter"[Publication Type] OR "news"[Publication Type] OR "newspaper article"[Publication Type] OR "overall"[Publication Type] OR "patient education handout"[Publication Type] OR "periodical index"[Publication Type] OR

"personal narrative"[Publication Type] OR "portrait"[Publication Type] OR "Review"[Publication Type] OR "Scientific Integrity Review"[Publication Type] OR "hascommenton"[All Fields] OR "Cartoons as Topic"[Mesh] OR "Review Literature as Topic"[Mesh] OR "case report*"[tiab] OR "case series"[tiab] OR "integrative research review*"[tiab] OR "integrative review*"[tiab] OR "literature review"[tiab] OR "narrative review"[tiab] OR "research integration"[tiab] OR "scoping review"[tiab] OR ((methodologic*[tiab] OR quantitative*[tiab]) AND (overview*[tiab] OR review*[tiab] OR synthesis*[tiab]))))

Embase: (((((('low back pain'/exp OR 'sciatica'/exp OR (("Low Backache*" OR "Lower Backache*" OR Lumbago OR Sciatic*):ti,ab) OR (('lumbar vertebra'/exp OR 'lumbosacral region'/exp OR (("Low Back" OR "Lower Back" OR Lumbar OR Lumbosacral OR "Lumbo-sacral"):ti,ab)) AND ('pain'/exp OR ((Ache* OR Aching OR Pain*):ti,ab))) OR (('radicular pain'/exp OR 'radiculopathy'/exp OR (("Radicular Pain" OR Radiculitides OR Radiculitis OR Radiculopath*):ti,ab) OR (('nerve root'/exp OR "Nerve Root":ti,ab) AND ('inflammation'/exp OR 'pain'/exp OR ((Ache* OR Aching OR Avulsion* OR Compress* OR Disorder* OR Entrap* OR Imping* OR Inflam* OR Irritat* OR Pinch* OR Trap*):ti,ab)))) AND ('low back pain'/exp OR 'lumbar vertebra'/exp OR 'lumbosacral region'/exp OR ((Low OR Lower OR Lumbar OR Lumbosacral OR "Lumbo-sacral"):ti,ab)))) AND ('return to work'/exp OR "Back to Work":ti,ab OR "Return to Work":ti,ab OR (('workplace'/exp OR work*:ti,ab) AND ((exercise* OR intervention*OR modification* OR modify OR program* OR therap* OR train* OR treat*):ti,ab))) AND ([1-1-2016]/sd NOT [11-5-2022]/sd) AND ([english]/lim) AND ('randomized controlled trial'/exp OR placebo:ti,ab OR random*:ti,ab OR trial*:ti OR 'meta analysis'/exp OR 'meta analysis topic'/exp OR 'systematic review'/exp OR 'systematic review (topic)'/exp OR metaanalys*:ti OR meta-analys*:ti OR "meta analys*":ti OR "systematic review":ti OR trial*:ti OR (((meta OR systematic*) AND (overview* OR review* OR synthesis*)):ti))) NOT ('chromatography'/exp OR 'chromosome'/exp OR 'congenital'/exp OR 'genetic polymorphism'/exp OR 'genetics'/exp OR 'genome'/exp OR 'genomics'/exp OR 'genotype'/exp OR 'mass spectrometry'/exp OR 'microbiome'/exp OR 'mutation'/exp OR 'pharmacokinetics'/exp OR 'polymorphism'/exp OR assay*:ti,ab OR cell:ti,ab OR cells:ti,ab OR chromosome*:ti,ab OR genetic*:ti,ab OR genome*:ti,ab OR genomic*:ti,ab OR "in vitro":ti,ab OR microbiom*:ti,ab OR microbiota*:ti,ab OR mutat*:ti,ab OR pharmacokinetic*:ti,ab OR polymorphism*:ti,ab OR SNP:ti,ab OR spectrometry:ti,ab OR tissue*:ti,ab)) NOT (('animal'/exp OR animal*:ti,ab OR ape:ti,ab OR apes:ti,ab OR canine*:ti,ab OR cat:ti,ab OR cats:ti,ab OR chimpanzee*:ti,ab OR dog:ti,ab OR dogs:ti,ab OR feline*:ti,ab OR hamster*:ti,ab OR lamb*:ti,ab OR mice:ti,ab OR monkey*:ti,ab OR mouse:ti,ab OR murine:ti,ab OR pig:ti,ab OR pigs:ti,ab OR piglet*:ti,ab OR porcine:ti,ab OR primate*:ti,ab OR rabbit*:ti,ab OR rat:ti,ab OR rats:ti,ab OR rodent*:ti,ab OR sheep*:ti,ab OR swine:ti,ab) NOT ('human'/exp OR human*:ti,ab OR man:ti,ab OR men:ti,ab OR patient*:ti,ab OR woman:ti,ab OR women:ti,ab))) NOT (('abstract report'/exp OR 'animal experiment'/exp OR 'book'/exp OR 'case finding'/exp OR 'case report'/exp OR 'case study'/exp OR 'conference paper'/exp OR 'editorial'/exp OR 'feasibility study'/exp OR 'in vitro study'/exp) AND 'letter'/exp OR 'note'/exp OR 'practice guideline'/exp OR 'review'/exp OR 'veterinary clinical trial'/exp OR 'veterinary study'/exp OR [conference abstract]/lim OR [conference paper]/lim OR [conference review]/lim OR [editorial]/lim OR [letter]/lim OR [note]/lim OR [short survey]/lim OR 'case report*':ti,ab OR 'case series':ti,ab OR 'integrative research review*':ti,ab OR 'integrative review*':ti,ab OR 'literature review':ti,ab OR 'narrative review':ti,ab OR 'research integration':ti,ab OR 'scoping review':ti,ab OR ((integrative NEAR/5 research NEAR/5 review*):ti,ab) OR ((methodologic* NEAR/5 overview*):ti,ab) OR ((methodologic* NEAR/5 review*):ti,ab) OR ((quantitativ* NEAR/5 overview*):ti,ab) OR ((quantitativ* NEAR/5 review*):ti,ab) OR ((quantitativ* NEAR/5 synthesi*):ti,ab) OR ((research NEAR/5 integration):ti,ab)))

Cochrane:

**ID**	**Search**
#1	MeSH descriptor: [Low Back Pain] explode all trees
#2	MeSH descriptor: [Sciatica] explode all trees
#3	(("Low Backache*" OR "Lower Backache*" OR Lumbago OR Sciatic*):ti,ab)

#4 MeSH descriptor: [Lumbar Vertebrae] explode all trees

#5 MeSH descriptor: [Lumbosacral Region] explode all trees

#6 (("Low Back" OR "Lower Back" OR Lumbar OR Lumbosacral OR "Lumbo-sacral"):ti,ab)

#7 OR/#4-#6

#8 MeSH descriptor: [Pain] explode all trees

#9 ((Ache* OR Aching OR Pain*):ti,ab)

#10 #8 OR #9

#11 #7 AND #10

#12 MeSH descriptor: [Radiculopathy] explode all trees

#13 (("Radicular Pain" OR Radiculitides OR Radiculitis OR Radiculopath*):ti,ab)

#14 #12 OR #13

#15 MeSH descriptor: [Spinal Nerve Roots] explode all trees

#16 "Nerve Root":ti,ab

#17 #15 OR #16

#18 MeSH descriptor: [Inflammation] explode all trees

#19 MeSH descriptor: [Pain] explode all trees

#20 ((Ache* OR Aching OR Avulsion* OR Compress* OR Disorder* OR Entrap* OR Imping* OR Inflam* OR Irritat* OR Pinch* OR Trap*):ti,ab)

#21 OR/#18-#20

#22 #14 OR #17 OR #20

#23 MeSH descriptor: [Low Back Pain] explode all trees

#24 MeSH descriptor: [Lumbar Vertebrae] explode all trees

#25 MeSH descriptor: [Lumbosacral Region] explode all trees

#26 ((Low OR Lower OR Lumbar OR Lumbosacral OR "Lumbo-sacral"):ti,ab)

#27 OR/#23-#26

#28 #22 AND #27

#29 #1 OR #2 OR #3 OR #11 OR #28

#30 MeSH descriptor: [Return to Work] explode all trees

#31 "Back to Work":ti,ab OR "Return to Work":ti,ab

#32 MeSH descriptor: [Workplace] explode all trees

#33 work*:ti,ab

#34 #32 AND #33

#35 ((exercise* OR intervention*OR modification* OR modify OR program* OR therap* OR train* OR treat*):ti,ab)

#36 #34 AND #35

#37 #30 OR #31 OR #36

#38 #29 AND #37

#39 MeSH descriptor: [Biomarkers] explode all trees

#40 MeSH descriptor: [Chromatography] explode all trees

#41 MeSH descriptor: [Chromosomes] explode all trees

#42 MeSH descriptor: [Echocardiography] explode all trees

#43 MeSH descriptor: [Genetics] in all MeSH products

#44 MeSH descriptor: [Genome] in all MeSH products

#45 MeSH descriptor: [Genotype] explode all trees

#46 MeSH descriptor: [Incidence] explode all trees

#47 MeSH descriptor: [Mass Spectrometry] explode all trees

#48 MeSH descriptor: [Microbiota] explode all trees

#49 MeSH descriptor: [Mutation] explode all trees

#50 MeSH descriptor: [Pharmacokinetics] explode all trees

#51 MeSH descriptor: [Polymorphism, Genetic] explode all trees

#52 Any MeSH descriptor in all MeSH products and with qualifier(s): [congenital - CN]

#53 Any MeSH descriptor in all MeSH products and with qualifier(s): [genetics - GE]

#54 Any MeSH descriptor in all MeSH products and with qualifier(s): [pharmacokinetics - PK]

#55 ((assay* OR biomarker* OR cell OR cells OR chromosome* OR genetic* OR genome* OR genomic* OR "in vitro" OR microbiom* OR microbiota* OR mutat* OR pharmacokinetic* OR polymorphism* OR SNP OR spectrometry OR tissue*):ti,ab)

#56 OR/#39-#55

#57 #38 NOT #56

#58 #57 AND publication date between January 2016 to May 2022, in Trials

Eligibility criteria:

- Inclusion criteria: ***A) Population:*** People aged 16 or above with non-specific low back pain with or without sciatica; People aged 16 or above with sciatica. ***B) Intervention:*** Combinations of interventions: Any combination of the non-invasive interventions (Exercise interventions, Postural therapies, Manual therapies, Electrotherapy, Orthotics and appliances, Acupuncture, Self-management strategies, Psychological interventions, Pharmacological treatment (oral/sublingual, rectal, intra-muscular and transdermal but not intravenous)); Uni-disciplinary programmes including combined concepts: where it is one profession (usually Physio) who may be using cognitive - behavioural principles or a cognitive - behavioural approach, alongside exercise / education; Multidisciplinary biopsychosocial programmes. Multidisciplinary defined as: 'multidisciplinary biopsychosocial programmes that target factors from the different domains (physical, psychological and social), delivered by clinicians from at least two different professional backgrounds'; Interventions/multidisciplinary programmes with a specified return to work focus (or including ergonomic interventions); Any other non-invasive intervention included in the guideline; Placebo/Sham/Attention control; and usual care/waiting list. ***C) Comparison:*** All interventions will be compared with each other. ***D) Study Type:*** Randomized controlled trials, systematic reviews, and non-randomized studies.
- Exclusion criteria: None.

PRISMA flow diagram:

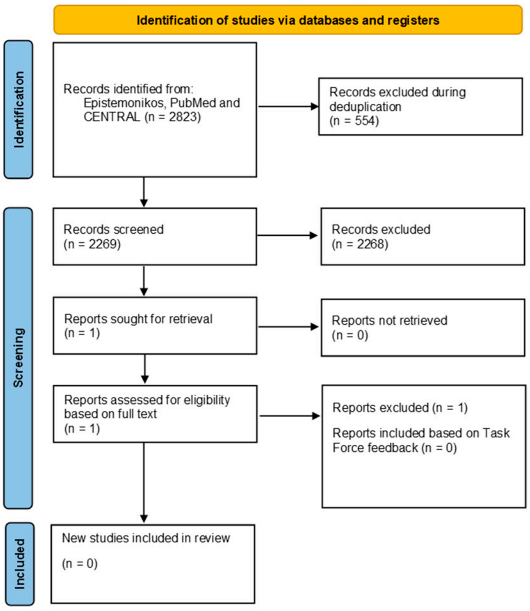

Question 6: Should psychological interventions (behavioural therapies, cognitive therapies, cognitive–behavioural approaches, mindfulness, and acceptance and commitment therapy) versus placebo or usual care/waiting list, or versus one or more interventions compared with each other or other non-invasive interventions (either alone or in combination), be used in patients with non-specific LBP and/or sciatica?

Databases searched: PubMed, Embase, and Cochrane

Search period: 01 January 2016 to 18 May 2022 (PubMed and Embase); January 2016 to May 2022 (Cochrane)

Search strategy used:

PubMed: ((((("Low Back Pain"[Mesh] OR "Sciatica"[Mesh] OR "Low Backache*"[tiab] OR "Lower Backache*"[tiab] OR Lumbago[tiab] OR Sciatic* [tiab] OR (("Lumbar Vertebrae"[Mesh] OR "Lumbosacral Region"[Mesh] OR "Low Back"[tiab] OR "Lower Back"[tiab] OR Lumbar[tiab] OR Lumbosacral[tiab] OR "Lumbo-sacral"[tiab]) AND ("Pain"[Mesh] OR Ache*[tiab] OR Aching[tiab] OR Pain*[tiab])) OR (("Radiculopathy"[Mesh] OR "Radicular Pain"[tiab] OR Radiculitides[tiab] OR Radiculitis[tiab] OR Radiculopath*[tiab] OR (("Spinal Nerve Roots"[Mesh] OR "Nerve Root"[tiab]) AND ("Inflammation"[Mesh] OR "Pain"[Mesh] OR Ache*[tiab] OR Aching[tiab] OR Avulsion*[tiab] OR Compress*[tiab] OR Disorder*[tiab] OR Entrap*[tiab] OR Imping*[tiab] OR Inflam*[tiab] OR Irritat*[tiab] OR Pinch*[tiab] OR Trap*[tiab]))) AND ("Low Back Pain"[Mesh] OR "Lumbar Vertebrae"[Mesh] OR "Lumbosacral Region"[Mesh] OR Low[tiab] OR Lower[tiab] OR Lumbar[tiab] OR Lumbosacral[tiab] OR "Lumbo-sacral"[tiab]))) AND ("Behavior Therapy"[Mesh:NoExp] OR "Cognitive Behavioral Therapy"[Mesh] OR "Acceptance and Commitment Therap*"[tiab] OR "Acceptance Therap*"[tiab] OR "Acceptance-based Therap*"[tiab] OR "Behavior Change Technique*"[tiab] OR "Behavior Modif*"[tiab] OR "Behavior Psychotherap*"[tiab] OR "Behavior Therap*"[tiab] OR "Behavior Treatment*"[tiab] OR "Behavioral Change Technique*"[tiab] OR "Behavioral Modif*"[tiab] OR "Behavioral Psychotherap*"[tiab] OR "Behavioral Therap*"[tiab] OR "Behavioral Treatment*"[tiab] OR "Behaviour Change Technique*"[tiab] OR "Behaviour Modif*"[tiab] OR "Behaviour Psychotherap*"[tiab] OR "Behaviour Therap*"[tiab] OR "Behaviour Treatment*"[tiab] OR "Behavioural Change Technique*"[tiab] OR "Behavioural Modif*"[tiab] OR "Behavioural Psychotherap*"[tiab] OR "Behavioural Therap*"[tiab] OR "Behavioural Treatment*"[tiab] OR "Cognition Therap*"[tiab] OR "Cognitive Psychotherap*"[tiab] OR "Cognitive Therap*"[tiab] OR "Commitment Therap*"[tiab] OR "Conditioning Therap*"[tiab] OR Mindful*[tiab] OR "Psychological Therap*"[tiab] OR "Psychological Treatment*"[tiab] OR "Psychological

Treatment*"[tiab] OR Psychotherap*[tiab]) AND (2016/1/1:2022/5/17[pdat]) AND (english[Filter]) AND ("Randomized Controlled Trial"[Publication Type] OR placebo[tiab] OR random*[tiab] OR trial*[ti] OR "Meta-Analysis"[Publication Type] OR "Systematic Review"[Publication Type] OR "Systematic Reviews as Topic"[Mesh] OR metaanalys*[ti] OR meta-analys*[ti] OR "meta analys*"[ti] OR "systematic review"[ti] OR trial*[ti] OR ((meta[ti] OR systematic*[ti]) AND (overview*[ti] OR review*[ti] OR synthesis*[ti])))) NOT ("Chromatography"[Mesh] OR "Chromosomes"[Mesh] OR "Genetics"[Mesh] OR "Genome"[Mesh] OR "Genomics"[Mesh] OR "Genotype"[Mesh] OR "Mass Spectrometry"[Mesh] OR "Microbiota"[Mesh] OR "Mutation"[Mesh] OR "Pharmacokinetics"[Mesh] OR "Polymorphism, Genetic"[Mesh] OR "congenital" [Subheading] OR "genetics"[Subheading] OR "pharmacokinetics"[Subheading] OR assay*[tiab] OR cell[tiab] OR cells[tiab] OR chromosome*[tiab] OR genetic*[tiab] OR genome*[tiab] OR genomic*[tiab] OR "in vitro"[tiab] OR microbiom*[tiab] OR microbiota*[tiab] OR mutat*[tiab] OR pharmacokinetic*[tiab] OR polymorphism*[tiab] OR SNP[tiab] OR spectrometry[tiab] OR tissue*[tiab])) NOT (("Animals"[Mesh] OR animal*[tiab] OR ape[tiab] OR apes[tiab] OR canine*[tiab] OR cat[tiab] OR cats[tiab] OR chimpanzee*[tiab] OR dog[tiab] OR dogs[tiab] OR feline*[tiab] OR hamster*[tiab] OR lamb*[tiab] OR mice[tiab] OR monkey*[tiab] OR mouse[tiab] OR murine[tiab] OR pig[tiab] OR pigs[tiab] OR piglet*[tiab] OR porcine[tiab] OR primate*[tiab] OR rabbit*[tiab] OR rat[tiab] OR rats[tiab] OR rodent*[tiab] OR sheep*[tiab] OR swine[tiab]) NOT ("Humans"[Mesh] OR human*[tiab] OR man[tiab] OR men[tiab] OR patient*[tiab] OR woman[tiab] OR women[tiab]))) NOT ("address"[Publication Type] OR "autobiography"[Publication Type] OR "bibliography"[Publication Type] OR "biography"[Publication Type] OR "Book Illustrations"[Publication Type] OR "Case Reports"[Publication Type] OR "Comment"[Publication Type] OR "congress"[Publication Type] OR "consensus development conference"[Publication Type] OR "consensus development conference, nih"[Publication Type] OR "dictionary"[Publication Type] OR "directory"[Publication Type] OR "editorial"[Publication Type] OR "Expression of Concern"[Publication Type] OR "Guideline"[Publication Type] OR "interactive tutorial"[Publication Type] OR "interview"[Publication Type] OR "lecture"[Publication Type] OR "legal case"[Publication Type] OR "legislation"[Publication Type] OR "letter"[Publication Type] OR "news"[Publication Type] OR "newspaper article"[Publication Type] OR "overall"[Publication Type] OR "patient education handout"[Publication Type] OR "periodical index"[Publication Type] OR "personal narrative"[Publication Type] OR "portrait"[Publication Type] OR "Review"[Publication Type] OR "Scientific Integrity Review"[Publication Type] OR "hascommenton"[All Fields] OR "Cartoons as Topic"[Mesh] OR "Review Literature as Topic"[Mesh] OR "case report*"[tiab] OR "case series"[tiab] OR "integrative research review*"[tiab] OR "integrative review*"[tiab] OR "literature review"[tiab] OR "narrative review"[tiab] OR "research integration"[tiab] OR "scoping review"[tiab] OR ((methodologic*[tiab] OR quantitative*[tiab]) AND (overview*[tiab] OR review*[tiab] OR synthesis*[tiab]))))

Embase: ((((('low back pain'/exp OR 'sciatica'/exp OR (("Low Backache*" OR "Lower Backache*" OR Lumbago OR Sciatic*):ti,ab) OR (('lumbar vertebra'/exp OR 'lumbosacral region'/exp OR (("Low Back" OR "Lower Back" OR Lumbar OR Lumbosacral OR "Lumbo-sacral"):ti,ab)) AND ('pain'/exp OR ((Ache* OR Aching OR Pain*):ti,ab))) OR (('radicular pain'/exp OR 'radiculopathy'/exp OR (("Radicular Pain" OR Radiculitides OR Radiculitis OR Radiculopath*):ti,ab) OR (('nerve root'/exp OR "Nerve Root":ti,ab) AND ('inflammation'/exp OR 'pain'/exp OR ((Ache* OR Aching OR Avulsion* OR Compress* OR Disorder* OR Entrap* OR Imping* OR Inflam* OR Irritat* OR Pinch* OR Trap*):ti,ab)))) AND ('low back pain'/exp OR 'lumbar vertebra'/exp OR 'lumbosacral region'/exp OR ((Low OR Lower OR Lumbar OR Lumbosacral OR "Lumbo-sacral"):ti,ab)))) AND ('behavior therapy'/de OR 'cognitive behavioral therapy'/exp OR 'mindfulness'/exp OR (("Acceptance and Commitment Therap*" OR "Acceptance Therap*" OR "Acceptance-based Therap*" OR "Behavior Change Technique*" OR "Behavior Modif*" OR "Behavior Psychotherap*" OR "Behavior Therap*" OR "Behavior Treatment*" OR "Behavioral Change Technique*" OR "Behavioral Modif*" OR "Behavioral Psychotherap*" OR "Behavioral Therap*" OR "Behavioral Treatment*" OR "Behaviour Change Technique*" OR "Behaviour Modif*" OR "Behaviour Psychotherap*" OR "Behaviour Therap*" OR "Behaviour Treatment*" OR "Behavioural Change Technique*" OR "Behavioural Modif*" OR "Behavioural Psychotherap*" OR "Behavioural Therap*" OR "Behavioural Treatment*" OR "Cognition Therap*" OR "Cognitive Psychotherap*" OR "Cognitive Therap*" OR "Commitment

Therap*" OR "Conditioning Therap*" OR Mindful* OR "Psychological Therap*" OR "Psychological Treatment*" OR "Psychological Treatment*" OR Psychotherap*):ti,ab)) AND ([1-1-2016]/sd NOT [18-5-2022]/sd) AND ([english]/lim) AND ('randomized controlled trial'/exp OR placebo:ti,ab OR random*:ti,ab OR trial*:ti OR 'meta analysis'/exp OR 'meta analysis topic'/exp OR 'systematic review'/exp OR 'systematic review (topic)'/exp OR metaanalys*:ti OR meta-analys*:ti OR "meta analys*":ti OR "systematic review":ti OR trial*:ti OR (((meta OR systematic*) AND (overview* OR review* OR synthesis*)):ti))) NOT ('chromatography'/exp OR 'chromosome'/exp OR 'congenital'/exp OR 'genetic polymorphism'/exp OR 'genetics'/exp OR 'genome'/exp OR 'genomics'/exp OR 'genotype'/exp OR 'mass spectrometry'/exp OR 'microbiome'/exp OR 'mutation'/exp OR 'pharmacokinetics'/exp OR 'polymorphism'/exp OR assay*:ti,ab OR cell:ti,ab OR cells:ti,ab OR chromosome*:ti,ab OR genetic*:ti,ab OR genome*:ti,ab OR genomic*:ti,ab OR "in vitro":ti,ab OR microbiom*:ti,ab OR microbiota*:ti,ab OR mutat*:ti,ab OR pharmacokinetic*:ti,ab OR polymorphism*:ti,ab OR SNP:ti,ab OR spectrometry:ti,ab OR tissue*:ti,ab)) NOT (('animal'/exp OR animal*:ti,ab OR ape:ti,ab OR apes:ti,ab OR canine*:ti,ab OR cat:ti,ab OR cats:ti,ab OR chimpanzee*:ti,ab OR dog:ti,ab OR dogs:ti,ab OR feline*:ti,ab OR hamster*:ti,ab OR lamb*:ti,ab OR mice:ti,ab OR monkey*:ti,ab OR mouse:ti,ab OR murine:ti,ab OR pig:ti,ab OR pigs:ti,ab OR piglet*:ti,ab OR porcine:ti,ab OR primate*:ti,ab OR rabbit*:ti,ab OR rat:ti,ab OR rats:ti,ab OR rodent*:ti,ab OR sheep*:ti,ab OR swine:ti,ab) NOT ('human'/exp OR human*:ti,ab OR man:ti,ab OR men:ti,ab OR patient*:ti,ab OR woman:ti,ab OR women:ti,ab))) NOT (('abstract report'/exp OR 'animal experiment'/exp OR 'book'/exp OR 'case finding'/exp OR 'case report'/exp OR 'case study'/exp OR 'conference paper'/exp OR 'editorial'/exp OR 'feasibility study'/exp OR 'in vitro study'/exp) AND 'letter'/exp OR 'note'/exp OR 'practice guideline'/exp OR 'review'/exp OR 'veterinary clinical trial'/exp OR 'veterinary study'/exp OR [conference abstract]/lim OR [conference paper]/lim OR [conference review]/lim OR [editorial]/lim OR [letter]/lim OR [note]/lim OR [short survey]/lim OR 'case report*':ti,ab OR 'case series':ti,ab OR 'integrative research review*':ti,ab OR 'integrative review*':ti,ab OR 'literature review':ti,ab OR 'narrative review':ti,ab OR 'research integration':ti,ab OR 'scoping review':ti,ab OR ((integrative NEAR/5 research NEAR/5 review*):ti,ab) OR ((methodologic* NEAR/5 overview*):ti,ab) OR ((methodologic* NEAR/5 review*):ti,ab) OR ((quantitativ* NEAR/5 overview*):ti,ab) OR ((quantitativ* NEAR/5 review*):ti,ab) OR ((quantitativ* NEAR/5 synthesi*):ti,ab) OR ((research NEAR/5 integration):ti,ab)))

Cochrane:

**ID**	**Search**
#1	MeSH descriptor: [Low Back Pain] explode all trees
#2	MeSH descriptor: [Sciatica] explode all trees
#3	(("Low Backache*" OR "Lower Backache*" OR Lumbago OR Sciatic*):ti,ab)
#4	MeSH descriptor: [Lumbar Vertebrae] explode all trees
#5	MeSH descriptor: [Lumbosacral Region] explode all trees
#6	(("Low Back" OR "Lower Back" OR Lumbar OR Lumbosacral OR "Lumbo-sacral"):ti,ab)
#7	OR/#4-#6
#8	MeSH descriptor: [Pain] explode all trees
#9	((Ache* OR Aching OR Pain*):ti,ab)
#10	#8 OR #9
#11	#7 AND #10
#12	MeSH descriptor: [Radiculopathy] explode all trees
#13	(("Radicular Pain" OR Radiculitides OR Radiculitis OR Radiculopath*):ti,ab)
#14	#12 OR #13
#15	MeSH descriptor: [Spinal Nerve Roots] explode all trees

#16 "Nerve Root":ti,ab

#17 #15 OR #16

#18 MeSH descriptor: [Inflammation] explode all trees

#19 MeSH descriptor: [Pain] explode all trees

#20 ((Ache* OR Aching OR Avulsion* OR Compress* OR Disorder* OR Entrap* OR Imping* OR Inflam* OR Irritat* OR Pinch* OR Trap*):ti,ab)

#21 OR/#18-#20

#22 #14 OR #17 OR #20

#23 MeSH descriptor: [Low Back Pain] explode all trees

#24 MeSH descriptor: [Lumbar Vertebrae] explode all trees

#25 MeSH descriptor: [Lumbosacral Region] explode all trees

#26 ((Low OR Lower OR Lumbar OR Lumbosacral OR "Lumbo-sacral"):ti,ab)

#27 OR/#23-#26

#28 #22 AND #27

#29 #1 OR #2 OR #3 OR #11 OR #28

#30 MeSH descriptor: [Behavior Therapy] this term only

#31 MeSH descriptor: [Cognitive Behavioral Therapy] explode all trees

#32 (("Acceptance and Commitment Therap*" OR "Acceptance Therap*" OR "Acceptance-based Therap*" OR "Behavior Change Technique*" OR "Behavior Modif*" OR "Behavior Psychotherap*" OR "Behavior Therap*" OR "Behavior Treatment*" OR "Behavioral Change Technique*" OR "Behavioral Modif*" OR "Behavioral Psychotherap*" OR "Behavioral Therap*" OR "Behavioral Treatment*" OR "Behaviour Change Technique*" OR "Behaviour Modif*" OR "Behaviour Psychotherap*" OR "Behaviour Therap*" OR "Behaviour Treatment*" OR "Behavioural Change Technique*" OR "Behavioural Modif*" OR "Behavioural Psychotherap*" OR "Behavioural Therap*" OR "Behavioural Treatment*" OR "Cognition Therap*" OR "Cognitive Psychotherap*" OR "Cognitive Therap*" OR "Commitment Therap*" OR "Conditioning Therap*" OR Mindful* OR "Psychological Therap*" OR "Psychological Treatment*" OR "Psychological Treatment*" OR Psychotherap*):ti,ab)

#33 OR/#30-#32

#34 #29 AND #33

#35 MeSH descriptor: [Biomarkers] explode all trees

#36 MeSH descriptor: [Chromatography] explode all trees

#37 MeSH descriptor: [Chromosomes] explode all trees

#38 MeSH descriptor: [Echocardiography] explode all trees

#39 MeSH descriptor: [Genetics] in all MeSH products

#40 MeSH descriptor: [Genome] in all MeSH products

#41 MeSH descriptor: [Genotype] explode all trees

#42 MeSH descriptor: [Incidence] explode all trees

#43 MeSH descriptor: [Mass Spectrometry] explode all trees

#44 MeSH descriptor: [Microbiota] explode all trees

#45 MeSH descriptor: [Mutation] explode all trees

#46	MeSH descriptor: [Pharmacokinetics] explode all trees

#47	MeSH descriptor: [Polymorphism, Genetic] explode all trees

#48	Any MeSH descriptor in all MeSH products and with qualifier(s): [congenital - CN]

#49	Any MeSH descriptor in all MeSH products and with qualifier(s): [genetics - GE]

#50	Any MeSH descriptor in all MeSH products and with qualifier(s): [pharmacokinetics - PK]

#51	((assay* OR biomarker* OR cell OR cells OR chromosome* OR genetic* OR genome* OR genomic* OR "in vitro" OR microbiom* OR microbiota* OR mutat* OR pharmacokinetic* OR polymorphism* OR SNP OR spectrometry OR tissue*):ti,ab)

#52	OR/#35-#51

#53	#34 NOT #52

#54	#53 AND publication date between January 2016 to May 2022

Eligibility criteria:

- Inclusion criteria: ***A) Population:*** People aged 16 or above with non-specific low back pain with or without sciatica; People aged 16 or above with sciatica. ***B) Intervention:*** Psychological interventions: Cognitive therapy; Behavioural therapy; Cognitive behavioural approach (CBA); Acceptance and commitment therapy (ACT); Mindfulness; Any other non-invasive intervention included in the guideline; Combination of interventions: any combination of the non-invasive interventions; Placebo/Sham/Attention control; Usual care/waiting-list. ***C) Comparison:*** All interventions will be compared with each other, unless otherwise stated. ***D) Study Type:*** Randomized controlled trials, systematic reviews, and non-randomized studies.
- Exclusion criteria: None.

PRISMA flow diagram:

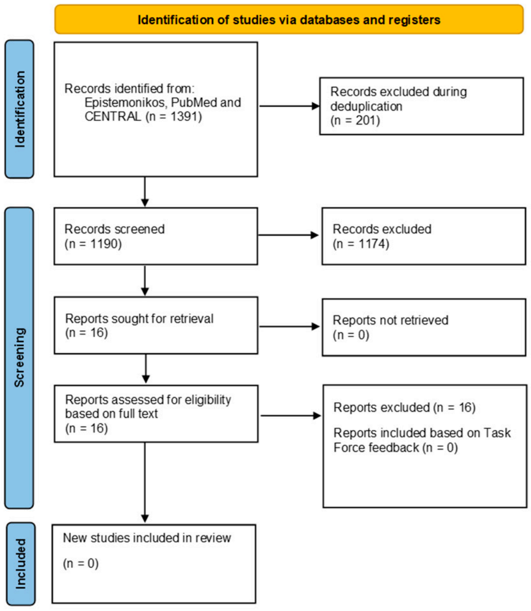

Question 7: Should epidural injections versus non-invasive treatments be used in patients with sciatica?

Databases searched: PubMed, Embase, and Cochrane

Search period: 01 July 2019 to 03 June 2022 (PubMed and Embase); July 2019 to June 2022 (Cochrane)

Search strategy used:

PubMed: ((((("Sciatica"[Mesh] OR Sciatic*[tiab] OR (("Radiculopathy"[Mesh] OR "Radicular Pain"[tiab] OR Radiculitides[tiab] OR Radiculitis[tiab] OR Radiculopath*[tiab] OR (("Spinal Nerve Roots"[Mesh] OR "Nerve Root"[tiab]) AND ("Inflammation"[Mesh] OR "Pain"[Mesh] OR Ache*[tiab] OR Aching[tiab] OR Avulsion*[tiab] OR Compress*[tiab] OR Disorder*[tiab] OR Entrap*[tiab] OR Imping*[tiab] OR Inflam*[tiab] OR Irritat*[tiab] OR Pinch*[tiab] OR Trap*[tiab]))) AND ("Low Back Pain"[Mesh] OR "Lumbar Vertebrae"[Mesh] OR "Lumbosacral Region"[Mesh] OR Low[tiab] OR Lower[tiab] OR Lumbar[tiab] OR Lumbosacral[tiab] OR "Lumbo-sacral"[tiab]))) AND ("Adrenal Cortex Hormones"[Mesh] OR "Analgesia, Epidural"[Mesh] OR "Anesthetics, Local"[Mesh] OR "Botulinum Toxins"[Mesh] OR "Hyaluronic Acid"[Mesh] OR "Injections"[Mesh] OR "Lidocaine"[Mesh] OR "Prolotherapy"[Mesh] OR "Regenerative Medicine"[Mesh] OR "Sclerosing Solutions"[Mesh] OR "Steroids"[Mesh] OR "Tumor Necrosis Factors"[Mesh] OR "Viscosupplementation"[Mesh] OR "Viscosupplements"[Mesh] OR "Anesthetics, Local"[Pharmacological Action] OR "Sclerosing Solutions"[Pharmacological Action] OR "Viscosupplements"[Pharmacological Action] OR "Adrenal Cortex Hormone*"[tiab] OR "Amo Vitrax"[tiab] OR Amvisc[tiab] OR Biolon[tiab] OR Botox[tiab] OR Botulin*[tiab] OR "Botulinum Neurotoxin*"[tiab] OR "Botulinum Toxin*"[tiab] OR "Clostridium botulinum Toxin*"[tiab] OR "Conduction Blocking Anesthetics*"[tiab] OR Corticoid*[tiab] OR Corticosteroid*[tiab] OR Dalcaine[tiab] OR Epidural*[tiab] OR Etamucine[tiab] OR Extradural[tiab] OR Glucocorticoid*[tiab] OR Glucocorticosteroid*[tiab] OR Healon[tiab] OR Hyaluronan[tiab] OR Hyaluronate*[tiab] OR Hyaluronic[tiab] OR Hyvisc[tiab] OR Injectable*[tiab] OR Injection*[tiab] OR "Lidocaine Carbonate"[tiab] OR "Lidocaine Hydrocarbonate"[tiab] OR "Lidocaine Hydrochloride"[tiab] OR Lidocaine[tiab] OR Lignocaine[tiab] OR "Local Anaesthes*"[tiab] OR "Local Anaesthetic*"[tiab] OR "Local Anesthes*"[tiab] OR "Local Anesthetic*"[tiab] OR Luronit[tiab] OR Octocaine[tiab] OR Peridural[tiab] OR "Proliferation Therap*"[tiab] OR Prolotherap*[tiab] OR "Regeneration Medicine*"[tiab] OR "Regeneration Therap*"[tiab] OR "Regenerative Therap*"[tiab] OR Scleros*[tiab] OR Sclerotherapy[tiab] OR Steroid*[tiab] OR "TNF Receptor Ligand*"[tiab] OR "Topical Anesthetic*"[tiab] OR "Tumor Necrosis Factor*"[tiab] OR "Visco Supplement*"[tiab] OR Viscosupplement*[tiab] OR Xylesthesin[tiab] OR Xylocaine[tiab] OR Xylocitin[tiab] OR Xyloneural[tiab] OR (("anti-tnf"[tiab] OR "tumor necrosis factor*"[tiab] OR "tumour necrosis factor*"[tiab]) AND (antagonist*[tiab] OR inhibit*[tiab])) OR ((caudal[tiab] OR interlaminar[tiab] OR lumbar[tiab] OR sacral[tiab] OR sacrum[tiab] OR transforaminal[tiab]) AND (analges*[tiab] OR block*[tiab]))) AND (2016/1/1:2022/5/18[pdat]) AND (english[Filter]) AND ("Randomized Controlled Trial"[Publication Type] OR placebo[tiab] OR random*[tiab] OR trial*[ti] OR "Meta-Analysis"[Publication Type] OR "Systematic Review"[Publication Type] OR "Systematic Reviews as Topic"[Mesh] OR metaanalys*[ti] OR meta-analys*[ti] OR "meta analys*"[ti] OR "systematic review"[ti] OR trial*[ti] OR ((meta[ti] OR systematic*[ti]) AND (overview*[ti] OR review*[ti] OR synthesis*[ti])))) NOT ("Chromatography"[Mesh] OR "Chromosomes"[Mesh] OR "Genetics"[Mesh] OR "Genome"[Mesh] OR "Genomics"[Mesh] OR "Genotype"[Mesh] OR "Mass Spectrometry"[Mesh] OR "Microbiota"[Mesh] OR "Mutation"[Mesh] OR "Pharmacokinetics"[Mesh] OR "Polymorphism, Genetic"[Mesh] OR "congenital" [Subheading] OR "genetics"[Subheading] OR "pharmacokinetics"[Subheading] OR assay*[tiab] OR cell[tiab] OR cells[tiab] OR chromosome*[tiab] OR genetic*[tiab] OR genome*[tiab] OR genomic*[tiab] OR "in vitro"[tiab] OR microbiom*[tiab] OR microbiota*[tiab] OR mutat*[tiab] OR pharmacokinetic*[tiab] OR polymorphism*[tiab] OR SNP[tiab] OR spectrometry[tiab] OR tissue*[tiab])) NOT (("Animals"[Mesh] OR animal*[tiab] OR ape[tiab] OR apes[tiab] OR canine*[tiab] OR cat[tiab] OR cats[tiab] OR chimpanzee*[tiab] OR dog[tiab] OR dogs[tiab] OR feline*[tiab] OR hamster*[tiab] OR lamb*[tiab] OR mice[tiab] OR monkey*[tiab] OR mouse[tiab] OR murine[tiab] OR pig[tiab] OR pigs[tiab] OR piglet*[tiab] OR porcine[tiab] OR primate*[tiab] OR rabbit*[tiab] OR rat[tiab] OR rats[tiab] OR rodent*[tiab] OR sheep*[tiab] OR swine[tiab]) NOT ("Humans"[Mesh] OR human*[tiab] OR man[tiab] OR men[tiab] OR patient*[tiab] OR woman[tiab] OR women[tiab]))) NOT ("address"[Publication Type] OR "autobiography"[Publication Type] OR "bibliography"[Publication Type] OR "biography"[Publication Type] OR "Book Illustrations"[Publication Type] OR "Case Reports"[Publication Type] OR "Comment"[Publication Type] OR "congress"[Publication Type] OR "consensus development conference"[Publication Type] OR "consensus development conference, nih"[Publication Type] OR "dictionary"[Publication Type] OR "directory"[Publication Type] OR "editorial"[Publication Type] OR

"Expression of Concern"[Publication Type] OR "Guideline"[Publication Type] OR "interactive tutorial"[Publication Type] OR "interview"[Publication Type] OR "lecture"[Publication Type] OR "legal case"[Publication Type] OR "legislation"[Publication Type] OR "letter"[Publication Type] OR "news"[Publication Type] OR "newspaper article"[Publication Type] OR "overall"[Publication Type] OR "patient education handout"[Publication Type] OR "periodical index"[Publication Type] OR "personal narrative"[Publication Type] OR "portrait"[Publication Type] OR "Review"[Publication Type] OR "Scientific Integrity Review"[Publication Type] OR "hascommenton"[All Fields] OR "Cartoons as Topic"[Mesh] OR "Review Literature as Topic"[Mesh] OR "case report*"[tiab] OR "case series"[tiab] OR "integrative research review*"[tiab] OR "integrative review*"[tiab] OR "literature review"[tiab] OR "narrative review"[tiab] OR "research integration"[tiab] OR "scoping review"[tiab] OR ((methodologic*[tiab] OR quantitative*[tiab]) AND (overview*[tiab] OR review*[tiab] OR synthesis*[tiab])))))

Embase: ((((('sciatica'/exp OR sciatic:ti,ab OR (('radicular pain'/exp OR 'radiculopathy'/exp OR (("Radicular Pain" OR Radiculitides OR Radiculitis OR Radiculopath*):ti,ab) OR (('nerve root'/exp OR "Nerve Root":ti,ab) AND ('inflammation'/exp OR 'pain'/exp OR ((Ache* OR Aching OR Avulsion* OR Compress* OR Disorder* OR Entrap* OR Imping* OR Inflam* OR Irritat* OR Pinch* OR Trap*):ti,ab)))) AND ('low back pain'/exp OR 'lumbar vertebra'/exp OR 'lumbosacral region'/exp OR ((Low OR Lower OR Lumbar OR Lumbosacral OR "Lumbo-sacral"):ti,ab)))) AND ('botulinum toxin'/exp OR 'corticosteroid'/exp OR 'epidural anesthesia'/exp OR 'hyaluronic acid'/exp OR 'injection'/exp OR 'lidocaine'/exp OR 'local anesthetic agent'/exp OR 'sclerosing agent'/exp OR 'steroid'/exp OR 'tumor necrosis factor'/exp OR 'viscosupplement'/exp OR 'viscosupplementation'/exp OR (("Adrenal Cortex Hormone*" OR "Amo Vitrax" OR Amvisc OR Biolon OR Botox OR Botulin* OR "Botulinum Neurotoxin*" OR "Botulinum Toxin*" OR "Clostridium botulinum Toxin*" OR "Conduction Blocking Anesthetics*" OR Corticoid* OR Corticosteroid* OR Dalcaine OR Epidural* OR Etamucine OR Extradural OR Glucocorticoid* OR Glucocorticosteroid* OR Healon OR Hyaluronan OR Hyaluronate* OR Hyaluronic OR Hyvisc OR Injectable* OR Injection* OR "Lidocaine Carbonate" OR "Lidocaine Hydrocarbonate" OR "Lidocaine Hydrochloride" OR Lidocaine OR Lignocaine OR "Local Anaesthes*" OR "Local Anaesthetic*" OR "Local Anesthes*" OR "Local Anesthetic*" OR Luronit OR Octocaine OR Peridural OR "Proliferation Therap*" OR Prolotherap* OR "Regeneration Medicine*" OR "Regeneration Therap*" OR "Regenerative Therap*" OR Scleros* OR Sclerotherapy OR Steroid* OR "TNF Receptor Ligand*" OR "Topical Anesthetic*" OR "Tumor Necrosis Factor*" OR "Visco Supplement*" OR Viscosupplement* OR Xylesthesin OR Xylocaine OR Xylocitin OR Xyloneural):ti,ab) OR ((("anti-tnf" OR "tumor necrosis factor*" OR "tumour necrosis factor*"):ti,ab) AND ((antagonist* OR inhibit*):ti,ab)) OR (((caudal OR interlaminar OR lumbar OR sacral OR sacrum OR transforaminal):ti,ab) AND ((analges* OR block*):ti,ab))) AND ([1-1-2016]/sd NOT [19-5-2022]/sd) AND ([english]/lim) AND ('randomized controlled trial'/exp OR placebo:ti,ab OR random*:ti,ab OR trial*:ti OR 'meta analysis'/exp OR 'meta analysis topic'/exp OR 'systematic review'/exp OR 'systematic review (topic)'/exp OR metaanalys*:ti OR meta-analys*:ti OR "meta analys*":ti OR "systematic review":ti OR trial*:ti OR (((meta OR systematic*) AND (overview* OR review* OR synthesis*)):ti))) NOT ('chromatography'/exp OR 'chromosome'/exp OR 'congenital'/exp OR 'genetic polymorphism'/exp OR 'genetics'/exp OR 'genome'/exp OR 'genomics'/exp OR 'genotype'/exp OR 'mass spectrometry'/exp OR 'microbiome'/exp OR 'mutation'/exp OR 'pharmacokinetics'/exp OR 'polymorphism'/exp OR assay*:ti,ab OR cell:ti,ab OR cells:ti,ab OR chromosome*:ti,ab OR genetic*:ti,ab OR genome*:ti,ab OR genomic*:ti,ab OR "in vitro":ti,ab OR microbiom*:ti,ab OR microbiota*:ti,ab OR mutat*:ti,ab OR pharmacokinetic*:ti,ab OR polymorphism*:ti,ab OR SNP:ti,ab OR spectrometry:ti,ab OR tissue*:ti,ab)) NOT (('animal'/exp OR animal*:ti,ab OR ape:ti,ab OR apes:ti,ab OR canine*:ti,ab OR cat:ti,ab OR cats:ti,ab OR chimpanzee*:ti,ab OR dog:ti,ab OR dogs:ti,ab OR feline*:ti,ab OR hamster*:ti,ab OR lamb*:ti,ab OR mice:ti,ab OR monkey*:ti,ab OR mouse:ti,ab OR murine:ti,ab OR pig:ti,ab OR pigs:ti,ab OR piglet*:ti,ab OR porcine:ti,ab OR primate*:ti,ab OR rabbit*:ti,ab OR rat:ti,ab OR rats:ti,ab OR rodent*:ti,ab OR sheep*:ti,ab OR swine:ti,ab) NOT ('human'/exp OR human*:ti,ab OR man:ti,ab OR men:ti,ab OR patient*:ti,ab OR woman:ti,ab OR women:ti,ab))) NOT (('abstract report'/exp OR 'animal experiment'/exp OR 'book'/exp OR 'case finding'/exp OR 'case report'/exp OR 'case study'/exp OR 'conference paper'/exp OR 'editorial'/exp OR 'feasibility study'/exp OR 'in vitro study'/exp) AND

'letter'/exp OR 'note'/exp OR 'practice guideline'/exp OR 'review'/exp OR 'veterinary clinical trial'/exp OR 'veterinary study'/exp OR [conference abstract]/lim OR [conference paper]/lim OR [conference review]/lim OR [editorial]/lim OR [letter]/lim OR [note]/lim OR [short survey]/lim OR 'case report*':ti,ab OR 'case series':ti,ab OR 'integrative research review*':ti,ab OR 'integrative review*':ti,ab OR 'literature review':ti,ab OR 'narrative review':ti,ab OR 'research integration':ti,ab OR 'scoping review':ti,ab OR ((integrative NEAR/5 research NEAR/5 review*):ti,ab) OR ((methodologic* NEAR/5 overview*):ti,ab) OR ((methodologic* NEAR/5 review*):ti,ab) OR ((quantitativ* NEAR/5 overview*):ti,ab) OR ((quantitativ* NEAR/5 review*):ti,ab) OR ((quantitativ* NEAR/5 synthesi*):ti,ab) OR ((research NEAR/5 integration):ti,ab)))

Cochrane:

ID	Search
#1	MeSH descriptor: [Sciatica] explode all trees
#2	Sciatic*:ti,ab
#3	MeSH descriptor: [Radiculopathy] explode all trees
#4	(("Radicular Pain" OR Radiculitides OR Radiculitis OR Radiculopath*):ti,ab)
#5	#3 OR #4
#6	MeSH descriptor: [Spinal Nerve Roots] explode all trees
#7	"Nerve Root":ti,ab
#8	#6 OR #7
#9	MeSH descriptor: [Inflammation] explode all trees
#10	MeSH descriptor: [Pain] explode all trees
#11	((Ache* OR Aching OR Avulsion* OR Compress* OR Disorder* OR Entrap* OR Imping* OR Inflam* OR Irritat* OR Pinch* OR Trap*):ti,ab)
#12	OR/#9-#11
#13	#5 OR #8 OR #12
#14	(MeSH descriptor: [Low Back Pain] explode all trees
#15	MeSH descriptor: [Lumbar Vertebrae] explode all trees
#16	MeSH descriptor: [Lumbosacral Region] explode all trees
#17	((Low OR Lower OR Lumbar OR Lumbosacral OR "Lumbo-sacral"):ti,ab)
#18	OR/#14-#17
#19	#13 AND #18
#20	#1 OR #2 OR #19
#21	MeSH descriptor: [Adrenal Cortex Hormones] explode all trees
#22	MeSH descriptor: [Analgesia, Epidural] explode all trees
#23	MeSH descriptor: [Anesthetics, Local] explode all trees
#24	MeSH descriptor: [Botulinum Toxins] explode all trees
#25	MeSH descriptor: [Hyaluronic Acid] explode all trees
#26	MeSH descriptor: [Injections] explode all trees
#27	MeSH descriptor: [Lidocaine] explode all trees
#28	MeSH descriptor: [Prolotherapy] explode all trees

#29 MeSH descriptor: [Regenerative Medicine] explode all trees

#30 MeSH descriptor: [Sclerosing Solutions] explode all trees

#31 MeSH descriptor: [Steroids] explode all trees

#32 MeSH descriptor: [Tumor Necrosis Factors] explode all trees

#33 MeSH descriptor: [Viscosupplementation] explode all trees

#34 MeSH descriptor: [Viscosupplements] explode all trees

#35 (("Adrenal Cortex Hormone*" OR "Amo Vitrax" OR Amvisc OR Biolon OR Botox OR Botulin* OR "Botulinum Neurotoxin*" OR "Botulinum Toxin*" OR "Clostridium botulinum Toxin*" OR "Conduction Blocking Anesthetics*" OR Corticoid* OR Corticosteroid* OR Dalcaine OR Epidural* OR Etamucine OR Extradural OR Glucocorticoid* OR Glucocorticosteroid* OR Healon OR Hyaluronan OR Hyaluronate* OR Hyaluronic OR Hyvisc OR Injectable* OR Injection* OR "Lidocaine Carbonate" OR "Lidocaine Hydrocarbonate" OR "Lidocaine Hydrochloride" OR Lidocaine OR Lignocaine OR "Local Anaesthes*" OR "Local Anaesthetic*" OR "Local Anesthes*" OR "Local Anesthetic*" OR Luronit OR Octocaine OR Peridural OR "Proliferation Therap*" OR Prolotherap* OR "Regeneration Medicine*" OR "Regeneration Therap*" OR "Regenerative Therap*" OR Scleros* OR Sclerotherapy OR Steroid* OR "TNF Receptor Ligand*" OR "Topical Anesthetic*" OR "Tumor Necrosis Factor*" OR "Visco Supplement*" OR Viscosupplement* OR Xylesthesin OR Xylocaine OR Xylocitin OR Xyloneural):ti,ab) OR ((("anti-tnf" OR "tumor necrosis factor*" OR "tumour necrosis factor*"):ti,ab) AND ((antagonist* OR inhibit*):ti,ab)) OR (((caudal OR interlaminar OR lumbar OR sacral OR sacrum OR transforaminal):ti,ab) AND ((analges* OR block*):ti,ab))

#36 OR/#21-#35

#37 #20 AND #36

#38 MeSH descriptor: [Biomarkers] explode all trees

#39 MeSH descriptor: [Chromatography] explode all trees

#40 MeSH descriptor: [Chromosomes] explode all trees

#41 MeSH descriptor: [Echocardiography] explode all trees

#42 MeSH descriptor: [Genetics] in all MeSH products

#43 MeSH descriptor: [Genome] in all MeSH products

#44 MeSH descriptor: [Genotype] explode all trees

#45 MeSH descriptor: [Incidence] explode all trees

#46 MeSH descriptor: [Mass Spectrometry] explode all trees

#47 MeSH descriptor: [Microbiota] explode all trees

#48 MeSH descriptor: [Mutation] explode all trees

#49 MeSH descriptor: [Pharmacokinetics] explode all trees

#50 MeSH descriptor: [Polymorphism, Genetic] explode all trees

#51 Any MeSH descriptor in all MeSH products and with qualifier(s): [congenital - CN]

#52 Any MeSH descriptor in all MeSH products and with qualifier(s): [genetics - GE]

#53 Any MeSH descriptor in all MeSH products and with qualifier(s): [pharmacokinetics - PK]

#54 ((assay* OR biomarker* OR cell OR cells OR chromosome* OR genetic* OR genome* OR genomic* OR "in vitro" OR microbiom* OR microbiota* OR mutat* OR pharmacokinetic* OR polymorphism* OR SNP OR spectrometry OR tissue*):ti,ab)

#55 OR/#38-#54

#56 #37 NOT #55

#57 #56 AND publication date between January 2016 and May 2022

Eligibility criteria:

- Inclusion criteria: ***A) Population:*** People aged 16 or above with non-specific low back pain with or without sciatica; People aged 16 or above with sciatica. ***B) Intervention:*** Epidural injections: Steroid (including steroid plus saline), Local anaesthetic, Anti-TNF, Combination: local anaesthetic+steroid. ***C) Comparison:*** Sham (needle alone)/placebo/saline, Usual care, Each other (including head-to-head comparisons between strata), Other treatment (non-invasive and invasive treatments being considered by the guideline for sciatica). ***D) Study Type:*** Randomized controlled trials, systematic reviews, and non-randomized studies.
- Exclusion criteria: None.

PRISMA flow diagram:

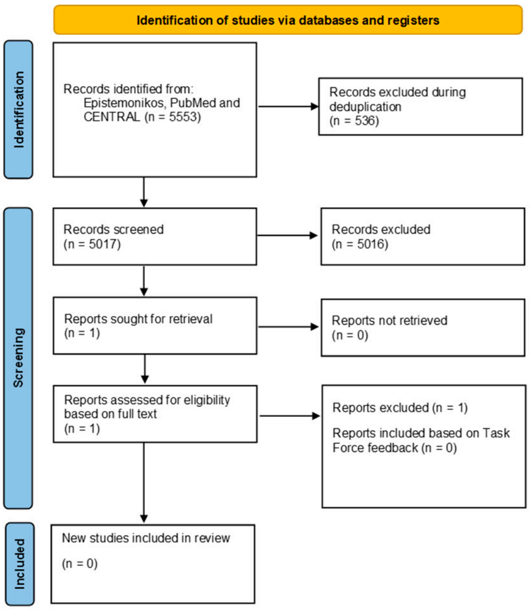

Question 8: Should image-concordant pathology or the presence of radicular symptoms versus no image-concordant pathology or the presence of radicular symptoms be used for predicting response to surgery in patients with suspected sciatica?

Databases searched: PubMed, Embase, and Cochrane

Search period: 01 January 2016 to 18 May 2022 (PubMed and Embase); January 2016 to May 2022 (Cochrane)

Search strategy used:

PubMed: ((((("Sciatica"[Mesh] OR Sciatic*[tiab] OR (("Radiculopathy"[Mesh] OR "Radicular Pain"[tiab] OR Radiculitides[tiab] OR Radiculitis[tiab] OR Radiculopath*[tiab] OR (("Spinal Nerve Roots"[Mesh] OR "Nerve Root"[tiab]) AND ("Inflammation"[Mesh] OR "Pain"[Mesh] OR Ache*[tiab] OR Aching[tiab] OR Avulsion*[tiab] OR Compress*[tiab] OR Disorder*[tiab] OR Entrap*[tiab] OR Imping*[tiab] OR Inflam*[tiab] OR Irritat*[tiab] OR Pinch*[tiab] OR Trap*[tiab]))) AND ("Low Back Pain"[Mesh] OR "Lumbar Vertebrae"[Mesh] OR "Lumbosacral Region"[Mesh] OR Low[tiab] OR Lower[tiab] OR Lumbar[tiab] OR Lumbosacral[tiab] OR "Lumbo-sacral"[tiab]))) AND ((("Pathology"[Mesh] OR "pathology"[Subheading] OR neuropatholog*[tiab] OR patholog*[tiab]) AND ("Diagnostic Imaging"[Mesh] OR "diagnostic imaging"[Subheading] OR image*[tiab] OR imaging*[tiab] OR neuroimage*[tiab])) OR (("Radiculopathy"[Mesh] OR "nerve root avulsion*"[tiab]

OR "nerve root compression*"[tiab] OR "nerve root disorder*"[tiab] OR "nerve root inflammation*"[tiab] OR radiculitides[tiab] OR radiculitis[tiab] OR radiculopath*[tiab]) AND ("Neurologic Manifestations"[Mesh] OR "Pain"[Mesh] OR "Signs and Symptoms"[Mesh] OR ache*[tiab] OR aching*[tiab] OR deficit*[tiab] OR dysfunction*[tiab] OR manifest*[tiab] OR pain*[tiab] OR sign[tiab] OR signs[tiab] OR symptom*[tiab]))) AND ("Ambulatory Surgical Procedures"[Mesh] OR "Elective Surgical Procedures"[Mesh] OR "General Surgery"[Mesh] OR "Neurosurgery"[Mesh] OR "Neurosurgical Procedures"[Mesh] OR "surgery"[Subheading] OR "intraoperative procedur*"[tiab] OR "intraoperative therap*"[tiab] OR "invasive procedur*"[tiab] OR neurosurger*[tiab] OR neurosurgical[tiab] OR operation*[tiab] OR "operative procedur*"[tiab] OR "operative therap*"[tiab] OR "perioperative procedur*"[tiab] OR "perioperative therap*"[tiab] OR "peroperative procedur*"[tiab] OR "peroperative therap*"[tiab] OR "preoperative procedur*"[tiab] OR "preoperative therap*"[tiab] OR surger*[tiab] OR "surgical procedur*"[tiab] OR "surgical therap*"[tiab]) AND (2016/1/1:2022/5/18[pdat]) AND (english[Filter]) AND ("Cohort Studies"[Mesh] OR "cohort analys*"[tiab] OR "cohort stud*"[tiab] OR "concurrent stud*"[tiab] OR "incidence stud*"[tiab] OR "Randomized Controlled Trial"[Publication Type] OR placebo[tiab] OR random*[tiab] OR trial*[ti] OR "Meta-Analysis"[Publication Type] OR "Systematic Review"[Publication Type] OR "Systematic Reviews as Topic"[Mesh] OR metaanalys*[ti] OR meta-analys*[ti] OR "meta analys*"[ti] OR "systematic review"[ti] OR trial*[ti] OR ((meta[ti] OR systematic*[ti]) AND (overview*[ti] OR review*[ti] OR synthesis*[ti])))) NOT ("Chromatography"[Mesh] OR "Chromosomes"[Mesh] OR "Genetics"[Mesh] OR "Genome"[Mesh] OR "Genomics"[Mesh] OR "Genotype"[Mesh] OR "Mass Spectrometry"[Mesh] OR "Microbiota"[Mesh] OR "Mutation"[Mesh] OR "Pharmacokinetics"[Mesh] OR "Polymorphism, Genetic"[Mesh] OR "congenital" [Subheading] OR "genetics"[Subheading] OR "pharmacokinetics"[Subheading] OR assay*[tiab] OR cell[tiab] OR cells[tiab] OR chromosome*[tiab] OR genetic*[tiab] OR genome*[tiab] OR genomic*[tiab] OR "in vitro"[tiab] OR microbiom*[tiab] OR microbiota*[tiab] OR mutat*[tiab] OR pharmacokinetic*[tiab] OR polymorphism*[tiab] OR SNP[tiab] OR spectrometry[tiab] OR tissue*[tiab])) NOT (("Animals"[Mesh] OR animal*[tiab] OR ape[tiab] OR apes[tiab] OR canine*[tiab] OR cat[tiab] OR cats[tiab] OR chimpanzee*[tiab] OR dog[tiab] OR dogs[tiab] OR feline*[tiab] OR hamster*[tiab] OR lamb*[tiab] OR mice[tiab] OR monkey*[tiab] OR mouse[tiab] OR murine[tiab] OR pig[tiab] OR pigs[tiab] OR piglet*[tiab] OR porcine[tiab] OR primate*[tiab] OR rabbit*[tiab] OR rat[tiab] OR rats[tiab] OR rodent*[tiab] OR sheep*[tiab] OR swine[tiab]) NOT ("Humans"[Mesh] OR human*[tiab] OR man[tiab] OR men[tiab] OR patient*[tiab] OR woman[tiab] OR women[tiab]))) NOT ("address"[Publication Type] OR "autobiography"[Publication Type] OR "bibliography"[Publication Type] OR "biography"[Publication Type] OR "Book Illustrations"[Publication Type] OR "Case Reports"[Publication Type] OR "Comment"[Publication Type] OR "congress"[Publication Type] OR "consensus development conference"[Publication Type] OR "consensus development conference, nih"[Publication Type] OR "dictionary"[Publication Type] OR "directory"[Publication Type] OR "editorial"[Publication Type] OR "Expression of Concern"[Publication Type] OR "Guideline"[Publication Type] OR "interactive tutorial"[Publication Type] OR "interview"[Publication Type] OR "lecture"[Publication Type] OR "legal case"[Publication Type] OR "legislation"[Publication Type] OR "letter"[Publication Type] OR "news"[Publication Type] OR "newspaper article"[Publication Type] OR "overall"[Publication Type] OR "patient education handout"[Publication Type] OR "periodical index"[Publication Type] OR "personal narrative"[Publication Type] OR "portrait"[Publication Type] OR "Review"[Publication Type] OR "Scientific Integrity Review"[Publication Type] OR "hascommenton"[All Fields] OR "Cartoons as Topic"[Mesh] OR "Review Literature as Topic"[Mesh] OR "case report*"[tiab] OR "case series"[tiab] OR "integrative research review*"[tiab] OR "integrative review*"[tiab] OR "literature review"[tiab] OR "narrative review"[tiab] OR "research integration"[tiab] OR "scoping review"[tiab] OR ((methodologic*[tiab] OR quantitative*[tiab]) AND (overview*[tiab] OR review*[tiab] OR synthesis*[tiab]))))

Embase: ((((('sciatica'/exp OR sciatic:ti,ab OR (('radicular pain'/exp OR 'radiculopathy'/exp OR (("Radicular Pain" OR Radiculitides OR Radiculitis OR Radiculopath*):ti,ab) OR (('nerve root'/exp OR "Nerve Root":ti,ab) AND ('inflammation'/exp OR 'pain'/exp OR ((Ache* OR Aching OR Avulsion* OR Compress* OR Disorder* OR Entrap* OR Imping* OR Inflam* OR Irritat* OR Pinch* OR Trap*):ti,ab)))) AND ('low back pain'/exp OR 'lumbar vertebra'/exp OR 'lumbosacral region'/exp OR

((Low OR Lower OR Lumbar OR Lumbosacral OR "Lumbo-sacral"):ti,ab)))) AND ((('pathology'/exp OR ((neuropatholog* OR patholog*):ti,ab)) AND ('diagnostic imaging'/exp OR ((image* OR imaging* OR neuroimage*):ti,ab))) OR (('radiculopathy'/exp OR (("nerve root avulsion*" OR "nerve root compression*" OR "nerve root disorder*" OR "nerve root inflammation*" OR radiculitides OR radiculitis OR radiculopath*):ti,ab)) AND ('physical disease by body function'/exp OR ((ache* OR aching* OR deficit* OR dysfunction* OR manifest* OR pain* OR sign OR signs OR symptom*):ti,ab)))) AND ('ambulatory surgery'/exp OR 'elective surgery'/exp OR 'general surgery'/exp OR 'neurosurgery'/exp OR 'neurosurgery'/exp OR (("intraoperative procedur*" OR "intraoperative therap*" OR "invasive procedur*" OR neurosurger* OR neurosurgical OR operation* OR "operative procedur*" OR "operative therap*" OR "perioperative procedur*" OR "perioperative therap*" OR "peroperative procedur*" OR "peroperative therap*" OR "preoperative procedur*" OR "preoperative therap*" OR surger* OR "surgical procedur*" OR "surgical therap*"):ti,ab)) AND ([1-1-2016]/sd NOT [19-5-2022]/sd) AND ([english]/lim) AND ('cohort analysis'/exp OR "cohort analys*":ti,ab OR "cohort stud*":ti,ab OR "concurrent stud*":ti,ab OR "incidence stud*":ti,ab OR 'randomized controlled trial'/exp OR placebo:ti,ab OR random*:ti,ab OR trial*:ti OR 'meta analysis'/exp OR 'meta analysis topic'/exp OR 'systematic review'/exp OR 'systematic review (topic)'/exp OR metaanalys*:ti OR meta-analys*:ti OR "meta analys*":ti OR "systematic review":ti OR trial*:ti OR (((meta OR systematic*) AND (overview* OR review* OR synthesis*)):ti))) NOT ('chromatography'/exp OR 'chromosome'/exp OR 'congenital'/exp OR 'genetic polymorphism'/exp OR 'genetics'/exp OR 'genome'/exp OR 'genomics'/exp OR 'genotype'/exp OR 'mass spectrometry'/exp OR 'microbiome'/exp OR 'mutation'/exp OR 'pharmacokinetics'/exp OR 'polymorphism'/exp OR assay*:ti,ab OR cell:ti,ab OR cells:ti,ab OR chromosome*:ti,ab OR genetic*:ti,ab OR genome*:ti,ab OR genomic*:ti,ab OR "in vitro":ti,ab OR microbiom*:ti,ab OR microbiota*:ti,ab OR mutat*:ti,ab OR pharmacokinetic*:ti,ab OR polymorphism*:ti,ab OR SNP:ti,ab OR spectrometry:ti,ab OR tissue*:ti,ab)) NOT (('animal'/exp OR animal*:ti,ab OR ape:ti,ab OR apes:ti,ab OR canine*:ti,ab OR cat:ti,ab OR cats:ti,ab OR chimpanzee*:ti,ab OR dog:ti,ab OR dogs:ti,ab OR feline*:ti,ab OR hamster*:ti,ab OR lamb*:ti,ab OR mice:ti,ab OR monkey*:ti,ab OR mouse:ti,ab OR murine:ti,ab OR pig:ti,ab OR pigs:ti,ab OR piglet*:ti,ab OR porcine:ti,ab OR primate*:ti,ab OR rabbit*:ti,ab OR rat:ti,ab OR rats:ti,ab OR rodent*:ti,ab OR sheep*:ti,ab OR swine:ti,ab) NOT ('human'/exp OR human*:ti,ab OR man:ti,ab OR men:ti,ab OR patient*:ti,ab OR woman:ti,ab OR women:ti,ab))) NOT (('abstract report'/exp OR 'animal experiment'/exp OR 'book'/exp OR 'case finding'/exp OR 'case report'/exp OR 'case study'/exp OR 'conference paper'/exp OR 'editorial'/exp OR 'feasibility study'/exp OR 'in vitro study'/exp) AND 'letter'/exp OR 'note'/exp OR 'practice guideline'/exp OR 'review'/exp OR 'veterinary clinical trial'/exp OR 'veterinary study'/exp OR [conference abstract]/lim OR [conference paper]/lim OR [conference review]/lim OR [editorial]/lim OR [letter]/lim OR [note]/lim OR [short survey]/lim OR 'case report*':ti,ab OR 'case series':ti,ab OR 'integrative research review*':ti,ab OR 'integrative review*':ti,ab OR 'literature review':ti,ab OR 'narrative review':ti,ab OR 'research integration':ti,ab OR 'scoping review':ti,ab OR ((integrative NEAR/5 research NEAR/5 review*):ti,ab) OR ((methodologic* NEAR/5 overview*):ti,ab) OR ((methodologic* NEAR/5 review*):ti,ab) OR ((quantitativ* NEAR/5 overview*):ti,ab) OR ((quantitativ* NEAR/5 review*):ti,ab) OR ((quantitativ* NEAR/5 synthesi*):ti,ab) OR ((research NEAR/5 integration):ti,ab)))

Cochrane:

**ID**	**Search**
#1	MeSH descriptor: [Sciatica] explode all trees
#2	Sciatic*:ti,ab
#3	MeSH descriptor: [Radiculopathy] explode all trees
#4	(("Radicular Pain" OR Radiculitides OR Radiculitis OR Radiculopath*):ti,ab)
#5	#3 OR #4
#6	MeSH descriptor: [Spinal Nerve Roots] explode all trees
#7	"Nerve Root":ti,ab

#8 #6 OR #7

#9 MeSH descriptor: [Inflammation] explode all trees

#10 MeSH descriptor: [Pain] explode all trees

#11 ((Ache* OR Aching OR Avulsion* OR Compress* OR Disorder* OR Entrap* OR Imping* OR Inflam* OR Irritat* OR Pinch* OR Trap*):ti,ab)

#12 OR/#9-#11

#13 #5 OR #8 OR #12

#14 (MeSH descriptor: [Low Back Pain] explode all trees

#15 MeSH descriptor: [Lumbar Vertebrae] explode all trees

#16 MeSH descriptor: [Lumbosacral Region] explode all trees

#17 ((Low OR Lower OR Lumbar OR Lumbosacral OR "Lumbo-sacral"):ti,ab)

#18 OR/#14-#17

#19 #13 AND #18

#20 #1 OR #2 OR #19

#21 MeSH descriptor: [Pathology] explode all trees

#22 MeSH descriptor: [] explode all trees and with qualifier(s): [pathology - PA]

#23 ((neuropatholog* OR patholog*):ti,ab)

#24 OR/#21-#23

#25 MeSH descriptor: [Diagnostic Imaging] explode all trees

#26 MeSH descriptor: [] explode all trees and with qualifier(s): [diagnostic imaging - DG]

#27 ((image* OR imaging* OR neuroimage*):ti,ab)

#28 OR/#25-#27

#29 #24 AND #28

#30 MeSH descriptor: [Radiculopathy] explode all trees

#31 (("nerve root avulsion*" OR "nerve root compression*" OR "nerve root disorder*" OR "nerve root inflammation*" OR radiculitides OR radiculitis OR radiculopath*):ti,ab)

#32 #30 OR #31

#33 MeSH descriptor: [Neurologic Manifestations] explode all trees

#34 MeSH descriptor: [Pain] explode all trees

#35 MeSH descriptor: [Signs and Symptoms] explode all trees

#36 ((ache* OR aching* OR deficit* OR dysfunction* OR manifest* OR pain* OR sign OR signs OR symptom*):ti,ab)

#37 OR/#33-#36

#38 #32 AND #37

#39 #29 OR #38

#40 MeSH descriptor: [Ambulatory Surgical Procedures] explode all trees

#41 MeSH descriptor: [Elective Surgical Procedures] explode all trees

#42 MeSH descriptor: [General Surgery] explode all trees

#43 MeSH descriptor: [Neurosurgery] explode all trees

#44 MeSH descriptor: [Neurosurgical Procedures] explode all trees

#45 MeSH descriptor: [] explode all trees and with qualifier(s): [surgery - SU]

#46 (("intraoperative procedur*" OR "intraoperative therap*" OR "invasive procedur*" OR neurosurger* OR neurosurgical OR operation* OR "operative procedur*" OR "operative therap*" OR "perioperative procedur*" OR "perioperative therap*" OR "peroperative procedur*" OR "peroperative therap*" OR "preoperative procedur*" OR "preoperative therap*" OR surger* OR "surgical procedur*" OR "surgical therap*"):ti,ab)

#47 OR/#40-#46

#48 #20 AND #39 AND #47

#49 MeSH descriptor: [Biomarkers] explode all trees

#50 MeSH descriptor: [Chromatography] explode all trees

#51 MeSH descriptor: [Chromosomes] explode all trees

#52 MeSH descriptor: [Echocardiography] explode all trees

#53 MeSH descriptor: [Genetics] in all MeSH products

#54 MeSH descriptor: [Genome] in all MeSH products

#55 MeSH descriptor: [Genotype] explode all trees

#56 MeSH descriptor: [Incidence] explode all trees

#57 MeSH descriptor: [Mass Spectrometry] explode all trees

#58 MeSH descriptor: [Microbiota] explode all trees

#59 MeSH descriptor: [Mutation] explode all trees

#60 MeSH descriptor: [Pharmacokinetics] explode all trees

#61 MeSH descriptor: [Polymorphism, Genetic] explode all trees

#62 Any MeSH descriptor in all MeSH products and with qualifier(s): [congenital - CN]

#63 Any MeSH descriptor in all MeSH products and with qualifier(s): [genetics - GE]

#64 Any MeSH descriptor in all MeSH products and with qualifier(s): [pharmacokinetics - PK]

#65 ((assay* OR biomarker* OR cell OR cells OR chromosome* OR genetic* OR genome* OR genomic* OR "in vitro" OR microbiom* OR microbiota* OR mutat* OR pharmacokinetic* OR polymorphism* OR SNP OR spectrometry OR tissue*):ti,ab)

#66 OR/#49-#65

#67 #48 NOT #66

#68 #67 AND publication date between January 2016 and May 2022

Eligibility criteria:

- Inclusion criteria: ***A) Population:*** People aged 16 or above with non-specific low back pain with or without sciatica; People aged 16 or above with sciatica. ***B) Intervention:*** Image concordant pathology (diagnosis supported by imaging - i.e. MRI or CT to see if compression is present or not); Radicular symptoms (pain that extends to leg vs. pain in back/buttock only). ***C) Comparison:*** Duration of symptoms**.** ***D) Study Type:*** Prospective and retrospective cohorts (with multivariate analysis adjusted for key confounders (if none are identified those with multivariate analysis adjusted for other confounders will be included) or randomised trials (if appropriate) with multivariate analysis adjusted for key confounders (if none are identified those with multivariate analysis adjusted for other confounders will be included).

- Exclusion criteria: None.

PRISMA flow diagram:

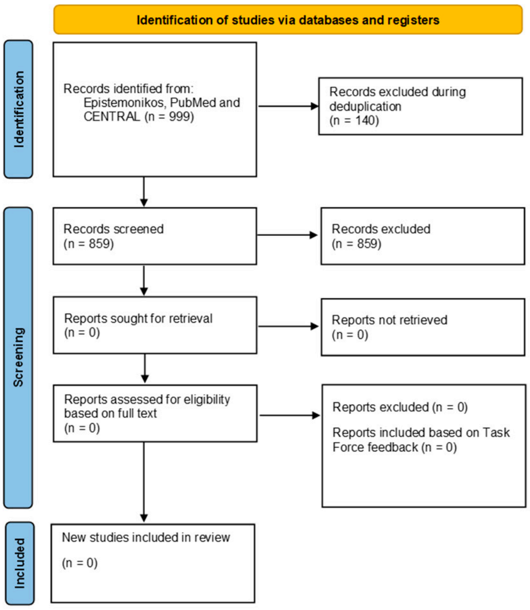

Question 9: Should spinal decompression versus usual care or other interventions be used in patients with sciatica?

Databases searched: PubMed, Embase, and Cochrane

Search period: 01 January 2016 to 19 May 2022 (PubMed and Embase); January 2016 to May 2022 (Cochrane)

Search strategy used:

PubMed: ((((("Sciatica"[Mesh] OR Sciatic*[tiab] OR (("Radiculopathy"[Mesh] OR "Radicular Pain"[tiab] OR Radiculitides[tiab] OR Radiculitis[tiab] OR Radiculopath*[tiab] OR (("Spinal Nerve Roots"[Mesh] OR "Nerve Root"[tiab]) AND ("Inflammation"[Mesh] OR "Pain"[Mesh] OR Ache*[tiab] OR Aching[tiab] OR Avulsion*[tiab] OR Compress*[tiab] OR Disorder*[tiab] OR Entrap*[tiab] OR Imping*[tiab] OR Inflam*[tiab] OR Irritat*[tiab] OR Pinch*[tiab] OR Trap*[tiab]))) AND ("Low Back Pain"[Mesh] OR "Lumbar Vertebrae"[Mesh] OR "Lumbosacral Region"[Mesh] OR Low[tiab] OR Lower[tiab] OR Lumbar[tiab] OR Lumbosacral[tiab] OR "Lumbo-sacral"[tiab]))) AND ("Decompression, Surgical"[Mesh:NoExp] OR "Diskectomy"[Mesh] OR "Foraminotomy"[Mesh] OR "Laminectomy"[Mesh] OR "Lumbar Vertebrae/surgery"[Mesh] OR "Total Disc Replacement"[Mesh] OR Accutherm[tiab] OR APLD[tiab] OR "Artificial Disc Replacement*"[tiab] OR "Artificial Disk Replacement*"[tiab] OR "Disc Replacement Arthroplast*"[tiab] OR Discectom*[tiab] OR Disctrode[tiab] OR Diskectom*[tiab] OR Disktrode[tiab] OR "Disk Replacement Arthroplast*"[tiab] OR Facetectom*[tiab] OR Fenestrat*[tiab] OR Foraminotom*[tiab] OR Laminectom*[tiab] OR Laminoplast*[tiab] OR Laminotom*[tiab] OR Microdecompression*[tiab] OR Microdiscectom*[tiab] OR Microdiskectom*[tiab] OR "Replacement Disc Arthroplast*"[tiab] OR "Replacement Disk Arthroplast*"[tiab] OR Sequestrectom*[tiab] OR Spinecath[tiab] OR "Total Disc Arthroplast*"[tiab] OR "Total Disc Replacement*"[tiab] OR "Total Disk Arthroplast*"[tiab] OR "Total Disk Replacement*"[tiab] OR Transdiscal[tiab] OR (decompress*[tiab] OR (disc[tiab] OR disk[tiab] OR intradiscal[tiab] OR lumb*[tiab] OR surg*[tiab])) OR ((disc[tiab] OR disk[tiab] OR intervertebral[tiab] OR intradiscal[tiab] OR percutaneous[tiab]) AND (annuloplast*[tiab] OR arthroplast*[tiab] OR biacuplast*[tiab] OR electrothermal[tiab] OR thermomodulation[tiab])) OR ((disc[tiab] or disk[tiab]) AND (excis*[tiab] OR remov*[tiab] OR replac*[tiab] OR resect*[tiab] OR surg*[tiab])) OR (lamina[tiab] AND (arthroplast*[tiab] OR excis*[tiab] OR remov*[tiab] OR replac*[tiab] OR

resect*[tiab] OR surg*[tiab])) OR (lumb*[tiab] AND (arthroplast*[tiab] OR excis*[tiab] OR remov*[tiab] OR replac*[tiab] OR resect*[tiab] OR surg*[tiab])) OR (micro*[tiab] AND (endoscop*[tiab] OR laser[tiab] OR surg*[tiab])) OR (thermal[tiab] AND procedure*[tiab])) AND (2016/1/1:2022/5/19[pdat]) AND (english[Filter]) AND ("Randomized Controlled Trial"[Publication Type] OR placebo[tiab] OR random*[tiab] OR trial*[ti] OR "Meta-Analysis"[Publication Type] OR "Systematic Review"[Publication Type] OR "Systematic Reviews as Topic"[Mesh] OR metaanalys*[ti] OR meta-analys*[ti] OR "meta analys*"[ti] OR "systematic review"[ti] OR trial*[ti] OR ((meta[ti] OR systematic*[ti]) AND (overview*[ti] OR review*[ti] OR synthesis*[ti])))) NOT ("Chromatography"[Mesh] OR "Chromosomes"[Mesh] OR "Genetics"[Mesh] OR "Genome"[Mesh] OR "Genomics"[Mesh] OR "Genotype"[Mesh] OR "Mass Spectrometry"[Mesh] OR "Microbiota"[Mesh] OR "Mutation"[Mesh] OR "Pharmacokinetics"[Mesh] OR "Polymorphism, Genetic"[Mesh] OR "congenital" [Subheading] OR "genetics"[Subheading] OR "pharmacokinetics"[Subheading] OR assay*[tiab] OR cell[tiab] OR cells[tiab] OR chromosome*[tiab] OR genetic*[tiab] OR genome*[tiab] OR genomic*[tiab] OR "in vitro"[tiab] OR microbiom*[tiab] OR microbiota*[tiab] OR mutat*[tiab] OR pharmacokinetic*[tiab] OR polymorphism*[tiab] OR SNP[tiab] OR spectrometry[tiab] OR tissue*[tiab])) NOT (("Animals"[Mesh] OR animal*[tiab] OR ape[tiab] OR apes[tiab] OR canine*[tiab] OR cat[tiab] OR cats[tiab] OR chimpanzee*[tiab] OR dog[tiab] OR dogs[tiab] OR feline*[tiab] OR hamster*[tiab] OR lamb*[tiab] OR mice[tiab] OR monkey*[tiab] OR mouse[tiab] OR murine[tiab] OR pig[tiab] OR pigs[tiab] OR piglet*[tiab] OR porcine[tiab] OR primate*[tiab] OR rabbit*[tiab] OR rat[tiab] OR rats[tiab] OR rodent*[tiab] OR sheep*[tiab] OR swine[tiab]) NOT ("Humans"[Mesh] OR human*[tiab] OR man[tiab] OR men[tiab] OR patient*[tiab] OR woman[tiab] OR women[tiab]))) NOT ("address"[Publication Type] OR "autobiography"[Publication Type] OR "bibliography"[Publication Type] OR "biography"[Publication Type] OR "Book Illustrations"[Publication Type] OR "Case Reports"[Publication Type] OR "Comment"[Publication Type] OR "congress"[Publication Type] OR "consensus development conference"[Publication Type] OR "consensus development conference, nih"[Publication Type] OR "dictionary"[Publication Type] OR "directory"[Publication Type] OR "editorial"[Publication Type] OR "Expression of Concern"[Publication Type] OR "Guideline"[Publication Type] OR "interactive tutorial"[Publication Type] OR "interview"[Publication Type] OR "lecture"[Publication Type] OR "legal case"[Publication Type] OR "legislation"[Publication Type] OR "letter"[Publication Type] OR "news"[Publication Type] OR "newspaper article"[Publication Type] OR "overall"[Publication Type] OR "patient education handout"[Publication Type] OR "periodical index"[Publication Type] OR "personal narrative"[Publication Type] OR "portrait"[Publication Type] OR "Review"[Publication Type] OR "Scientific Integrity Review"[Publication Type] OR "hascommenton"[All Fields] OR "Cartoons as Topic"[Mesh] OR "Review Literature as Topic"[Mesh] OR "case report*"[tiab] OR "case series"[tiab] OR "integrative research review*"[tiab] OR "integrative review*"[tiab] OR "literature review"[tiab] OR "narrative review"[tiab] OR "research integration"[tiab] OR "scoping review"[tiab] OR ((methodologic*[tiab] OR quantitative*[tiab]) AND (overview*[tiab] OR review*[tiab] OR synthesis*[tiab]))))

Embase: ((((('sciatica'/exp OR sciatic:ti,ab OR (('radicular pain'/exp OR 'radiculopathy'/exp OR (("Radicular Pain" OR Radiculitides OR Radiculitis OR Radiculopath*):ti,ab) OR (('nerve root'/exp OR "Nerve Root":ti,ab) AND ('inflammation'/exp OR 'pain'/exp OR ((Ache* OR Aching OR Avulsion* OR Compress* OR Disorder* OR Entrap* OR Imping* OR Inflam* OR Irritat* OR Pinch* OR Trap*):ti,ab)))) AND ('low back pain'/exp OR 'lumbar vertebra'/exp OR 'lumbosacral region'/exp OR ((Low OR Lower OR Lumbar OR Lumbosacral OR "Lumbo-sacral"):ti,ab)))) AND ('decompression surgery'/de OR 'discectomy'/exp OR 'foraminotomy'/exp OR 'total disc replacement'/exp OR ('lumbar vertebra'/exp AND 'surgery'/lnk) OR ((Accutherm OR APLD OR "Artificial Disc Replacement*" OR "Artificial Disk Replacement*" OR "Disc Replacement Arthroplast*" OR Discectom* OR Disctrode OR Diskectom* OR Disktrode OR "Disk Replacement Arthroplast*" OR Facetectom* OR Fenestrat* OR Foraminotom* OR Laminectom* OR Laminoplast* OR Laminotom* OR Microdecompression* OR Microdiscectom* OR Microdiskectom* OR "Replacement Disc Arthroplast*" OR "Replacement Disk Arthroplast*" OR Sequestrectom* OR Spinecath OR "Total Disc Arthroplast*" OR "Total Disc Replacement*" OR "Total Disk Arthroplast*" OR "Total Disk Replacement*" OR Transdiscal):ti,ab) OR ((decompress* NEAR/2 (disc OR disk OR intradiscal OR lumb* OR surg*)):ti,ab) OR (((disc OR

disk OR intervertebral OR intradiscal OR percutaneous) NEAR/2 (annuloplast* OR arthroplast* OR biacuplast* OR electrothermal OR thermomodulation)):ti,ab) OR (((disc or disk) NEAR/2 (excis* OR remov* OR replac* OR resect* OR surg*)):ti,ab) OR ((lamina NEAR/2 (arthroplast* OR excis* OR remov* OR replac* OR resect* OR surg*)):ti,ab) OR ((lumb* NEAR/2 (arthroplast* OR excis* OR remov* OR replac* OR resect* OR surg*)):ti,ab) OR ((micro* NEAR2 (endoscop* OR laser OR surg*)):ti,ab) OR ((thermal NEAR/2 procedure*):ti,ab)) AND ([1-1-2016]/sd NOT [20-5-2022]/sd) AND ([english]/lim) AND ('randomized controlled trial'/exp OR placebo:ti,ab OR random*:ti,ab OR trial*:ti OR 'meta analysis'/exp OR 'meta analysis topic'/exp OR 'systematic review'/exp OR 'systematic review (topic)'/exp OR metaanalys*:ti OR meta-analys*:ti OR "meta analys*":ti OR "systematic review":ti OR trial*:ti OR (((meta OR systematic*) AND (overview* OR review* OR synthesis*)):ti))) NOT ('chromatography'/exp OR 'chromosome'/exp OR 'congenital'/exp OR 'genetic polymorphism'/exp OR 'genetics'/exp OR 'genome'/exp OR 'genomics'/exp OR 'genotype'/exp OR 'mass spectrometry'/exp OR 'microbiome'/exp OR 'mutation'/exp OR 'pharmacokinetics'/exp OR 'polymorphism'/exp OR assay*:ti,ab OR cell:ti,ab OR cells:ti,ab OR chromosome*:ti,ab OR genetic*:ti,ab OR genome*:ti,ab OR genomic*:ti,ab OR "in vitro":ti,ab OR microbiom*:ti,ab OR microbiota*:ti,ab OR mutat*:ti,ab OR pharmacokinetic*:ti,ab OR polymorphism*:ti,ab OR SNP:ti,ab OR spectrometry:ti,ab OR tissue*:ti,ab)) NOT (('animal'/exp OR animal*:ti,ab OR ape:ti,ab OR apes:ti,ab OR canine*:ti,ab OR cat:ti,ab OR cats:ti,ab OR chimpanzee*:ti,ab OR dog:ti,ab OR dogs:ti,ab OR feline*:ti,ab OR hamster*:ti,ab OR lamb*:ti,ab OR mice:ti,ab OR monkey*:ti,ab OR mouse:ti,ab OR murine:ti,ab OR pig:ti,ab OR pigs:ti,ab OR piglet*:ti,ab OR porcine:ti,ab OR primate*:ti,ab OR rabbit*:ti,ab OR rat:ti,ab OR rats:ti,ab OR rodent*:ti,ab OR sheep*:ti,ab OR swine:ti,ab) NOT ('human'/exp OR human*:ti,ab OR man:ti,ab OR men:ti,ab OR patient*:ti,ab OR woman:ti,ab OR women:ti,ab))) NOT (('abstract report'/exp OR 'animal experiment'/exp OR 'book'/exp OR 'case finding'/exp OR 'case report'/exp OR 'case study'/exp OR 'conference paper'/exp OR 'editorial'/exp OR 'feasibility study'/exp OR 'in vitro study'/exp) AND 'letter'/exp OR 'note'/exp OR 'practice guideline'/exp OR 'review'/exp OR 'veterinary clinical trial'/exp OR 'veterinary study'/exp OR [conference abstract]/lim OR [conference paper]/lim OR [conference review]/lim OR [editorial]/lim OR [letter]/lim OR [note]/lim OR [short survey]/lim OR 'case report*':ti,ab OR 'case series':ti,ab OR 'integrative research review*':ti,ab OR 'integrative review*':ti,ab OR 'literature review':ti,ab OR 'narrative review':ti,ab OR 'research integration':ti,ab OR 'scoping review':ti,ab OR ((integrative NEAR/5 research NEAR/5 review*):ti,ab) OR ((methodologic* NEAR/5 overview*):ti,ab) OR ((methodologic* NEAR/5 review*):ti,ab) OR ((quantitativ* NEAR/5 overview*):ti,ab) OR ((quantitativ* NEAR/5 review*):ti,ab) OR ((quantitativ* NEAR/5 synthesi*):ti,ab) OR ((research NEAR/5 integration):ti,ab)))

Cochrane:

ID	Search
#1	MeSH descriptor: [Sciatica] explode all trees
#2	Sciatic*:ti,ab
#3	MeSH descriptor: [Radiculopathy] explode all trees
#4	(("Radicular Pain" OR Radiculitides OR Radiculitis OR Radiculopath*):ti,ab)
#5	#3 OR #4
#6	MeSH descriptor: [Spinal Nerve Roots] explode all trees
#7	"Nerve Root":ti,ab
#8	#6 OR #7
#9	MeSH descriptor: [Inflammation] explode all trees
#10	MeSH descriptor: [Pain] explode all trees
#11	((Ache* OR Aching OR Avulsion* OR Compress* OR Disorder* OR Entrap* OR Imping* OR Inflam* OR Irritat* OR Pinch* OR Trap*):ti,ab)
#12	OR/#9-#11

#13 #5 OR #8 OR #12

#14 (MeSH descriptor: [Low Back Pain] explode all trees

#15 MeSH descriptor: [Lumbar Vertebrae] explode all trees

#16 MeSH descriptor: [Lumbosacral Region] explode all trees

#17 ((Low OR Lower OR Lumbar OR Lumbosacral OR "Lumbo-sacral"):ti,ab)

#18 OR/#14-#17

#19 #13 AND #18

#20 #1 OR #2 OR #19

#21 MeSH descriptor: [Decompression, Surgical] explode all trees

#22 MeSH descriptor: [Diskectomy] explode all trees

#23 MeSH descriptor: [Foraminotomy] explode all trees

#24 MeSH descriptor: [Laminectomy] explode all trees

#25 MeSH descriptor: [Lumbar Vertebrae] explode all trees and with qualifier(s): [surgery - SU]

#26 MeSH descriptor: [Total Disc Replacement] explode all trees

#27 ((Accutherm OR APLD OR "Artificial Disc Replacement*" OR "Artificial Disk Replacement*" OR "Disc Replacement Arthroplast*" OR Discectom* OR Disctrode OR Diskectom* OR Disktrode OR "Disk Replacement Arthroplast*" OR Facetectom* OR Fenestrat* OR Foraminotom* OR Laminectom* OR Laminoplast* OR Laminotom* OR Microdecompression* OR Microdiscectom* OR Microdiskectom* OR "Replacement Disc Arthroplast*" OR "Replacement Disk Arthroplast*" OR Sequestrectom* OR Spinecath OR "Total Disc Arthroplast*" OR "Total Disc Replacement*" OR "Total Disk Arthroplast*" OR "Total Disk Replacement*" OR Transdiscal):ti,ab) OR ((decompress* NEAR/2 (disc OR disk OR intradiscal OR lumb* OR surg*)):ti,ab) OR (((disc OR disk OR intervertebral OR intradiscal OR percutaneous) NEAR/2 (annuloplast* OR arthroplast* OR biacuplast* OR electrothermal OR thermomodulation)):ti,ab) OR (((disc or disk) NEAR/2 (excis* OR remov* OR replac* OR resect* OR surg*)):ti,ab) OR ((lamina NEAR/2 (arthroplast* OR excis* OR remov* OR replac* OR resect* OR surg*)):ti,ab) OR ((lumb* NEAR/2 (arthroplast* OR excis* OR remov* OR replac* OR resect* OR surg*)):ti,ab) OR ((micro* NEAR2 (endoscop* OR laser OR surg*)):ti,ab) OR ((thermal NEAR/2 procedure*):ti,ab)

#28 OR/#21-#27

#29 #20 AND #28

#30 MeSH descriptor: [Biomarkers] explode all trees

#31 MeSH descriptor: [Chromatography] explode all trees

#32 MeSH descriptor: [Chromosomes] explode all trees

#33 MeSH descriptor: [Echocardiography] explode all trees

#34 MeSH descriptor: [Genetics] in all MeSH products

#35 MeSH descriptor: [Genome] in all MeSH products

#36 MeSH descriptor: [Genotype] explode all trees

#37 MeSH descriptor: [Incidence] explode all trees

#38 MeSH descriptor: [Mass Spectrometry] explode all trees

#39 MeSH descriptor: [Microbiota] explode all trees

#40 MeSH descriptor: [Mutation] explode all trees

#41 MeSH descriptor: [Pharmacokinetics] explode all trees

#42 MeSH descriptor: [Polymorphism, Genetic] explode all trees

#43 Any MeSH descriptor in all MeSH products and with qualifier(s): [congenital - CN]

#44 Any MeSH descriptor in all MeSH products and with qualifier(s): [genetics - GE]

#45 Any MeSH descriptor in all MeSH products and with qualifier(s): [pharmacokinetics - PK]

#46 ((assay* OR biomarker* OR cell OR cells OR chromosome* OR genetic* OR genome* OR genomic* OR "in vitro" OR microbiom* OR microbiota* OR mutat* OR pharmacokinetic* OR polymorphism* OR SNP OR spectrometry OR tissue*):ti,ab)

#47 OR/#30-#46

#48 #29 NOT #47

#49 #48 AND publication date between January 2016 and May 2022

Eligibility criteria:

- Inclusion criteria: ***A) Population:*** People aged 16 or above with sciatica; Populations with neurogenic claudication causing leg pain will be included. ***B) Intervention:*** Spinal decompression (Laminectomy, Discectomy, Facetectomy, Foraminotomy, Fenestration, Spinal decompression, Sequestration, Laminotomy) or usual care. ***C) Comparison:*** All interventions will be compared with each other. ***D) Study Type:*** Randomized controlled trials, systematic reviews, and non-randomized studies.
- Exclusion criteria: None.

PRISMA flow diagram:

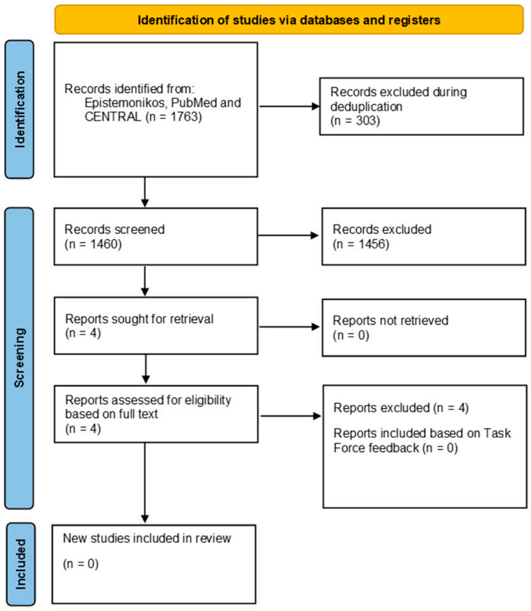

Question 10: Should radiofrequency denervation for facet joint pain versus placebo or usual care, or versus other treatments, be used in patients with non-specific LBP?

Databases searched: PubMed, Embase, and Cochrane

Search period: 01 January 2016 to 19 May 2022 (PubMed and Embase); January 2016 to May 2022 (Cochrane)

Search strategy used:

PubMed: ((((("Low Back Pain"[Mesh] OR "Sciatica"[Mesh] OR "Low Backache*"[tiab] OR "Lower Backache*"[tiab] OR Lumbago[tiab] OR Sciatic* [tiab] OR (("Lumbar Vertebrae"[Mesh] OR "Lumbosacral Region"[Mesh] OR "Low Back"[tiab] OR "Lower Back"[tiab] OR Lumbar[tiab] OR Lumbosacral[tiab] OR "Lumbo-sacral"[tiab]) AND ("Pain"[Mesh] OR Ache*[tiab] OR Aching[tiab] OR Pain*[tiab])) OR (("Radiculopathy"[Mesh] OR "Radicular Pain"[tiab] OR Radiculitides[tiab] OR Radiculitis[tiab] OR Radiculopath*[tiab] OR (("Spinal Nerve Roots"[Mesh] OR "Nerve Root"[tiab]) AND ("Inflammation"[Mesh] OR "Pain"[Mesh] OR Ache*[tiab] OR Aching[tiab] OR Avulsion*[tiab] OR Compress*[tiab] OR Disorder*[tiab] OR Entrap*[tiab] OR Imping*[tiab] OR Inflam*[tiab] OR Irritat*[tiab] OR Pinch*[tiab] OR Trap*[tiab]))) AND ("Low Back Pain"[Mesh] OR "Lumbar Vertebrae"[Mesh] OR "Lumbosacral Region"[Mesh] OR Low[tiab] OR Lower[tiab] OR Lumbar[tiab] OR Lumbosacral[tiab] OR "Lumbo-sacral"[tiab]))) AND ("Ablation Techniques"[Mesh] OR "Catheter Ablation"[Mesh] OR "Denervation"[Mesh] OR "Pulsed Radiofrequency Treatment"[Mesh] OR "Rhizotomy"[Mesh] OR Ablat*[tiab] OR Chemodenervation[tiab] OR Cryoablat*[tiab] OR Cryosurg*[tiab] OR Denervat*[tiab] OR Neurectom*[tiab] OR Neuroly*[tiab] OR Neurotom*[tiab] OR "Radio Frequency"[tiab] OR Radiofrequency[tiab] OR Rhizotom*[tiab] OR Rhizoly*[tiab] OR (Catheter*[tiab] AND (Cool*[tiab] OR Cold[tiab] OR Cryo*[tiab] OR Electric*[tiab] OR Percutaneous[tiab] OR Transvenous[tiab]))) AND ("Zygapophyseal Joint"[Mesh] OR Apophyseal*[tiab] OR Facet*[tiab] OR "Z Joint*"[tiab] OR Zygapophyseal*[tiab] OR Zygapophysial*[tiab] OR (Media*[tiab] AND (Branch*[tiab] OR Nerve*[tiab]))) AND (2016/1/1:2022/5/19[pdat]) AND (english[Filter]) AND ("Randomized Controlled Trial"[Publication Type] OR placebo[tiab] OR random*[tiab] OR trial*[ti] OR "Meta-Analysis"[Publication Type] OR "Systematic Review"[Publication Type] OR "Systematic Reviews as Topic"[Mesh] OR metaanalys*[ti] OR meta-analys*[ti] OR "meta analys*"[ti] OR "systematic review"[ti] OR trial*[ti] OR ((meta[ti] OR systematic*[ti]) AND (overview*[ti] OR review*[ti] OR synthesis*[ti])))) NOT ("Chromatography"[Mesh] OR "Chromosomes"[Mesh] OR "Genetics"[Mesh] OR "Genome"[Mesh] OR "Genomics"[Mesh] OR "Genotype"[Mesh] OR "Mass Spectrometry"[Mesh] OR "Microbiota"[Mesh] OR "Mutation"[Mesh] OR "Pharmacokinetics"[Mesh] OR "Polymorphism, Genetic"[Mesh] OR "congenital" [Subheading] OR "genetics"[Subheading] OR "pharmacokinetics"[Subheading] OR assay*[tiab] OR cell[tiab] OR cells[tiab] OR chromosome*[tiab] OR genetic*[tiab] OR genome*[tiab] OR genomic*[tiab] OR "in vitro"[tiab] OR microbiom*[tiab] OR microbiota*[tiab] OR mutat*[tiab] OR pharmacokinetic*[tiab] OR polymorphism*[tiab] OR SNP[tiab] OR spectrometry[tiab] OR tissue*[tiab])) NOT (("Animals"[Mesh] OR animal*[tiab] OR ape[tiab] OR apes[tiab] OR canine*[tiab] OR cat[tiab] OR cats[tiab] OR chimpanzee*[tiab] OR dog[tiab] OR dogs[tiab] OR feline*[tiab] OR hamster*[tiab] OR lamb*[tiab] OR mice[tiab] OR monkey*[tiab] OR mouse[tiab] OR murine[tiab] OR pig[tiab] OR pigs[tiab] OR piglet*[tiab] OR porcine[tiab] OR primate*[tiab] OR rabbit*[tiab] OR rat[tiab] OR rats[tiab] OR rodent*[tiab] OR sheep*[tiab] OR swine[tiab]) NOT ("Humans"[Mesh] OR human*[tiab] OR man[tiab] OR men[tiab] OR patient*[tiab] OR woman[tiab] OR women[tiab]))) NOT ("address"[Publication Type] OR "autobiography"[Publication Type] OR "bibliography"[Publication Type] OR "biography"[Publication Type] OR "Book Illustrations"[Publication Type] OR "Case Reports"[Publication Type] OR "Comment"[Publication Type] OR "congress"[Publication Type] OR "consensus development conference"[Publication Type] OR "consensus development conference, nih"[Publication Type] OR "dictionary"[Publication Type] OR "directory"[Publication Type] OR "editorial"[Publication Type] OR "Expression of Concern"[Publication Type] OR "Guideline"[Publication Type] OR "interactive tutorial"[Publication Type] OR "interview"[Publication Type] OR "lecture"[Publication Type] OR "legal case"[Publication Type] OR "legislation"[Publication Type] OR "letter"[Publication Type] OR "news"[Publication Type] OR "newspaper article"[Publication Type] OR "overall"[Publication Type] OR "patient education handout"[Publication Type] OR "periodical index"[Publication Type] OR "personal narrative"[Publication Type] OR "portrait"[Publication Type] OR "Review"[Publication Type] OR "Scientific Integrity Review"[Publication Type] OR "hascommenton"[All Fields] OR "Cartoons as Topic"[Mesh] OR "Review Literature as Topic"[Mesh] OR "case report*"[tiab] OR "case series"[tiab] OR "integrative research review*"[tiab] OR "integrative review*"[tiab] OR "literature review"[tiab] OR "narrative review"[tiab] OR "research integration"[tiab] OR "scoping review"[tiab] OR ((methodologic*[tiab] OR quantitative*[tiab]) AND (overview*[tiab] OR review*[tiab] OR synthesis*[tiab]))))

Embase: (((((('low back pain'/exp OR 'sciatica'/exp OR (("Low Backache*" OR "Lower Backache*" OR Lumbago OR Sciatic*):ti,ab) OR (('lumbar vertebra'/exp OR 'lumbosacral region'/exp OR (("Low Back" OR "Lower Back" OR Lumbar OR Lumbosacral OR "Lumbo-sacral"):ti,ab)) AND ('pain'/exp OR ((Ache* OR Aching OR Pain*):ti,ab))) OR (('radicular pain'/exp OR 'radiculopathy'/exp OR (("Radicular Pain" OR Radiculitides OR Radiculitis OR Radiculopath*):ti,ab) OR (('nerve root'/exp OR "Nerve Root":ti,ab) AND ('inflammation'/exp OR 'pain'/exp OR ((Ache* OR Aching OR Avulsion* OR Compress* OR Disorder* OR Entrap* OR Imping* OR Inflam* OR Irritat* OR Pinch* OR Trap*):ti,ab)))) AND ('low back pain'/exp OR 'lumbar vertebra'/exp OR 'lumbosacral region'/exp OR ((Low OR Lower OR Lumbar OR Lumbosacral OR "Lumbo-sacral"):ti,ab)))) AND ('ablation therapy'/exp OR 'catheter ablation'/exp OR 'cryoablation'/exp OR 'denervation'/exp OR 'neurolysis'/exp OR 'pulsed radiofrequency treatment'/exp OR 'radiofrequency ablation device'/exp OR 'radiofrequency ablation'/exp OR 'rhizotomy'/exp OR ((Ablat* OR Chemodenervation OR Cryoablat* OR Cryosurg* OR Denervat* OR Neurectom* OR Neuroly* OR Neurotom* OR "Radio Frequency" OR Radiofrequency OR Rhizotom* OR Rhizoly*):ti,ab) OR ((Catheter* AND (Cool* OR Cold OR Cryo* OR Electric* OR Percutaneous OR Transvenous)):ti,ab)) AND ('zygapophyseal joint'/exp OR ((Apophyseal* OR Facet* OR "Z Joint*" OR Zygapophyseal* OR Zygapophysial*):ti,ab) OR ((Media* AND (Branch* OR Nerve*)):ti,ab)) AND ([1-1-2016]/sd NOT [20-5-2022]/sd) AND ([english]/lim) AND ('randomized controlled trial'/exp OR placebo:ti,ab OR random*:ti,ab OR trial*:ti OR 'meta analysis'/exp OR 'meta analysis topic'/exp OR 'systematic review'/exp OR 'systematic review (topic)'/exp OR metaanalys*:ti OR meta-analys*:ti OR "meta analys*":ti OR "systematic review":ti OR trial*:ti OR (((meta OR systematic*) AND (overview* OR review* OR synthesis*)):ti))) NOT ('chromatography'/exp OR 'chromosome'/exp OR 'congenital'/exp OR 'genetic polymorphism'/exp OR 'genetics'/exp OR 'genome'/exp OR 'genomics'/exp OR 'genotype'/exp OR 'mass spectrometry'/exp OR 'microbiome'/exp OR 'mutation'/exp OR 'pharmacokinetics'/exp OR 'polymorphism'/exp OR assay*:ti,ab OR cell:ti,ab OR cells:ti,ab OR chromosome*:ti,ab OR genetic*:ti,ab OR genome*:ti,ab OR genomic*:ti,ab OR "in vitro":ti,ab OR microbiom*:ti,ab OR microbiota*:ti,ab OR mutat*:ti,ab OR pharmacokinetic*:ti,ab OR polymorphism*:ti,ab OR SNP:ti,ab OR spectrometry:ti,ab OR tissue*:ti,ab)) NOT (('animal'/exp OR animal*:ti,ab OR ape:ti,ab OR apes:ti,ab OR canine*:ti,ab OR cat:ti,ab OR cats:ti,ab OR chimpanzee*:ti,ab OR dog:ti,ab OR dogs:ti,ab OR feline*:ti,ab OR hamster*:ti,ab OR lamb*:ti,ab OR mice:ti,ab OR monkey*:ti,ab OR mouse:ti,ab OR murine:ti,ab OR pig:ti,ab OR pigs:ti,ab OR piglet*:ti,ab OR porcine:ti,ab OR primate*:ti,ab OR rabbit*:ti,ab OR rat:ti,ab OR rats:ti,ab OR rodent*:ti,ab OR sheep*:ti,ab OR swine:ti,ab) NOT ('human'/exp OR human*:ti,ab OR man:ti,ab OR men:ti,ab OR patient*:ti,ab OR woman:ti,ab OR women:ti,ab))) NOT (('abstract report'/exp OR 'animal experiment'/exp OR 'book'/exp OR 'case finding'/exp OR 'case report'/exp OR 'case study'/exp OR 'conference paper'/exp OR 'editorial'/exp OR 'feasibility study'/exp OR 'in vitro study'/exp) AND 'letter'/exp OR 'note'/exp OR 'practice guideline'/exp OR 'review'/exp OR 'veterinary clinical trial'/exp OR 'veterinary study'/exp OR [conference abstract]/lim OR [conference paper]/lim OR [conference review]/lim OR [editorial]/lim OR [letter]/lim OR [note]/lim OR [short survey]/lim OR 'case report*':ti,ab OR 'case series':ti,ab OR 'integrative research review*':ti,ab OR 'integrative review*':ti,ab OR 'literature review':ti,ab OR 'narrative review':ti,ab OR 'research integration':ti,ab OR 'scoping review':ti,ab OR ((integrative NEAR/5 research NEAR/5 review*):ti,ab) OR ((methodologic* NEAR/5 overview*):ti,ab) OR ((methodologic* NEAR/5 review*):ti,ab) OR ((quantitativ* NEAR/5 overview*):ti,ab) OR ((quantitativ* NEAR/5 review*):ti,ab) OR ((quantitativ* NEAR/5 synthesi*):ti,ab) OR ((research NEAR/5 integration):ti,ab)))

Cochrane:

**ID**	**Search**
#1	MeSH descriptor: [Low Back Pain] explode all trees
#2	MeSH descriptor: [Sciatica] explode all trees
#3	(("Low Backache*" OR "Lower Backache*" OR Lumbago OR Sciatic*):ti,ab)
#4	MeSH descriptor: [Lumbar Vertebrae] explode all trees
#5	MeSH descriptor: [Lumbosacral Region] explode all trees

#6 (("Low Back" OR "Lower Back" OR Lumbar OR Lumbosacral OR "Lumbo-sacral"):ti,ab)

#7 OR/#4-#6

#8 MeSH descriptor: [Pain] explode all trees

#9 ((Ache* OR Aching OR Pain*):ti,ab)

#10 #8 OR #9

#11 #7 AND #10

#12 MeSH descriptor: [Radiculopathy] explode all trees

#13 (("Radicular Pain" OR Radiculitides OR Radiculitis OR Radiculopath*):ti,ab)

#14 #12 OR #13

#15 MeSH descriptor: [Spinal Nerve Roots] explode all trees

#16 "Nerve Root":ti,ab

#17 #15 OR #16

#18 MeSH descriptor: [Inflammation] explode all trees

#19 MeSH descriptor: [Pain] explode all trees

#20 ((Ache* OR Aching OR Avulsion* OR Compress* OR Disorder* OR Entrap* OR Imping* OR Inflam* OR Irritat* OR Pinch* OR Trap*):ti,ab)

#21 OR/#18-#20

#22 #14 OR #17 OR #20

#23 MeSH descriptor: [Low Back Pain] explode all trees

#24 MeSH descriptor: [Lumbar Vertebrae] explode all trees

#25 MeSH descriptor: [Lumbosacral Region] explode all trees

#26 ((Low OR Lower OR Lumbar OR Lumbosacral OR "Lumbo-sacral"):ti,ab)

#27 OR/#23-#26

#28 #22 AND #27

#29 #1 OR #2 OR #3 OR #11 OR #28

#30 MeSH descriptor: [Ablation Techniques] explode all trees

#31 MeSH descriptor: [Catheter Ablation] explode all trees

#32 MeSH descriptor: [Denervation] explode all trees

#33 MeSH descriptor: [Pulsed Radiofrequency Treatment] explode all trees

#34 MeSH descriptor: [Rhizotomy] explode all trees

#35 ((Ablat* OR Chemodenervation OR Cryoablat* OR Cryosurg* OR Denervat* OR Neurectom* OR Neuroly* OR Neurotom* OR "Radio Frequency" OR Radiofrequency OR Rhizotom* OR Rhizoly*):ti,ab) OR ((Catheter* AND (Cool* OR Cold OR Cryo* OR Electric* OR Percutaneous OR Transvenous)):ti,ab)

#36 OR/#30-#35

#37 MeSH descriptor: [Zygapophyseal Joint] explode all trees

#38 ((Apophyseal* OR Facet* OR "Z Joint*" OR Zygapophyseal* OR Zygapophysial*):ti,ab) OR ((Media* AND (Branch* OR Nerve*)):ti,ab)

#39 #37 OR #38

#40 #29 AND #36 AND #39

#41 MeSH descriptor: [Biomarkers] explode all trees

#42 MeSH descriptor: [Chromatography] explode all trees

#43 MeSH descriptor: [Chromosomes] explode all trees

#44 MeSH descriptor: [Echocardiography] explode all trees

#45 MeSH descriptor: [Genetics] in all MeSH products

#46 MeSH descriptor: [Genome] in all MeSH products

#47 MeSH descriptor: [Genotype] explode all trees

#48 MeSH descriptor: [Incidence] explode all trees

#49 MeSH descriptor: [Mass Spectrometry] explode all trees

#50 MeSH descriptor: [Microbiota] explode all trees

#51 MeSH descriptor: [Mutation] explode all trees

#52 MeSH descriptor: [Pharmacokinetics] explode all trees

#53 MeSH descriptor: [Polymorphism, Genetic] explode all trees

#54 Any MeSH descriptor in all MeSH products and with qualifier(s): [congenital - CN]

#55 Any MeSH descriptor in all MeSH products and with qualifier(s): [genetics - GE]

#56 Any MeSH descriptor in all MeSH products and with qualifier(s): [pharmacokinetics - PK]

#57 ((assay* OR biomarker* OR cell OR cells OR chromosome* OR genetic* OR genome* OR genomic* OR "in vitro" OR microbiom* OR microbiota* OR mutat* OR pharmacokinetic* OR polymorphism* OR SNP OR spectrometry OR tissue*):ti,ab)

#58 OR/#41-#57

#59 #40 NOT #58

#60 #59 AND publication date between January 2016 to May 2022

Eligibility criteria:

- Inclusion criteria: ***A) Population:*** People aged 16 or above with non-specific low back pain with or without sciatica; People aged 16 or above with sciatica. ***B) Intervention:*** Radiofrequency denervation of facet joint medial branch. ***C) Comparison:*** Placebo/Sham/Attention control or usual care/waiting list. ***D) Study Type:*** Randomized controlled trials, systematic reviews, non-randomized studies.
- Exclusion criteria: None.

PRISMA flow diagram:

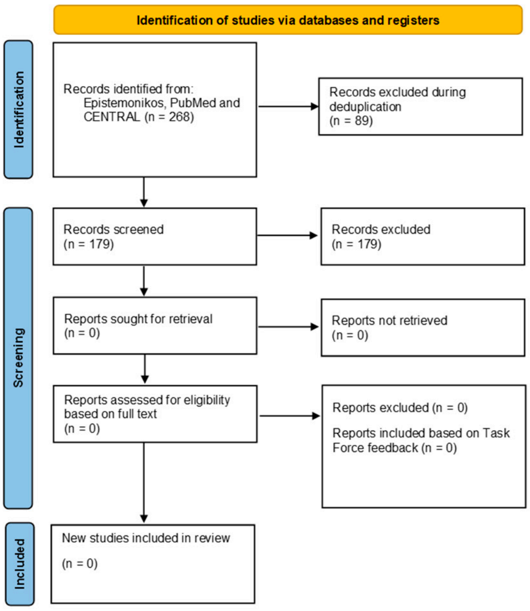

Question 11: Should pain neuroscience education versus no pain neuroscience education be used to reduce disability, pain and recurrence in patients with chronic non-specific LBP?

Databases searched: PubMed, Embase, and Cochrane

Search period: 01 January 2016 to 19 May 2022 (PubMed and Embase); January 2016 to May 2022 (Cochrane)

Search strategy used:

PubMed: ((((("Low Back Pain"[Mesh] OR "Sciatica"[Mesh] OR "Low Backache*"[tiab] OR "Lower Backache*"[tiab] OR Lumbago[tiab] OR Sciatic* [tiab] OR (("Lumbar Vertebrae"[Mesh] OR "Lumbosacral Region"[Mesh] OR "Low Back"[tiab] OR "Lower Back"[tiab] OR Lumbar[tiab] OR Lumbosacral[tiab] OR "Lumbo-sacral"[tiab]) AND ("Pain"[Mesh] OR Ache*[tiab] OR Aching[tiab] OR Pain*[tiab])) OR (("Radiculopathy"[Mesh] OR "Radicular Pain"[tiab] OR Radiculitides[tiab] OR Radiculitis[tiab] OR Radiculopath*[tiab] OR (("Spinal Nerve Roots"[Mesh] OR "Nerve Root"[tiab]) AND ("Inflammation"[Mesh] OR "Pain"[Mesh] OR Ache*[tiab] OR Aching[tiab] OR Avulsion*[tiab] OR Compress*[tiab] OR Disorder*[tiab] OR Entrap*[tiab] OR Imping*[tiab] OR Inflam*[tiab] OR Irritat*[tiab] OR Pinch*[tiab] OR Trap*[tiab]))) AND ("Low Back Pain"[Mesh] OR "Lumbar Vertebrae"[Mesh] OR "Lumbosacral Region"[Mesh] OR Low[tiab] OR Lower[tiab] OR Lumbar[tiab] OR Lumbosacral[tiab] OR "Lumbo-sacral"[tiab]))) AND ("Educational Status"[Mesh] OR "Educational Technology"[Mesh] OR "Patient Education as Topic"[Mesh] OR "Health Education"[Mesh] OR "Neurosciences/education"[Mesh] OR "education"[Subheading] OR "Neuroscience Continuing Education"[tiab] OR "Neuroscience Education"[tiab] OR ((Education*[ti] OR Instruction*[ti]) AND (Activit*[tiab] OR Patient*[tiab] OR Status*[tiab] OR Technolog*[tiab]))) AND (2016/1/1:2022/5/19[pdat]) AND (english[Filter]) AND ("Cohort Studies"[Mesh] OR "cohort analys*"[tiab] OR "cohort stud*"[tiab] OR "concurrent stud*"[tiab] OR "incidence stud*"[tiab] OR "Randomized Controlled Trial"[Publication Type] OR placebo[tiab] OR random*[tiab] OR trial*[ti] OR "Meta-Analysis"[Publication Type] OR "Systematic Review"[Publication Type] OR "Systematic Reviews as Topic"[Mesh] OR metaanalys*[ti] OR meta-analys*[ti] OR "meta analys*"[ti] OR "systematic review"[ti] OR trial*[ti] OR ((meta[ti] OR systematic*[ti]) AND (overview*[ti] OR review*[ti] OR synthesis*[ti])))) NOT ("Chromatography"[Mesh] OR "Chromosomes"[Mesh] OR "Genetics"[Mesh] OR "Genome"[Mesh] OR "Genomics"[Mesh] OR "Genotype"[Mesh] OR "Mass Spectrometry"[Mesh] OR "Microbiota"[Mesh] OR "Mutation"[Mesh] OR "Pharmacokinetics"[Mesh] OR "Polymorphism, Genetic"[Mesh] OR "congenital" [Subheading] OR "genetics"[Subheading] OR "pharmacokinetics"[Subheading] OR assay*[tiab] OR cell[tiab] OR cells[tiab] OR chromosome*[tiab]

OR genetic*[tiab] OR genome*[tiab] OR genomic*[tiab] OR "in vitro"[tiab] OR microbiom*[tiab] OR microbiota*[tiab] OR mutat*[tiab] OR pharmacokinetic*[tiab] OR polymorphism*[tiab] OR SNP[tiab] OR spectrometry[tiab] OR tissue*[tiab])) NOT (("Animals"[Mesh] OR animal*[tiab] OR ape[tiab] OR apes[tiab] OR canine*[tiab] OR cat[tiab] OR cats[tiab] OR chimpanzee*[tiab] OR dog[tiab] OR dogs[tiab] OR feline*[tiab] OR hamster*[tiab] OR lamb*[tiab] OR mice[tiab] OR monkey*[tiab] OR mouse[tiab] OR murine[tiab] OR pig[tiab] OR pigs[tiab] OR piglet*[tiab] OR porcine[tiab] OR primate*[tiab] OR rabbit*[tiab] OR rat[tiab] OR rats[tiab] OR rodent*[tiab] OR sheep*[tiab] OR swine[tiab]) NOT ("Humans"[Mesh] OR human*[tiab] OR man[tiab] OR men[tiab] OR patient*[tiab] OR woman[tiab] OR women[tiab]))) NOT ("address"[Publication Type] OR "autobiography"[Publication Type] OR "bibliography"[Publication Type] OR "biography"[Publication Type] OR "Book Illustrations"[Publication Type] OR "Case Reports"[Publication Type] OR "Comment"[Publication Type] OR "congress"[Publication Type] OR "consensus development conference"[Publication Type] OR "consensus development conference, nih"[Publication Type] OR "dictionary"[Publication Type] OR "directory"[Publication Type] OR "editorial"[Publication Type] OR "Expression of Concern"[Publication Type] OR "Guideline"[Publication Type] OR "interactive tutorial"[Publication Type] OR "interview"[Publication Type] OR "lecture"[Publication Type] OR "legal case"[Publication Type] OR "legislation"[Publication Type] OR "letter"[Publication Type] OR "news"[Publication Type] OR "newspaper article"[Publication Type] OR "overall"[Publication Type] OR "patient education handout"[Publication Type] OR "periodical index"[Publication Type] OR "personal narrative"[Publication Type] OR "portrait"[Publication Type] OR "Review"[Publication Type] OR "Scientific Integrity Review"[Publication Type] OR "hascommenton"[All Fields] OR "Cartoons as Topic"[Mesh] OR "Review Literature as Topic"[Mesh] OR "case report*"[tiab] OR "case series"[tiab] OR "integrative research review*"[tiab] OR "integrative review*"[tiab] OR "literature review"[tiab] OR "narrative review"[tiab] OR "research integration"[tiab] OR "scoping review"[tiab] OR ((methodologic*[tiab] OR quantitative*[tiab]) AND (overview*[tiab] OR review*[tiab] OR synthesis*[tiab]))))

Embase: ((((('low back pain'/exp OR 'sciatica'/exp OR (("Low Backache*" OR "Lower Backache*" OR Lumbago OR Sciatic*):ti,ab) OR (('lumbar vertebra'/exp OR 'lumbosacral region'/exp OR (("Low Back" OR "Lower Back" OR Lumbar OR Lumbosacral OR "Lumbo-sacral"):ti,ab)) AND ('pain'/exp OR ((Ache* OR Aching OR Pain*):ti,ab))) OR (('radicular pain'/exp OR 'radiculopathy'/exp OR (("Radicular Pain" OR Radiculitides OR Radiculitis OR Radiculopath*):ti,ab) OR (('nerve root'/exp OR "Nerve Root":ti,ab) AND ('inflammation'/exp OR 'pain'/exp OR ((Ache* OR Aching OR Avulsion* OR Compress* OR Disorder* OR Entrap* OR Imping* OR Inflam* OR Irritat* OR Pinch* OR Trap*):ti,ab)))) AND ('low back pain'/exp OR 'lumbar vertebra'/exp OR 'lumbosacral region'/exp OR ((Low OR Lower OR Lumbar OR Lumbosacral OR "Lumbo-sacral"):ti,ab)))) AND ('educational status'/exp OR 'educational technology'/exp OR 'health education'/exp OR 'patient education'/exp OR "Neuroscience Continuing Education":ti,ab OR "Neuroscience Education":ti,ab OR ((Education*:ti OR Instruction*:ti) AND (Activit*:ti,ab OR Patient*:ti,ab OR Status*:ti,ab OR Technolog*:ti,ab))) AND ([1-1-2016]/sd NOT [20-5-2022]/sd) AND ([english]/lim) AND ('cohort analysis'/exp OR "cohort analys*":ti,ab OR "cohort stud*":ti,ab OR "concurrent stud*":ti,ab OR "incidence stud*":ti,ab OR 'randomized controlled trial'/exp OR placebo:ti,ab OR random*:ti,ab OR trial*:ti OR 'meta analysis'/exp OR 'meta analysis topic'/exp OR 'systematic review'/exp OR 'systematic review (topic)'/exp OR metaanalys*:ti OR meta-analys*:ti OR "meta analys*":ti OR "systematic review":ti OR trial*:ti OR (((meta OR systematic*) AND (overview* OR review* OR synthesis*)):ti))) NOT ('chromatography'/exp OR 'chromosome'/exp OR 'congenital'/exp OR 'genetic polymorphism'/exp OR 'genetics'/exp OR 'genome'/exp OR 'genomics'/exp OR 'genotype'/exp OR 'mass spectrometry'/exp OR 'microbiome'/exp OR 'mutation'/exp OR 'pharmacokinetics'/exp OR 'polymorphism'/exp OR assay*:ti,ab OR cell:ti,ab OR cells:ti,ab OR chromosome*:ti,ab OR genetic*:ti,ab OR genome*:ti,ab OR genomic*:ti,ab OR "in vitro":ti,ab OR microbiom*:ti,ab OR microbiota*:ti,ab OR mutat*:ti,ab OR pharmacokinetic*:ti,ab OR polymorphism*:ti,ab OR SNP:ti,ab OR spectrometry:ti,ab OR tissue*:ti,ab)) NOT (('animal'/exp OR animal*:ti,ab OR ape:ti,ab OR apes:ti,ab OR canine*:ti,ab OR cat:ti,ab OR cats:ti,ab OR chimpanzee*:ti,ab OR dog:ti,ab OR dogs:ti,ab OR feline*:ti,ab OR hamster*:ti,ab OR lamb*:ti,ab OR mice:ti,ab OR monkey*:ti,ab OR mouse:ti,ab OR murine:ti,ab OR pig:ti,ab OR pigs:ti,ab OR piglet*:ti,ab OR porcine:ti,ab OR primate*:ti,ab OR rabbit*:ti,ab OR rat:ti,ab

OR rats:ti,ab OR rodent*:ti,ab OR sheep*:ti,ab OR swine:ti,ab) NOT ('human'/exp OR human*:ti,ab OR man:ti,ab OR men:ti,ab OR patient*:ti,ab OR woman:ti,ab OR women:ti,ab))) NOT (('abstract report'/exp OR 'animal experiment'/exp OR 'book'/exp OR 'case finding'/exp OR 'case report'/exp OR 'case study'/exp OR 'conference paper'/exp OR 'editorial'/exp OR 'feasibility study'/exp OR 'in vitro study'/exp) AND 'letter'/exp OR 'note'/exp OR 'practice guideline'/exp OR 'review'/exp OR 'veterinary clinical trial'/exp OR 'veterinary study'/exp OR [conference abstract]/lim OR [conference paper]/lim OR [conference review]/lim OR [editorial]/lim OR [letter]/lim OR [note]/lim OR [short survey]/lim OR 'case report*':ti,ab OR 'case series':ti,ab OR 'integrative research review*':ti,ab OR 'integrative review*':ti,ab OR 'literature review':ti,ab OR 'narrative review':ti,ab OR 'research integration':ti,ab OR 'scoping review':ti,ab OR ((integrative NEAR/5 research NEAR/5 review*):ti,ab) OR ((methodologic* NEAR/5 overview*):ti,ab) OR ((methodologic* NEAR/5 review*):ti,ab) OR ((quantitativ* NEAR/5 overview*):ti,ab) OR ((quantitativ* NEAR/5 review*):ti,ab) OR ((quantitativ* NEAR/5 synthesi*):ti,ab) OR ((research NEAR/5 integration):ti,ab)))

Cochrane:

**ID**	**Search**
#1	MeSH descriptor: [Low Back Pain] explode all trees
#2	MeSH descriptor: [Sciatica] explode all trees
#3	(("Low Backache*" OR "Lower Backache*" OR Lumbago OR Sciatic*):ti,ab)
#4	MeSH descriptor: [Lumbar Vertebrae] explode all trees
#5	MeSH descriptor: [Lumbosacral Region] explode all trees
#6	(("Low Back" OR "Lower Back" OR Lumbar OR Lumbosacral OR "Lumbo-sacral"):ti,ab)
#7	OR/#4-#6
#8	MeSH descriptor: [Pain] explode all trees
#9	((Ache* OR Aching OR Pain*):ti,ab)
#10	#8 OR #9
#11	#7 AND #10
#12	MeSH descriptor: [Radiculopathy] explode all trees
#13	(("Radicular Pain" OR Radiculitides OR Radiculitis OR Radiculopath*):ti,ab)
#14	#12 OR #13
#15	MeSH descriptor: [Spinal Nerve Roots] explode all trees
#16	"Nerve Root":ti,ab
#17	#15 OR #16
#18	MeSH descriptor: [Inflammation] explode all trees
#19	MeSH descriptor: [Pain] explode all trees
#20	((Ache* OR Aching OR Avulsion* OR Compress* OR Disorder* OR Entrap* OR Imping* OR Inflam* OR Irritat* OR Pinch* OR Trap*):ti,ab)
#21	OR/#18-#20
#22	#14 OR #17 OR #20
#23	MeSH descriptor: [Low Back Pain] explode all trees
#24	MeSH descriptor: [Lumbar Vertebrae] explode all trees
#25	MeSH descriptor: [Lumbosacral Region] explode all trees

#26 ((Low OR Lower OR Lumbar OR Lumbosacral OR "Lumbo-sacral"):ti,ab)

#27 OR/#23-#26

#28 #22 AND #27

#29 #1 OR #2 OR #3 OR #11 OR #28

#30 MeSH descriptor: [Educational Status] explode all trees

#31 MeSH descriptor: [Educational Technology] explode all trees

#32 MeSH descriptor: [Patient Education as Topic] explode all trees

#33 MeSH descriptor: [Health Educators] explode all trees

#34 MeSH descriptor: [Neurosciences] explode all trees and with qualifier(s): [education - ED]

#35 MeSH descriptor: [] explode all trees and with qualifier(s): [education - ED]

#36 "Neuroscience Continuing Education":ti,ab OR "Neuroscience Education":ti,ab OR ((Education*:ti OR Instruction*:ti) AND (Activit*:ti,ab OR Patient*:ti,ab OR Status*:ti,ab OR Technolog*:ti,ab))

#37 OR/#30-#36

#38 #29 AND #37

#39 MeSH descriptor: [Biomarkers] explode all trees

#40 MeSH descriptor: [Chromatography] explode all trees

#41 MeSH descriptor: [Chromosomes] explode all trees

#42 MeSH descriptor: [Echocardiography] explode all trees

#43 MeSH descriptor: [Genetics] in all MeSH products

#44 MeSH descriptor: [Genome] in all MeSH products

#45 MeSH descriptor: [Genotype] explode all trees

#46 MeSH descriptor: [Incidence] explode all trees

#47 MeSH descriptor: [Mass Spectrometry] explode all trees

#48 MeSH descriptor: [Microbiota] explode all trees

#49 MeSH descriptor: [Mutation] explode all trees

#50 MeSH descriptor: [Pharmacokinetics] explode all trees

#51 MeSH descriptor: [Polymorphism, Genetic] explode all trees

#52 Any MeSH descriptor in all MeSH products and with qualifier(s): [congenital - CN]

#53 Any MeSH descriptor in all MeSH products and with qualifier(s): [genetics - GE]

#54 Any MeSH descriptor in all MeSH products and with qualifier(s): [pharmacokinetics - PK]

#55 ((assay* OR biomarker* OR cell OR cells OR chromosome* OR genetic* OR genome* OR genomic* OR "in vitro" OR microbiom* OR microbiota* OR mutat* OR pharmacokinetic* OR polymorphism* OR SNP OR spectrometry OR tissue*):ti,ab)

#56 OR/#39-#55

#57 #38 NOT #56

#58 #57 AND publication date between January 2016 to May 2022, in Trials

Eligibility criteria:

- Inclusion criteria: ***A) Population:*** People aged 16 or above with non-specific low back pain with or without sciatica; People aged 16 or above with sciatica. ***B) Intervention:*** Pain neuroscience education (PNE) or therapeutic neuroscience education or "explain pain" was required to be a component of the experimental group. The PNE could be delivered in isolation or in combination with other forms of physiotherapy treatment: including exercise, manual therapy, acupuncture or dry needling. Since there is no standardization for the delivery of PNE, all forms of delivery will be considered: such as group instruction, individual explanation, the use of presentations, books or leaflets to supplement the explanations. ***C) Comparison:*** All control groups were considered provided they did not include PNE. This may have included waitlist controls, physiotherapy, other educational methods or no treatment. ***D) Study Type:*** Randomized controlled trials.
- Exclusion criteria: None.

PRISMA flow diagram:

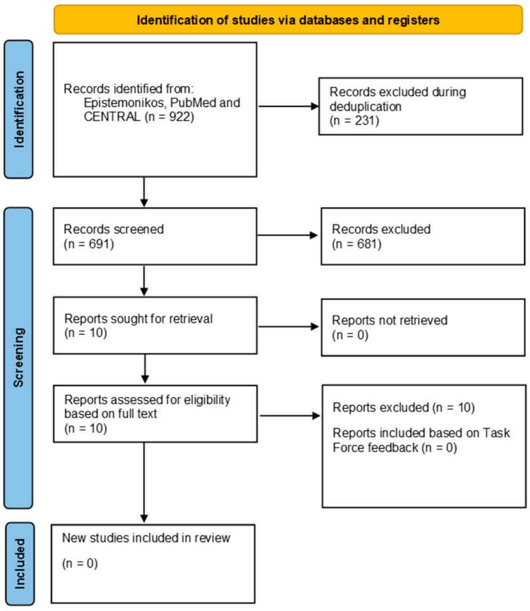

1.2. Epidemiological data

1a) Epidemiology

Database searched: PubMed and Embase

Search period: 01 April 2017 to 28 April 2022

Search strategy used:

Pubmed: (((("Low Back Pain"[Mesh] OR "Sciatica"[Mesh] OR "Low Backache*"[tiab] OR "Lower Backache*"[tiab] OR Lumbago[tiab] OR Sciatic* [tiab] OR (("Lumbar Vertebrae"[Mesh] OR "Lumbosacral Region"[Mesh] OR "Low Back"[tiab] OR "Lower Back"[tiab] OR Lumbar[tiab] OR Lumbosacral[tiab] OR "Lumbo-sacral"[tiab]) AND ("Pain"[Mesh] OR Ache*[tiab] OR Aching[tiab] OR Pain*[tiab])) OR (("Radiculopathy"[Mesh] OR "Radicular Pain"[tiab] OR Radiculitides[tiab] OR Radiculitis[tiab] OR Radiculopath*[tiab] OR (("Spinal Nerve Roots"[Mesh] OR "Nerve Root"[tiab]) AND ("Inflammation"[Mesh] OR "Pain"[Mesh] OR Ache*[tiab] OR Aching[tiab] OR Avulsion*[tiab] OR Compress*[tiab] OR Disorder*[tiab] OR Entrap*[tiab] OR Imping*[tiab] OR Inflam*[tiab] OR Irritat*[tiab] OR Pinch*[tiab] OR Trap*[tiab]))) AND ("Low Back Pain"[Mesh] OR "Lumbar Vertebrae"[Mesh] OR "Lumbosacral Region"[Mesh] OR Low[tiab] OR Lower[tiab] OR Lumbar[tiab] OR Lumbosacral[tiab] OR "Lumbo-sacral"[tiab])))) AND (("Epidemiological Monitoring"[Majr] OR "Incidence"[Majr] OR "epidemiology"[Subheading] OR "Population Surveillance"[Majr] OR

"Prevalence"[Majr] OR denominator[tiab] OR epidemiolog*[ti] OR frequency[ti] OR incident[ti] OR incidence[tiab] OR population-based[tiab] OR prevalent[ti] OR prevalence[tiab] OR proportion[ti] OR rate[ti] OR surveillance[tiab]))) AND ((("Meta-Analysis"[Publication Type] OR "Systematic Review"[Publication Type] OR "Systematic Reviews as Topic"[Mesh] OR "Systematic review*"[ti]) AND (2017/4/1:3000/12/31[pdat]) AND english[Filter]))) NOT (("Comment"[Publication Type] OR "Editorial"[Publication Type] OR "Ephemera"[Publication Type] OR "Letter"[Publication Type] OR "Newspaper Article"[Publication Type] OR "News"[Publication Type] OR comment*[ti] OR protocol*[ti]))

Embase: ('low back pain'/exp OR 'sciatica'/exp OR (("Low Backache*" OR "Lower Backache*" OR Lumbago OR Sciatic*):ti,ab) OR (('lumbar vertebra'/exp OR 'lumbosacral region'/exp OR (("Low Back" OR "Lower Back" OR Lumbar OR Lumbosacral OR "Lumbo-sacral"):ti,ab)) AND ('pain'/exp OR ((Ache* OR Aching OR Pain*):ti,ab))) OR (('radicular pain'/exp OR 'radiculopathy'/exp OR (("Radicular Pain" OR Radiculitides OR Radiculitis OR Radiculopath*):ti,ab) OR (('nerve root'/exp OR "Nerve Root":ti,ab) AND ('inflammation'/exp OR 'pain'/exp OR ((Ache* OR Aching OR Avulsion* OR Compress* OR Disorder* OR Entrap* OR Imping* OR Inflam* OR Irritat* OR Pinch* OR Trap*):ti,ab)))) AND ('low back pain'/exp OR 'lumbar vertebra'/exp OR 'lumbosacral region'/exp OR ((Low OR Lower OR Lumbar OR Lumbosacral OR "Lumbo-sacral"):ti,ab)))) AND (('epidemiological monitoring'/exp/mj OR 'incidence'/exp/mj OR 'population surveillance'/exp/mj OR 'prevalence'/exp OR denominator:ti,ab OR epidemiolog*:ti OR frequency:ti OR incident:ti OR incidence:ti,ab OR 'population-based':ti,ab OR prevalent:ti OR prevalence:ti,ab OR proportion:ti OR rate:ti OR surveillance:ti,ab)) AND ((('meta analysis'/exp OR 'meta analysis topic'/exp OR 'meta analysis (topic)'/exp OR 'systematic review'/exp OR 'systematic review topic'/exp OR 'systematic review (topic)'/exp OR 'systematic review$':ti) AND ([1-4-2017]/sd NOT [01-01-3000]/sd) AND [english]/lim)) NOT (('abstract report'/exp OR 'conference paper'/exp OR 'editorial'/exp OR 'letter'/exp OR 'note'/exp OR [conference abstract]/lim OR [conference paper]/lim OR [conference review]/lim OR [editorial]/lim OR [letter]/lim OR [note]/lim OR [short survey]/lim))

Eligibility criteria:

- Inclusion criteria: ***A) Epidemiology***: Incidence and prevalence of interventions and outcomes of relevant questions. ***B) Population:*** Participants of all age groups. ***C) Study Type:*** Systematic reviews.
- Exclusion criteria: None.

1b) Epidemiology with intervention

Database searched: PubMed and Embase

Search period: Not applied

Search strategy used:

Pubmed: (((("Low Back Pain"[Mesh] OR "Sciatica"[Mesh] OR "Low Backache*"[tiab] OR "Lower Backache*"[tiab] OR Lumbago[tiab] OR Sciatic* [tiab] OR "Low Back pain"[tiab]) AND ("Incidence"[Mesh] OR "epidemiology"[Subheading] OR "Prevalence"[Mesh] OR epidemiolog*[tiab] incidence[tiab] OR population-based[tiab] OR prevalence[tiab] OR proportion[tiab] OR surveillance[tiab])) AND ("Pain Management"[Majr] OR "Diagnostic Imaging"[Majr] OR "Risk Assessment"[Majr] OR "Drug Therapy"[Majr] OR "drug therapy" [Subheading] OR "Return to Work"[Majr] OR "Psychotherapy"[Majr] OR "Pathology"[Majr] OR "Radiculopathy"[Majr] OR "Neurosciences/education"[Majr] OR "Denervation"[Majr] OR "Decompression"[Majr] OR Imaging[tiab] OR "stratified management"[tiab] OR X-ray[tiab] OR MRI[tiab] OR Radiography[tiab] OR Magnetic resonance imaging[tiab] OR "risk assessment tool*"[tiab] OR "pharmacological treatment"[tiab] OR return-to-work[tiab] OR Psychological therap*[tiab] OR epidural injection*[tiab] OR concordant pathology[tiab] OR radicular symptom*[tiab] OR "pain neuroscience education"[tiab] OR "radiofrequency denervation"[tiab] OR "spinal decompression"[tiab])) AND (English[Filter])) NOT (("address"[Publication Type] OR "autobiography"[Publication Type] OR "bibliography"[Publication Type] OR "biography"[Publication Type] OR "Book

Illustrations"[Publication Type] OR "Case Reports"[Publication Type] OR "Comment"[Publication Type] OR "congress"[Publication Type] OR "consensus development conference"[Publication Type] OR "consensus development conference, nih"[Publication Type] OR "dictionary"[Publication Type] OR "directory"[Publication Type] OR "editorial"[Publication Type] OR "Expression of Concern"[Publication Type] OR "Guideline"[Publication Type] OR "interactive tutorial"[Publication Type] OR "interview"[Publication Type] OR "lecture"[Publication Type] OR "legal case"[Publication Type] OR "legislation"[Publication Type] OR "letter"[Publication Type] OR "Meta-Analysis"[Publication Type] OR "news"[Publication Type] OR "newspaper article"[Publication Type] OR "overall"[Publication Type] OR "patient education handout"[Publication Type] OR "periodical index"[Publication Type] OR "personal narrative"[Publication Type] OR "portrait"[Publication Type] OR "Review"[Publication Type] OR "Scientific Integrity Review"[Publication Type] OR "Systematic Review"[Publication Type] OR "hascommenton"[All Fields] OR "Cartoons as Topic"[Mesh] OR "Meta-Analysis as Topic"[Mesh] OR "Review Literature as Topic"[Mesh] OR "Systematic Reviews as Topic"[Mesh] OR "case report*"[tiab] OR "case series"[tiab] OR "integrative research review*"[tiab] OR "integrative review*"[tiab] OR "literature review"[tiab] OR meta-analys*[tiab] OR "meta analys*"[tiab] OR metaanalys*[tiab] OR "narrative review"[tiab] OR "research integration"[tiab] OR "scoping review"[tiab] OR ((methodologic*[tiab] OR quantitative*[tiab] OR systematic*[tiab]) AND (overview*[tiab] OR review*[tiab] OR synthesis*[tiab]))))

Embase: 'low back pain'/exp OR 'sciatica'/exp OR 'low backache*':ti,ab OR 'lower backache*':ti,ab OR lumbago:ti,ab OR sciatic*:ti,ab OR 'low back pain':ti,ab AND ('incidence'/exp OR 'epidemiology'/mj OR 'prevalence'/mj OR epidemiolog*:ti,ab OR incidence:ti,ab OR 'population-based':ti,ab OR prevalence:ti,ab OR proportion:ti,ab OR surveillance:ti,ab) AND ('disease management'/mj OR 'radiodiagnosis'/mj OR 'risk assessment'/mj OR 'drug therapy'/mj OR 'return to work'/mj OR 'psychotherapy'/mj OR 'epidural drug administration'/mj OR 'pathology'/mj OR 'radiculopathy'/mj OR 'neuroscience'/mj OR 'denervation'/mj OR 'decompression'/mj OR imaging:ti,ab OR 'stratified management':ti,ab OR 'x ray':ti,ab OR mri:ti,ab OR radiography:ti,ab OR 'magnetic resonance imaging':ti,ab OR 'risk assessment tool*':ti,ab OR 'pharmacological treatment':ti,ab OR 'return to work' OR 'psychological therap':ti,ab OR 'epidural injection*':ti,ab OR 'concordant pathology':ti,ab OR 'radicular symptom*':ti,ab OR 'pain neuroscience education':ti,ab OR 'radiofrequency denervation':ti,ab OR 'spinal decompression':ti,ab) AND ([english]/lim) NOT (('abstract report'/exp OR 'animal experiment'/exp OR 'book'/exp OR 'case finding'/exp OR 'case report'/exp OR 'case study'/exp OR 'conference paper'/exp OR 'editorial'/exp OR 'feasibility study'/exp OR 'in vitro study'/exp OR 'letter'/exp OR 'meta analysis'/exp OR 'meta analysis topic'/exp OR 'meta analysis (topic)'/exp OR 'note'/exp OR 'practice guideline'/exp OR 'review'/exp OR 'systematic review'/exp OR 'systematic review topic'/exp OR 'systematic review (topic)'/exp OR 'veterinary clinical trial'/exp OR 'veterinary study'/exp OR [conference abstract]/lim OR [conference paper]/lim OR [conference review]/lim OR [editorial]/lim OR [letter]/lim OR [note]/lim OR [short survey]/lim OR 'case report*':ti,ab OR 'case series':ti,ab OR 'integrative research review*':ti,ab OR 'integrative review*':ti,ab OR 'literature review':ti,ab OR 'meta analys*':ti,ab OR metaanalys*:ti,ab OR meta*analys*:ti,ab OR 'narrative review':ti,ab OR 'research integration':ti,ab OR 'scoping review':ti,ab OR ((integrative NEAR/5 research NEAR/5 review*):ti,ab) OR ((methodologic* NEAR/5 overview*):ti,ab) OR ((methodologic* NEAR/5 review*):ti,ab) OR ((quantitativ* NEAR/5 overview*):ti,ab) OR ((quantitativ* NEAR/5 review*):ti,ab) OR ((quantitativ* NEAR/5 synthesi*):ti,ab) OR ((research NEAR/5 integration):ti,ab) OR ((systematic* NEAR/5 overview*):ti,ab) OR ((systematic* NEAR/5 review*):ti,ab)))

Eligibility criteria:

- Inclusion criteria: ***A) Epidemiology***: Incidence and prevalence of interventions and outcomes of relevant questions. ***B) Population:*** Participants of all age groups. ***C) Study Type:*** Primary studies.
- Exclusion criteria: None.

1.3. Contextual factors

1a) Patient values and preferences

Database searched: PubMed and Embase

Search period: 01 April 2017 to 28 April 2022

Search strategy used:

PubMed: ((("Attitude to Health"[Mesh] OR "Patient Participation"[Mesh] OR "Patient Satisfaction"[Mesh] OR preference*[tiab] OR values[tiab] OR utility[tiab] OR "trade-off"[tiab] OR "trade off"[tiab] OR perspective*[tiab]) AND (("Low Back Pain"[Mesh] OR "Sciatica"[Mesh] OR "Low Backache*"[tiab] OR "Lower Backache*"[tiab] OR Lumbago[tiab] OR Sciatic* [tiab] OR (("Lumbar Vertebrae"[Mesh] OR "Lumbosacral Region"[Mesh] OR "Low Back"[tiab] OR "Lower Back"[tiab] OR Lumbar[tiab] OR Lumbosacral[tiab] OR "Lumbo-sacral"[tiab]) AND ("Pain"[Mesh] OR Ache*[tiab] OR Aching[tiab] OR Pain*[tiab])) OR (("Radiculopathy"[Mesh] OR "Radicular Pain"[tiab] OR Radiculitides[tiab] OR Radiculitis[tiab] OR Radiculopath*[tiab] OR (("Spinal Nerve Roots"[Mesh] OR "Nerve Root"[tiab]) AND ("Inflammation"[Mesh] OR "Pain"[Mesh] OR Ache*[tiab] OR Aching[tiab] OR Avulsion*[tiab] OR Compress*[tiab] OR Disorder*[tiab] OR Entrap*[tiab] OR Imping*[tiab] OR Inflam*[tiab] OR Irritat*[tiab] OR Pinch*[tiab] OR Trap*[tiab]))) AND ("Low Back Pain"[Mesh] OR "Lumbar Vertebrae"[Mesh] OR "Lumbosacral Region"[Mesh] OR Low[tiab] OR Lower[tiab] OR Lumbar[tiab] OR Lumbosacral[tiab] OR "Lumbo-sacral"[tiab]))))) AND ((("Meta-Analysis"[Publication Type] OR "Systematic Review"[Publication Type] OR "Systematic Reviews as Topic"[Mesh] OR "Systematic review*"[ti]) AND (2017/4/1:3000/12/31[pdat]) AND english[Filter]))) NOT (("Comment"[Publication Type] OR "Editorial"[Publication Type] OR "Ephemera"[Publication Type] OR "Letter"[Publication Type] OR "Newspaper Article"[Publication Type] OR "News"[Publication Type] OR comment*[ti] OR protocol*[ti]))

Embase: ('attitude to health'/exp OR 'patient participation'/exp OR 'patient satisfaction'/exp OR preference$:ti,ab OR values:ti,ab OR utility:ti,ab OR 'trade-off':ti,ab OR 'trade off':ti,ab OR perspective$:ti,ab) AND (('low back pain'/exp OR 'sciatica'/exp OR (("Low Backache*" OR "Lower Backache*" OR Lumbago OR Sciatic*):ti,ab) OR (('lumbar vertebra'/exp OR 'lumbosacral region'/exp OR (("Low Back" OR "Lower Back" OR Lumbar OR Lumbosacral OR "Lumbo-sacral"):ti,ab)) AND ('pain'/exp OR ((Ache* OR Aching OR Pain*):ti,ab))) OR (('radicular pain'/exp OR 'radiculopathy'/exp OR (("Radicular Pain" OR Radiculitides OR Radiculitis OR Radiculopath*):ti,ab) OR (('nerve root'/exp OR "Nerve Root":ti,ab) AND ('inflammation'/exp OR 'pain'/exp OR ((Ache* OR Aching OR Avulsion* OR Compress* OR Disorder* OR Entrap* OR Imping* OR Inflam* OR Irritat* OR Pinch* OR Trap*):ti,ab)))) AND ('low back pain'/exp OR 'lumbar vertebra'/exp OR 'lumbosacral region'/exp OR ((Low OR Lower OR Lumbar OR Lumbosacral OR "Lumbo-sacral"):ti,ab))))) AND ((('meta analysis'/exp OR 'meta analysis topic'/exp OR 'meta analysis (topic)'/exp OR 'systematic review'/exp OR 'systematic review topic'/exp OR 'systematic review (topic)'/exp OR 'systematic review$':ti) AND ([1-4-2017]/sd NOT [01-01-3000]/sd) AND [english]/lim)) NOT (('abstract report'/exp OR 'conference paper'/exp OR 'editorial'/exp OR 'letter'/exp OR 'note'/exp OR [conference abstract]/lim OR [conference paper]/lim OR [conference review]/lim OR [editorial]/lim OR [letter]/lim OR [note]/lim OR [short survey]/lim))

Eligibility criteria:

- Inclusion criteria: ***A) Patients' values and preferences*** – defined as the relative importance that people place on health outcomes. Keywords included Discrete choice; Decision making; Decision Support Systems; Patient participation; Patient satisfaction; Patient perception; choice; value; attitude; expectation; User participation; choice; valuation; perspective; Preference score; probability trade-off; best-worst scaling; Quality of life; EQ5D; EuroQoL 5D; SF 12; SF 36; Health related Quality of Life; HRQoL. ***B) Population:*** Patients/ Caregivers/ Physicians/ stakeholders related to low back pain. ***D) Study Type:*** Systematic reviews.
- Exclusion criteria: None.

1b) Patient values and preferences with intervention

Database searched: PubMed and Embase

Search period: Not applied.

Search strategy used:

PubMed: (((("Attitude to Health"[Mesh] OR preference*[ti] OR values[ti] OR utility[ti] OR "trade-off"[ti] OR "trade off"[ti] OR perspective*[ti]) AND ("Low Back Pain"[Mesh] OR "Sciatica"[Mesh] OR "Low Backache*"[tiab] OR "Lower Backache*"[tiab] OR Lumbago[tiab] OR Sciatic* [tiab] OR "Low Back pain"[tiab])) AND ("Pain Management"[Majr] OR "Diagnostic Imaging"[Majr] OR "Risk Assessment"[Majr] OR "Drug Therapy"[Majr] OR "drug therapy" [Subheading] OR "Return to Work"[Majr] OR "Psychotherapy"[Majr] OR "Pathology"[Majr] OR "Radiculopathy"[Majr] OR "Neurosciences/education"[Majr] OR "Denervation"[Majr] OR "Decompression"[Majr] OR Imaging[tiab] OR "stratified management"[tiab] OR X-ray[tiab] OR MRI[tiab] OR Radiography[tiab] OR Magnetic resonance imaging[tiab] OR "risk assessment tool*"[tiab] OR "pharmacological treatment"[tiab] OR return-to-work[tiab] OR Psychological therap*[tiab] OR epidural injection*[tiab] OR concordant pathology[tiab] OR radicular symptom*[tiab] OR "pain neuroscience education"[tiab] OR "radiofrequency denervation"[tiab] OR "spinal decompression"[tiab])) AND (English[Filter])) NOT (("address"[Publication Type] OR "autobiography"[Publication Type] OR "bibliography"[Publication Type] OR "biography"[Publication Type] OR "Book Illustrations"[Publication Type] OR "Case Reports"[Publication Type] OR "Comment"[Publication Type] OR "congress"[Publication Type] OR "consensus development conference"[Publication Type] OR "consensus development conference, nih"[Publication Type] OR "dictionary"[Publication Type] OR "directory"[Publication Type] OR "editorial"[Publication Type] OR "Expression of Concern"[Publication Type] OR "Guideline"[Publication Type] OR "interactive tutorial"[Publication Type] OR "interview"[Publication Type] OR "lecture"[Publication Type] OR "legal case"[Publication Type] OR "legislation"[Publication Type] OR "letter"[Publication Type] OR "Meta-Analysis"[Publication Type] OR "news"[Publication Type] OR "newspaper article"[Publication Type] OR "overall"[Publication Type] OR "patient education handout"[Publication Type] OR "periodical index"[Publication Type] OR "personal narrative"[Publication Type] OR "portrait"[Publication Type] OR "Review"[Publication Type] OR "Scientific Integrity Review"[Publication Type] OR "Systematic Review"[Publication Type] OR "hascommenton"[All Fields] OR "Cartoons as Topic"[Mesh] OR "Meta-Analysis as Topic"[Mesh] OR "Review Literature as Topic"[Mesh] OR "Systematic Reviews as Topic"[Mesh] OR "case report*"[tiab] OR "case series"[tiab] OR "integrative research review*"[tiab] OR "integrative review*"[tiab] OR "literature review"[tiab] OR meta-analys*[tiab] OR "meta analys*"[tiab] OR metaanalys*[tiab] OR "narrative review"[tiab] OR "research integration"[tiab] OR "scoping review"[tiab] OR ((methodologic*[tiab] OR quantitative*[tiab] OR systematic*[tiab]) AND (overview*[tiab] OR review*[tiab] OR synthesis*[tiab]))))

Embase: ('attitude to health'/exp OR preference$:ti OR values:ti OR utility:ti OR 'trade-off':ti OR 'trade off':ti OR perspective$:ti) AND ('low back pain'/exp OR 'sciatica'/exp OR 'low backache*':ti,ab OR 'lower backache*':ti,ab OR lumbago:ti,ab OR sciatic*:ti,ab OR 'low back pain':ti,ab) AND ('disease management'/mj OR 'radiodiagnosis'/mj OR 'risk assessment'/mj OR 'drug therapy'/mj OR 'return to work'/mj OR 'psychotherapy'/mj OR 'epidural drug administration'/mj OR 'pathology'/mj OR 'radiculopathy'/mj OR 'neuroscience'/mj OR 'denervation'/mj OR 'decompression'/mj OR imaging:ti,ab OR 'stratified management':ti,ab OR 'x ray':ti,ab OR mri:ti,ab OR radiography:ti,ab OR 'magnetic resonance imaging':ti,ab OR 'risk assessment tool*':ti,ab OR 'pharmacological treatment':ti,ab OR 'return to work' OR 'psychological therap':ti,ab OR 'epidural injection*':ti,ab OR 'concordant pathology':ti,ab OR 'radicular symptom*':ti,ab OR 'pain neuroscience education':ti,ab OR 'radiofrequency denervation':ti,ab OR 'spinal decompression':ti,ab) AND ([english]/lim) NOT (('abstract report'/exp OR 'animal experiment'/exp OR 'book'/exp OR 'case finding'/exp OR 'case report'/exp OR 'case study'/exp OR 'conference paper'/exp OR 'editorial'/exp OR 'feasibility study'/exp OR 'in vitro study'/exp) AND 'letter'/exp OR 'meta analysis'/exp OR 'meta analysis topic'/exp OR 'meta analysis (topic)'/exp OR 'note'/exp OR 'practice guideline'/exp OR 'review'/exp OR 'systematic review'/exp OR 'systematic review topic'/exp OR 'systematic review (topic)'/exp OR 'veterinary clinical trial'/exp OR 'veterinary study'/exp OR [conference abstract]/lim OR [conference paper]/lim OR [conference review]/lim OR [editorial]/lim OR [letter]/lim OR [note]/lim OR [short survey]/lim OR

'case report*':ti,ab OR 'case series':ti,ab OR 'integrative research review*':ti,ab OR 'integrative review*':ti,ab OR 'literature review':ti,ab OR 'meta analys*':ti,ab OR metaanalys*:ti,ab OR meta*analys*:ti,ab OR 'narrative review':ti,ab OR 'research integration':ti,ab OR 'scoping review':ti,ab OR ((integrative NEAR/5 research NEAR/5 review*):ti,ab) OR ((methodologic* NEAR/5 overview*):ti,ab) OR ((methodologic* NEAR/5 review*):ti,ab) OR ((quantitativ* NEAR/5 overview*):ti,ab) OR ((quantitativ* NEAR/5 review*):ti,ab) OR ((quantitativ* NEAR/5 synthesi*):ti,ab) OR ((research NEAR/5 integration):ti,ab) OR ((systematic* NEAR/5 overview*):ti,ab) OR ((systematic* NEAR/5 review*):ti,ab))

Eligibility criteria:

- Inclusion criteria: ***A) Patients' values and preferences*** – defined as the relative importance that people place on health outcomes. Keywords included Discrete choice; Decision making; Decision Support Systems; Patient participation; Patient satisfaction; Patient perception; choice; value; attitude; expectation; User participation; choice; valuation; perspective; Preference score; probability trade-off; best-worst scaling; Quality of life; EQ5D; EuroQoL 5D; SF 12; SF 36; Health related Quality of Life; HRQoL. ***B) Population:*** Patients/ Caregivers/ Physicians/ stakeholders related to low back pain. ***D) Study Type:*** Primary studies.
- Exclusion criteria: None.

2a) Equity

Database searched: PubMed and Embase

Search period: 01 April 2017 to 28 April 2022

Search strategy used:

PubMed: ((((((Health Services Accessibility[Majr] OR "Access to Health Care"[tiab] OR "Access to Health Service*"[tiab] OR "Access to HealthCare"[tiab] OR "Access To Medic*"[tiab] OR "Access to Medication*"[tiab] OR "Access to Therap*"[tiab] OR "Access to Treat*"[tiab] OR "Accessibility of Health Service*"[tiab] OR "Availability of Health Service*"[tiab] OR coercion[tiab] OR coercive*[tiab] OR controvers*[tiab] OR "Health Services Accessibilit*"[tiab] OR "Health Services Availabilit*"[tiab] OR "Medication Access*"[tiab] OR "Program Accessibilit*"[tiab])) OR ((Healthcare Disparities[Majr] OR equit*[tiab] OR "Health Care Disparit*"[tiab] OR "Health Care Inequalit*"[tiab] OR "Healthcare Disparit*"[tiab] OR "Healthcare Inequalit*"[tiab] OR inequit*[tiab]))) OR (("Morals"[Majr] OR "ethics"[Subheading] OR ethic*[tiab] OR fairness[tiab] OR moral*[tiab] OR unethical[tiab]))) AND (("Low Back Pain"[Mesh] OR "Sciatica"[Mesh] OR "Low Backache*"[tiab] OR "Lower Backache*"[tiab] OR Lumbago[tiab] OR Sciatic* [tiab] OR (("Lumbar Vertebrae"[Mesh] OR "Lumbosacral Region"[Mesh] OR "Low Back"[tiab] OR "Lower Back"[tiab] OR Lumbar[tiab] OR Lumbosacral[tiab] OR "Lumbo-sacral"[tiab]) AND ("Pain"[Mesh] OR Ache*[tiab] OR Aching[tiab] OR Pain*[tiab])) OR (("Radiculopathy"[Mesh] OR "Radicular Pain"[tiab] OR Radiculitides[tiab] OR Radiculitis[tiab] OR Radiculopath*[tiab] OR (("Spinal Nerve Roots"[Mesh] OR "Nerve Root"[tiab]) AND ("Inflammation"[Mesh] OR "Pain"[Mesh] OR Ache*[tiab] OR Aching[tiab] OR Avulsion*[tiab] OR Compress*[tiab] OR Disorder*[tiab] OR Entrap*[tiab] OR Imping*[tiab] OR Inflam*[tiab] OR Irritat*[tiab] OR Pinch*[tiab] OR Trap*[tiab]))) AND ("Low Back Pain"[Mesh] OR "Lumbar Vertebrae"[Mesh] OR "Lumbosacral Region"[Mesh] OR Low[tiab] OR Lower[tiab] OR Lumbar[tiab] OR Lumbosacral[tiab] OR "Lumbo-sacral"[tiab]))))) AND ((("Meta-Analysis"[Publication Type] OR "Systematic Review"[Publication Type] OR "Systematic Reviews as Topic"[Mesh] OR "Systematic review*"[ti]) AND (2017/4/1:3000/12/31[pdat]) AND english[Filter]))) NOT (("Comment"[Publication Type] OR "Editorial"[Publication Type] OR "Ephemera"[Publication Type] OR "Letter"[Publication Type] OR "Newspaper Article"[Publication Type] OR "News"[Publication Type] OR comment*[ti] OR protocol*[ti]))

Embase: 'health care access'/mj OR 'access to health care':ti,ab OR 'access to health service*':ti,ab OR 'access to healthcare':ti,ab OR 'access to medic*':ti,ab OR 'access to medication*':ti,ab OR 'access to therap*':ti,ab OR 'access to treat*':ti,ab OR 'accessibility of health service*':ti,ab OR 'availability of

health service*':ti,ab OR coercion:ti,ab OR coercive*:ti,ab OR controvers*:ti,ab OR 'health services accessibilit*':ti,ab OR 'health services availabilit*':ti,ab OR 'medication access*':ti,ab OR 'program accessibilit*':ti,ab OR ('health care disparity'/mj OR equit*:ti,ab OR 'health care disparit*':ti,ab OR 'health care inequalit*':ti,ab OR 'healthcare disparit*':ti,ab OR 'healthcare inequalit*':ti,ab OR inequit*:ti,ab) OR ('morality'/mj OR 'ethics'/mj OR ethic*:ti,ab OR fairness:ti,ab OR moral*:ti,ab OR unethical:ti,ab) AND (('low back pain'/exp OR 'sciatica'/exp OR (("Low Backache*" OR "Lower Backache*" OR Lumbago OR Sciatic*):ti,ab) OR (('lumbar vertebra'/exp OR 'lumbosacral region'/exp OR (("Low Back" OR "Lower Back" OR Lumbar OR Lumbosacral OR "Lumbo-sacral"):ti,ab)) AND ('pain'/exp OR ((Ache* OR Aching OR Pain*):ti,ab))) OR (('radicular pain'/exp OR 'radiculopathy'/exp OR (("Radicular Pain" OR Radiculitides OR Radiculitis OR Radiculopath*):ti,ab) OR (('nerve root'/exp OR "Nerve Root":ti,ab) AND ('inflammation'/exp OR 'pain'/exp OR ((Ache* OR Aching OR Avulsion* OR Compress* OR Disorder* OR Entrap* OR Imping* OR Inflam* OR Irritat* OR Pinch* OR Trap*):ti,ab)))) AND ('low back pain'/exp OR 'lumbar vertebra'/exp OR 'lumbosacral region'/exp OR ((Low OR Lower OR Lumbar OR Lumbosacral OR "Lumbo-sacral"):ti,ab))))) AND ((('meta analysis'/exp OR 'meta analysis topic'/exp OR 'meta analysis (topic)'/exp OR 'systematic review'/exp OR 'systematic review topic'/exp OR 'systematic review (topic)'/exp OR 'systematic review$':ti) AND ([1-4-2017]/sd NOT [01-01-3000]/sd) AND [english]/lim)) NOT (('abstract report'/exp OR 'conference paper'/exp OR 'editorial'/exp OR 'letter'/exp OR 'note'/exp OR [conference abstract]/lim OR [conference paper]/lim OR [conference review]/lim OR [editorial]/lim OR [letter]/lim OR [note]/lim OR [short survey]/lim))

Eligibility criteria:

- Inclusion criteria: ***A) Health inequity*** – was defined as systematic, socially produced (and therefore modifiable) and unfair differences in health. Populations may be considered at risk of disadvantage because of demographic and social characteristics such as a person's place of residence, race/ethnicity/culture/language, occupation, gender/sex, religion, education, socioeconomic status, or social capital (PROGRESS) and other characteristics such as age, disability or temporary conditions that put people at risk of health inequities, due across axes such as access, opportunity to benefit or capacity to implement changes. Keywords included: health service accessibility; access to health care; access to health service; access to medication; access to therapy; access to treat; coercion; coercive; health service availability; program accessibility; healthcare disparities; healthcare inequalities; inequities; morals; ethics; fairness; unethical. ***B) Population:*** Patients/ Caregivers/ Physicians/ stakeholders related to low back pain. ***C) Study Type:*** Systematic reviews.
- Exclusion criteria: None.

2b) Equity with intervention

Database searched: PubMed and Embase

Search period: Not applied

Search strategy used:

PubMed: (((((((Health Services Accessibility[Majr] OR "Access to Health Care"[tiab] OR "Access to Health Service*"[tiab] OR "Access to HealthCare"[tiab] OR "Access To Medic*"[tiab] OR "Access to Medication*"[tiab] OR "Access to Therap*"[tiab] OR "Access to Treat*"[tiab] OR "Accessibility of Health Service*"[tiab] OR "Availability of Health Service*"[tiab] OR coercion[tiab] OR coercive*[tiab] OR controvers*[tiab] OR "Health Services Accessibilit*"[tiab] OR "Health Services Availabilit*"[tiab] OR "Medication Access*"[tiab] OR "Program Accessibilit*"[tiab])) OR ((Healthcare Disparities[Majr] OR equit*[tiab] OR "Health Care Disparit*"[tiab] OR "Health Care Inequalit*"[tiab] OR "Healthcare Disparit*"[tiab] OR "Healthcare Inequalit*"[tiab] OR inequit*[tiab]))) OR (("Morals"[Majr] OR "ethics"[Subheading] OR ethic*[tiab] OR fairness[tiab] OR moral*[tiab] OR unethical[tiab]))) AND ("Low Back Pain"[Mesh] OR "Sciatica"[Mesh] OR "Low Backache*"[tiab] OR "Lower Backache*"[tiab] OR Lumbago[tiab] OR Sciatic* [tiab] OR "Low Back pain"[tiab])) AND ("Pain Management"[Majr] OR "Diagnostic Imaging"[Majr] OR "Risk

Assessment"[Majr] OR "Drug Therapy"[Majr] OR "drug therapy" [Subheading] OR "Return to Work"[Majr] OR "Psychotherapy"[Majr] OR "Pathology"[Majr] OR "Radiculopathy"[Majr] OR "Neurosciences/education"[Majr] OR "Denervation"[Majr] OR "Decompression"[Majr] OR Imaging[tiab] OR "stratified management"[tiab] OR X-ray[tiab] OR MRI[tiab] OR Radiography[tiab] OR Magnetic resonance imaging[tiab] OR "risk assessment tool*"[tiab] OR "pharmacological treatment"[tiab] OR return-to-work[tiab] OR Psychological therap*[tiab] OR epidural injection*[tiab] OR concordant pathology[tiab] OR radicular symptom*[tiab] OR "pain neuroscience education"[tiab] OR "radiofrequency denervation"[tiab] OR "spinal decompression"[tiab])) AND (English[Filter])) NOT (("address"[Publication Type] OR "autobiography"[Publication Type] OR "bibliography"[Publication Type] OR "biography"[Publication Type] OR "Book Illustrations"[Publication Type] OR "Case Reports"[Publication Type] OR "Comment"[Publication Type] OR "congress"[Publication Type] OR "consensus development conference"[Publication Type] OR "consensus development conference, nih"[Publication Type] OR "dictionary"[Publication Type] OR "directory"[Publication Type] OR "editorial"[Publication Type] OR "Expression of Concern"[Publication Type] OR "Guideline"[Publication Type] OR "interactive tutorial"[Publication Type] OR "interview"[Publication Type] OR "lecture"[Publication Type] OR "legal case"[Publication Type] OR "legislation"[Publication Type] OR "letter"[Publication Type] OR "Meta-Analysis"[Publication Type] OR "news"[Publication Type] OR "newspaper article"[Publication Type] OR "overall"[Publication Type] OR "patient education handout"[Publication Type] OR "periodical index"[Publication Type] OR "personal narrative"[Publication Type] OR "portrait"[Publication Type] OR "Review"[Publication Type] OR "Scientific Integrity Review"[Publication Type] OR "Systematic Review"[Publication Type] OR "hascommenton"[All Fields] OR "Cartoons as Topic"[Mesh] OR "Meta-Analysis as Topic"[Mesh] OR "Review Literature as Topic"[Mesh] OR "Systematic Reviews as Topic"[Mesh] OR "case report*"[tiab] OR "case series"[tiab] OR "integrative research review*"[tiab] OR "integrative review*"[tiab] OR "literature review"[tiab] OR meta-analys*[tiab] OR "meta analys*"[tiab] OR metaanalys*[tiab] OR "narrative review"[tiab] OR "research integration"[tiab] OR "scoping review"[tiab] OR ((methodologic*[tiab] OR quantitative*[tiab] OR systematic*[tiab]) AND (overview*[tiab] OR review*[tiab] OR synthesis*[tiab]))))

Embase: 'health care access'/mj OR 'access to health care':ti,ab OR 'access to health service*':ti,ab OR 'access to healthcare':ti,ab OR 'access to medic*':ti,ab OR 'access to medication*':ti,ab OR 'access to therap*':ti,ab OR 'access to treat*':ti,ab OR 'accessibility of health service*':ti,ab OR 'availability of health service*':ti,ab OR coercion:ti,ab OR coercive*:ti,ab OR controvers*:ti,ab OR 'health services accessibilit*':ti,ab OR 'health services availabilit*':ti,ab OR 'medication access*':ti,ab OR 'program accessibilit*':ti,ab OR ('health care disparity'/mj OR equit*:ti,ab OR 'health care disparit*':ti,ab OR 'health care inequalit*':ti,ab OR 'healthcare disparit*':ti,ab OR 'healthcare inequalit*':ti,ab OR inequit*:ti,ab) OR ('morality'/mj OR 'ethics'/mj OR ethic*:ti,ab OR fairness:ti,ab OR moral*:ti,ab OR unethical:ti,ab) AND ('low back pain'/exp OR 'sciatica'/exp OR 'low backache*':ti,ab OR 'lower backache*':ti,ab OR lumbago:ti,ab OR sciatic*:ti,ab OR 'low back pain':ti,ab) AND ('disease management'/mj OR 'radiodiagnosis'/mj OR 'risk assessment'/mj OR 'drug therapy'/mj OR 'return to work'/mj OR 'psychotherapy'/mj OR 'epidural drug administration'/mj OR 'pathology'/mj OR 'radiculopathy'/mj OR 'neuroscience'/mj OR 'denervation'/mj OR 'decompression'/mj OR imaging:ti,ab OR 'stratified management':ti,ab OR 'x ray':ti,ab OR mri:ti,ab OR radiography:ti,ab OR 'magnetic resonance imaging':ti,ab OR 'risk assessment tool*':ti,ab OR 'pharmacological treatment':ti,ab OR 'return to work' OR 'psychological therap':ti,ab OR 'epidural injection*':ti,ab OR 'concordant pathology':ti,ab OR 'radicular symptom*':ti,ab OR 'pain neuroscience education':ti,ab OR 'radiofrequency denervation':ti,ab OR 'spinal decompression':ti,ab) AND ([english]/lim) NOT (('abstract report'/exp OR 'animal experiment'/exp OR 'book'/exp OR 'case finding'/exp OR 'case report'/exp OR 'case study'/exp OR 'conference paper'/exp OR 'editorial'/exp OR 'feasibility study'/exp OR 'in vitro study'/exp) AND 'letter'/exp OR 'meta analysis'/exp OR 'meta analysis topic'/exp OR 'meta analysis (topic)'/exp OR 'note'/exp OR 'practice guideline'/exp OR 'review'/exp OR 'systematic review'/exp OR 'systematic review topic'/exp OR 'systematic review (topic)'/exp OR 'veterinary clinical trial'/exp OR 'veterinary study'/exp OR [conference abstract]/lim OR [conference paper]/lim OR [conference review]/lim OR [editorial]/lim OR [letter]/lim OR [note]/lim OR [short survey]/lim OR 'case report*':ti,ab OR 'case series':ti,ab OR 'integrative research review*':ti,ab OR 'integrative

review*':ti,ab OR 'literature review':ti,ab OR 'meta analys*':ti,ab OR metaanalys*:ti,ab OR meta*analys*:ti,ab OR 'narrative review':ti,ab OR 'research integration':ti,ab OR 'scoping review':ti,ab OR ((integrative NEAR/5 research NEAR/5 review*):ti,ab) OR ((methodologic* NEAR/5 overview*):ti,ab) OR ((methodologic* NEAR/5 review*):ti,ab) OR ((quantitativ* NEAR/5 overview*):ti,ab) OR ((quantitativ* NEAR/5 review*):ti,ab) OR ((quantitativ* NEAR/5 synthesi*):ti,ab) OR ((research NEAR/5 integration):ti,ab) OR ((systematic* NEAR/5 overview*):ti,ab) OR ((systematic* NEAR/5 review*):ti,ab))

Eligibility criteria:

- Inclusion criteria: ***A) Health inequity*** – was defined as systematic, socially produced (and therefore modifiable) and unfair differences in health. Populations may be considered at risk of disadvantage because of demographic and social characteristics such as a person's place of residence, race/ethnicity/culture/language, occupation, gender/sex, religion, education, socioeconomic status, or social capital (PROGRESS) and other characteristics such as age, disability or temporary conditions that put people at risk of health inequities, due across axes such as access, opportunity to benefit or capacity to implement changes. Keywords included: health service accessibility; access to health care; access to health service; access to medication; access to therapy; access to treat; coercion; coercive; health service availability; program accessibility; healthcare disparities; healthcare inequalities; inequities; morals; ethics; fairness; unethical. ***B) Population:*** Patients/ Caregivers/ Physicians/ stakeholders related to low back pain. ***C) Study Type:*** Primary studies.
- Exclusion criteria: None.

3a) Feasibility

Database searched: PubMed and Embase

Search period: 01 April 2017 to 02 May 2022

Search strategy used:

PubMed: ((((("Feasibility Studies"[Majr] OR feasib*[tiab]) OR (facilita*[tiab] OR usabilit*[tiab])) OR (barrier*[tiab] OR difficult*[tiab] OR hurdle*[tiab])) AND (("Low Back Pain"[Mesh] OR "Sciatica"[Mesh] OR "Low Backache*"[tiab] OR "Lower Backache*"[tiab] OR Lumbago[tiab] OR Sciatic* [tiab] OR (("Lumbar Vertebrae"[Mesh] OR "Lumbosacral Region"[Mesh] OR "Low Back"[tiab] OR "Lower Back"[tiab] OR Lumbar[tiab] OR Lumbosacral[tiab] OR "Lumbo-sacral"[tiab]) AND ("Pain"[Mesh] OR Ache*[tiab] OR Aching[tiab] OR Pain*[tiab])) OR (("Radiculopathy"[Mesh] OR "Radicular Pain"[tiab] OR Radiculitides[tiab] OR Radiculitis[tiab] OR Radiculopath*[tiab] OR (("Spinal Nerve Roots"[Mesh] OR "Nerve Root"[tiab]) AND ("Inflammation"[Mesh] OR "Pain"[Mesh] OR Ache*[tiab] OR Aching[tiab] OR Avulsion*[tiab] OR Compress*[tiab] OR Disorder*[tiab] OR Entrap*[tiab] OR Imping*[tiab] OR Inflam*[tiab] OR Irritat*[tiab] OR Pinch*[tiab] OR Trap*[tiab]))) AND ("Low Back Pain"[Mesh] OR "Lumbar Vertebrae"[Mesh] OR "Lumbosacral Region"[Mesh] OR Low[tiab] OR Lower[tiab] OR Lumbar[tiab] OR Lumbosacral[tiab] OR "Lumbo-sacral"[tiab]))))) AND ((("Meta-Analysis"[Publication Type] OR "Systematic Review"[Publication Type] OR "Systematic Reviews as Topic"[Mesh] OR "Systematic review*"[ti]) AND (2017/4/1:3000/12/31[pdat]) AND english[Filter]))) NOT (("Comment"[Publication Type] OR "Editorial"[Publication Type] OR "Ephemera"[Publication Type] OR "Letter"[Publication Type] OR "Newspaper Article"[Publication Type] OR "News"[Publication Type] OR comment*[ti] OR protocol*[ti]))

Embase: ('feasibility study'/mj OR ((feasib*):ti,ab)) OR (((facilita* OR usabilit*):ti,ab)) OR (((barrier* OR difficult* OR hurdle*):ti,ab)) AND (('low back pain'/exp OR 'sciatica'/exp OR (("Low Backache*" OR "Lower Backache*" OR Lumbago OR Sciatic*):ti,ab) OR (('lumbar vertebra'/exp OR 'lumbosacral region'/exp OR (("Low Back" OR "Lower Back" OR Lumbar OR Lumbosacral OR "Lumbo-sacral"):ti,ab)) AND ('pain'/exp OR ((Ache* OR Aching OR Pain*):ti,ab))) OR (('radicular pain'/exp OR 'radiculopathy'/exp OR (("Radicular Pain" OR Radiculitides OR Radiculitis OR Radiculopath*):ti,ab) OR (('nerve root'/exp OR "Nerve Root":ti,ab) AND ('inflammation'/exp OR

'pain'/exp OR ((Ache* OR Aching OR Avulsion* OR Compress* OR Disorder* OR Entrap* OR Imping* OR Inflam* OR Irritat* OR Pinch* OR Trap*):ti,ab)))) AND ('low back pain'/exp OR 'lumbar vertebra'/exp OR 'lumbosacral region'/exp OR ((Low OR Lower OR Lumbar OR Lumbosacral OR "Lumbo-sacral"):ti,ab))))) AND ((('meta analysis'/exp OR 'meta analysis topic'/exp OR 'meta analysis (topic)'/exp OR 'systematic review'/exp OR 'systematic review topic'/exp OR 'systematic review (topic)'/exp OR 'systematic review$':ti) AND ([1-4-2017]/sd NOT [01-01-3000]/sd) AND [english]/lim)) NOT (('abstract report'/exp OR 'conference paper'/exp OR 'editorial'/exp OR 'letter'/exp OR 'note'/exp OR [conference abstract]/lim OR [conference paper]/lim OR [conference review]/lim OR [editorial]/lim OR [letter]/lim OR [note]/lim OR [short survey]/lim))

Eligibility criteria:

- Inclusion criteria: ***A) Feasibility*** – addressed whether the procedure could be implemented, or if there were substantial barriers to overcome. Keywords included effectiveness; efficacy; facilitative; usability; barriers; difficulty; hurdles; impedes; impediment; limiting; obstacle. ***B) Population:*** Patients/ Caregivers/ Physicians/ stakeholders related to low back pain. ***D) Study Type:*** Systematic reviews.
- Exclusion criteria: None.

3b) Feasibility with intervention

Database searched: PubMed and Embase

Search period: Not applied

PubMed: (((((("Feasibility Studies"[Majr] OR feasib*[ti]) OR (facilita*[ti] OR usabilit*[ti])) OR (barrier*[ti] OR difficult*[ti] OR hurdle*[ti])) AND ("Low Back Pain"[Mesh] OR "Sciatica"[Mesh] OR "Low Backache*"[tiab] OR "Lower Backache*"[tiab] OR Lumbago[tiab] OR Sciatic* [tiab] OR "Low Back pain"[tiab])) AND ("Pain Management"[Majr] OR "Diagnostic Imaging"[Majr] OR "Risk Assessment"[Majr] OR "Drug Therapy"[Majr] OR "drug therapy" [Subheading] OR "Return to Work"[Majr] OR "Psychotherapy"[Majr] OR "Pathology"[Majr] OR "Radiculopathy"[Majr] OR "Neurosciences/education"[Majr] OR "Denervation"[Majr] OR "Decompression"[Majr] OR Imaging[tiab] OR "stratified management"[tiab] OR X-ray[tiab] OR MRI[tiab] OR Radiography[tiab] OR Magnetic resonance imaging[tiab] OR "risk assessment tool*"[tiab] OR "pharmacological treatment"[tiab] OR return-to-work[tiab] OR Psychological therap*[tiab] OR epidural injection*[tiab] OR concordant pathology[tiab] OR radicular symptom*[tiab] OR "pain neuroscience education"[tiab] OR "radiofrequency denervation"[tiab] OR "spinal decompression"[tiab])) AND (English[Filter])) NOT (("address"[Publication Type] OR "autobiography"[Publication Type] OR "bibliography"[Publication Type] OR "biography"[Publication Type] OR "Book Illustrations"[Publication Type] OR "Case Reports"[Publication Type] OR "Comment"[Publication Type] OR "congress"[Publication Type] OR "consensus development conference"[Publication Type] OR "consensus development conference, nih"[Publication Type] OR "dictionary"[Publication Type] OR "directory"[Publication Type] OR "editorial"[Publication Type] OR "Expression of Concern"[Publication Type] OR "Guideline"[Publication Type] OR "interactive tutorial"[Publication Type] OR "interview"[Publication Type] OR "lecture"[Publication Type] OR "legal case"[Publication Type] OR "legislation"[Publication Type] OR "letter"[Publication Type] OR "Meta-Analysis"[Publication Type] OR "news"[Publication Type] OR "newspaper article"[Publication Type] OR "overall"[Publication Type] OR "patient education handout"[Publication Type] OR "periodical index"[Publication Type] OR "personal narrative"[Publication Type] OR "portrait"[Publication Type] OR "Review"[Publication Type] OR "Scientific Integrity Review"[Publication Type] OR "Systematic Review"[Publication Type] OR "hascommenton"[All Fields] OR "Cartoons as Topic"[Mesh] OR "Meta-Analysis as Topic"[Mesh] OR "Review Literature as Topic"[Mesh] OR "Systematic Reviews as Topic"[Mesh] OR "case report*"[tiab] OR "case series"[tiab] OR "integrative research review*"[tiab] OR "integrative review*"[tiab] OR "literature review"[tiab] OR meta-analys*[tiab] OR "meta analys*"[tiab] OR metaanalys*[tiab] OR "narrative review"[tiab] OR "research integration"[tiab] OR "scoping review"[tiab] OR ((methodologic*[tiab] OR quantitative*[tiab] OR systematic*[tiab]) AND (overview*[tiab] OR review*[tiab] OR synthesis*[tiab]))))

Embase: ('feasibility study'/mj OR ((feasib*):ti)) OR (((facilita* OR usabilit*):ti)) OR (((barrier* OR difficult* OR hurdle*):ti)) AND ('low back pain'/exp OR 'sciatica'/exp OR 'low backache*':ti,ab OR 'lower backache*':ti,ab OR lumbago:ti,ab OR sciatic*:ti,ab OR 'low back pain':ti,ab) AND ('disease management'/mj OR 'radiodiagnosis'/mj OR 'risk assessment'/mj OR 'drug therapy'/mj OR 'return to work'/mj OR 'psychotherapy'/mj OR 'epidural drug administration'/mj OR 'pathology'/mj OR 'radiculopathy'/mj OR 'neuroscience'/mj OR 'denervation'/mj OR 'decompression'/mj OR imaging:ti,ab OR 'stratified management':ti,ab OR 'x ray':ti,ab OR mri:ti,ab OR radiography:ti,ab OR 'magnetic resonance imaging':ti,ab OR 'risk assessment tool*':ti,ab OR 'pharmacological treatment':ti,ab OR 'return to work' OR 'psychological therap':ti,ab OR 'epidural injection*':ti,ab OR 'concordant pathology':ti,ab OR 'radicular symptom*':ti,ab OR 'pain neuroscience education':ti,ab OR 'radiofrequency denervation':ti,ab OR 'spinal decompression':ti,ab) AND ([english]/lim) NOT (('abstract report'/exp OR 'animal experiment'/exp OR 'book'/exp OR 'case finding'/exp OR 'case report'/exp OR 'case study'/exp OR 'conference paper'/exp OR 'editorial'/exp OR 'feasibility study'/exp OR 'in vitro study'/exp) AND 'letter'/exp OR 'meta analysis'/exp OR 'meta analysis topic'/exp OR 'meta analysis (topic)'/exp OR 'note'/exp OR 'practice guideline'/exp OR 'review'/exp OR 'systematic review'/exp OR 'systematic review topic'/exp OR 'systematic review (topic)'/exp OR 'veterinary clinical trial'/exp OR 'veterinary study'/exp OR [conference abstract]/lim OR [conference paper]/lim OR [conference review]/lim OR [editorial]/lim OR [letter]/lim OR [note]/lim OR [short survey]/lim OR 'case report*':ti,ab OR 'case series':ti,ab OR 'integrative research review*':ti,ab OR 'integrative review*':ti,ab OR 'literature review':ti,ab OR 'meta analys*':ti,ab OR metaanalys*:ti,ab OR meta*analys*:ti,ab OR 'narrative review':ti,ab OR 'research integration':ti,ab OR 'scoping review':ti,ab OR ((integrative NEAR/5 research NEAR/5 review*):ti,ab) OR ((methodologic* NEAR/5 overview*):ti,ab) OR ((methodologic* NEAR/5 review*):ti,ab) OR ((quantitativ* NEAR/5 overview*):ti,ab) OR ((quantitativ* NEAR/5 review*):ti,ab) OR ((quantitativ* NEAR/5 synthesi*):ti,ab) OR ((research NEAR/5 integration):ti,ab) OR ((systematic* NEAR/5 overview*):ti,ab) OR ((systematic* NEAR/5 review*):ti,ab))

Eligibility criteria:

- Inclusion criteria: ***A) Feasibility*** – addressed whether the procedure could be implemented, or if there were substantial barriers to overcome. Keywords included effectiveness; efficacy; facilitative; usability; barriers; difficulty; hurdles; impedes; impediment; limiting; obstacle. ***B) Population:*** Patients/ Caregivers/ Physicians/ stakeholders related to low back pain. ***C) Study Type:*** Primary studies.
- Exclusion criteria: None.

4a) Acceptability

Database searched: PubMed and Embase

Search period: 01 April 2017 to 28 April 2022

Search strategy used:

PubMed: ((("Patient Acceptance of Health Care"[Mesh] OR acceptab*[tiab] OR barrier*[tiab] OR facilitator*[tiab]) AND (("Low Back Pain"[Mesh] OR "Sciatica"[Mesh] OR "Low Backache*"[tiab] OR "Lower Backache*"[tiab] OR Lumbago[tiab] OR Sciatic* [tiab] OR (("Lumbar Vertebrae"[Mesh] OR "Lumbosacral Region"[Mesh] OR "Low Back"[tiab] OR "Lower Back"[tiab] OR Lumbar[tiab] OR Lumbosacral[tiab] OR "Lumbo-sacral"[tiab]) AND ("Pain"[Mesh] OR Ache*[tiab] OR Aching[tiab] OR Pain*[tiab])) OR (("Radiculopathy"[Mesh] OR "Radicular Pain"[tiab] OR Radiculitides[tiab] OR Radiculitis[tiab] OR Radiculopath*[tiab] OR (("Spinal Nerve Roots"[Mesh] OR "Nerve Root"[tiab]) AND ("Inflammation"[Mesh] OR "Pain"[Mesh] OR Ache*[tiab] OR Aching[tiab] OR Avulsion*[tiab] OR Compress*[tiab] OR Disorder*[tiab] OR Entrap*[tiab] OR Imping*[tiab] OR Inflam*[tiab] OR Irritat*[tiab] OR Pinch*[tiab] OR Trap*[tiab]))) AND ("Low Back Pain"[Mesh] OR "Lumbar Vertebrae"[Mesh] OR "Lumbosacral Region"[Mesh] OR Low[tiab] OR Lower[tiab] OR Lumbar[tiab] OR Lumbosacral[tiab] OR "Lumbo-sacral"[tiab]))))) AND ((("Meta-Analysis"[Publication Type] OR "Systematic Review"[Publication Type] OR "Systematic Reviews as Topic"[Mesh] OR "Systematic

review*"[ti]) AND (2017/4/1:3000/12/31[pdat]) AND english[Filter]))) NOT (("Comment"[Publication Type] OR "Editorial"[Publication Type] OR "Ephemera"[Publication Type] OR "Letter"[Publication Type] OR "Newspaper Article"[Publication Type] OR "News"[Publication Type] OR comment*[ti] OR protocol*[ti]))

Embase: ('patient attitude'/exp OR acceptab*:ti,ab OR barrier*:ti,ab OR facilitator*:ti,ab) AND ('low back pain'/exp OR 'sciatica'/exp OR 'low backache*':ti,ab OR 'lower backache*':ti,ab OR lumbago:ti,ab OR sciatic*:ti,ab OR (('lumbar vertebra'/exp OR 'lumbosacral region'/exp OR 'low back':ti,ab OR 'lower back':ti,ab OR lumbar:ti,ab OR lumbosacral:ti,ab OR 'lumbo-sacral':ti,ab) AND ('pain'/exp OR ache*:ti,ab OR aching:ti,ab OR pain*:ti,ab)) OR (('radicular pain'/exp OR 'radiculopathy'/exp OR 'radicular pain':ti,ab OR radiculitides:ti,ab OR radiculitis:ti,ab OR radiculopath*:ti,ab OR (('nerve root'/exp OR 'nerve root':ti,ab) AND ('inflammation'/exp OR 'pain'/exp OR ache*:ti,ab OR aching:ti,ab OR avulsion*:ti,ab OR compress*:ti,ab OR disorder*:ti,ab OR entrap*:ti,ab OR imping*:ti,ab OR inflam*:ti,ab OR irritat*:ti,ab OR pinch*:ti,ab OR trap*:ti,ab))) AND ('low back pain'/exp OR 'lumbar vertebra'/exp OR 'lumbosacral region'/exp OR low:ti,ab OR lower:ti,ab OR lumbar:ti,ab OR lumbosacral:ti,ab OR 'lumbo-sacral':ti,ab))) AND ('meta analysis'/exp OR 'meta analysis topic'/exp OR 'meta analysis (topic)'/exp OR 'systematic review'/exp OR 'systematic review topic'/exp OR 'systematic review (topic)'/exp OR 'systematic review$':ti) AND [1-4-2017]/sd NOT [01-01-3000]/sd AND [english]/lim NOT ('abstract report'/exp OR 'conference paper'/exp OR 'editorial'/exp OR 'letter'/exp OR 'note'/exp OR [conference abstract]/lim OR [conference paper]/lim OR [conference review]/lim OR [editorial]/lim OR [letter]/lim OR [note]/lim OR [short survey]/lim)

Eligibility criteria:

- Inclusion criteria: ***A) Acceptability*** – was defined as are key stakeholders likely to find the procedure acceptable (given the relative importance they attach to the desirable and undesirable consequences of the option; the timing of the benefits, harms, and costs; and their moral values). Keywords included attitude to health; acceptability; adherence; agreement; attitude; belief; compliance; collaboration; cooperation; empower; empowerment; experience; motivation; negotiation; participation; partnership; perception; perspective; reinforcement; views; willing; cooperative behavior; patient-provider agreement; shared; joint; informed; collaborative decision making; involved or participatory choice or decision making. ***B) Population:*** Patients/ Caregivers/ Physicians/ stakeholders related to low back pain. ***C) Study Type:*** Systematic reviews.
- Exclusion criteria: None.

4b) Acceptability with intervention

Database searched: PubMed and Embase

Search period: Not applied

PubMed: (((("Patient Acceptance of Health Care"[Mesh] OR acceptab*[tiab] OR barrier*[tiab] OR facilitator*[tiab]) AND ("Low Back Pain"[Mesh] OR "Sciatica"[Mesh] OR "Low Backache*"[tiab] OR "Lower Backache*"[tiab] OR Lumbago[tiab] OR Sciatic* [tiab] OR "Low Back pain"[tiab])) AND ("Pain Management"[Majr] OR "Diagnostic Imaging"[Majr] OR "Risk Assessment"[Majr] OR "Drug Therapy"[Majr] OR "drug therapy" [Subheading] OR "Return to Work"[Majr] OR "Psychotherapy"[Majr] OR "Pathology"[Majr] OR "Radiculopathy"[Majr] OR "Neurosciences/education"[Majr] OR "Denervation"[Majr] OR "Decompression"[Majr] OR Imaging[tiab] OR "stratified management"[tiab] OR X-ray[tiab] OR MRI[tiab] OR Radiography[tiab] OR Magnetic resonance imaging[tiab] OR "risk assessment tool*"[tiab] OR "pharmacological treatment"[tiab] OR return-to-work[tiab] OR Psychological therap*[tiab] OR epidural injection*[tiab] OR concordant pathology[tiab] OR radicular symptom*[tiab] OR "pain neuroscience education"[tiab] OR "radiofrequency denervation"[tiab] OR "spinal decompression"[tiab])) AND (English[Filter])) NOT (("address"[Publication Type] OR "autobiography"[Publication Type] OR "bibliography"[Publication Type] OR "biography"[Publication Type] OR "Book Illustrations"[Publication Type] OR "Case Reports"[Publication Type] OR "Comment"[Publication

Type] OR "congress"[Publication Type] OR "consensus development conference"[Publication Type] OR "consensus development conference, nih"[Publication Type] OR "dictionary"[Publication Type] OR "directory"[Publication Type] OR "editorial"[Publication Type] OR "Expression of Concern"[Publication Type] OR "Guideline"[Publication Type] OR "interactive tutorial"[Publication Type] OR "interview"[Publication Type] OR "lecture"[Publication Type] OR "legal case"[Publication Type] OR "legislation"[Publication Type] OR "letter"[Publication Type] OR "Meta-Analysis"[Publication Type] OR "news"[Publication Type] OR "newspaper article"[Publication Type] OR "overall"[Publication Type] OR "patient education handout"[Publication Type] OR "periodical index"[Publication Type] OR "personal narrative"[Publication Type] OR "portrait"[Publication Type] OR "Review"[Publication Type] OR "Scientific Integrity Review"[Publication Type] OR "Systematic Review"[Publication Type] OR "hascommenton"[All Fields] OR "Cartoons as Topic"[Mesh] OR "Meta-Analysis as Topic"[Mesh] OR "Review Literature as Topic"[Mesh] OR "Systematic Reviews as Topic"[Mesh] OR "case report*"[tiab] OR "case series"[tiab] OR "integrative research review*"[tiab] OR "integrative review*"[tiab] OR "literature review"[tiab] OR meta-analys*[tiab] OR "meta analys*"[tiab] OR metaanalys*[tiab] OR "narrative review"[tiab] OR "research integration"[tiab] OR "scoping review"[tiab] OR ((methodologic*[tiab] OR quantitative*[tiab] OR systematic*[tiab]) AND (overview*[tiab] OR review*[tiab] OR synthesis*[tiab]))))

Embase: ('patient attitude'/exp OR acceptab*:ti,ab OR barrier*:ti,ab OR facilitator*:ti,ab) AND ('low back pain'/exp OR 'sciatica'/exp OR 'low backache*':ti,ab OR 'lower backache*':ti,ab OR lumbago:ti,ab OR sciatic*:ti,ab OR 'low back pain':ti,ab) AND ('disease management'/mj OR 'radiodiagnosis'/mj OR 'risk assessment'/mj OR 'drug therapy'/mj OR 'return to work'/mj OR 'psychotherapy'/mj OR 'epidural drug administration'/mj OR 'pathology'/mj OR 'radiculopathy'/mj OR 'neuroscience'/mj OR 'denervation'/mj OR 'decompression'/mj OR imaging:ti,ab OR 'stratified management':ti,ab OR 'x ray':ti,ab OR mri:ti,ab OR radiography:ti,ab OR 'magnetic resonance imaging':ti,ab OR 'risk assessment tool*':ti,ab OR 'pharmacological treatment':ti,ab OR 'return to work' OR 'psychological therap':ti,ab OR 'epidural injection*':ti,ab OR 'concordant pathology':ti,ab OR 'radicular symptom*':ti,ab OR 'pain neuroscience education':ti,ab OR 'radiofrequency denervation':ti,ab OR 'spinal decompression':ti,ab) AND ([english]/lim) NOT (('abstract report'/exp OR 'animal experiment'/exp OR 'book'/exp OR 'case finding'/exp OR 'case report'/exp OR 'case study'/exp OR 'conference paper'/exp OR 'editorial'/exp OR 'feasibility study'/exp OR 'in vitro study'/exp) AND 'letter'/exp OR 'meta analysis'/exp OR 'meta analysis topic'/exp OR 'meta analysis (topic)'/exp OR 'note'/exp OR 'practice guideline'/exp OR 'review'/exp OR 'systematic review'/exp OR 'systematic review topic'/exp OR 'systematic review (topic)'/exp OR 'veterinary clinical trial'/exp OR 'veterinary study'/exp OR [conference abstract]/lim OR [conference paper]/lim OR [conference review]/lim OR [editorial]/lim OR [letter]/lim OR [note]/lim OR [short survey]/lim OR 'case report*':ti,ab OR 'case series':ti,ab OR 'integrative research review*':ti,ab OR 'integrative review*':ti,ab OR 'literature review':ti,ab OR 'meta analys*':ti,ab OR metaanalys*:ti,ab OR meta*analys*:ti,ab OR 'narrative review':ti,ab OR 'research integration':ti,ab OR 'scoping review':ti,ab OR ((integrative NEAR/5 research NEAR/5 review*):ti,ab) OR ((methodologic* NEAR/5 overview*):ti,ab) OR ((methodologic* NEAR/5 review*):ti,ab) OR ((quantitativ* NEAR/5 overview*):ti,ab) OR ((quantitativ* NEAR/5 review*):ti,ab) OR ((quantitativ* NEAR/5 synthesi*):ti,ab) OR ((research NEAR/5 integration):ti,ab) OR ((systematic* NEAR/5 overview*):ti,ab) OR ((systematic* NEAR/5 review*):ti,ab))

Eligibility criteria:

- Inclusion criteria: ***A) Acceptability*** – was defined as are key stakeholders likely to find the procedure acceptable (given the relative importance they attach to the desirable and undesirable consequences of the option; the timing of the benefits, harms, and costs; and their moral values). Keywords included attitude to health; acceptability; adherence; agreement; attitude; belief; compliance; collaboration; cooperation; empower; empowerment; experience; motivation; negotiation; participation; partnership; perception; perspective; reinforcement; views; willing; cooperative behavior; patient-provider agreement; shared; joint; informed; collaborative decision making; involved or participatory choice or decision making. ***B) Population:*** Patients/ Caregivers/ Physicians/ stakeholders related to low back pain. ***C) Study Type:*** Primary studies.
- Exclusion criteria: None.

5a) Implementation

Database searched: PubMed and Embase

Search period: 01 April 2017 to 28 April 2022

Search strategy used:

PubMed: (((((("Clinical Protocols"[Majr] OR "Consensus"[Majr] OR "Critical Pathways"[Majr] OR "Guideline"[Publication Type] OR "Guidelines as Topic"[Majr] OR "Health Planning Guidelines"[Majr] OR advice[tiab] OR advise*[tiab] OR consensus[tiab] OR frame-work*[tiab] OR framework*[tiab] OR guidance*[tiab] OR guideline*[tiab] OR policies[tiab] OR policy[tiab] OR protocol*[tiab] OR recommend*[tiab] OR standard*[tiab] OR statement*[tiab])) AND ((accordance[tiab] OR adhere*[tiab] OR adopt*[tiab] OR aware*[tiab] OR barrier*[tiab] OR compliance*[tiab] OR complies[tiab] OR comply*[tiab] OR concordance[tiab] OR disseminat*[tiab] OR facilitat*[tiab] OR implement*[tiab] OR incorporat*[tiab] OR integrat*[tiab] OR spread*[tiab] OR sustain*[tiab] OR takeup*[tiab] OR take-up*[tiab] OR uptake*[tiab] OR up-take*[tiab]))) OR (("Guideline Adherence"[Majr] OR "Guideline Implementation"[tiab]))) AND (("Low Back Pain"[Mesh] OR "Sciatica"[Mesh] OR "Low Backache*"[tiab] OR "Lower Backache*"[tiab] OR Lumbago[tiab] OR Sciatic* [tiab] OR (("Lumbar Vertebrae"[Mesh] OR "Lumbosacral Region"[Mesh] OR "Low Back"[tiab] OR "Lower Back"[tiab] OR Lumbar[tiab] OR Lumbosacral[tiab] OR "Lumbo-sacral"[tiab]) AND ("Pain"[Mesh] OR Ache*[tiab] OR Aching[tiab] OR Pain*[tiab])) OR (("Radiculopathy"[Mesh] OR "Radicular Pain"[tiab] OR Radiculitides[tiab] OR Radiculitis[tiab] OR Radiculopath*[tiab] OR (("Spinal Nerve Roots"[Mesh] OR "Nerve Root"[tiab]) AND ("Inflammation"[Mesh] OR "Pain"[Mesh] OR Ache*[tiab] OR Aching[tiab] OR Avulsion*[tiab] OR Compress*[tiab] OR Disorder*[tiab] OR Entrap*[tiab] OR Imping*[tiab] OR Inflam*[tiab] OR Irritat*[tiab] OR Pinch*[tiab] OR Trap*[tiab]))) AND ("Low Back Pain"[Mesh] OR "Lumbar Vertebrae"[Mesh] OR "Lumbosacral Region"[Mesh] OR Low[tiab] OR Lower[tiab] OR Lumbar[tiab] OR Lumbosacral[tiab] OR "Lumbo-sacral"[tiab]))))) AND ((("Meta-Analysis"[Publication Type] OR "Systematic Review"[Publication Type] OR "Systematic Reviews as Topic"[Mesh] OR "Systematic review*"[ti]) AND (2017/4/1:3000/12/31[pdat]) AND english[Filter]))) NOT (("Comment"[Publication Type] OR "Editorial"[Publication Type] OR "Ephemera"[Publication Type] OR "Letter"[Publication Type] OR "Newspaper Article"[Publication Type] OR "News"[Publication Type] OR comment*[ti] OR protocol*[ti]))

Embase: ('clinical protocol'/mj OR 'consensus'/mj OR 'clinical pathway'/mj OR 'guideline'/mj OR 'health care planning'/mj OR ((advice OR advise* OR consensus OR frame-work* OR framework* OR guidance* OR guideline* OR policies OR policy OR protocol* OR recommend* OR standard* OR statement*):ti,ab)) AND (((accordance OR adhere* OR adopt* OR aware* OR barrier* OR compliance* OR complies OR comply* OR concordance OR disseminat* OR facilitat* OR implement* OR incorporat* OR integrat* OR spread* OR sustain* OR takeup* OR take-up* OR uptake* OR up-take*):ti,ab)) OR (('protocol compliance'/mj OR 'Guideline Implementation':ti,ab)) AND (('low back pain'/exp OR 'sciatica'/exp OR (("Low Backache*" OR "Lower Backache*" OR Lumbago OR Sciatic*):ti,ab) OR (('lumbar vertebra'/exp OR 'lumbosacral region'/exp OR (("Low Back" OR "Lower Back" OR Lumbar OR Lumbosacral OR "Lumbo-sacral"):ti,ab)) AND ('pain'/exp OR ((Ache* OR Aching OR Pain*):ti,ab))) OR (('radicular pain'/exp OR 'radiculopathy'/exp OR (("Radicular Pain" OR Radiculitides OR Radiculitis OR Radiculopath*):ti,ab) OR (('nerve root'/exp OR "Nerve Root":ti,ab) AND ('inflammation'/exp OR 'pain'/exp OR ((Ache* OR Aching OR Avulsion* OR Compress* OR Disorder* OR Entrap* OR Imping* OR Inflam* OR Irritat* OR Pinch* OR Trap*):ti,ab)))) AND ('low back pain'/exp OR 'lumbar vertebra'/exp OR 'lumbosacral region'/exp OR ((Low OR Lower OR Lumbar OR Lumbosacral OR "Lumbo-sacral"):ti,ab))))) AND ((('meta analysis'/exp OR 'meta analysis topic'/exp OR 'meta analysis (topic)'/exp OR 'systematic review'/exp OR 'systematic review topic'/exp OR 'systematic review (topic)'/exp OR 'systematic review$':ti) AND ([1-4-2017]/sd NOT [01-01-3000]/sd) AND [english]/lim)) NOT (('abstract report'/exp OR 'conference paper'/exp OR 'editorial'/exp OR 'letter'/exp OR 'note'/exp OR [conference abstract]/lim OR [conference paper]/lim OR [conference review]/lim OR [editorial]/lim OR [letter]/lim OR [note]/lim OR [short survey]/lim))

Eligibility criteria:

- Inclusion criteria: ***A) Implementation*** – interventions was defined as any intervention aiming to improve the uptake of guideline recommendations in practice. Keywords we are looking for: accordance; adherence; adopt; aware; concordance; barrier; compliance; comply; disseminate; facilitate; implement; incorporate; integrate; spread; sustain; take-up; uptake; diffusion of innovation; health plan implementation; information dissemination; guideline adherence; organizational innovation; guideline implementation; health plan implementation; information dissemination; information distribution; innovation diffusion; institutional implementation; policy implementation; protocol implementation; clinical protocols; consensus; critical pathways; guideline; advice; framework; guidance; policies; recommendation; standard; statement. ***B) Population:*** Patients/ Caregivers/ Physicians/ stakeholders related to low back pain. ***C) Study Type:*** Systematic reviews.
- Exclusion criteria: None.

5b) Implementation with intervention

Database searched: PubMed and Embase

Search period: Not applied

PubMed: ((((((("Clinical Protocols"[Majr] OR "Consensus"[Majr] OR "Critical Pathways"[Majr] OR "Guideline"[Publication Type] OR "Guidelines as Topic"[Majr] OR "Health Planning Guidelines"[Majr] OR advice[tiab] OR advise*[tiab] OR consensus[tiab] OR frame-work*[tiab] OR framework*[tiab] OR guidance*[tiab] OR guideline*[tiab] OR policies[tiab] OR policy[tiab] OR protocol*[tiab] OR recommend*[tiab] OR standard*[tiab] OR statement*[tiab])) AND ((accordance[tiab] OR adhere*[tiab] OR adopt*[tiab] OR aware*[tiab] OR barrier*[tiab] OR compliance*[tiab] OR complies[tiab] OR comply*[tiab] OR concordance[tiab] OR disseminat*[tiab] OR facilitat*[tiab] OR implement*[tiab] OR incorporat*[tiab] OR integrat*[tiab] OR spread*[tiab] OR sustain*[tiab] OR takeup*[tiab] OR take-up*[tiab] OR uptake*[tiab] OR up-take*[tiab]))) OR (("Guideline Adherence"[Majr] OR "Guideline Implementation"[tiab]))) AND ("Low Back Pain"[Mesh] OR "Sciatica"[Mesh] OR "Low Backache*"[tiab] OR "Lower Backache*"[tiab] OR Lumbago[tiab] OR Sciatic* [tiab] OR "Low Back pain"[tiab])) AND ("Pain Management"[Majr] OR "Diagnostic Imaging"[Majr] OR "Risk Assessment"[Majr] OR "Drug Therapy"[Majr] OR "drug therapy" [Subheading] OR "Return to Work"[Majr] OR "Psychotherapy"[Majr] OR "Pathology"[Majr] OR "Radiculopathy"[Majr] OR "Neurosciences/education"[Majr] OR "Denervation"[Majr] OR "Decompression"[Majr] OR Imaging[tiab] OR "stratified management"[tiab] OR X-ray[tiab] OR MRI[tiab] OR Radiography[tiab] OR Magnetic resonance imaging[tiab] OR "risk assessment tool*"[tiab] OR "pharmacological treatment"[tiab] OR return-to-work[tiab] OR Psychological therap*[tiab] OR epidural injection*[tiab] OR concordant pathology[tiab] OR radicular symptom*[tiab] OR "pain neuroscience education"[tiab] OR "radiofrequency denervation"[tiab] OR "spinal decompression"[tiab])) AND (English[Filter])) NOT (("address"[Publication Type] OR "autobiography"[Publication Type] OR "bibliography"[Publication Type] OR "biography"[Publication Type] OR "Book Illustrations"[Publication Type] OR "Case Reports"[Publication Type] OR "Comment"[Publication Type] OR "congress"[Publication Type] OR "consensus development conference"[Publication Type] OR "consensus development conference, nih"[Publication Type] OR "dictionary"[Publication Type] OR "directory"[Publication Type] OR "editorial"[Publication Type] OR "Expression of Concern"[Publication Type] OR "Guideline"[Publication Type] OR "interactive tutorial"[Publication Type] OR "interview"[Publication Type] OR "lecture"[Publication Type] OR "legal case"[Publication Type] OR "legislation"[Publication Type] OR "letter"[Publication Type] OR "Meta-Analysis"[Publication Type] OR "news"[Publication Type] OR "newspaper article"[Publication Type] OR "overall"[Publication Type] OR "patient education handout"[Publication Type] OR "periodical index"[Publication Type] OR "personal narrative"[Publication Type] OR "portrait"[Publication Type] OR "Review"[Publication Type] OR "Scientific Integrity Review"[Publication Type] OR "Systematic Review"[Publication Type] OR "hascommenton"[All Fields] OR "Cartoons as Topic"[Mesh] OR "Meta-Analysis as Topic"[Mesh] OR "Review Literature as Topic"[Mesh] OR "Systematic Reviews as Topic"[Mesh] OR "case report*"[tiab] OR "case series"[tiab] OR "integrative research review*"[tiab] OR "integrative review*"[tiab] OR "literature

review"[tiab] OR meta-analys*[tiab] OR "meta analys*"[tiab] OR metaanalys*[tiab] OR "narrative review"[tiab] OR "research integration"[tiab] OR "scoping review"[tiab] OR ((methodologic*[tiab] OR quantitative*[tiab] OR systematic*[tiab]) AND (overview*[tiab] OR review*[tiab] OR synthesis*[tiab]))))

Embase: ('clinical protocol'/mj OR 'consensus'/mj OR 'clinical pathway'/mj OR 'guideline'/mj OR 'health care planning'/mj OR ((advice OR advise* OR consensus OR frame-work* OR framework* OR guidance* OR guideline* OR policies OR policy OR protocol* OR recommend* OR standard* OR statement*):ti,ab)) AND (((accordance OR adhere* OR adopt* OR aware* OR barrier* OR compliance* OR complies OR comply* OR concordance OR disseminat* OR facilitat* OR implement* OR incorporat* OR integrat* OR spread* OR sustain* OR takeup* OR take-up* OR uptake* OR up-take*):ti,ab)) OR (('protocol compliance'/mj OR 'Guideline Implementation':ti,ab)) AND ('low back pain'/exp OR 'sciatica'/exp OR 'low backache*':ti,ab OR 'lower backache*':ti,ab OR lumbago:ti,ab OR sciatic*:ti,ab OR 'low back pain':ti,ab) AND ('disease management'/mj OR 'radiodiagnosis'/mj OR 'risk assessment'/mj OR 'drug therapy'/mj OR 'return to work'/mj OR 'psychotherapy'/mj OR 'epidural drug administration'/mj OR 'pathology'/mj OR 'radiculopathy'/mj OR 'neuroscience'/mj OR 'denervation'/mj OR 'decompression'/mj OR imaging:ti,ab OR 'stratified management':ti,ab OR 'x ray':ti,ab OR mri:ti,ab OR radiography:ti,ab OR 'magnetic resonance imaging':ti,ab OR 'risk assessment tool*':ti,ab OR 'pharmacological treatment':ti,ab OR 'return to work' OR 'psychological therap':ti,ab OR 'epidural injection*':ti,ab OR 'concordant pathology':ti,ab OR 'radicular symptom*':ti,ab OR 'pain neuroscience education':ti,ab OR 'radiofrequency denervation':ti,ab OR 'spinal decompression':ti,ab) AND ([english]/lim) NOT (('abstract report'/exp OR 'animal experiment'/exp OR 'book'/exp OR 'case finding'/exp OR 'case report'/exp OR 'case study'/exp OR 'conference paper'/exp OR 'editorial'/exp OR 'feasibility study'/exp OR 'in vitro study'/exp) AND 'letter'/exp OR 'meta analysis'/exp OR 'meta analysis topic'/exp OR 'meta analysis (topic)'/exp OR 'note'/exp OR 'practice guideline'/exp OR 'review'/exp OR 'systematic review'/exp OR 'systematic review topic'/exp OR 'systematic review (topic)'/exp OR 'veterinary clinical trial'/exp OR 'veterinary study'/exp OR [conference abstract]/lim OR [conference paper]/lim OR [conference review]/lim OR [editorial]/lim OR [letter]/lim OR [note]/lim OR [short survey]/lim OR 'case report*':ti,ab OR 'case series':ti,ab OR 'integrative research review*':ti,ab OR 'integrative review*':ti,ab OR 'literature review':ti,ab OR 'meta analys*':ti,ab OR metaanalys*:ti,ab OR meta*analys*:ti,ab OR 'narrative review':ti,ab OR 'research integration':ti,ab OR 'scoping review':ti,ab OR ((integrative NEAR/5 research NEAR/5 review*):ti,ab) OR ((methodologic* NEAR/5 overview*):ti,ab) OR ((methodologic* NEAR/5 review*):ti,ab) OR ((quantitativ* NEAR/5 overview*):ti,ab) OR ((quantitativ* NEAR/5 review*):ti,ab) OR ((quantitativ* NEAR/5 synthesi*):ti,ab) OR ((research NEAR/5 integration):ti,ab) OR ((systematic* NEAR/5 overview*):ti,ab) OR ((systematic* NEAR/5 review*):ti,ab))

Eligibility criteria:

- Inclusion criteria: ***A) Implementation*** – interventions was defined as any intervention aiming to improve the uptake of guideline recommendations in practice. Keywords we are looking for: accordance; adherence; adopt; aware; concordance; barrier; compliance; comply; disseminate; facilitate; implement; incorporate; integrate; spread; sustain; take-up; uptake; diffusion of innovation; health plan implementation; information dissemination; guideline adherence; organizational innovation; guideline implementation; health plan implementation; information dissemination; information distribution; innovation diffusion; institutional implementation; policy implementation; protocol implementation; clinical protocols; consensus; critical pathways; guideline; advice; framework; guidance; policies; recommendation; standard; statement. ***B) Population:*** Patients/ Caregivers/ Physicians/ stakeholders related to low back pain. ***C) Study Type:*** Primary studies.
- Exclusion criteria: None.

6a) Cost information

Database searched: PubMed and Embase

Search period: 01 April 2017 to 28 April 2022

PubMed: (((("Low Back Pain"[Mesh] OR "Sciatica"[Mesh] OR "Low Backache*"[tiab] OR "Lower Backache*"[tiab] OR Lumbago[tiab] OR Sciatic* [tiab] OR (("Lumbar Vertebrae"[Mesh] OR "Lumbosacral Region"[Mesh] OR "Low Back"[tiab] OR "Lower Back"[tiab] OR Lumbar[tiab] OR Lumbosacral[tiab] OR "Lumbo-sacral"[tiab]) AND ("Pain"[Mesh] OR Ache*[tiab] OR Aching[tiab] OR Pain*[tiab])) OR (("Radiculopathy"[Mesh] OR "Radicular Pain"[tiab] OR Radiculitides[tiab] OR Radiculitis[tiab] OR Radiculopath*[tiab] OR (("Spinal Nerve Roots"[Mesh] OR "Nerve Root"[tiab]) AND ("Inflammation"[Mesh] OR "Pain"[Mesh] OR Ache*[tiab] OR Aching[tiab] OR Avulsion*[tiab] OR Compress*[tiab] OR Disorder*[tiab] OR Entrap*[tiab] OR Imping*[tiab] OR Inflam*[tiab] OR Irritat*[tiab] OR Pinch*[tiab] OR Trap*[tiab]))) AND ("Low Back Pain"[Mesh] OR "Lumbar Vertebrae"[Mesh] OR "Lumbosacral Region"[Mesh] OR Low[tiab] OR Lower[tiab] OR Lumbar[tiab] OR Lumbosacral[tiab] OR "Lumbo-sacral"[tiab])))) AND (((("Economics"[Mesh:NoExp] OR "Costs and Cost Analysis"[Mesh] OR "Value of Life"[Mesh:NoExp] OR "Economics, Hospital"[Mesh] OR "Economics, Medical"[Mesh] OR "Economics, Nursing"[Mesh:NoExp] OR "Economics, Pharmaceutical"[Mesh] OR "Fees and Charges"[Mesh] OR "Budgets"[Mesh]) OR (((low[tiab] OR high[tiab] OR health*[tiab] OR estimate*[tiab] OR variable*[tiab] OR unit[tiab]) AND cost*[tiab]))) OR (fiscal[tiab] OR funding[tiab] OR financial[tiab] OR finance[tiab])) OR (economic*[tiab] OR pharmacoeconomic*[tiab] OR price*[tiab] OR pricing[tiab]))) AND ((("Meta-Analysis"[Publication Type] OR "Systematic Review"[Publication Type] OR "Systematic Reviews as Topic"[Mesh] OR "Systematic review*"[ti]) AND (2017/4/1:3000/12/31[pdat]) AND english[Filter]))) NOT (("Comment"[Publication Type] OR "Editorial"[Publication Type] OR "Ephemera"[Publication Type] OR "Letter"[Publication Type] OR "Newspaper Article"[Publication Type] OR "News"[Publication Type] OR comment*[ti] OR protocol*[ti]))

Embase: ('low back pain'/exp OR 'sciatica'/exp OR (("Low Backache*" OR "Lower Backache*" OR Lumbago OR Sciatic*):ti,ab) OR (('lumbar vertebra'/exp OR 'lumbosacral region'/exp OR (("Low Back" OR "Lower Back" OR Lumbar OR Lumbosacral OR "Lumbo-sacral"):ti,ab)) AND ('pain'/exp OR ((Ache* OR Aching OR Pain*):ti,ab))) OR (('radicular pain'/exp OR 'radiculopathy'/exp OR (("Radicular Pain" OR Radiculitides OR Radiculitis OR Radiculopath*):ti,ab) OR (('nerve root'/exp OR "Nerve Root":ti,ab) AND ('inflammation'/exp OR 'pain'/exp OR ((Ache* OR Aching OR Avulsion* OR Compress* OR Disorder* OR Entrap* OR Imping* OR Inflam* OR Irritat* OR Pinch* OR Trap*):ti,ab)))) AND ('low back pain'/exp OR 'lumbar vertebra'/exp OR 'lumbosacral region'/exp OR ((Low OR Lower OR Lumbar OR Lumbosacral OR "Lumbo-sacral"):ti,ab)))) AND ('socioeconomics'/de OR 'economic evaluation'/exp OR 'economic aspect'/de OR 'financial management'/de OR 'health care cost'/de OR 'health care financing'/de OR 'health economics'/de OR 'hospital cost'/de OR 'pharmacoeconomics'/de OR 'drug cost'/de OR fiscal:ti,ab OR financial:ti,ab OR finance:ti,ab OR funding:ti,ab OR ((cost NEAR/3 (estimate* OR variable* OR unit)):ti,ab)) AND ((('meta analysis'/exp OR 'meta analysis topic'/exp OR 'meta analysis (topic)'/exp OR 'systematic review'/exp OR 'systematic review topic'/exp OR 'systematic review (topic)'/exp OR 'systematic review$':ti) AND ([1-4-2017]/sd NOT [01-01-3000]/sd) AND [english]/lim)) NOT (('abstract report'/exp OR 'conference paper'/exp OR 'editorial'/exp OR 'letter'/exp OR 'note'/exp OR [conference abstract]/lim OR [conference paper]/lim OR [conference review]/lim OR [editorial]/lim OR [letter]/lim OR [note]/lim OR [short survey]/lim))

Eligibility criteria:

- Inclusion criteria: ***A) Concepts:*** Low back pain and cost of the interventions shared. ***B) Types of studies:*** Systematic reviews.
- Exclusion criteria: None.

6b) Cost information with intervention

Database searched: PubMed and Embase

Search period: Not applied

PubMed: (((("Low Back Pain"[Mesh] OR "Sciatica"[Mesh] OR "Low Backache*"[tiab] OR "Lower Backache*"[tiab] OR Lumbago[tiab] OR Sciatic* [tiab] OR "Low Back pain"[tiab]) AND

(((("Economics"[Mesh:NoExp] OR "Costs and Cost Analysis"[Mesh] OR "Value of Life"[Mesh:NoExp] OR "Economics, Hospital"[Mesh] OR "Economics, Medical"[Mesh] OR "Economics, Nursing"[Mesh:NoExp] OR "Economics, Pharmaceutical"[Mesh] OR "Fees and Charges"[Mesh] OR "Budgets"[Mesh]) OR (((low[tiab] OR high[tiab] OR health*[tiab] OR estimate*[tiab] OR variable*[tiab] OR unit[tiab]) AND cost*[tiab]))) OR (fiscal[tiab] OR funding[tiab] OR financial[tiab] OR finance[tiab])) OR (economic*[tiab] OR pharmacoeconomic*[tiab] OR price*[tiab] OR pricing[tiab]))) AND ("Pain Management"[Majr] OR "Diagnostic Imaging"[Majr] OR "Risk Assessment"[Majr] OR "Drug Therapy"[Majr] OR "drug therapy" [Subheading] OR "Return to Work"[Majr] OR "Psychotherapy"[Majr] OR "Pathology"[Majr] OR "Radiculopathy"[Majr] OR "Neurosciences/education"[Majr] OR "Denervation"[Majr] OR "Decompression"[Majr] OR Imaging[tiab] OR "stratified management"[tiab] OR X-ray[tiab] OR MRI[tiab] OR Radiography[tiab] OR Magnetic resonance imaging[tiab] OR "risk assessment tool*"[tiab] OR "pharmacological treatment"[tiab] OR return-to-work[tiab] OR Psychological therap*[tiab] OR epidural injection*[tiab] OR concordant pathology[tiab] OR radicular symptom*[tiab] OR "pain neuroscience education"[tiab] OR "radiofrequency denervation"[tiab] OR "spinal decompression"[tiab])) AND (English[Filter])) NOT (("address"[Publication Type] OR "autobiography"[Publication Type] OR "bibliography"[Publication Type] OR "biography"[Publication Type] OR "Book Illustrations"[Publication Type] OR "Case Reports"[Publication Type] OR "Comment"[Publication Type] OR "congress"[Publication Type] OR "consensus development conference"[Publication Type] OR "consensus development conference, nih"[Publication Type] OR "dictionary"[Publication Type] OR "directory"[Publication Type] OR "editorial"[Publication Type] OR "Expression of Concern"[Publication Type] OR "Guideline"[Publication Type] OR "interactive tutorial"[Publication Type] OR "interview"[Publication Type] OR "lecture"[Publication Type] OR "legal case"[Publication Type] OR "legislation"[Publication Type] OR "letter"[Publication Type] OR "Meta-Analysis"[Publication Type] OR "news"[Publication Type] OR "newspaper article"[Publication Type] OR "overall"[Publication Type] OR "patient education handout"[Publication Type] OR "periodical index"[Publication Type] OR "personal narrative"[Publication Type] OR "portrait"[Publication Type] OR "Review"[Publication Type] OR "Scientific Integrity Review"[Publication Type] OR "Systematic Review"[Publication Type] OR "hascommenton"[All Fields] OR "Cartoons as Topic"[Mesh] OR "Meta-Analysis as Topic"[Mesh] OR "Review Literature as Topic"[Mesh] OR "Systematic Reviews as Topic"[Mesh] OR "case report*"[tiab] OR "case series"[tiab] OR "integrative research review*"[tiab] OR "integrative review*"[tiab] OR "literature review"[tiab] OR meta-analys*[tiab] OR "meta analys*"[tiab] OR metaanalys*[tiab] OR "narrative review"[tiab] OR "research integration"[tiab] OR "scoping review"[tiab] OR ((methodologic*[tiab] OR quantitative*[tiab] OR systematic*[tiab]) AND (overview*[tiab] OR review*[tiab] OR synthesis*[tiab]))))

Embase: 'low back pain'/exp OR 'sciatica'/exp OR 'low backache*':ti,ab OR 'lower backache*':ti,ab OR lumbago:ti,ab OR sciatic*:ti,ab OR 'low back pain':ti,ab AND ('socioeconomics'/de OR 'economic evaluation'/exp OR 'economic aspect'/de OR 'financial management'/de OR 'health care cost'/de OR 'health care financing'/de OR 'health economics'/de OR 'hospital cost'/de OR 'pharmacoeconomics'/de OR 'drug cost'/de OR fiscal:ti,ab OR financial:ti,ab OR finance:ti,ab OR funding:ti,ab OR ((cost NEAR/3 (estimate* OR variable* OR unit)):ti,ab)) AND ('disease management'/mj OR 'radiodiagnosis'/mj OR 'risk assessment'/mj OR 'drug therapy'/mj OR 'return to work'/mj OR 'psychotherapy'/mj OR 'epidural drug administration'/mj OR 'pathology'/mj OR 'radiculopathy'/mj OR 'neuroscience'/mj OR 'denervation'/mj OR 'decompression'/mj OR imaging:ti,ab OR 'stratified management':ti,ab OR 'x ray':ti,ab OR mri:ti,ab OR radiography:ti,ab OR 'magnetic resonance imaging':ti,ab OR 'risk assessment tool*':ti,ab OR 'pharmacological treatment':ti,ab OR 'return to work' OR 'psychological therap':ti,ab OR 'epidural injection*':ti,ab OR 'concordant pathology':ti,ab OR 'radicular symptom*':ti,ab OR 'pain neuroscience education':ti,ab OR 'radiofrequency denervation':ti,ab OR 'spinal decompression':ti,ab) AND ([english]/lim) NOT (('abstract report'/exp OR 'animal experiment'/exp OR 'book'/exp OR 'case finding'/exp OR 'case report'/exp OR 'case study'/exp OR 'conference paper'/exp OR 'editorial'/exp OR 'feasibility study'/exp OR 'in vitro study'/exp) AND 'letter'/exp OR 'meta analysis'/exp OR 'meta analysis topic'/exp OR 'meta analysis (topic)'/exp OR 'note'/exp OR 'practice guideline'/exp OR 'review'/exp OR 'systematic review'/exp OR 'systematic review topic'/exp OR 'systematic review (topic)'/exp OR 'veterinary clinical trial'/exp OR 'veterinary

study'/exp OR [conference abstract]/lim OR [conference paper]/lim OR [conference review]/lim OR [editorial]/lim OR [letter]/lim OR [note]/lim OR [short survey]/lim OR 'case report*':ti,ab OR 'case series':ti,ab OR 'integrative research review*':ti,ab OR 'integrative review*':ti,ab OR 'literature review':ti,ab OR 'meta analys*':ti,ab OR metaanalys*:ti,ab OR meta*analys*:ti,ab OR 'narrative review':ti,ab OR 'research integration':ti,ab OR 'scoping review':ti,ab OR ((integrative NEAR/5 research NEAR/5 review*):ti,ab) OR ((methodologic* NEAR/5 overview*):ti,ab) OR ((methodologic* NEAR/5 review*):ti,ab) OR ((quantitativ* NEAR/5 overview*):ti,ab) OR ((quantitativ* NEAR/5 review*):ti,ab) OR ((quantitativ* NEAR/5 synthesi*):ti,ab) OR ((research NEAR/5 integration):ti,ab) OR ((systematic* NEAR/5 overview*):ti,ab) OR ((systematic* NEAR/5 review*):ti,ab))

Eligibility criteria:

- Inclusion criteria: ***A) Concepts:*** Low back pain and cost of the interventions shared. ***B) Types of studies:*** Primary studies.
- Exclusion criteria: None.
