# Supplementary material for: Saudi Clinical Practice Guideline for the Assessment and Management of Low Back Pain and Sciatica in Adults
Source: J Clin Med. 2026 Jan 8;15(2):528. doi: 10.3390/jcm15020528 (PMC12842004; doi:10.3390/jcm15020528)

Supplementary Material S2: Forest Plots

This supplementary material contains the new forest plots generated using the Cochrane RevMan software for all questions with quantitative evidence synthesis.

Question 1: Should validated risk assessment tools versus no validated risk assessment tools be used for screening patients with LBP and/or sciatica who are at risk of poor outcome or delayed improvement?

Figure S1. STarT Back risk tool versus no risk tool stratification. Outcome 1: Chronic pain risk item set at 4 months to predict pain.

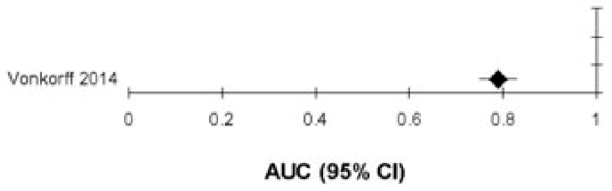

Very high risk of bias (RoB assessed using the PROBAST checklist)

- Sensitivity: 72%
- Specificity: 70%
- AUC: 0.79 (Range, 0.75-0.83)

Figure S2. STarT Back risk tool versus no risk tool stratification. Outcome 2: Low back pain perception scale (self-reported) at 12 months to predict recovery from pain.

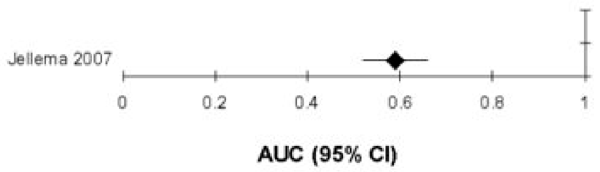

Very high risk of bias (RoB assessed using the PROBAST checklist)
Recovery: cut-off ≥ 2

- Sensitivity 80%; Specificity 27%
- AUC 0.59 (Range, 0.52-0.66)

Recovery: cut-off ≥ 4

- Sensitvity: 30%; Specificity 81%
- AUC 0.59 (Range, 0.52-0.66)

Calibration

- Intercept 0.02 (Range, 0.02-0.03)
- Slope 0.95 (Range, 0.93–0.97)

Figure S3. STarT Back risk tool versus no risk tool stratification. Outcome 3: Modified ÖREBRO at 6 months to predict problem severity.

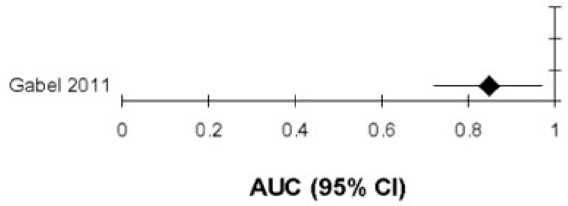

High risk of bias (Assessed using the PROBAST checklist)

- Sensitivity: 88%
- Specificity: 85.7%
- AUC: 0.88 (Range, 0.78-0.99)

Figure S4. STarT Back risk tool versus no risk tool stratification. Outcome 4: Oswestry Disability Questionnaire (ODI) at 1 month to predict functional improvement.

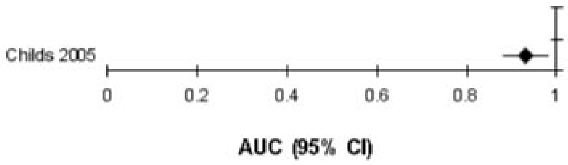

Very high risk of bias (Assessed using the PROBAST checklist)
AUC: 0.93 (Range, 0.88-0.98)

Figure S5. STarT Back risk tool versus no risk tool stratification. Outcome 5: STarT Back Screening Tool for predicting pain (as assessed using the NRS, and PGIC scale = Patient's Global Impression of Change, score 1-7).

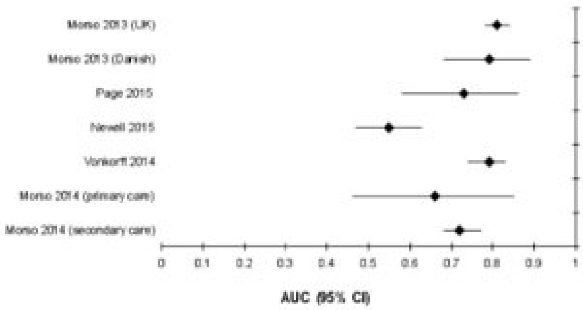

UK at 3 months

- AUC 0.68 (Range, 0.55-0.81) - Very high risk of bias

Danish translation at 3 months

- AUC 0.79 (Range, 0.68-0.89) - Very high risk of bias

At 6 months (primary care):

- AUC 0.66 (Range, 0.46-0.85) - Very high risk of bias

At 6 months (secondary care):

- AUC 0.73 (Range, 0.72-0.73) - Very high risk of bias

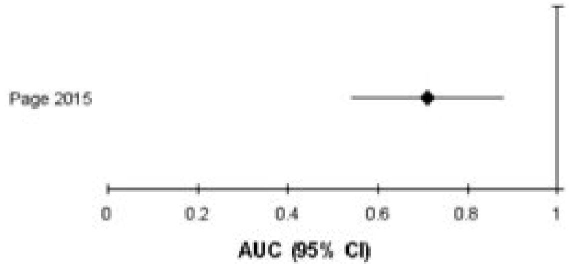

At 12 months (secondary care):

- AUC 0.71 (Range, 0.54-0.88) – High risk of bias

(RoB assessed using the PROBAST checklist)

Figure S6. STarT Back risk tool versus no risk tool stratification. Outcome 6: STarT Back Screening Tool for predicting functional improvement (as assessed using a variety of methods including self-report, ODI, RMDQ, global rating of change).

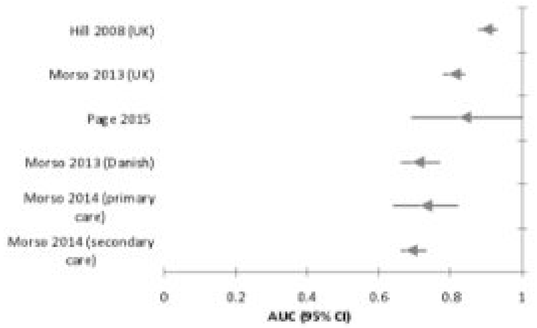

UK at 3 months

- AUC 0.81 (Range, 0.78-0.84) – Very high risk of bias

Danish translation at 3 months

- AUC 0.71 (Range, 0.66-0.77) - Very high risk of bias

At 6 months (primary care):

- AUC 0.82 (Range, 0.73-0.9) – Low risk of bias
- Sensitivity: 80.1% Specificity 65.4%

At 6 months (secondary care):

- AUC 0.77 (Range, 0.69-0.84) – Very high risk of bias

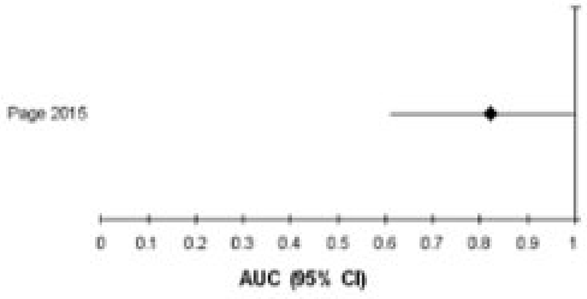

At 12 months (secondary care):

- AUC 0.82 (Range, 0.61-0.1) – High risk of bias

(RoB assessed using the PROBAST checklist)

Question 2: Should validated risk assessment or clinical prediction tools, compared with no tools or with each other, be used to stratify the management of patients with non-specific LBP and/or sciatica based on the outcome of the tool or questionnaire?

Figure S7. Forest plot of comparison: 1 STarT Back risk tool vs. no risk tool stratification, outcome: 1.1 Quality of life(SF-12, Physical Component Score(PCS),0-100) > 4 months (follow-up 1 year).

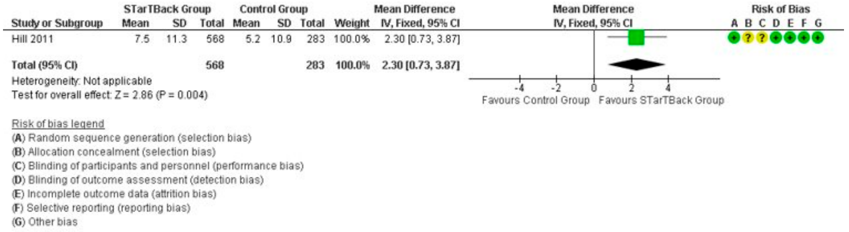

Figure S8. Forest plot of comparison: 1 STarT Back risk tool vs. no risk tool stratification, outcome: 1.2 Quality of life(SF-12, Mental Component Score(MCS),0-100) > 4 m (follow-up 1 year).

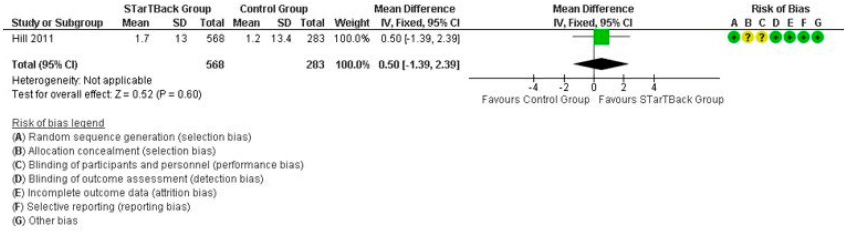

Figure S9. Forest plot of comparison: 1 STarT Back risk tool vs. no risk tool stratification, outcome: 1.3 Pain Severity (VAS,0-10) > 4 m (follow-up 1 year).

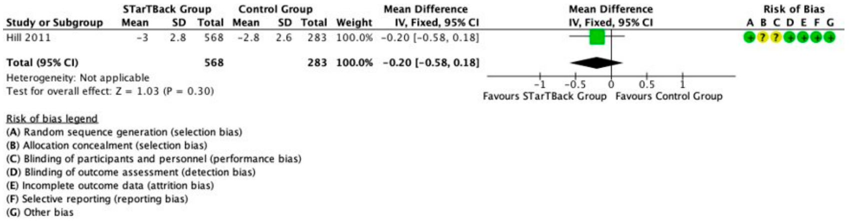

Figure S10. Forest plot of comparison: 1 STarT Back risk tool vs. no risk tool stratification, outcome: 1.4 Function (RMDQ, 0-24) > 4 m (follow-up 1 year).

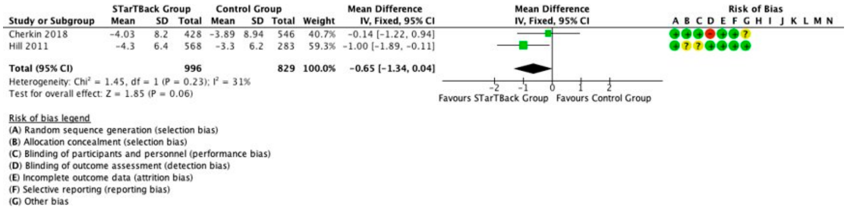

Figure S11. Forest plot of comparison: 1 STarT Back risk tool vs. no risk tool stratification, outcome: 1.5 Psychological Distress (HADS, anxiety subscale, 0-21) > 4 m (follow-up 1 year).

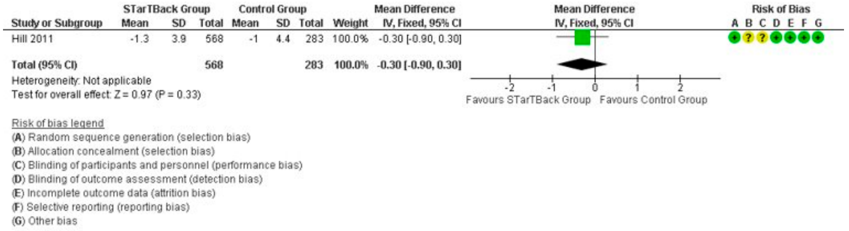

Figure S12. Forest plot of comparison: 1 STarT Back risk tool vs. no risk tool stratification, outcome: 1.6 Psychological Distress (HADS, depression subscale, 0-21) > 4 m (follow-up 1 year).

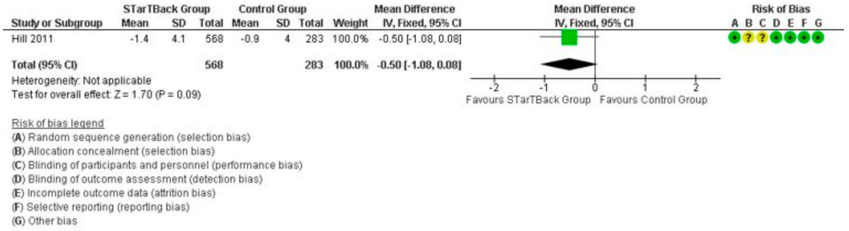

Figure S13. Forest plot of comparison: 1 STarT Back risk tool vs. no risk tool stratification, outcome: 1.7 Healthcare utilization.

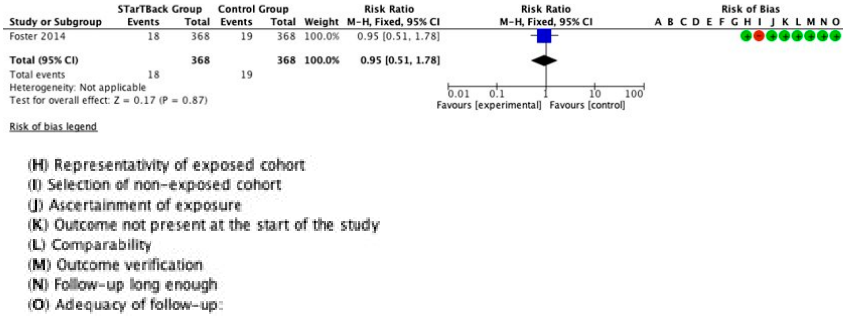

Question 3: Should imaging (plain radiography [X-ray] or magnetic resonance imaging [MRI]) versus no investigation be used to improve functional disability, pain, or psychological distress in patients with LBP and/or sciatica?

Figure S14. Forest plot of comparison: 1 Imaging vs. no imaging for low back pain with/without sciatica, Outcome 1.3: Health-related quality of life (SF-36, 0-100) > 4 months - 1 year (range of scores: 0-100; Better indicated by higher values (RCT).

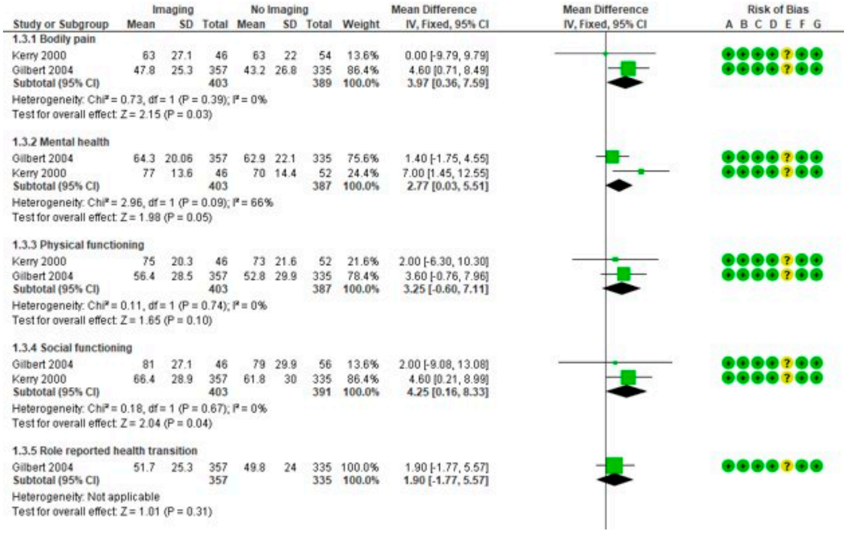

Figure S15. Forest plot of comparison: 1 Imaging vs. no imaging for low back pain with/without sciatica, Outcome 1.6: Pain severity (ALBP score, 0-100) > 4 months - 1 year (follow-up 24 months; range of scores: 0-100; Better indicated by lower values) (RCT)

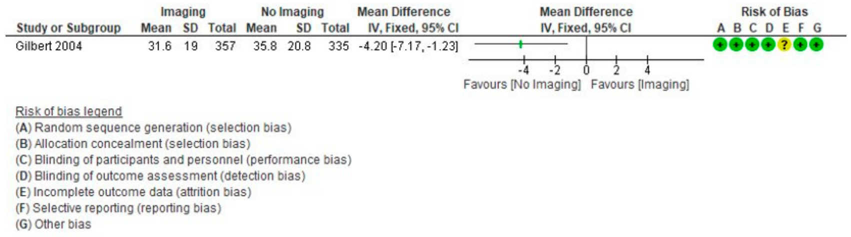

Figure S16. Forest plot of comparison: 1 Imaging vs. no imaging for low back pain with/without sciatica, Outcome 1.8: Function (RMDQ, 0-24) > 4 months - 1 year (follow-up 1 year; range of scores: 0-24; Better indicated by lower values) (RCT).

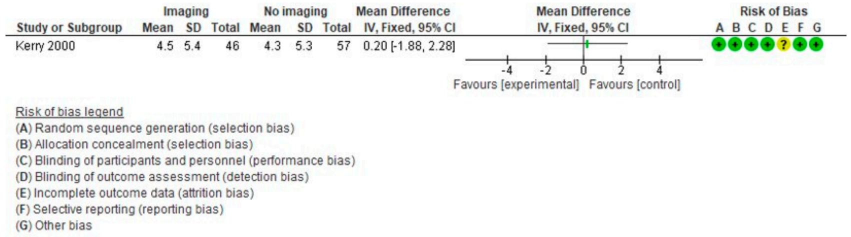

Figure S17. Forest plot of comparison: 1 Imaging vs. no imaging for low back pain with/without sciatica, Outcome 1.10: Psychological distress (HADS Anxiety Score, 0-21) > 4 months - 1 year (follow-up 1 year; range of scores: 0-21; Better indicated by lower values) (RCT).

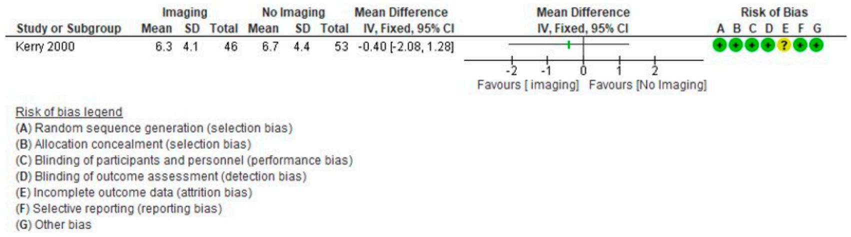

Figure S18. Forest plot of comparison: 1 Imaging vs. no imaging for low back pain with/without sciatica, Outcome 1.12: Psychological distress (HADS Depression Score, 0-21) > 4 months - 1 year (follow-up 1 year; range of scores: 0-21; Better indicated by lower values) (RCT).

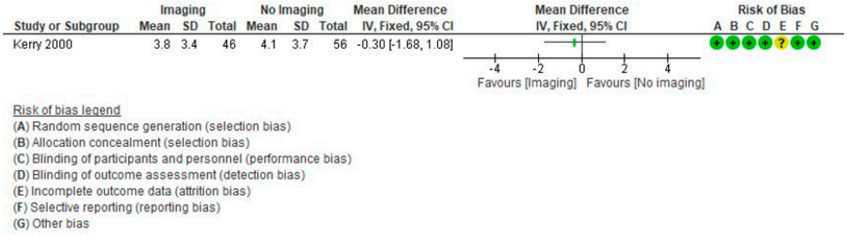

Figure S19. Forest plot of comparison: 1 Imaging vs. no imaging for low back pain with/without sciatica, Outcome 1.13: Healthcare utilization > 4 months to 1 year.

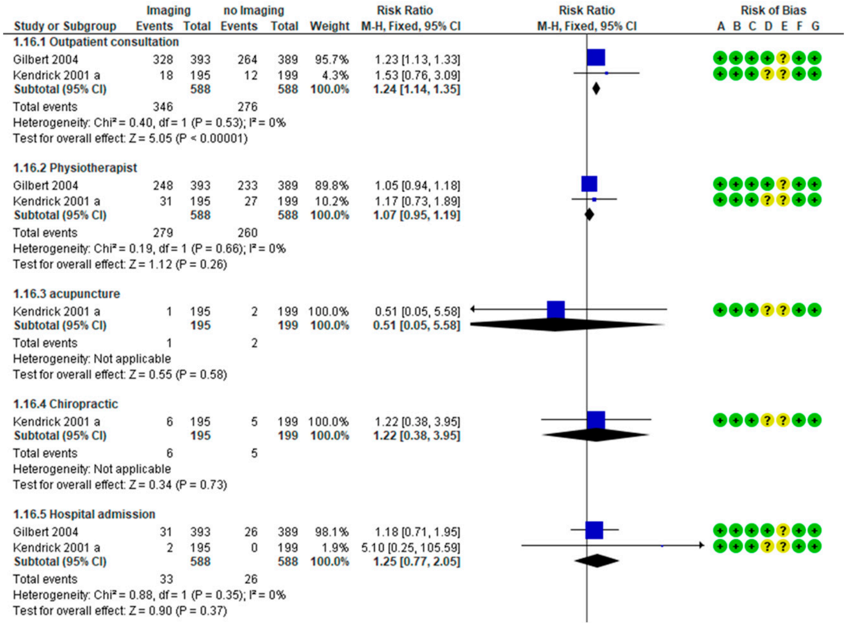

Figure S20. Forest plot of comparison: 1 Imaging vs. no imaging for low back pain with/without sciatica, Outcome 1.13: Healthcare utilization > 4 months to 1 year.

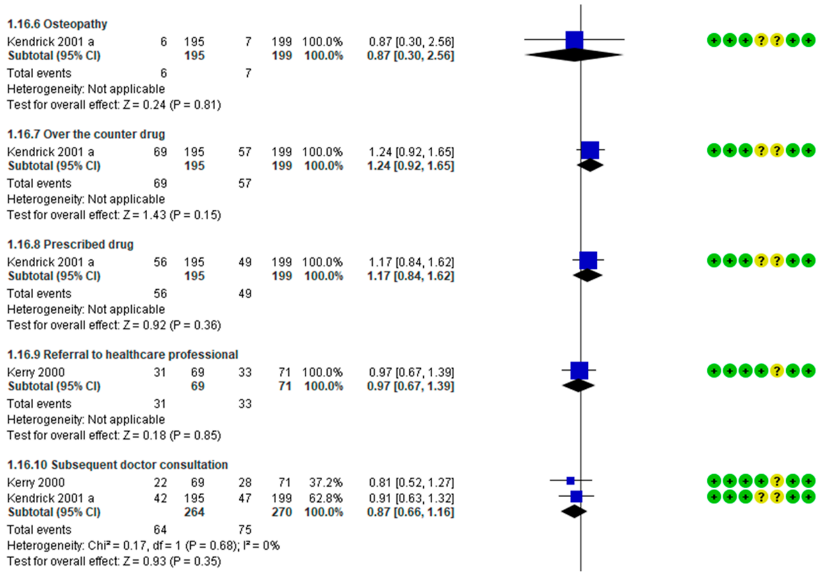

Figure S21. Forest plot of comparison: 1 Imaging vs. no imaging for low back pain with/without sciatica, Outcome 1.13: Healthcare utilization > 4 months to 1 year.

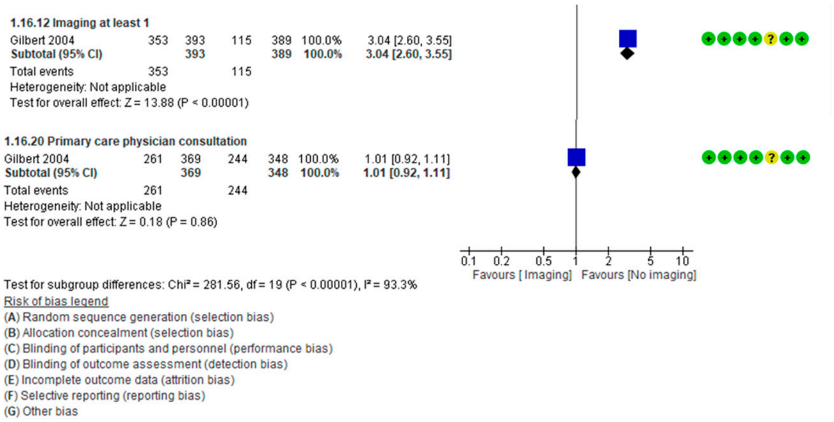

Imaging vs. no imaging for low back pain with/without sciatica, Outcome 1.26: Responder criteria (>30% improvement in pain and function) - not reported

Imaging vs. no imaging for low back pain with/without sciatica, Outcome 1.27: Adverse events: Morbidity - not reported

Question 4: Should pharmacological treatment versus placebo or usual care/waiting list, or one or more pharmacological interventions compared with each other, be used in patients with sciatica?

Figure S22. Forest plot of comparison: 1 NSAIDs vs. placebo, outcome: 1.1 Pain Severity (VAS, change score) ≤ 4 months.

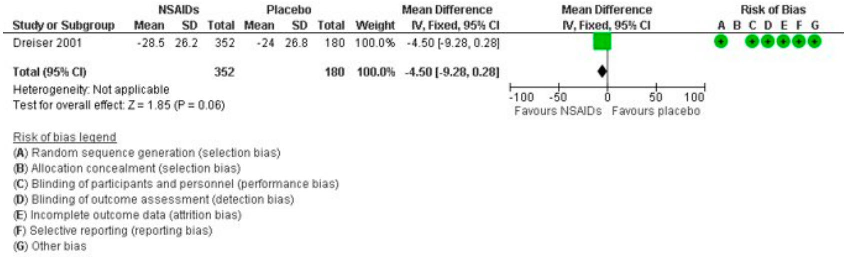

Figure S23. Forest plot of comparison: 1 NSAIDs vs. placebo, outcome: 1.5 Adverse events: Morbidity ≤ 4 months.

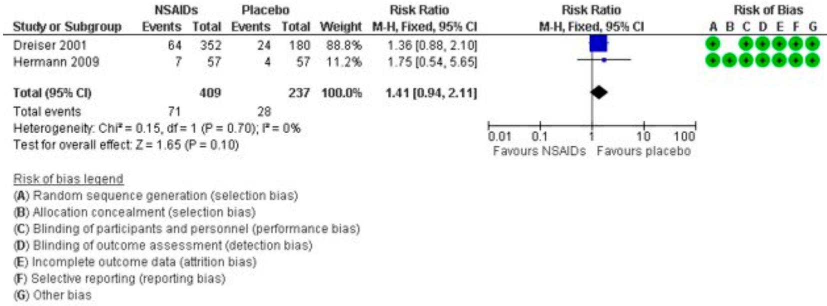

Figure S24. Forest plot of comparison: 2 Benzodiazepines vs. placebo, outcome: 2.2 Responder criteria (VAS pain reduction of 50%) ≤ 4 months.

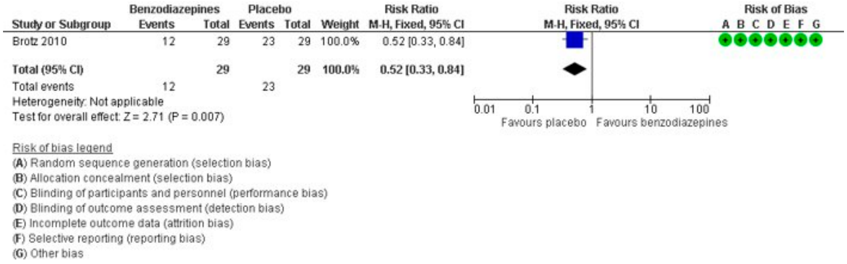

Figure S25. Forest plot of comparison: 3 Gabapentinoids vs. placebo, outcome: 3.1 Pain severity (VAS, NRS, 0-10, change score) ≤ 4 months.

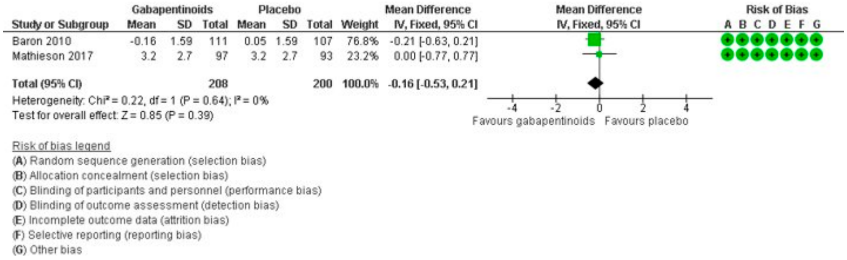

Figure S26. Forest plot of comparison: 3 Gabapentinoids vs. placebo, outcome: 3.2 Pain severity (pain at rest, 0-3, high is poor, final value) ≤ 4 months.

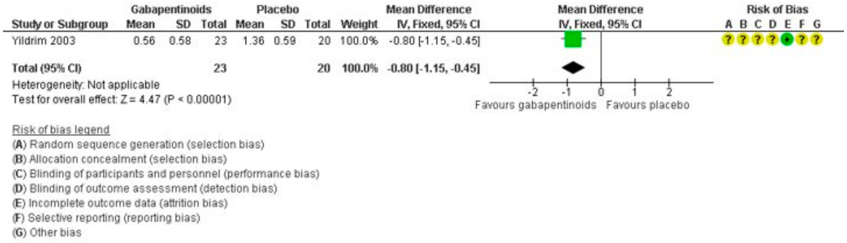

Figure S27. Forest plot of comparison: 3 Gabapentinoids vs. placebo, outcome: 3.3 Pain severity (NRS, 0-10, high is poor, final value) > 4 months.

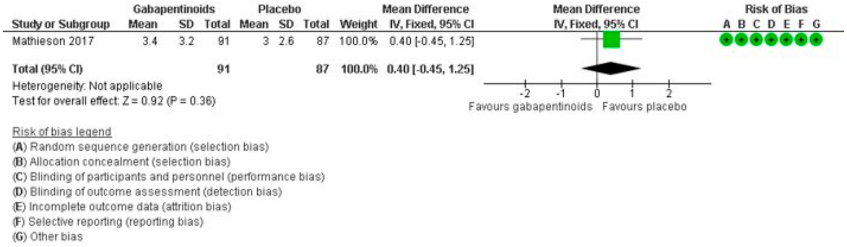

Figure S28. Forest plot of comparison: 3 Gabapentinoids vs. placebo, outcome: 3.4 Function (Roland Disability Questionnaire, 0-23, high is poor, final value) ≤ 4 months.

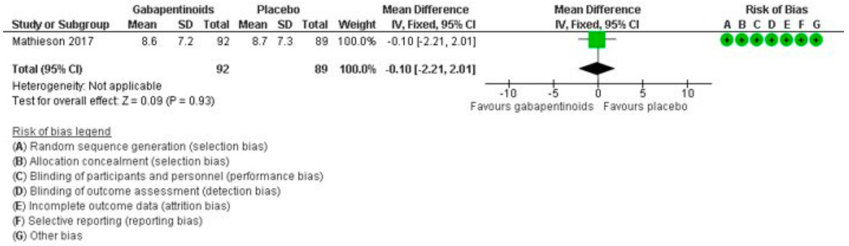

Figure S29. Forest plot of comparison: 3 Gabapentinoids vs. placebo, outcome: 3.5 Function (Roland Disability Questionnaire, 0-23, high is poor, final value) at > 4 months.

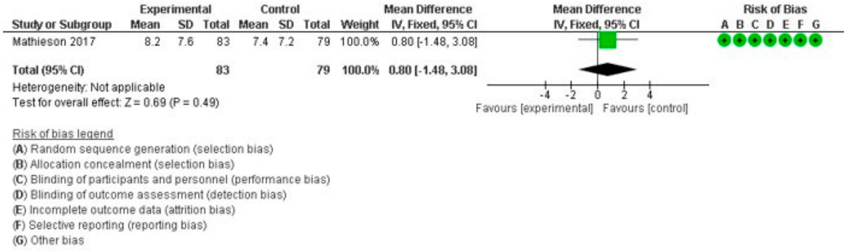

Figure S30. Forest plot of comparison: 3 Gabapentinoids vs. placebo, outcome: 3.6 Adverse events (morbidity) ≤ 4 months.

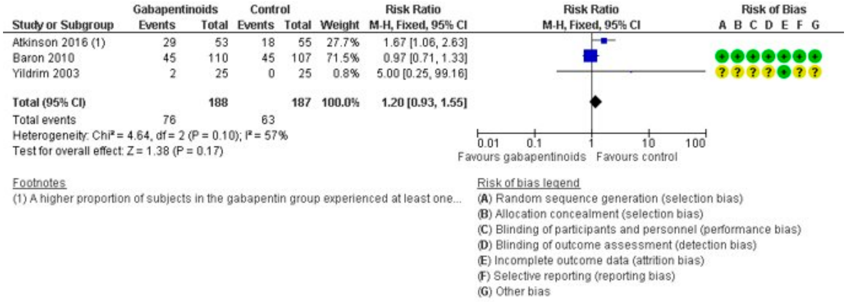

Figure S31. Forest plot of comparison: 3 Gabapentinoids vs. placebo, outcome: 3.7 Adverse events (morbidity) at > 4 months.

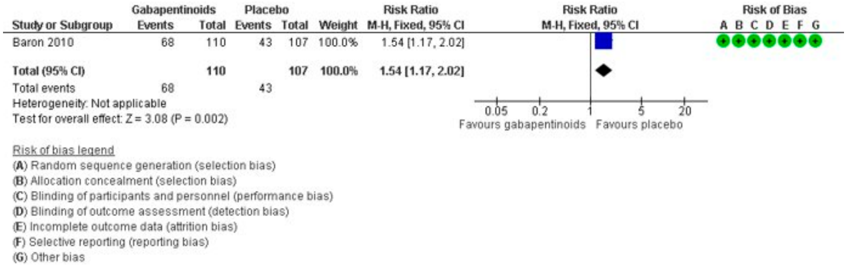

Figure S32. Forest plot of comparison: 4 Corticosteroids vs. placebo, outcome: 4.1 Pain severity (NRS, 0-10, high is poor, change score) ≤ 4 months.

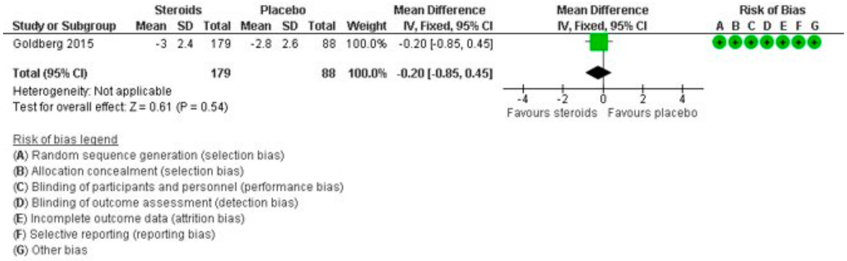

Figure S33. Forest plot of comparison: 4 Corticosteroids vs. placebo, outcome: 4.2 Pain severity (NRS, 0-10, high is poor, change score) at > 4 months.

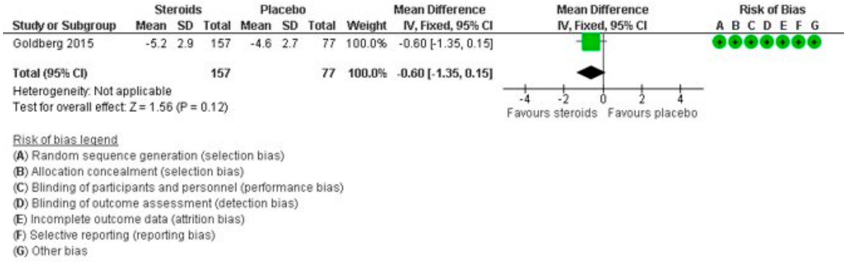

Figure S34. Forest plot of comparison: 4 Corticosteroids vs. placebo, outcome: 4.3 Function (ODI, 0-100, high is poor, change score) ≤ 4 months.

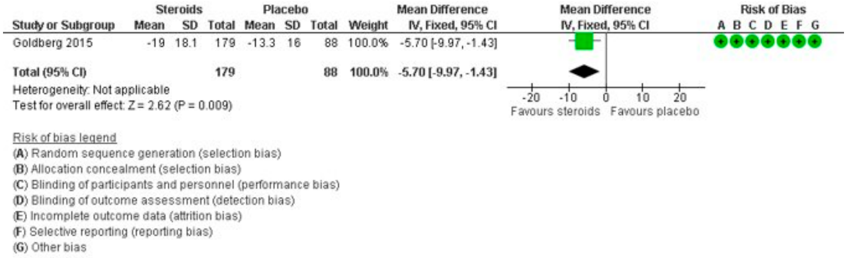

Figure S35. Forest plot of comparison: 4 Corticosteroids vs. placebo, outcome: 4.4 Function (ODI, 0-100, high is poor, change score) at > 4 months.

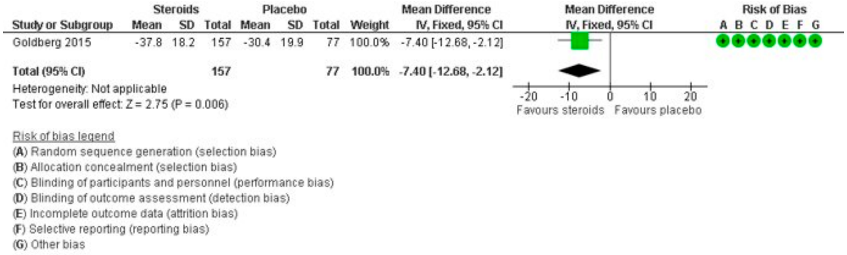

Figure S36. Forest plot of comparison: 4 Corticosteroids vs. placebo, outcome: 4.5 Adverse events (morbidity) ≤ 4 months.

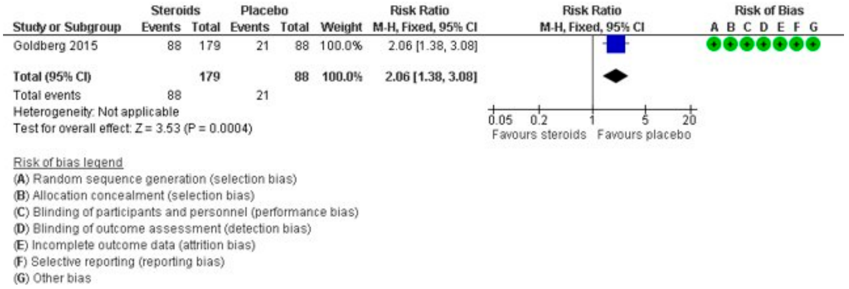

Question 5: Should interventions or multidisciplinary programmes with a specified return to work focus (or including ergonomic interventions) versus placebo, usual care or waiting list, or versus one or more interventions compared with each other or other non-invasive interventions (either alone or in combination) be used in patients with non-specific LBP and/or sciatica?

Figure S37. Forest plot of comparison: 1. Multidisciplinary programs vs. placebo or usual care, outcome 1: Function (RMDQ 0-24, change score).

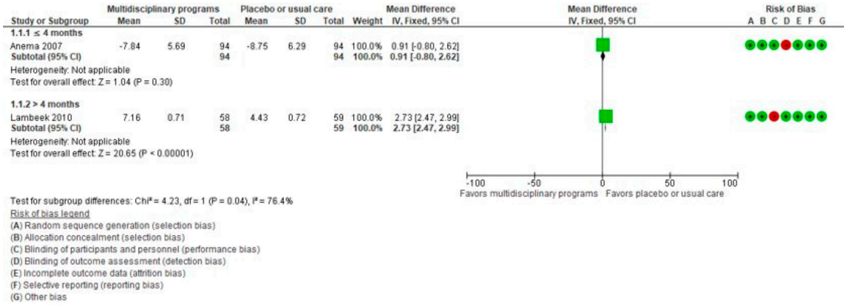

Figure S38. Forest plot of comparison: 1. Multidisciplinary programs vs. placebo or usual care, outcome 2: Quality of life (EQ-5D 0-1, change score) ≤ 4 months.

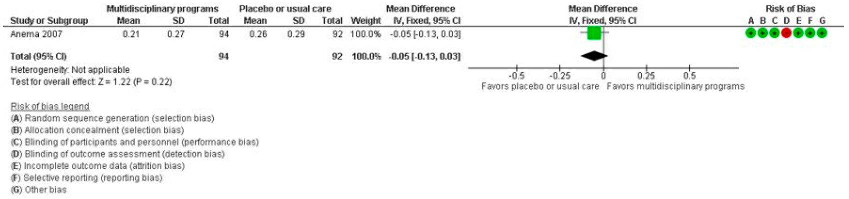

Figure S39. Forest plot of comparison: 1. Multidisciplinary programs vs. placebo or usual care, outcome 3: Pain (NRS 0-10, change score).

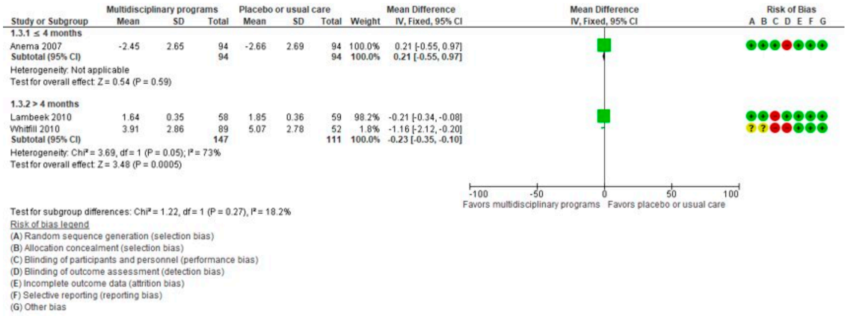

Figure S40. Forest plot of comparison: 1. Multidisciplinary programs vs. placebo or usual care, outcome 4: Days to return to work (final value) ≤ 4 months.

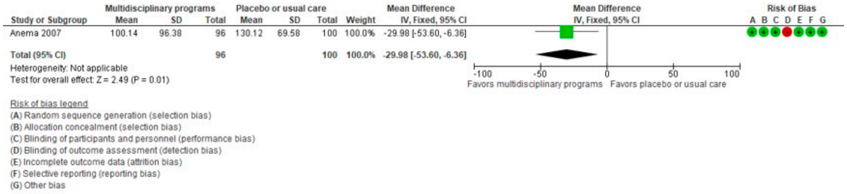

Figure S41. Forest plot of comparison: 1. Multidisciplinary programs vs. placebo or usual care, outcome 5: Psychological distress (BDI, 0-63) > 4 months.

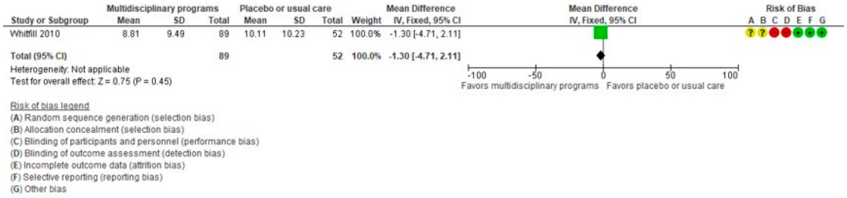

Forest plot of comparison: 1. Multidisciplinary programs vs. placebo or usual care, outcome 6: Responder criteria (>30% improvement in pain and function) - not reported.

Question 6: Should psychological interventions (behavioural therapies, cognitive therapies, cognitive–behavioural approaches, mindfulness, and acceptance and commitment therapy) versus placebo or usual care/waiting list, or versus one or more interventions compared with each other or other non-invasive interventions (either alone or in combination), be used in patients with non-specific LBP and/or sciatica?

Figure S42. Forest plot of comparison: 1. Cognitive behavioral approaches vs. placebo, outcome: 1.2 Pain severity (pain and impairment relationship scale) ≥ 4 months.

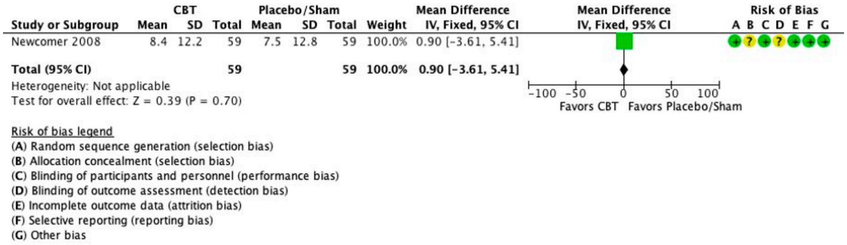

Figure S43. Forest plot of comparison: 1. Cognitive behavioral approaches vs. placebo, outcome: 1.3 Function (ODI, 0-100) ≥ 4 months.

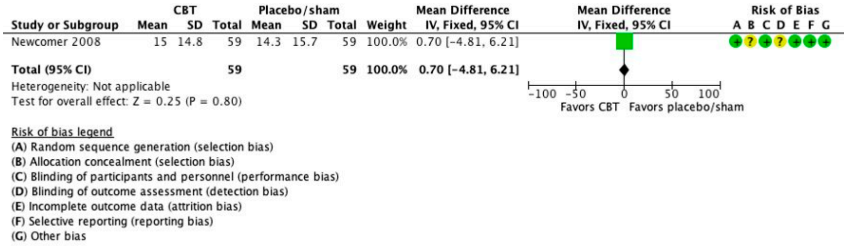

Figure S44. Forest plot of comparison: 2. Cognitive behavioral approaches vs. usual care/waiting list, outcome: 2.1 Pain severity (VAS 0-10, final values).

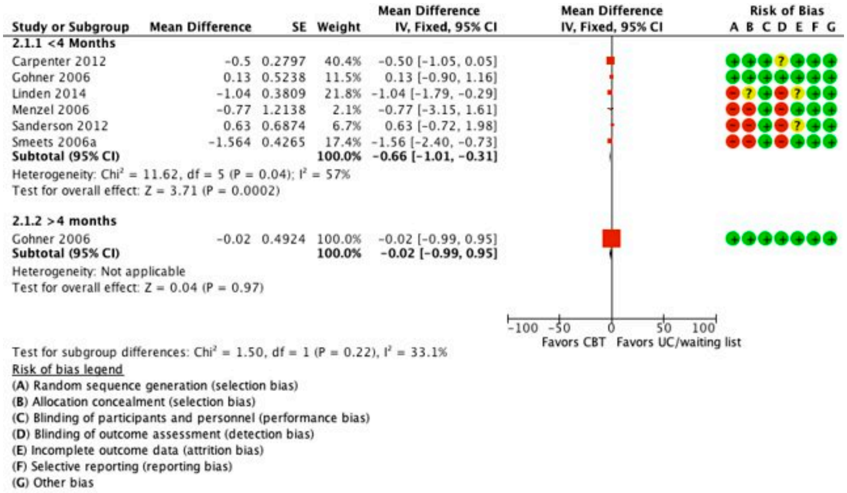

Figure S45. Forest plot of comparison: 2. Cognitive behavioral approaches vs. usual care/waiting list, outcome: 2.2 Function (RMDQ, 0-24 or 0-23).

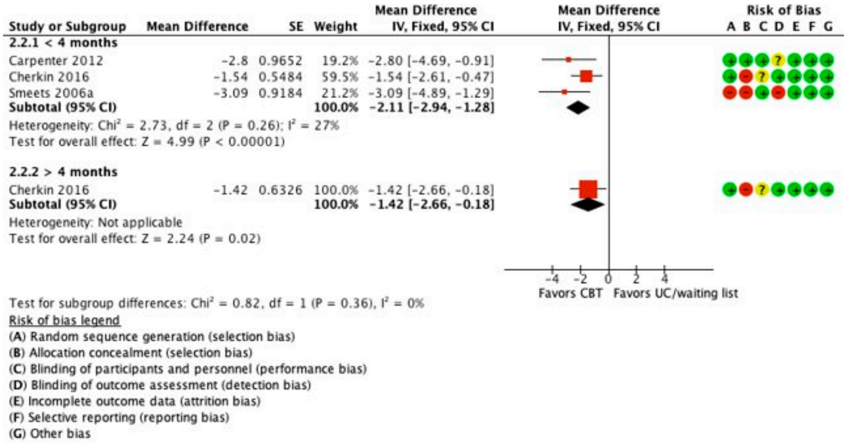

Figure S46. Forest plot of comparison: 2. Cognitive behavioral approaches vs. usual care/waiting list, outcome: 2.3 Function (PDI, 0-70).

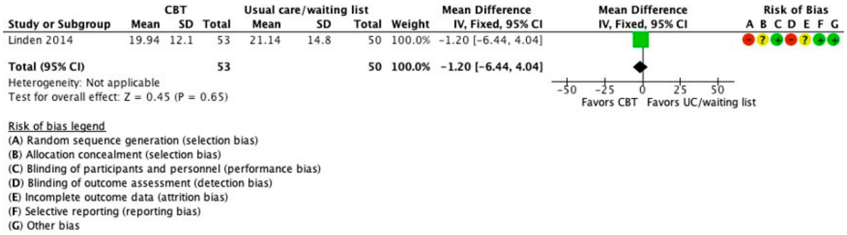

Figure S47. Forest plot of comparison: 2. Cognitive behavioral approaches vs. usual care/waiting list, outcome: 2.4 Quality of life (SF-36 perceived general health, first question of general perception subscale, 0-5).

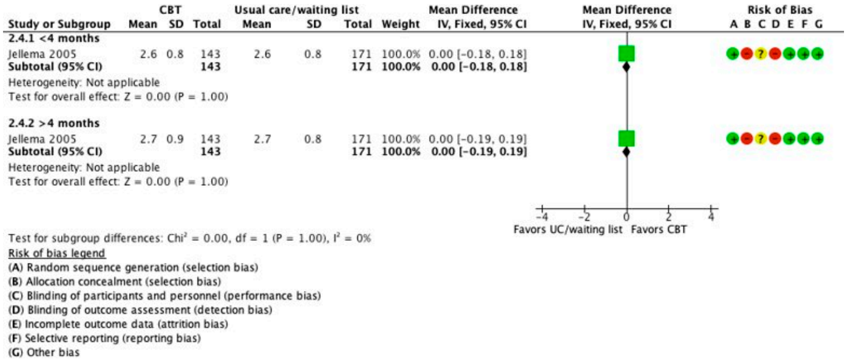

Figure S48. Forest plot of comparison: 2. Cognitive behavioral approaches vs. usual care/waiting list, outcome: 2.5 Psychological distress (BDI 0-63) < 4 months.

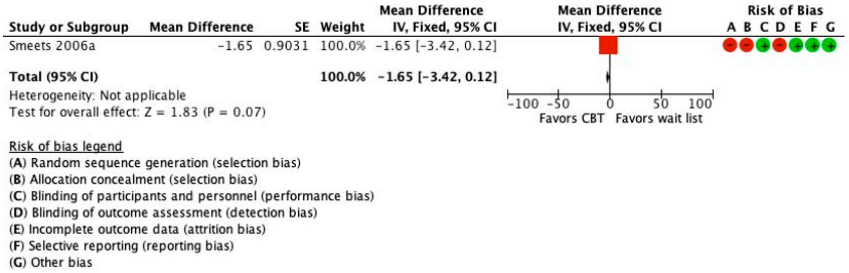

Figure S49. Forest plot of comparison: 3. Mindfulness vs. usual care/waiting list, outcome: 3.1 Pain severity (McGill pain 0-78).

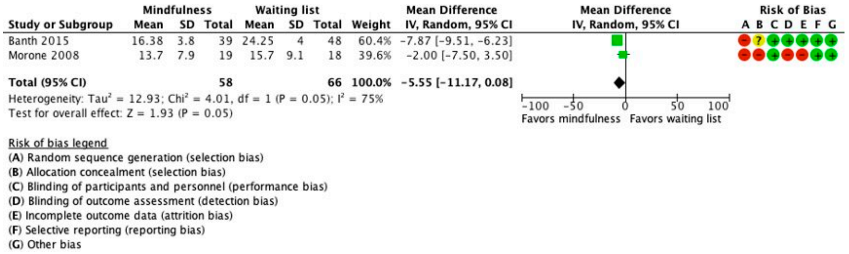

Figure S50. Forest plot of comparison: 3. Mindfulness vs. usual care/waiting list, outcome: 3.2 Pain severity (NRS 1-100).

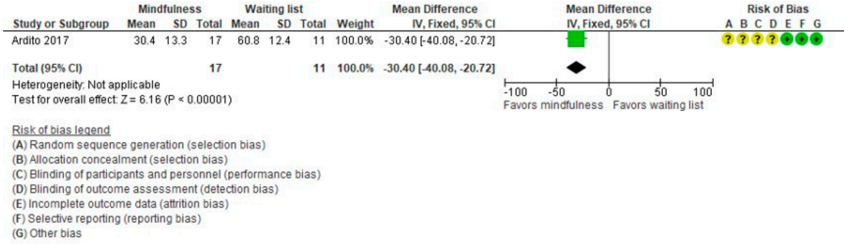

Figure S51. Forest plot of comparison: 3. Mindfulness vs. usual care/waiting list, outcome: 3.3 Function (RMDQ 0-24).

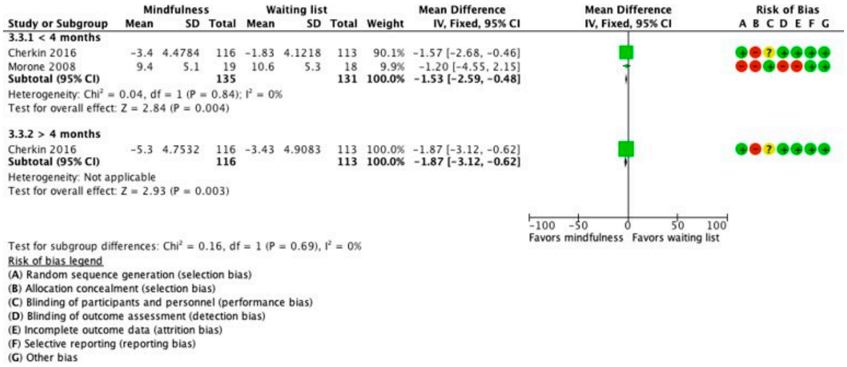

Figure S52. Forest plot of comparison: 3. Mindfulness vs. usual care/waiting list, outcome: 3.4 Quality of life (SF-36, 0-100) ≤ 4 months.

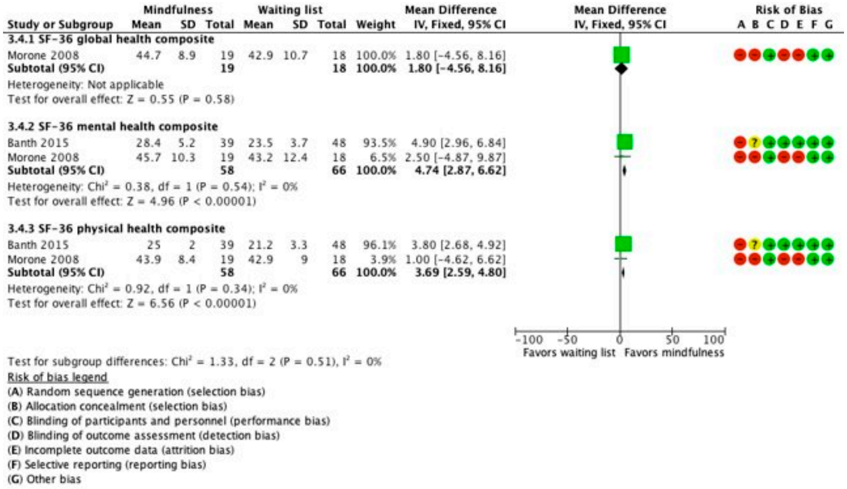

Figure S53. Forest plot of comparison: 3. Mindfulness vs. usual care/waiting list, outcome: 3.5 Quality of life (SF-36, 0-100) > 4 months.

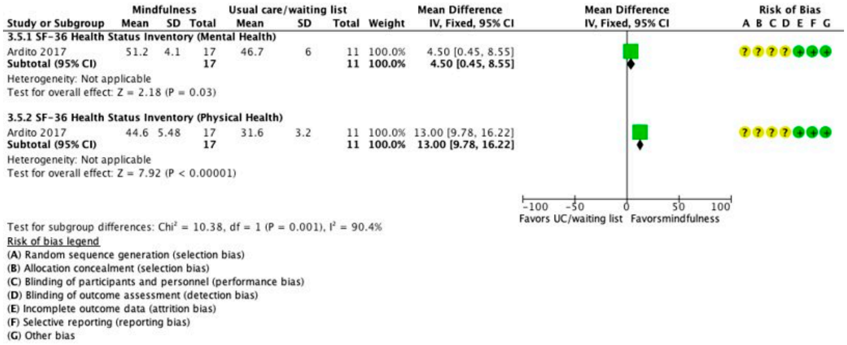

Figure S54. Forest plot of comparison: 3. Mindfulness vs. usual care/waiting list, outcome: 3.6 Depression (Beck Depression Inventory [BDI-II]).

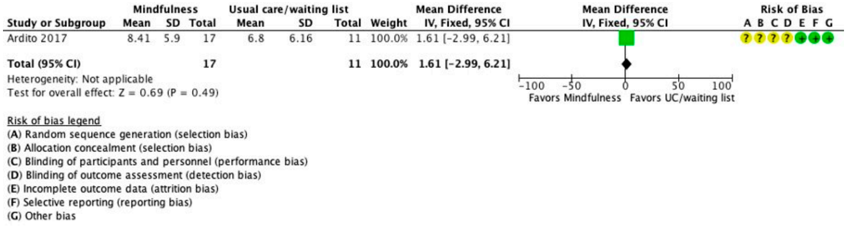

Figure S55. Forest plot of comparison: 4. Cognitive therapy vs. usual care/waiting list, outcome: 4.2 Quality of life (SF-36) > 4 months.

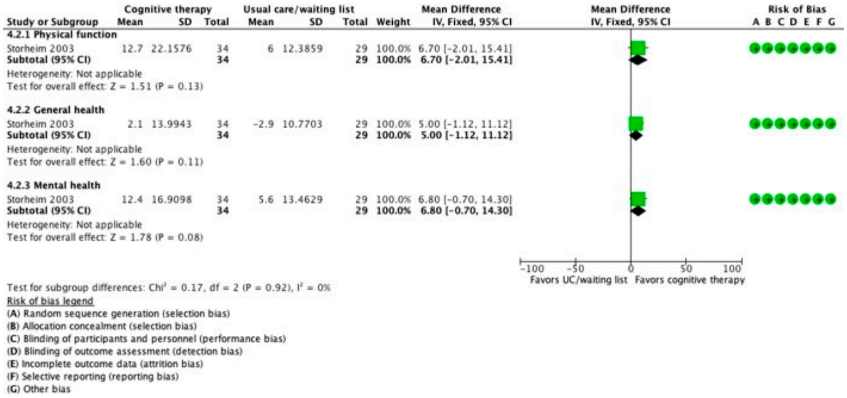

Forest plot of comparison: 1. Cognitive behavioral approaches vs. placebo, outcome: 1.1 Short-term results (≤ 4 months) - not reported.

Forest plot of comparison: 4. Cognitive therapy vs. usual care/waiting list, outcome: 4.1 Short-term results (≤ 4 months) - not reported.

Question 7. Should epidural injections versus non-invasive treatments be used in patients with sciatica?

Figure S56. Forest plot of comparison: 1 Image-guided: Steroid + anesthetic epidural vs. combination of non-invasive interventions caused by (≥ 70%) disc prolapse, outcome: 1.1 Pain (VAS- scale 1-10).

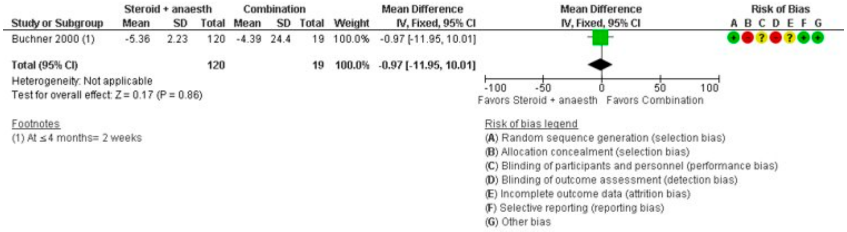

Figure S57. Forest plot of comparison: 2. Non-image-guided: Steroid + anesthetic epidural vs. pharmacological treatment (NSAIDs) caused by (≥ 70%) disc prolapse, outcome: 2.1 Pain (VAS) ≤ 4 months (scale 1-10).

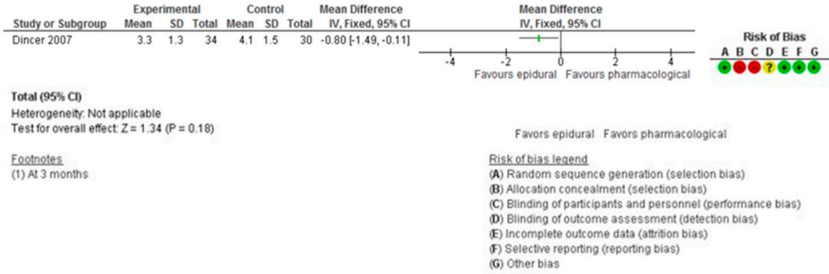

Figure S58. Forest plot of comparison: 2. Non-image-guided: Steroid + anesthetic epidural versus Pharmacological treatment (NSAIDs) caused by (≥70%) disc prolapse, outcome: 2.2 Disability (Oswetry disability questionnaire) ≤ 4 months (scale 1-100).

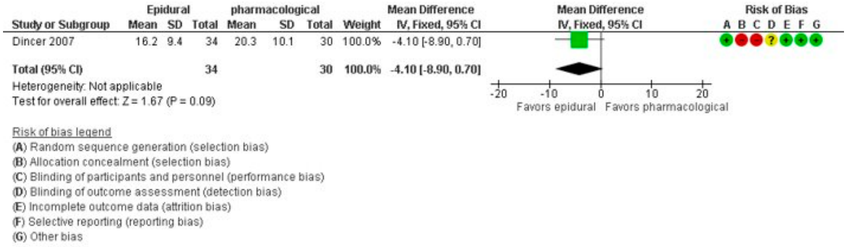

Figure S59. Forest plot of comparison: 2. Non-image-guided: Steroid + anesthetic epidural versus Pharmacological treatment (NSAIDs) caused by (≥70%) disc prolapse, outcome: 2.3 Healthcare utilisation (no. using paracetamol at follow-up) ≤4 months.

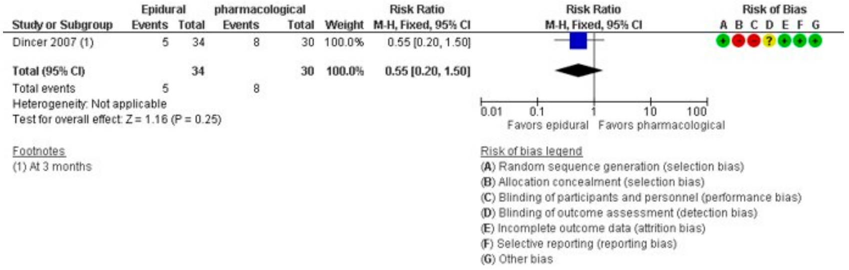

Figure S60. Forest plot of comparison: 3. Non-image-guided: Steroid + anesthetic epidural versus Pharmacological treatment (Combination NSAIDS+ Opioids+Muscle relaxants) in sciatica caused by (≥70%) disc prolapse, outcome: 3.1 Pain (VAS - scale 1-10).

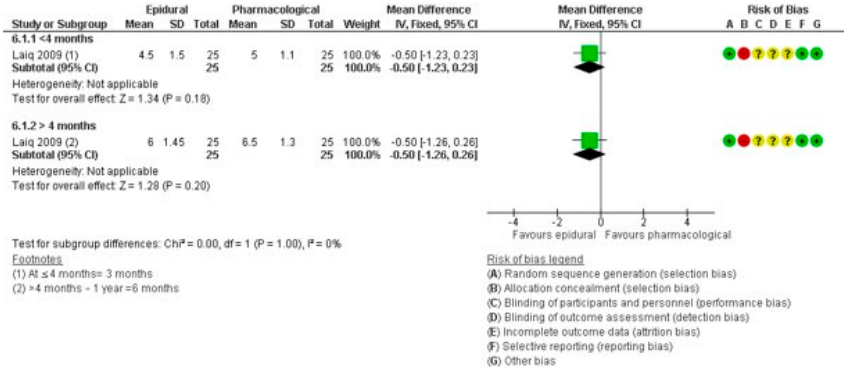

Figure S61. Forest plot of comparison: 3. Non-image-guided: Steroid + anesthetic epidural versus Pharmacological treatment (Combination NSAIDS+ Opioids+Muscle relaxants) in sciatica caused by (≥70%) disc prolapse, outcome: 3.2 Adverse events – morbidity (minor adverse events defined as flushing and headache, or back ache).

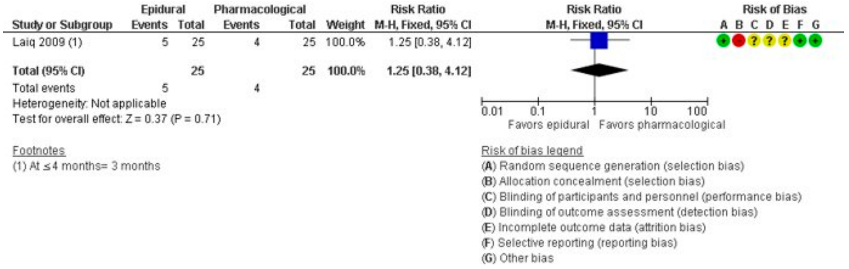

Question 8. Should image-concordant pathology or the presence of radicular symptoms versus no image-concordant pathology or the presence of radicular symptoms be used for predicting response to surgery in patients with suspected sciatica?

Figure S62. Forest plot of comparison: 1 Image-concordant vs. no image-concordant pathology or presence of radicular symptoms, outcome: 1. Leg pain greater than back pain on 50% improvement in pain assessed by VAS in 1 year (Adjusted OR, sciatica population - surgery: discectomy).

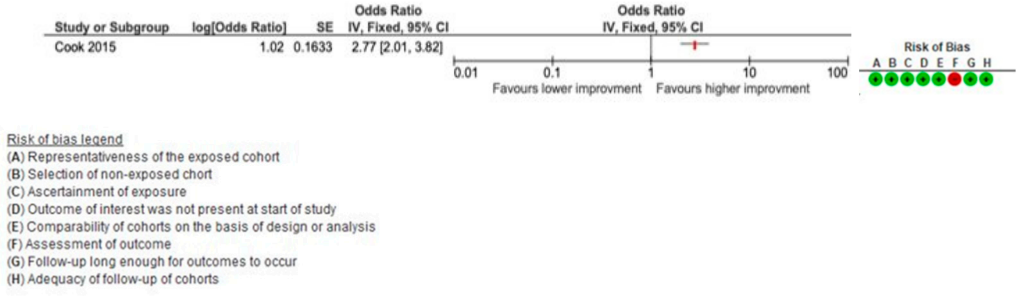

Figure S63. Forest plot of comparison: 1 Image-concordant vs. no image-concordant pathology or presence of radicular symptoms, outcome: 3. Pre-operative leg pain (VAS > 43) vs. pre-operative leg pain (VAS ≤ 43) on leg pain (VAS ≤ 10 mm) at 12 months (Adjusted ORs) [adults aged 18-65 with back or leg pain].

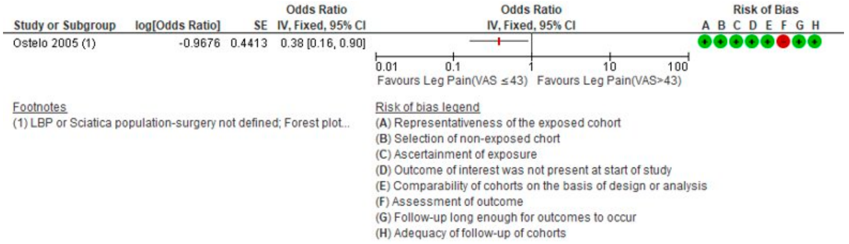

Figure S64. Forest plot of comparison: 1 Image-concordant vs. no image-concordant pathology or presence of radicular symptoms, outcome: 4. Effects for leg pain greater than back pain on 50% improvement in function assessed by ODI in 1 year (Adjusted ORs) [adults with sciatica].

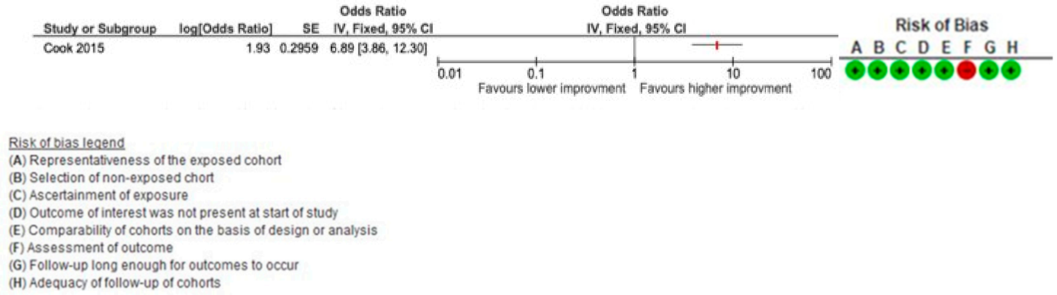

Figure S65. Forest plot of comparison: 1 Image-concordant vs. no image-concordant pathology or presence of radicular symptoms, Outcome 7: Leg pain greater than back pain predicting function (ODI) at 4 years (Adjusted mean difference, low back pain and/or sciatica population) – surgery: open decompressive laminectomy.

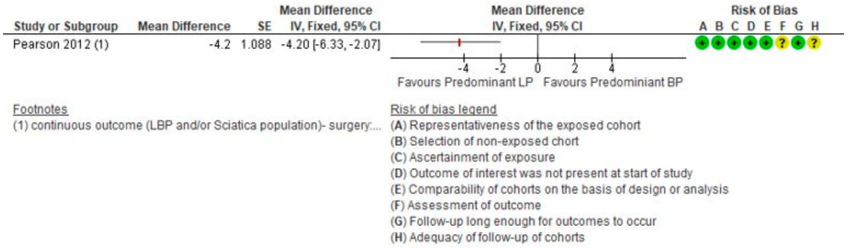

Outcome 6: Surgery conversion rate – not reported.

Outcome 8: Adverse events: Morbidity – not reported.

Outcome 9: Adverse events: Re-operation rate – not reported.

Outcome 10: Adverse events: Mortality – not reported.

Outcome 11: Quality of life – not reported.

Question 9: Should spinal decompression versus usual care or other interventions be used in patients with sciatica?

Figure S66. Forest plot of comparison: 1 Discectomy vs. usual care, Outcome: 1.1 Quality of life, (SF-36 0-100) ≤ 4 months.

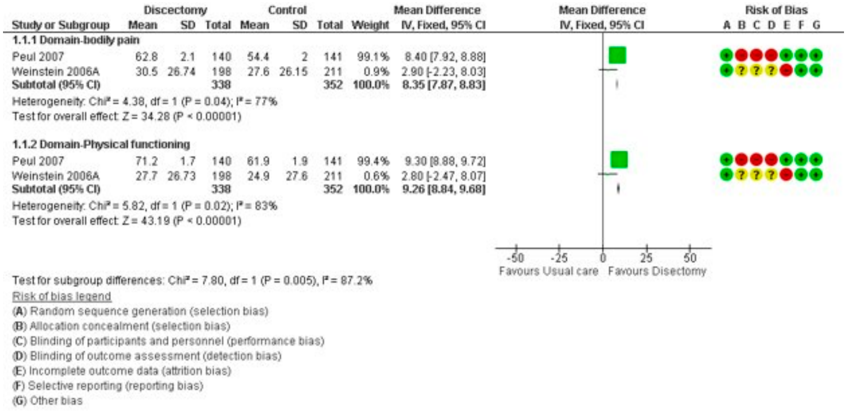

Figure S67. Forest plot of comparison: 1 Discectomy vs. usual care, Outcome: 1.2 Quality of life (SF-36, 0-100) at 2 years.

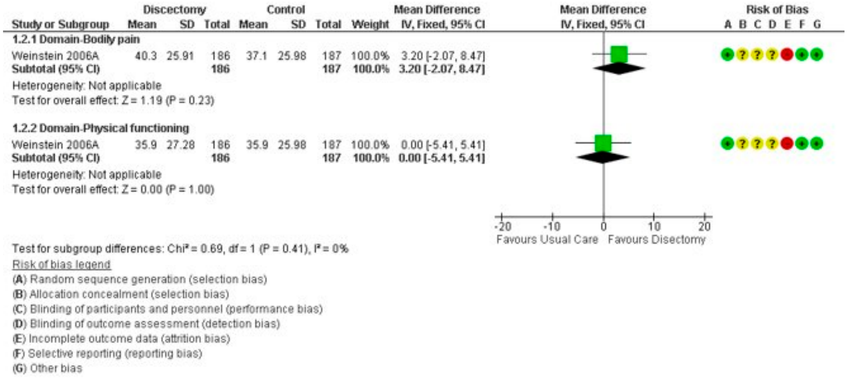

Figure S68. Forest plot of comparison: 1 Discectomy vs. usual care, Outcome: 1.4 Leg pain severity (VAS, 0-10) > 4 months to 1 year.

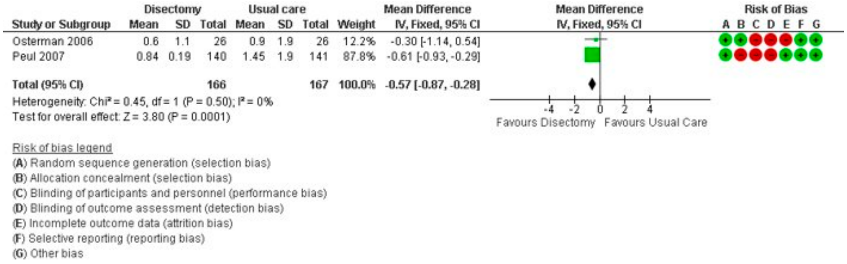

Figure S69. Forest plot of comparison: 1 Discectomy vs. usual care, Outcome: 1.5 Back pain severity (VAS, 0-10) > 4 months to 1 year.

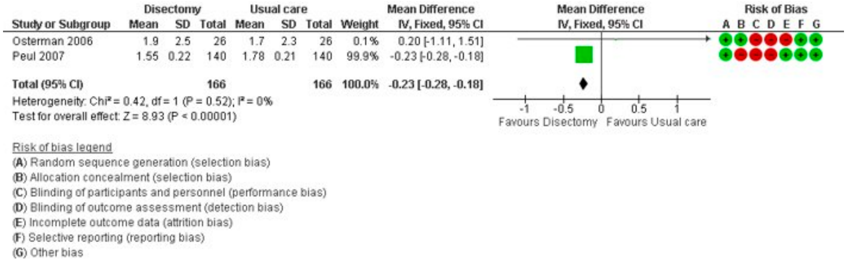

Figure S70. Forest plot of comparison: 1 Discectomy vs. usual care, Outcome: 1.6 Pain severity (Sciatica Bothersomeness Index, 0-24) > 4 months to 1 year.

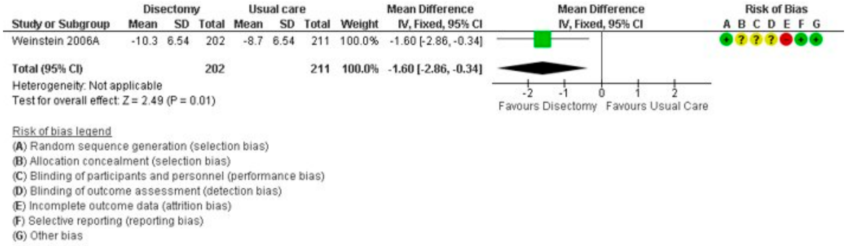

Figure S71. Forest plot of comparison: 1 Discectomy vs. usual care, Outcome: 1.8 Function (ODI, change score) > 4 months to 1 year).

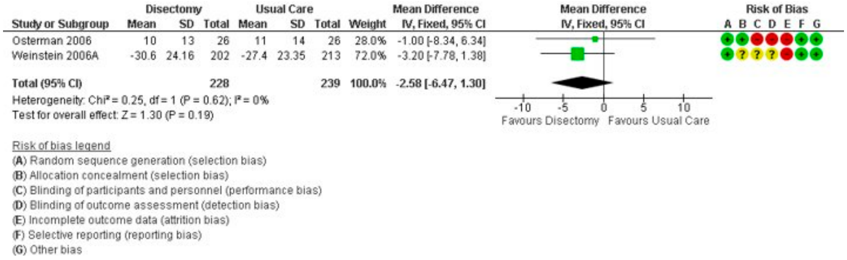

Figure S72. Forest plot of comparison: 1 Discectomy vs. usual care, Outcome: 1.9 Adverse events: Morbidity.

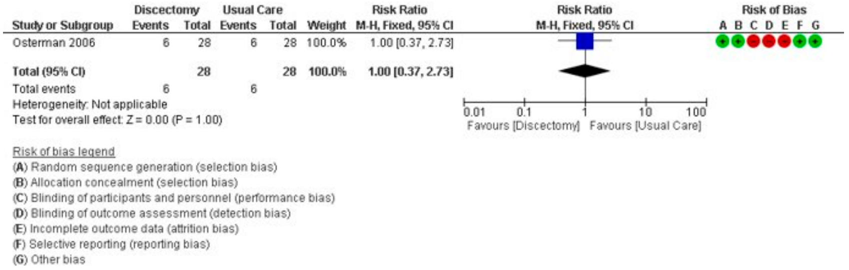

Figure S73. Forest plot of comparison: 1 Discectomy vs. usual care, Outcome: 1.13 Healthcare utilization (number of patients with additional physical therapy visits) > 4 months to 2 years.

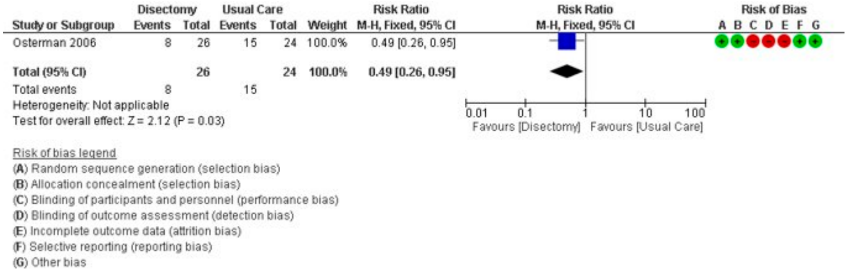

Figure S74. Forest plot of comparison: 1 Discectomy versus usual care, Outcome: 1.14 Adverse events: Mortality.

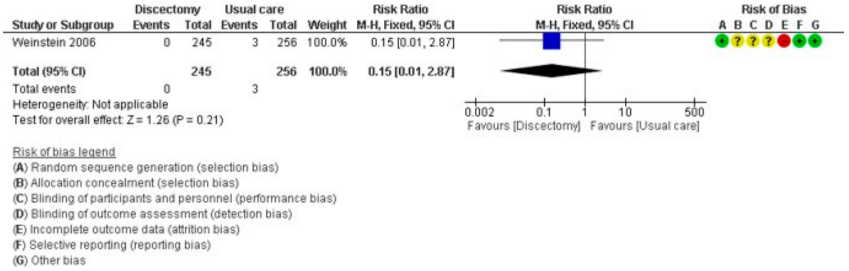

Figure S75. Forest plot of comparison: 2 Discectomy vs. combination treatment (manual therapy + biomechanical exercise + self-management), Outcome: 2.1 Quality of life (SF-36, 0-100) ≤ 4 months.

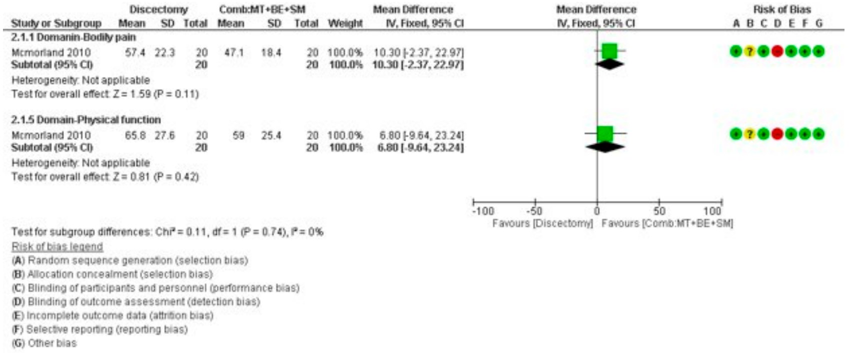

Figure S76. Forest plot of comparison: 2 Discectomy vs. combination treatment (manual therapy + biomechanical exercise + self-management), Outcome: 2.2 Pain severity (McGill, 0-78) ≤ 4 months.

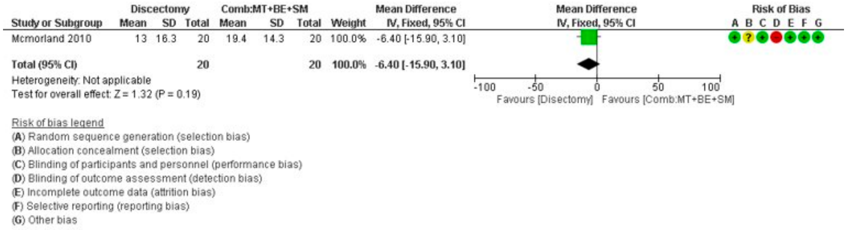

Figure S77. Forest plot of comparison: 2 Discectomy vs. combination treatment (manual therapy + biomechanical exercise + self-management), Outcome: 2.3 Function (Roland Morris) at 12 weeks.

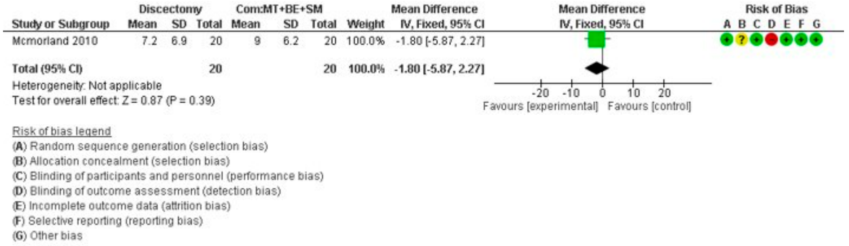

Figure S78. Forest plot of comparison: 3 Percutaneous decompression vs. usual care, Outcome: 3.2 Pain severity (leg pain NVS, 0-10) 4 months to 2 years.

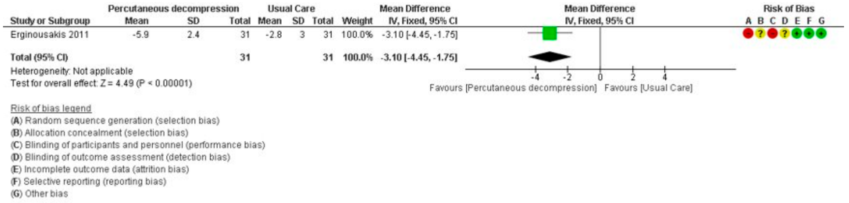

Figure S79. Forest plot of comparison: 4 Plasma disc decompression vs. other treatment (epidural steroid), Outcome: 4.2 Pain severity (leg pain VAS, 0-10) > 4 months to 6 months.

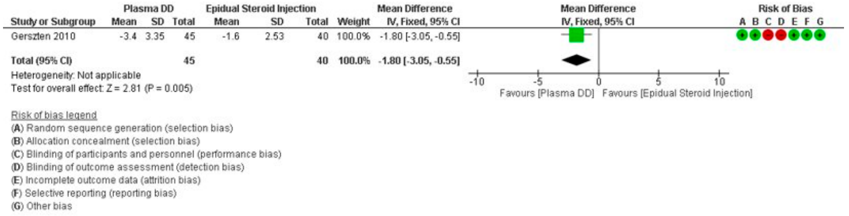

Figure S80. Forest plot of comparison: 4 Plasma disc decompression vs. other treatment (epidural steroid), Outcome: 4.3 Pain severity (back pain; VAS, 0-10) at 6 months.

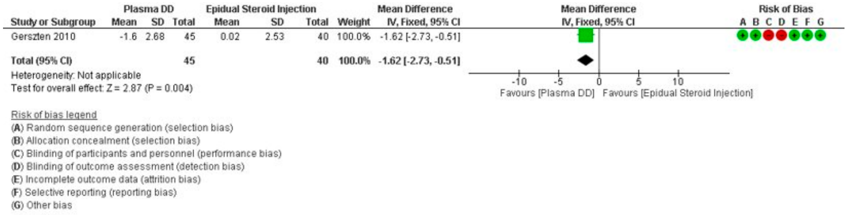

Figure S81. Forest plot of comparison: 4 Plasma disc decompression vs. other treatment (epidural steroid), Outcome: 4.4 Function (ODI, 0-100) at 6 months.

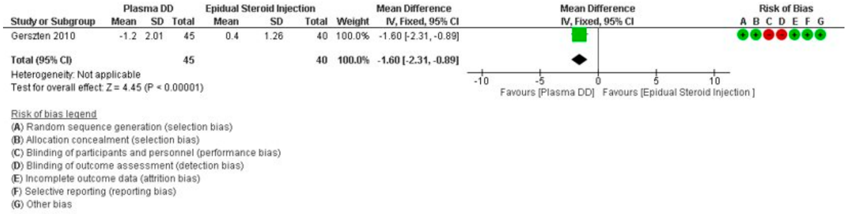

Figure S82. Forest plot of comparison: 4 Plasma disc decompression vs. other treatment (epidural steroid), Outcome: 4.5 Adverse events (procedure-related) at 6 months.

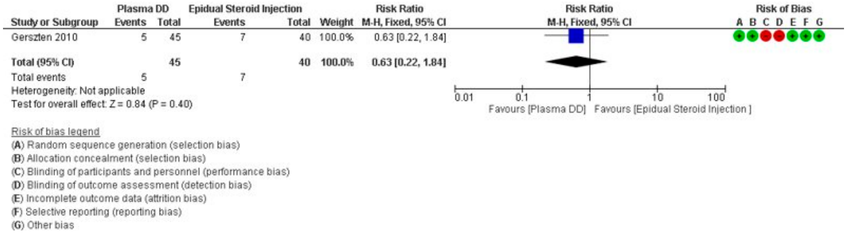

Figure S83. Forest plot of comparison: 4 Plasma disc decompression vs. other treatment (epidural steroid), Outcome: 4.10 Adverse events: Mortality.

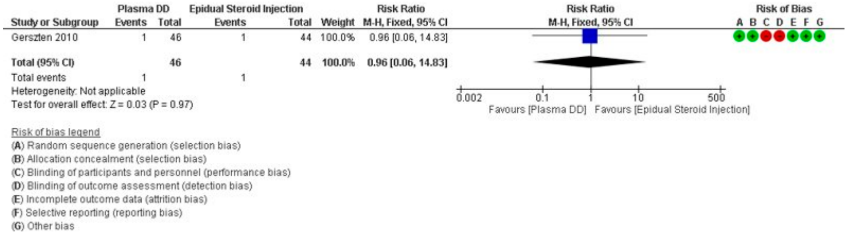

Figure S84. Forest plot of comparison: 4 Plasma disc decompression vs. other treatment (epidural steroid), Outcome: 4.6 Adverse events: Morbidity.

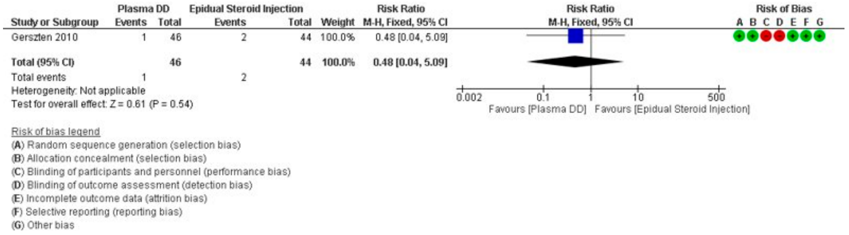

Figure S85. Forest plot of comparison: 5 Laminectomy vs. usual care, Outcome: 5.1 Quality of life (SF-36, 0-100) > 4 months to 1 year.

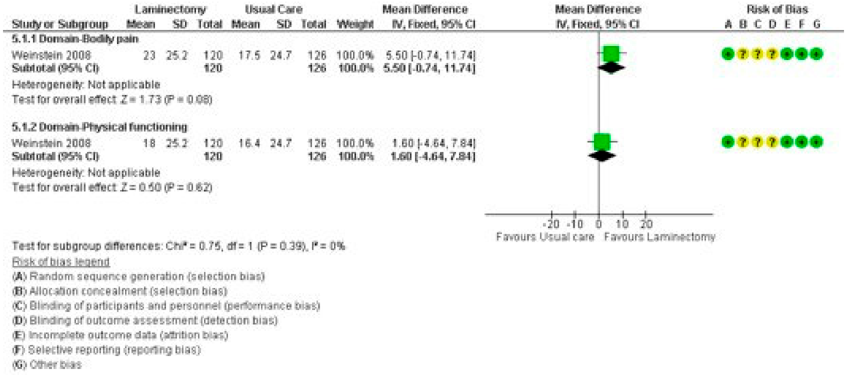

Figure S86. Forest plot of comparison: 5 Laminectomy vs. usual care, Outcome: 5.2 Pain severity (Low Back Pain Bothersomeness Index, 0-24) > 4 months to 1 year.

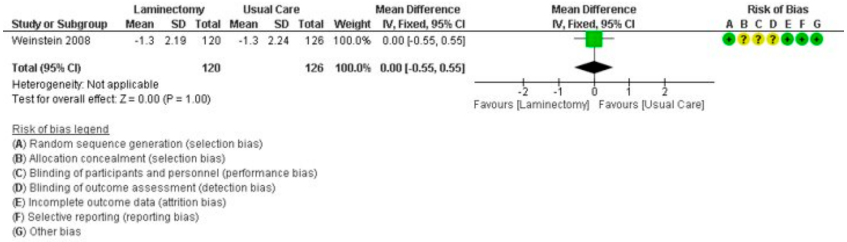

Figure S87. Forest plot of comparison: 5 Laminectomy vs. usual care, Outcome: 5.3 Pain severity (Sciatica Bothersomeness Index, 0-24) > 4 months to 1 year.

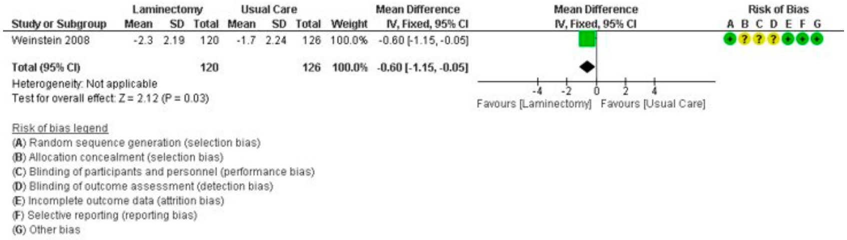

Figure S88. Forest plot of comparison: 5 Laminectomy vs. usual care, Outcome: 5.4 Function (ODI, 0-100, change scores) > 4 months to 1 year.

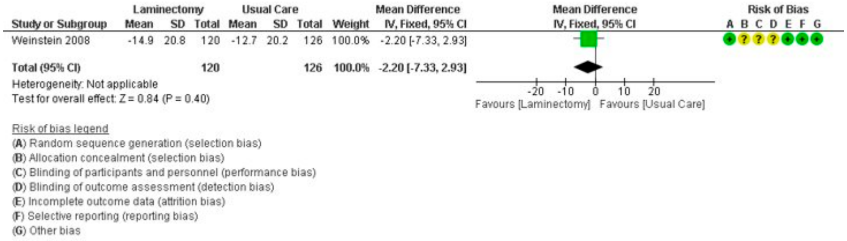

Figure S89. Forest plot of comparison: 5 Laminectomy vs. usual care, Outcome: 5.8 Adverse events: Mortality.

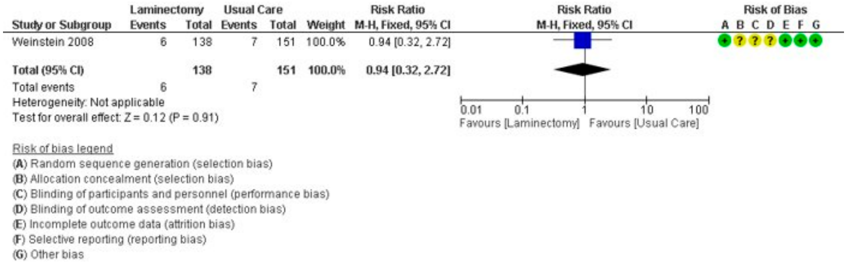

Revision rate - not reported.

Failure rate - not reported.

Question 10. Should radiofrequency denervation for facet joint pain versus placebo or usual care, or versus other treatments, be used in patients with non-specific LBP?

Figure S90. Forest plot of comparison: 1. Radiofrequency denervation vs. placebo/sham, Outcome 1: Pain (VAS) 0-10.

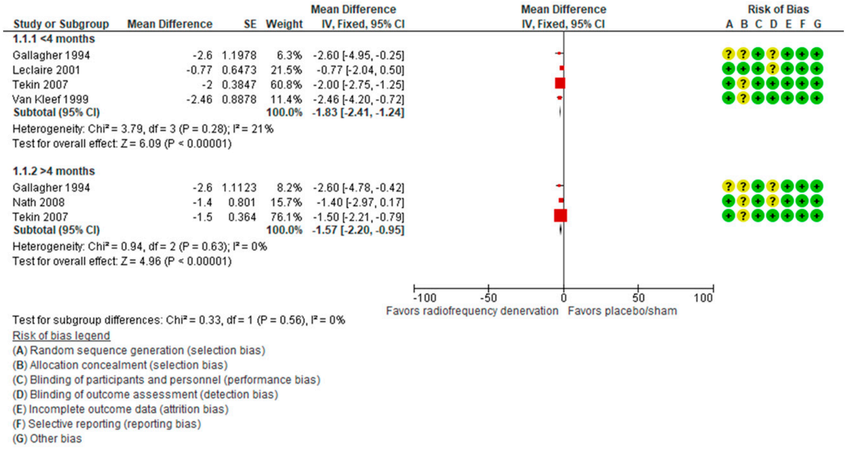

Figure S91. Forest plot of comparison: 1. Radiofrequency denervation vs. placebo/sham, Outcome 2: Function (RMDQ, 0-100 change; positive value = improvement) ≤ 4 months.

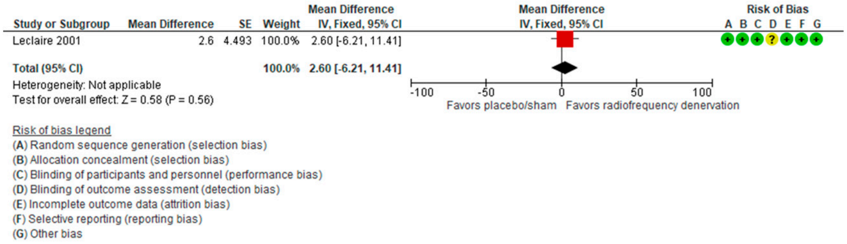

Figure S92. Forest plot of comparison: 1. Radiofrequency denervation vs. placebo/sham, Outcome 3: Quality of life (SF-36) ≤ 4 months.

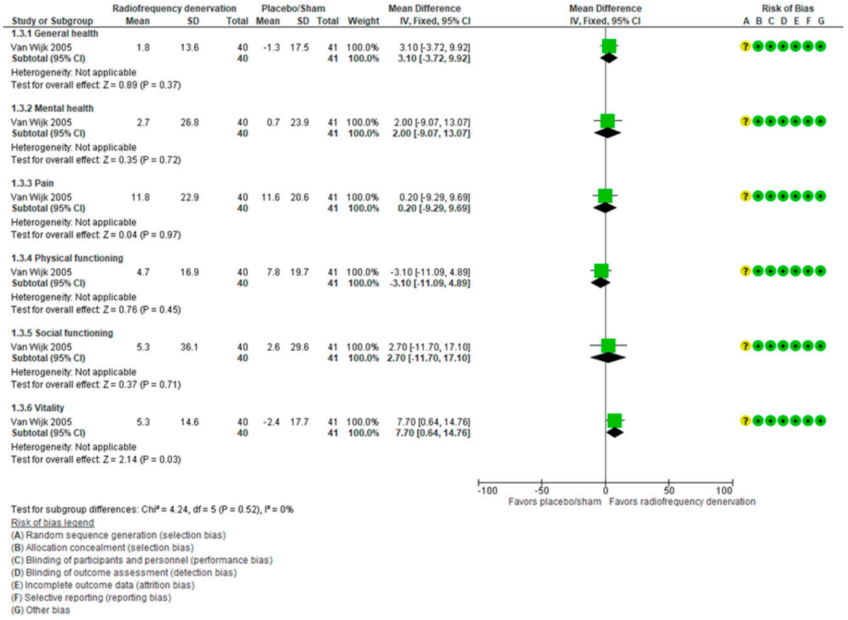

Figure S93. Forest plot of comparison: 1. Radiofrequency denervation vs. placebo/sham, Outcome 4: Adverse events ≤ 4 months).

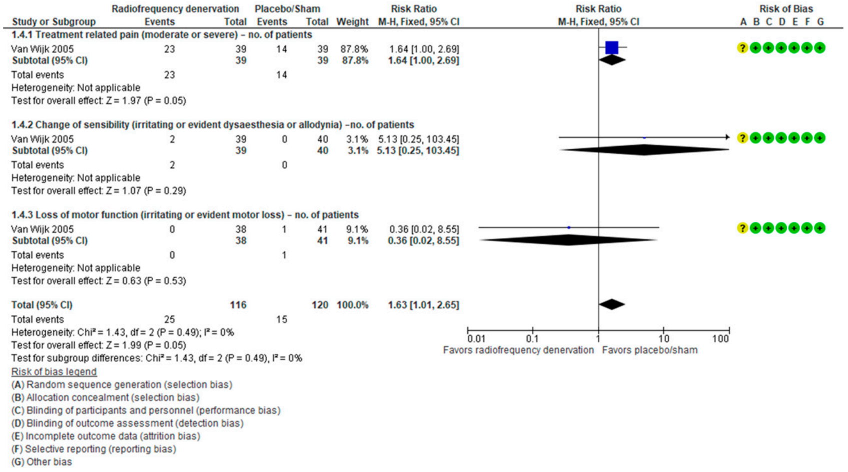

Figure S94. Forest plot of comparison: 1. Radiofrequency denervation vs. placebo/sham, Outcome 5: Healthcare utilization.

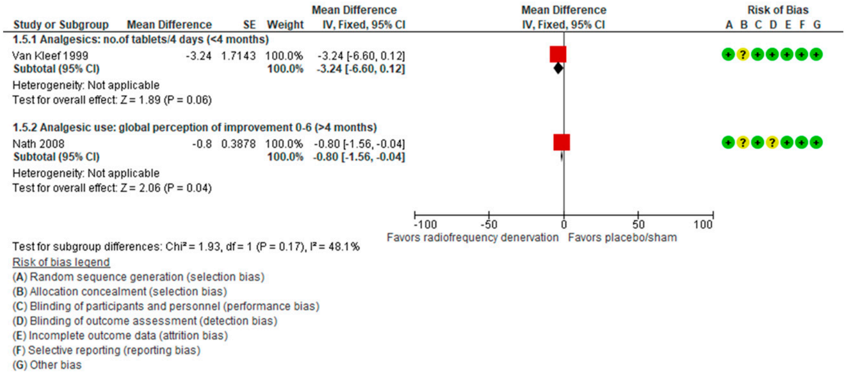

Figure S95. Forest plot of comparison: 1. Radiofrequency denervation vs. placebo/sham, Outcome 6: Responder criteria (number of patients with more than 50% back pain or pain reduction – global perceived effect).

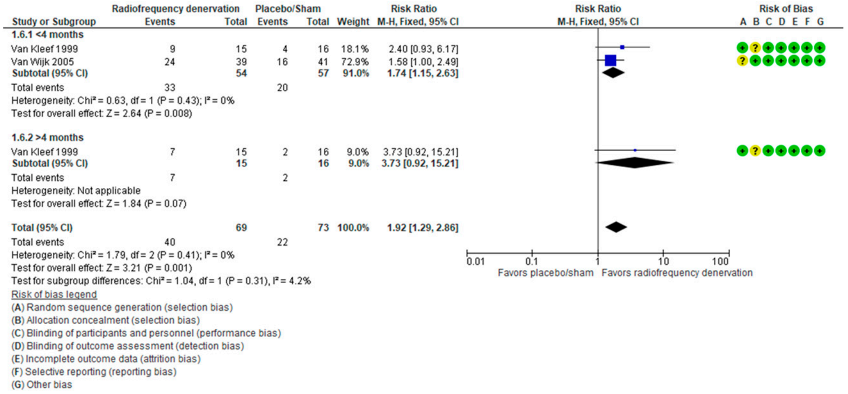

Figure S96. Forest plot of comparison: 2. Radiofrequency denervation vs. medial branch block, Outcome 1: Pain (VNS) 0-10.

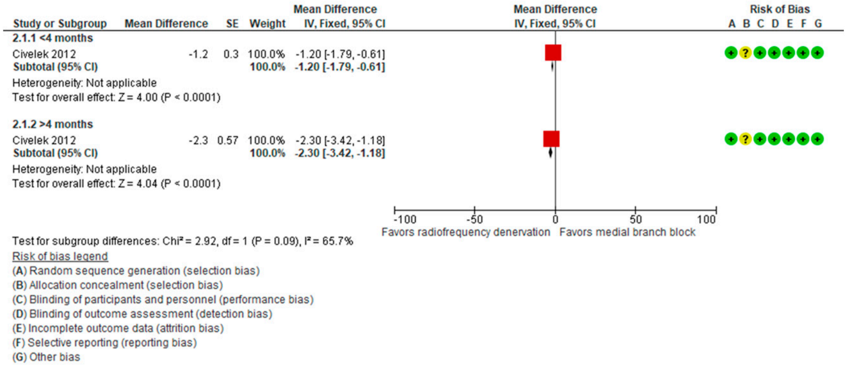

Figure S97. Forest plot of comparison: 2. Radiofrequency denervation vs. medial branch block, Outcome 2: Quality of life (EQ-5D) 5-15 scale (low score = better).

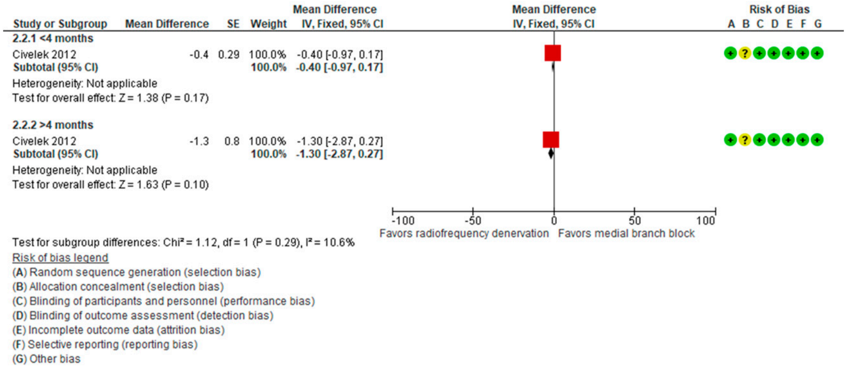

Psychological distress (as measured by the Hospital Anxiety and Depression Scale [HADS], General Health Questionnaire [GHQ], Beck Depression, Inventory [BDI], Brief Pain Inventory [BPI], or the State-Trait Anxiety Inventory [STAI]) – not reported.

Question 11. Should pain neuroscience education versus no pain neuroscience education be used to reduce disability, pain and recurrence in patients with chronic non-specific LBP?

Figure S98. Forest plot of comparison: 1 Pain neuroscience education vs. no pain neuroscience education, outcome: 1.1 Pain - short term (follow up: mean 31.8 days; NRS, 0-10).

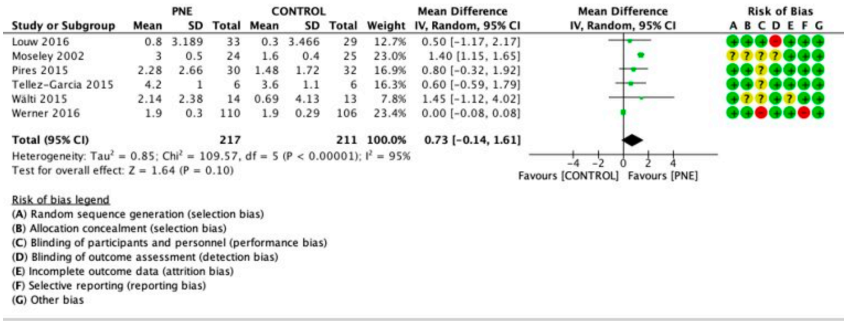

Figure S99. Forest plot of comparison: 1 Pain neuroscience education + intervention vs. pain neuroscience education outcome: 1.2 Pain short-term (PNE+INTERVENTION) (follow up: mean 32.6 days; assessed with: NRS; Scale from: 0 to 10).

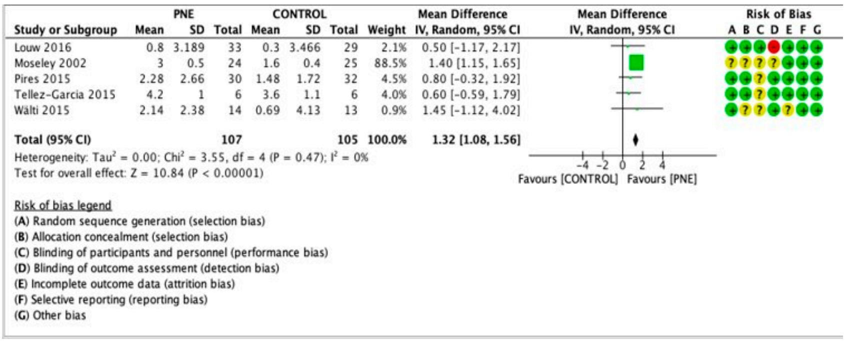

Figure S100. Forest plot of comparison: 1 Pain neuroscience education vs. no pain neuroscience education , outcome: 1.3 Pain in long term (12 month) (assessed with: NRS; Scale from: 0 to 10).

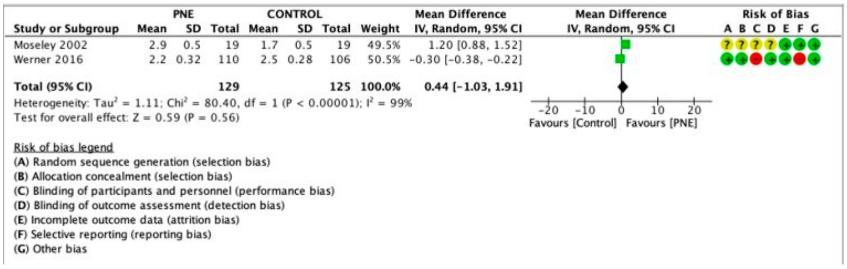

Figure S101. Forest plot of comparison: 1 Pain neuroscience education vs. no pain neuroscience education, outcome: 2.1 Disability short-term (follow up: mean 32.8 days; assessed with: RMDQ; Scale from: 0 to 24).

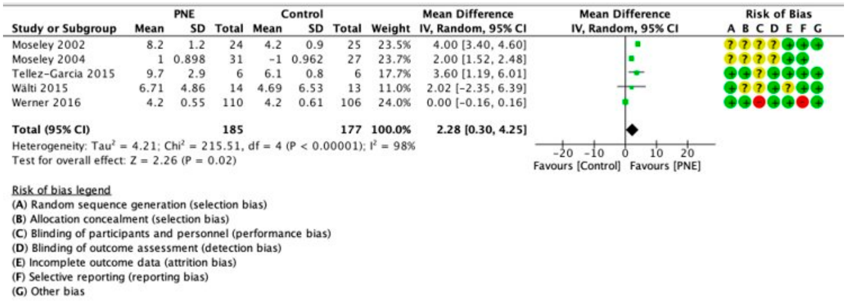

Figure S102. Forest plot of comparison: 1 Pain neuroscience education + intervention vs. no pain neuroscience education, outcome: 2.2 Disability (Intervention +PNE) (follow up: mean 40 days; assessed with: RMDQ; Scale from: 0 to 24).

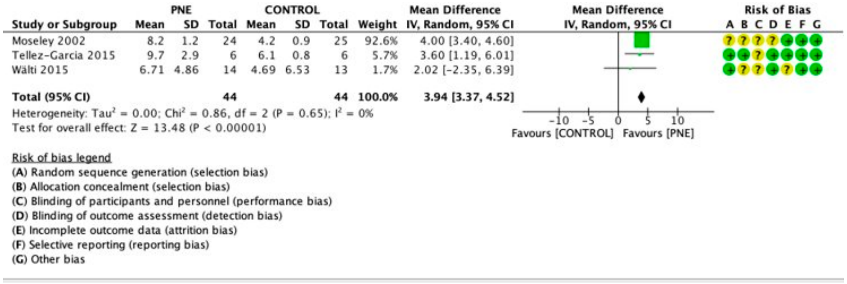

Figure S103. Forest plot of comparison: 1 Pain neuroscience education vs. no pain neuroscience education, outcome: 2.3 Disability in long -term (follow up: mean 12 months; assessed with: RMDQ; Scale from: 0 to 24).

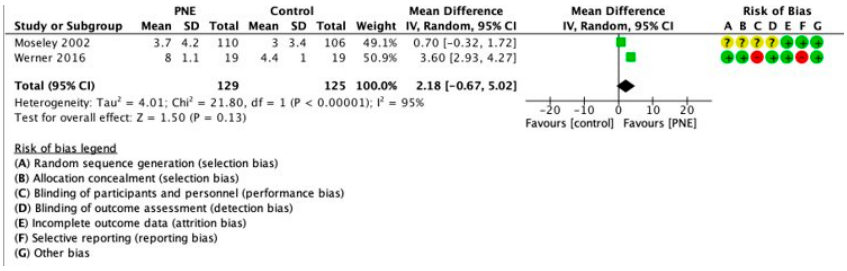

Figure S104. Forest plot of comparison: 1 Pain neuroscience education vs. no pain neuroscience education, outcome: 3.1 Psychological effects (follow up: mean 3.33 weeks; assessed with: Tampa Scale of Kinesiophobia; Scale from: 13 to 52).

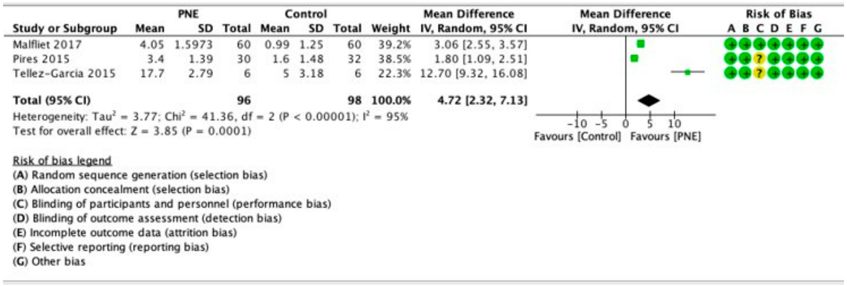

Supplement: Supplementary file 1 [file jcm-15-00528-s001.zip › Supplementary Material S2.pdf]
