# Supplementary material for: Saudi Clinical Practice Guideline for the Assessment and Management of Low Back Pain and Sciatica in Adults
Source: J Clin Med. 2026 Jan 8;15(2):528. doi: 10.3390/jcm15020528 (PMC12842004; doi:10.3390/jcm15020528)
Supplement: Supplementary file 1 [file jcm-15-00528-s001.zip › Supplementary Material S3.pdf]

Supplementary Material S3: Risk Assessment Tools and Stratification

The following table was taken from source NICE guideline NG59 [37] and describes the risk tool contents identified from studies included in its review:

Tool	Number of items	Description
Chronic Pain Risk Item Set [125]	22	A score derived from an original Chronic Pain Risk Score, a tool assessing pain intensity, pain interference with activities, number of activity limitation days due to pain, pain persistence in the past 6 months, a depressive symptom scale, and the number of painful anatomic sites. The simplified Chronic Pain Risk Item Set includes:<br>● 3 items on back pain intensity (scored on a 0-10 scale)<br>● Average/usual pain<br>● Worst pain<br>● Pain right now<br>● 3 items on back pain-related activity interference (scored on a 0-10 scale)<br>● Interference with usual activities<br>● Interference with social and family activities o Interference with work or housework activities<br>● 1 item on back pain persistence (back pain days in the prior 6 months)<br>● 7 items from the Pain Health Questionnaire (PHQ)-15, assessing an expanded number of pain sites and pain bothersomeness rating for each site (response format: not bothered at all, bothered a little, bothered a lot; score obtained by summing all ratings)<br>● Back pain<br>● Stomach pain<br>● Pain in arms, legs, or joints<br>● Headaches<br>● Neck pain<br>● Pelvic/groin pain<br>● Widespread pain<br>● 8 items from the Pain Health Questionnaire (PHQ)-8 to assess depressive symptoms severity.
Eleven-Item version of the Tampa Scale of Kinesiophobia (TSK-11) [126]	11	11-item questionnaire derived from an original 17-item Tampa Scale of Kinesiophobia. TSK 11 evaluates the degree of fear on movement and injury or re-injury in individuals with low back pain. Items are scored from 1 (strongly disagree) to 4 (strongly agree). Potential scores range 11- 44, with higher scores indicating greater fear on movement and injury or re-injury due to pain.
Fear Avoidance Beliefs Questionnaire (FABQ) [126]	4+7	A questionnaire assessing fear avoidance beliefs specific to low back pain. It consists of a 4-item physical activity scale (FABQ-PA, score range 0-24) and a 7-item work scale (FABQ-W, score range 0-42). 4-item physical activity scale (FABQ-PA) statements:<br>● Physical activity makes my pain worse<br>● Physical activity might harm my back<br>● I should not do physical activities which (might) make my pain worse<br>● I cannot do physical activities which (might) make my pain worse<br>7-item work scale (FABQ-W) statements:<br>● My pain was caused by my work or by an accident at work<br>● My work aggravated my pain<br>● My work is too heavy for me<br>● My work makes or would make my pain worse<br>● My work might harm my back<br>● I should not do my normal work with my present pain

		● I do not think that I will be back to my normal work within 3 months.<br>Each item is scored on a 'completely disagree' (0) – 'unsure' (3) – 'completely agree' (6) scale. Total score for each subscale is calculated as the total sum of scores of all items in that subscale. Higher levels indicate higher levels of fear avoidance beliefs. (available from bpb-us-w2.wpmucdn.cohttps://bpb-us-w2.wpmucdn.com/sites.udel.edu/dist/c/3448/files/2023/01/LowBack.pdf; last accessed 10/26/2023)
Hancock CPR (clinical prediction rule) [127]	3	A 3-item clinical prediction rule for the identification of patients with acute low back pain (within 12 weeks of symptom onset), presenting to primary care, likely to recover rapidly from acute low back pain.<br>● Baseline pain. Feature associated with a more rapid recovery: ≤7/10 on numerical pain rating scale<br>● Duration of current symptoms. Feature associated with a more rapid recovery: ≤5 days<br>● Number of previous episodes of low back pain. Feature associated with a more rapid recovery: ≤1previous episodes<br>Status on the prediction rule is determined by calculating the number of predictors of recovery present. On the basis of the number of positive features present (0, 1, 2, or 3 features positive), each patient can be assigned to one of 4 strata, representing their status on the prediction rule. (Hancock MJ et al. Can rate of recovery be predicted in patients with acute low back pain? Development of a clinical prediction rule. European Journal of Pain 2009; 13:51-55)
Low back pain perception scale [45]	5	A scale on low back pain perception containing a total of 5 items:<br>● Worrying<br>● Coping<br>● Limitations due to low back pain<br>● Expectation regarding pain relief<br>● Pain interference.<br>All items have a yes/no response format; the total score is derived by totalling number of 'yes' responses. Higher scores indicate greater risk.
Nine-Item Patient Health Questionnaire (PHQ-9) [126]	9	A 9-item questionnaire used to assess degree to which depressive symptoms have on a patient with low back pain (scores range from 0-27).<br>● Little interest or pleasure in doing things<br>● Feeling down, depressed or hopeless<br>● Trouble falling or staying asleep, or sleeping too much<br>● Feeling tired or having little energy<br>● Poor appetite or overeating<br>● Feeling bad about yourself – or that you are a failure or have let yourself or your family down<br>● Trouble concentrating on things, such as reading the newspaper or watching television<br>● Moving or speaking so slowly that other people could have noticed, or being so fidgety or restless that you have been moving around a lot more than usual<br>● Thoughts that you would be better off dead or of hurting yourself in some way.<br>Each item is scored on a 'not at all' (0) – 'nearly every day' (3) scale. A total score is calculated by adding up responses to all items. High scores indicate elevated depressive symptoms (major depression is diagnosed if ≥ 5 depressive symptom criteria have been present more than half the days in the past 2 weeks and one of the symptoms is depressed mood or anhedonia). (available from http://www.ncbi.nlm.nih.gov/pmc/articles/PMC1495268/pdf/jgi_01114.pdf; last accessed 10/26/2023)
Örebro Musculoskeletal Screening Questionnaire (ÖMSPQ, modified version of ÖMSPQ) [44]	25 (21)	The Örebro Musculoskeletal Screening Questionnaire (ÖMSPQ) is a modified version of the original Örebro Musculoskeletal Pain Questionnaire (ÖMSPQ). Four critical characteristics of the original questionnaire are retained in the ÖMSPQ: question number and order, scoring format and total score. All scored 21 ÖMSPQ items are included in the ÖMSPQ, with one being renamed and 4 additional ADL being combined with the physical function questions.

		1. Region. Where do you have your pain/problem? Back or neck, arm, leg, both sides, several body areas.<br>2. Absenteeism. Due to your pain/problem, how many days of work or 'normal daily routine' have you missed? '0 days' (1), '1-2 days' (2), '3-7 days' (3), '8-14 days' (4), '15-28 days' (5), '1 month' (6), '2 months' (7), '3-6 months' (8), '6-12 months' (9), 'over 1 year' (10)<br>3. Duration. How long have you had your current pain/problem? '0-1 weeks' (1), '1-2 weeks' (2), '3-4 weeks' (3), '4-5 weeks' (4), '6-8 weeks' (5), '9-11 weeks' (6), '3-6 months' (7), '6-9 months' (7), '9-12 months' (9), 'over 1 year' (10)<br>4. Burdensome. Do you feel your work or normal daily routine is a burden to you (eg heavy or monotonous)? 'Not at all' (0) – 'extremely' (10)<br>5. Intensity acute. How would you rate your pain/problem during the past week, or since the injury if less than a week ago? 'No pain/problem' (0) – 'worst possible' (10)<br>6. Severity chronic. Since your injury (or in the past 3 months if it is not a recent injury), in general, how has your pain/problem been? 'No pain/problem' (0) – 'worst possible' (10)<br>7. Frequency. Since your injury (or in the past 3 months if it is not a recent injury), in general, how often is your pain/problem present? 'Never' (0) – 'all the time' (10)<br>8. Coping. Over the last week, or since the injury if it were less than a week ago, on an average day, how well can you cope with or control your pain/problem? 'Not at all' (0) – 'completely' (10)<br>9. Anxiety. Over the last week or since the injury if it were less than a week ago, on an average day, how tense or anxious have you felt? 'Not at all' (0) – 'extremely' (10)<br>10. Depression. Over the last week or since the injury if it were less than a week ago, on an average day, how depressed or 'down' have you felt? 'Not at all' (0) – 'extremely ' (10)<br>11. Recovery expectation problem. In your view how large is the risk that your current pain/problem may become persistent? 'No risk' (0) – 'very large risk' (10)<br>12. Recovery expectation work. What are the chances you will be doing your work or normal daily routine in 6 months' time? 'No chance' (0) – 'very large chance' (10)<br>13. Job satisfaction. How satisfied are you with your current life situation (work/normal daily routine, home, friends)? 'Not at all' (0) – 'completely' (10)<br>14. Fear-avoid: activity. Physical activity makes my pain/problem worse. 'Completely disagree' (0) – 'Completely agree' (10)<br>15. Fear-avoid: stop work. An increase in my pain/problem tells me I should stop what I am doing until my pain/problem decreases. 'Completely disagree' (0) – 'Completely agree' (10)<br>16. Fear-avoid: not work. I should not do my work or normal daily routine with my present pain/problem. 'Completely disagree' (0) – 'Completely agree' (10)<br>17. Light work/chores. I can manage light work for up to an hour (eg lift, carry or move light objects < 5 kg). 'Not at all' (0) – 'completely' (10)<br>18. Walk/recreation. I can walk for an hour or participate in my normal light recreational or sporting activities. 'Not at all' (0) – 'completely' (10)<br>19. Home activity. I can manage my regular home activities and chores (cleaning, steps, use a chair, family duties, etc). 'Not at all' (0) – 'completely' (10)<br>20. ADL and social. I can manage my regular daily routine and social activities (shopping, transport or seeing friends). 'Not at all' (0) – 'completely' (10)<br>21. Sleep/move in bed. I can sleep at night or move normally in bed. 'Not at all' (0) – 'completely' (10)<br>Items are rated 0 to 10 points where higher scores indicate increased risk. Scores for items 8, 12, 13, and 17 to 21 are reversed and calculated as (10 - score). The

		item assessing pain sites is scored by counting the number of pain sites and multiplying by 2. The total score is calculated as the total sum of scores of all items (score range: 0-210), with high scores indicating an increased risk of poor outcome. Cut-off ranges in ÖMSPQ are used to indicate low (<95), moderate (95-112), and high (>112) risk of delayed recovery from low back pain.
Örebro Musculoskeletal Pain Questionnaire (ÖMPQ, Acute Low Back Pain Screening Questionnaire) [44,45,128–130]	25 (21)	25-questions questionnaire, of which 21 are scored on a 0-10 points response scale. The 21 scored items assess 5 proposed constructs: function, pain, psychological (mood, perceptions of work, patients' estimate of prognosis), fear avoidance and miscellaneous.<br>● Items 1–3 concern the number of regions of the body affected by pain, the duration of pain and the duration of sick leave from work in the previous 18 months because of pain.<br>● Items 4 and 13 focus on the patients' perception of their work (is their work heavy, are they satisfied with their job).<br>● Items 5–8 assess the patient's perception of pain (current pain intensity, average pain intensity, pain frequency) and coping strategies (control over pain).<br>● Items 9–12 assess the patient's feelings of anxiety, depression, their perception of pain becoming chronic and their chance of getting back to work in a 6-months' time.<br>● Items 14–16 involve fear avoidance beliefs and behaviours in response to pain.<br>● Items 17–21 focus on activities of daily living (light working, walking, household work, shopping, sleeping).<br>Items are rated 0 to 10 points where higher scores indicate increased risk. Scores for items 8, 12, 13, and 17 to 21 are reversed and calculated as (10 - score). The item assessing pain sites is scored by counting the number of pain sites and multiplying by 2. The total score is calculated as the total sum of scores of all items (score range: 0-210), with high scores indicating an increased risk of poor outcome. Cut-off ranges in ÖMSPQ are used to indicate low (90-100) and high (105-119) risk of prolonged recovery from low back pain.<br>Some authors (164) (130) use different risk thresholds (based on sensitivity and specificity thresholds and Linton & Hallden 1998): low risk (score <90), moderate risk (score 90-105), and high risk for prolonged disability (score > 105). (available from http://occmed.oxfordjournals.org/content/58/6/447.full.pdf; last accessed 10/26/2023)
Pain Catastrophizing Scale [126]	13	A 13-item questionnaire (score range 0-52) assessing the degree of catastrophic cognitions due to low back pain.<br>● I worry all the time about whether the pain will end (helplessness)<br>● I feel I can't go on (helplessness)<br>● It's terrible and I think it's never going to get any better (helplessness)<br>● It's awful and I feel that it overwhelms me (helplessness)<br>● I feel I can't stand it anymore (helplessness)<br>● I become afraid that the pain will get worse (magnification)<br>● I keep thinking of other painful events (magnification)<br>● I anxiously want the pain to go away (rumination)<br>● I can't seem to keep it out of my mind (rumination)<br>● I keep thinking about how much it hurts (rumination)<br>● I keep thinking about how badly I want the pain to stop (rumination)<br>● There is nothing I can do to reduce the intensity of the pain (helplessness)<br>● I wonder whether something serious may happen (magnification)<br>Each item is scored on a 'not at all' (0) – 'all the time' (5) scale. The total score is calculated by adding up responses to all items. Higher scores indicate higher levels of pain catastrophizing. Three subscales (PCS rumination, PCS magnification, PCS helplessness) scores are computed by summing up the responses to the relevant items (Sullivan MJL, Bishop SR, Pivik J. The Pain Catatrophizing Scale: Development and validation. Psychological Assessment 1995; 7(4):524-532.)
Spinal manipulation clinical	5	A clinical prediction rule for the identification of patients with low back pain who are likely to benefit from a manipulation intervention (achieving at least 50%

prediction rule [131]		improvement in disability within 1 week with a maximum of 2 manipulation interventions). It contains 5 criteria:<br>● Duration of current episode of low back pain. Definition of positive outcome: < 16 days<br>● Extent of distal symptoms (assessed with a body diagram; distribution is categorized as being in the back, buttock, thigh or leg (distal to the knee) as described by Werneke et al, Spine 1993). Definition of positive outcome: no symptoms extending distal to the knee<br>● FABQ (Fear Avoidance Beliefs Questionnaire) work subscale score (7 items with potential score range 0-42; higher scores representing increased fear avoidance beliefs). Definition of positive outcome: < 19 points<br>● Segmental mobility testing (tested over the spinous processes of the vertebrae with the patient prone and the neck in neutral rotation. The examiner applies a gentle but firm, anteriorly directed pressure with their hand on the spinous process and assesses a segment as normal, hypomobile, or hypermobile on the basis of their anticipation of what normal mobility would feel like at that level, compared with the mobility detected in the segments above and below). Definition of positive outcome: ≥ 1 hypomobile segment in the lumbar spine<br>● Hip internal rotation range of motion (tested bilaterally with the patient lying prone and with the cervical spine at the midline. The leg opposite that to be measured is placed in approximately 30 degrees of hip abduction, to enable the tested hip to be freely moved. The lower extremity of the side to be tested is kept in line with the body, and the knee on that side is flexed to 90 degrees. A gravity inclinometer is placed on the distal aspect of the fibula in line with the bone. Internal rotation is measured at the point in which the pelvis first begins to move). Definition of positive outcome: ≥ 1 hip with > 35 degrees of internal rotation range of motion. A threshold of ≥4 criteria identifies a positive outcome and < 3 a negative outcome, based on Flynn et al (2002).
STarT Back Screening Tool (SBT) [42,126,132]	9	A 9-item questionnaire about physical and psychosocial predictors of back pain used to categorize patients with Low Back Pain in primary care settings, based on risk for poor disability outcomes. It has been translated into several languages and has cross-cultural validity.<br>9 Items:<br>● Radiating leg pain<br>● Pain elsewhere (shoulder or neck)<br>● Disability (walking)<br>● Disability (self-care)<br>● Fear<br>● Anxiety<br>● Pessimistic patient expectations<br>● Low mood<br>● Bothersomeness Each item is scored dichotomously, either 0 or 1.<br>All items have a 'disagree' (0)/'agree' (1) response format, except from the bothersomeness item, which has a 'not at all' (0)/ 'slightly' (0)/ 'moderately' (0)/ 'very much' (1)/ 'extremely' (1) response format.<br>Two scores are finally calculated:<br>● SBT overall score (0-9): determined by the sum of all positive responses.<br>● SBT psychosocial subscale score (0-5): determined by the sum of all items related to fear, anxiety, catastrophizing, depression and bothersomeness.<br>On the basis of both scores, patients are categorized into 3 groups:<br>● SBT high risk group (overall score ≥4): high levels of psychosocial prognostic factors are present with or without physical factors present,<br>● SBT medium risk group (overall score >3, psychosocial subscale score <4): physical and psychosocial factors are present but not a high levels of psychosocial factors,<br>● SBT low risk group (overall score 0-3): few prognostic factors are present.<br>When SBT is administered at 2 time points (cf Beneciuck et al 2014, SBT administered at intake and after 4 weeks), a SBT change categorization may be used

		to describe the variation in the patients' SBT overall score (determined by summing all positive responses, 0-9) over time:<br>● Improved: SBT risk categorization changed from medium to low, high to low or high to medium risk<br>● Stable: SBT risk categorization remained low or medium risk<br>● Worsened: SBT risk categorization changed from low to medium, low to high, medium to high, or remained high risk.<br>(available from https://startback.hfac.keele.ac.uk/wp-content/uploads/2022/02/Keele_STarT_Back9_item.pdf; last accessed 10/20/2023)

Other assessment tools that have been preliminarily tested in small studies for Saudi Arabia include the Japanese Orthopedic Association Back Pain Evaluation Questionnaire [133], the Tunisian version of the Oswestry Disability Index (ODI) [134], the Pain Behavior Scale (PaBS) in the context of chronic low back pain [135], the Leeds Assessment of Neuropathic Symptoms and Signs (LANSS) pain scale [136] and the Pain Self-Efficacy Questionnaire (PSEQ-A, chronic low back pain) [137].
