# Supplementary material for: Saudi Clinical Practice Guideline for the Assessment and Management of Low Back Pain and Sciatica in Adults
Source: J Clin Med. 2026 Jan 8;15(2):528. doi: 10.3390/jcm15020528 (PMC12842004; doi:10.3390/jcm15020528)
Supplement: Supplementary file 1 [file jcm-15-00528-s001.zip › Supplementary Material S4.pdf]

Supplementary Material S4: Cost Tables

Intervention	Applicable costs
Spine MRI (with or without contrast)	2000 SAR
Cervical spine MRI (with or without contrast)	1500 SAR
Lumbar spine MRI (with or without contrast)	1555 SAR
Cervical spine or lumbosacral CT (without contrast)	720 SAR
Cervical spine lumbosacral CT (with contrast)	960 SAR
Lumbar X-ray	156 SAR
Nerve conduction study (one nerve)	150 SAR
Table 9 in the study by Bargi et al, 2015 [138] contains the distribution of the total cost by radiology type.	
Cognitive therapy (1 session)	800 SAR
Physical therapy: core muscle strengthening exercises	100 SAR
Physical therapy: core muscle stretching	80 SAR
Ultrasound for pain relief (one area)	50 SAR
Extracorporeal shock wave therapy (one area) for pain relief	3300 SAR
Exploration and decompression of the spinal canal using laminectomy (three levels)	9900 SAR
Exploration and decompression of the spinal canal using discectomy, spinal fusion	25000 SAR

CT: computerized tomography; MRI: magnetic resonance imaging; SAR: Saudi Arabian Riyal.

Scientific name	Trade name	Strength	Dosage form	Applicable costs
**Anesthetic Agents**				
Propofol	Recofol	10 mg/ml	Solution for injection	101.4 SAR
	Propoven	1%	Solution for injection	102.9 SAR
	Propofol-Lipuro	1%	Solution for injection or infusion	68.95 SAR
Ketamine	Tekam	10 mg/ml	Solution for injection	6.5 SAR
	Tekam	50 mg/ml	Solution for injection	26.55 SAR
Fentanyl	Fentanyl Medis	10 ml	Solution for injection	63.8 SAR
	Fentanyl Medis	2 ml	Solution for injection	25.85 SAR
	Fentanyl-Janssen	0.05 mg/ml	Solution for injection	49.65 SAR
	Fentanyl Piramal	0.05 mg/ml	Solution for injection	93.75 SAR
Bupivacaine	Bucaine	0.5 mg/ml, 0.25 mg/ml	Solution for injection	16.95 SAR
	Marcaine	0.5 mg/ml	Solution for injection	77.8 SAR
	Betolina	5 mg/ml	Solution for injection	58.7 SAR
Ropivacaine	Naropin	10 mg/ml	Solution for injection	144.9 SAR
	Naropin	7.5 mg/ml	Solution for injection	121.65 SAR
	Naropin	2 mg/ml	Solution for injection	67.1 SAR
**Muscle relaxants**				

Succinylcholine	Succinyl Siccum	500 mg	Solution for injection	79.55 SAR
Suxamethonium chloride	Lysthenon Siccum	500 mg	Solution for injection	119.45 SAR
Rocuronium	Esmeron	10 mg/ml	Solution for injection	215.05 SAR
**Analgesics**				
Morphine	Oramorph	20 mg/ml	Oral Solution	30.35 SAR
	MST Continus	10 mg	Extended-release tablet	47.05 SAR
	MST Continus	30 mg	Extended-release tablet	116.2 SAR
	MST Continus	60 mg	Extended-release tablet	218.9 SAR
	Morphine sulphate	10 mg/ml	Solution for injection	40.7 SAR
Hydromorphone	Jurnista	8 mg	Extended-release tablet	155.3 SAR
	Jurnista	16 mg		245.4 SAR
	Jurnista	32 mg		453 SAR
Paracetamol	Paramol	500 mg	Tablet	3.95 SAR
Diclofenac	Voltaren retard	100 mg	Extended-release tablet	27.3 SAR
	Olfen	100 mg	Capsule	24.55 SAR
	Rapidus	25 mg	Film-coated tablet	17.15 SAR
	Diclogesic	12.5 mg	Suppository	4.6 SAR
Ketorolac	Ketothrom	30 mg	Solution for injection	35.2 SAR
Dexamethasone	Examethasone sodium phosphate	4 mg	Solution for injection	8.55 SAR
**Non-steroidal anti-inflammatory drugs**				
Diclofenac potassium	Diclopid	50 mg	Film-coated tablet	9.1 SAR
Diclofenac sodium	Diclomax	50 mg	Tablet	19.8 SAR
	Rofenac	100 mg	Suppository	15.85 SAR
	Voltic	25 mg	Film-coated tablet	14.7 SAR
Caffeine, ibuprofen	Profinal xp	65 mg, 400 mg	Tablet	11.05 SAR
Ibuprofen	Profinal	400 mg	Film-coated tablet	12.05 SAR
	Fenbid spansules	300 mg	Extended-release capsule	18.2 SAR
	Fenbid spansules	300 mg	Extended-release capsule	48.75 SAR
	Brufen	600 mg	Tablet	19.55 SAR
	Brufen	400 mg	Tablet	13.6 SAR
	Nurofen	100 mg/5 ml	Suspension	15.3 SAR
Meloxicam	Neoxicam	7.5 mg	Tablet	5.15 SAR
	Neoxicam	15 mg	Tablet	7.75 SAR
	Coxicam	7.5 mg	Tablet	24.3 SAR
	Coxicam	7.5 mg	Tablet	10.85 SAR
	Coxicam	15 mg	Tablet	13.3 SAR
	Meloxi	7.5 mg	Tablet	13.9 SAR
	Meloxi	15 mg	Tablet	20.9 SAR
	Oximal	7.5 mg	Tablet	19.65 SAR
	Oximal	7.5 mg	Tablet	8.7 SAR
	Oximal	15 mg	Tablet	9.9 SAR
	Mobic	15 mg	Tablet	14.8 SAR
	Mobic	15 mg	Tablet	44.45 SAR
	Mobic	7.5 mg	Tablet	27 SAR
	Mobic	7.5 mg	Tablet	10.85 SAR

Etoricoxib	Arcoxia	120 mg	Tablet	18.6 SAR
	Arcoxia	60 mg	Tablet	37.2 SAR
	Arcoxia	60 mg	Tablet	74.4 SAR
	Arcoxia	90 mg	Tablet	74.4 SAR
	Arcoxia	90 mg	Tablet	37.2 SAR
**Local non-steroidal anti-inflammatory drugs**				
Ketoprofen	Fastum	2.5%	Gel	10.3 SAR
Diclofenac diethylamine	Voltaren emulgel	1%	Gel	26 SAR
Diclofenac sodium	Avotrene emulgel	50 g	Gel	7.2 SAR
	Avotrene emulgel	100 g	Gel	14.4 SAR
	Rofenac	1%	Gel	8.7 SAR
	Rofenac	1%	Gel	13.45 SAR
Diclofenac diethylamine	Rumafen	1%	Gel	11.75 SAR
Diclofenac diethylamine	Rumafen	1%	Gel	23.5 SAR
Gabapentin	Nulex	100 mg	Capsule	12.75 SAR
	Nulex	300 mg	Capsule	65.4 SAR
	Nulex	400 mg	Capsule	76.9 SAR
	Nulex	600 mg	Film-coated tablet	69.2 SAR
	Nulex	800 mg	Film-coated tablet	92.3 SAR
	Neurontin	400 mg	Hard capsule	128.1 SAR
	Neurontin	300 mg	Hard capsule	109.5 SAR
	Gapentin	400 mg	Capsule	105.5 SAR
	Gapentin	300 mg	Capsule	89.8 SAR
	Neuroplex	400 mg	Capsule	94.95 SAR
Vitamin b6 (pyridoxine hydrochloride) , vitamin b1 (thiamine hydrochloride) , vitamin b12 (cyanocobalamin)	Neurocomb	100 mg, 100 mg, 1 mg	Solution for intramuscular injection	10.1 SAR
Vitamin b1 (thiamine), vitamin b6 (pyridoxine), vitamin b12 (cyanocobalamin)	3v	100 mg, 100 mg, 1 mg	Solution for injection	8.6 SAR
Vitamin b1 (thiamine), vitamin b6 (pyridoxine), vitamin b12 (cyanocobalamin)	3v	100 mg, 200 mg, 0.2 mg	Tablet	12.4 SAR
Vitamin b12 (cyanocobalamin)	Jectin-12	1 mg	Solution for injection	7.35 SAR
Vitamin b6 (pyridoxine), vitamin b12 (cyanocobalamin), folic acid	Trio Be	0.5 mg, 3 mg, 0.8 mg	Tablet	10.75 SAR
Tamadol hydrochloride	Nomal	50 mg	Capsule	7.1 SAR
	Tramal	50mg	Capsule	34.95 SAR
	Tramal Retard	100 mg	Film-coated tablet	22.65 SAR
	TRAMAL 50 MG CAPSULE	50	Capsule	15.55 SAR
Tramadol hydrochloride, paracetamol	Zaldiar	37.5 mg, 325 mg	Film-coated tablet	27.1 SAR
Tizanidine	Tilax	2 mg	Tablet	16.4 SAR
	Tilax	4 mg	Tablet	26.9 SAR
	Sirdalud	2 mg	Tablet	18.75 SAR
	Sirdalud	4 mg	Tablet	30.7 SAR
	Relezin	4 mg	Tablet	24.2 SAR
	Relezin	2 mg	Tablet	14.75 SAR
TBaclofen	Lioresal	10 mg	Tablet	44.9 SAR
	Lioresal	25 mg	Tablet	90.65 SAR
	Baclofen Intrathecal Sintetica	0.05 mg/ml	Solution for injection	274.9 SAR
	Baclofen Intrathecal Sintetica	2 mg/ml	Solution for injection	2748.9 SAR

	Baclofen Intrathecal Sintetica	2 mg/ml	Solution for injection	1099.55 SAR
	Baclofen Intrathecal Sintetica	0.5 mg/ml	Solution for injection	1374.45 SAR
	Baclon	10 mg	Tablet	31.4 SAR
	Baclon	25 mg	Tablet	63.45
**Trigger point injection**				
Chlorzoxazone, paracetamol	Relaxon	250 mg, 300 mg	Capsule	15.9 SAR
Chlorzoxazone	Relaxon forte	500 mg	Tablet	16.2 SAR
Lornoxicam	Xefo	8 mg/ml	Powder for solution	15.95 SAR
Lornoxicam	Zenorit	8 mg/ml	Solution for injection	12 SAR
Lidocaine (lignocaine) hydrochloride, epinephrine	Xylocaine Dental + adrenaline	20 mg/ml, 12.5 mg/ml	Solution for injection	161.25 SAR
Methylprednisolone acetate	Depo medrol	40 mg/ml	Powder for solution	10.75 SAR
Methylprednisolone acetate	Depo medrol	40 mg/ml	Powder for solution	19.55 SAR

SAR: Saudi Arabian Riyal.

Broad Spectrum Antibiotics

**Scientific name**	**Trade Name**	**Strength**	**Dosage form**	**Applicable costs**
Cefazolin	Cefazolin	1 mg	Solution for injection	11.2 SAR
Azithromycin	Zencin	500 mg	Film-coated tablet	26.4 SAR
	Zocin	250 mg	Capsule	36.2 SAR
	Zerox	500 mg	Film-coated tablet	26.4 SAR
	Zerox	200 mg/5 ml	Powder for oral suspension	13.65 SAR
	Zimax	200 mg/5 ml	Powder for oral suspension	18 SAR
	Zocin	300 mg/7.5 ml	Suspension	15.15 SAR
Metronidazole	Metronidazole	5 mg/ml	Solution for injection	18.9 SAR
	Flagyl	250 mg	Tablet	10.05 SAR
	Flagyl	500 mg	Tablet	19.15 SAR
	Nidazole	5 mg/ml	Solution for injection	6.8 SAR
	Anazol	20 mg/5 ml	Suspension	10.05 SAR
	Anazol	125 mg/5 ml	Suspension	6.9 SAR
	Suprazole	200 mg	Tablet	6.65 SAR
	Furazol	250 mg, 200 mg	Tablet	11.4 SAR
	Amrizole	5 mg/1 ml	Solution for injection	6.85 SAR
	Metronidazole	5 mg/1 ml	Solution for injection	6.8 SAR
Clarithromycin	Klarimid	500 mg	Film-coated tablet	36.1 SAR
	Klarimid	250 mg	Film-coated tablet	22 SAR
	Klacid	125 mg/ml	Oral suspension	46.6 SAR
	Clarixin	500 mg	Tablet	46.05 SAR
	Claritt	500 mg	Film-coated tablet	56.8 SAR
	Claritt	250 mg/5 ml	Suspension	51.2 SAR
	Claritt xl	500 mg	Extended-release tablet	60.15 SAR
	Klacid	500	Powder for injection	54.9 SAR
	Klarimid	250 mg/5 ml	Suspension	55.05 SAR
Vancomycin	Vancomycin Alpharma	500 mg	Powder for solution	45.15 SAR
	Vancomycin Alpharma	1 g	Powder for solution	76.9 SAR
	Sterile Vancomycin	1 g	Solution for injection	340.3 SAR

	Sterile Vancomycin	500 mg	Solution for injection	180.25 SAR
Linezolid	Zyvox	2 mg/ml	Solution for injection	1601.75 SAR
	Zersa	2 mg/ml	Solution for infusion	136.9 SAR
	Zersa	600 mg	Film-coated tablet	1929.6 SAR
	Zersa	20 mg/ml	Oral suspension	630 SAR
	Dilizolen	600 mg	Tablet	1404.45 SAR
	Linid	2 mg/ml	Solution	196 SAR
	Zillion	600 mg	Film-coated tablet	1447.2 SAR
	Kinzado	600 mg	Film-coated tablet	632 SAR
	Lindec	600 mg	Film-coated tablet	1706 SAR
Piperacillin, tazobactam	Tazocin	4 mg, 0.5 mg	Powder for solution	58.15 SAR
Cefepime hydrochloride	Cefafour	1 g	Powder for solution	33.65 SAR
Ceftazidime	Zidime	1 g	Powder for solution	51.55 SAR
	Forta-Z	1 g	Powder for solution	38.95 SAR
	Zidime	2 g	Powder for solution	95.4 SAR
	Fortum	2 g	Powder for solution	477.55 SAR
	Fortum	1 g	Powder for solution	238.75 SAR
	Fortum	500 mg	Powder for solution	29.95 SAR
	Fortum	500 mg	Powder for solution	124.75 SAR
	Negacef	1 g	Solution for injection	43.3 SAR
	Negacef	2 g	Solution for injection	69.95 SAR
Meropenem Trihydrate	Meropenem	1 g	Powder for solution	356.4 SAR
	Meropenem	500 g	Powder for solution	211.95 SAR
Imipenem, Cilastatin	Imipenem-Cilastatin Labatec	500 g	Powder for solution	313.35 SAR
Oseltamivir	Oselow	75 mg	Capsule	71.85 SAR
	Tabuflu	75 mg	Capsule	82.4 SAR
	Oselta	75 mg	Capsule	91.55 SAR
	Tamiflu	75 mg	Capsule	79.85 SAR

SAR: Saudi Arabian Riyal.
